# Phenomics reveals a novel putative chloroplast fatty acid transporter in the marine diatom *Skeletonema marinoi* involved in temperature acclimation

**DOI:** 10.1038/s41598-019-51683-y

**Published:** 2019-10-22

**Authors:** Oskar N. Johansson, Mats Töpel, Jenny Egardt, Matthew I. M. Pinder, Mats X. Andersson, Anna Godhe, Adrian K. Clarke

**Affiliations:** 1 Department of Biological and Environmental Sciences, University of Gothenburg, Box 461, 40530 Gothenburg, Sweden; 2 Department of Marine Sciences, University of Gothenburg, Box 462, 40530 Gothenburg, Sweden; 3 Gothenburg Global Biodiversity Center (GGBC), Box 461, 40530 Gothenburg, Sweden

**Keywords:** Cell biology, Mutagenesis

## Abstract

Diatoms are the dominant phytoplankton in temperate oceans and coastal regions and yet little is known about the genetic basis underpinning their global success. Here, we address this challenge by developing the first phenomic approach for a diatom, screening a collection of randomly mutagenized but identifiably tagged transformants. Based upon their tolerance to temperature extremes, several compromised mutants were identified revealing genes either stress related or encoding hypothetical proteins of unknown function. We reveal one of these hypothetical proteins is a novel putative chloroplast fatty acid transporter whose loss affects several fatty acids including the two omega-3, long-chain polyunsaturated fatty acids - eicosapentaenoic and docosahexaenoic acid, both of which have medical importance as dietary supplements and industrial significance in aquaculture and biofuels. This mutant phenotype not only provides new insights into the fatty acid biosynthetic pathways in diatoms but also highlights the future value of phenomics for revealing specific gene functions in these ecologically important phytoplankton.

## Introduction

Phytoplankton are the primary producers in oceans that sustains most marine life. Of these, diatoms are the most diverse and ecologically successful of the eukaryotic lineages and are distinguished by their unique and highly ornamented silicified cell wall, or frustule^1^. As the most abundant of the phytoplankton, diatoms contribute to almost a quarter of the global photosynthetic CO_2_ fixation, as well as driving the biogeochemical cycles that sequester and mineralize carbon, nitrogen and silica^2^. Chain-forming diatoms play a particularly important role in these cycles, especially in forming fast-sinking aggregates that eventually sequester CO_2_ in ocean floor sediments. One of the most common genera of chain-forming diatoms is *Skeletonema*, which dominates coastal waters globally especially during spring blooms^3^. In Scandinavian waters, the species *S*. *marinoi* is a crucial primary producer throughout the year and can form dormant resting stages that survive in sediments for more than a century^4,5^.

Despite the global predominance of chain-forming diatoms, much remains unknown about the underlying genetic basis for their ecological success and how this might respond to future climate and habitat changes. Diatoms fundamentally differ from plants and other microalgae by having evolved from a secondary endosymbiotic event supposedly between a red alga and a heterotrophic flagellate^6^. Together with extensive horizontal gene transfers, this complex evolutionary history for diatoms has resulted in an unusual gene composition as revealed by whole-genome sequencing^7–11^. The broad diversity of gene sequences within diatom genomes along with a high proportion of genes encoding hypothetical proteins continue to be major obstacles for elucidating the precise function of genes and their role within cellular regulatory networks. A proven and informative genetic approach for investigating gene function is mutagenesis by establishing causal links between specific gene disruptions and observable phenotypic changes. On a genomic scale, large collections of randomly mutagenized transformants have been particularly successful in this regard and have provided a wealth of information on individual gene functions and associated pathways. For photosynthetic organisms, the tremendous success of such large collections is exemplified by the T-DNA insertion lines for *Arabidopsis thaliana*^12^ and more recently for the green, freshwater microalgae *Chlamydomonas reinhardtii*^13^.

We have recently taken on the challenge of establishing the first collection of randomly mutagenized lines for a marine diatom, that being for the centric species *S*. *marinoi*^14^. A PCR-based method was then developed to reliably map for the first time in a diatom the insertion site within each transformant using the recently sequenced genome for *S*. *marinoi* (http://cemeb.science.gu.se/research/target-species-imago/skeletonema-marinoi, Töpel *et al*. in preparation). Having proposed *S*. *marinoi* as a new genetic model for chain-forming diatoms^14^, we now test its suitability for the forward-genetics approach of identifying genes responsible for different ecologically important characteristics. In this study, we have screened over a hundred transformants for their ability to acclimate to low and high growth temperatures, of which six significantly differed from the wild type responses. Mapping of the disrupted gene in each of these transformants revealed a range of proteins with both predicted and unknown function. More detailed structural analysis of one of these hypothetical proteins revealed a putative chloroplast fatty acid (FA) transporter that appears unique to centric diatoms. It is the loss of this transporter and the subsequent changes in FA composition that not only compromises the mutant’s ability to tolerate cold temperatures but also improves acclimation to high temperatures. Indeed, the altered cellular FA composition within this mutant suggests that this unusual fatty-acid transporter acts upon eicosapentaenoic acid (EPA), an omega-3, long-chain polyunsaturated FA (LC-PUFA) of significant medical and biotechnological importance. This study highlights the fact that much of our understanding of FA biosynthesis in diatoms remains fragmentary, with features clearly distinct from those in other photosynthetic organisms. Moreover, it emphasizes the combined value of mutagenesis and phenomics as one of the most informative approaches to elucidating gene functions.

## Methods

### Culture conditions

The *S*. *marinoi* strain R05AC that was originally collected in 2010 from sediments in Öresund, Sweden (55°59.16 = N, 12°44.02 = E) was obtained for this study from the Gothenburg University algae bank (GUMACC, https://marine.gu.se/english/research/marine-biology/algal-bank). Cultures of *S*. *marinoi* R05AC were genetically transformed by multi-pulse electroporation as previously described to produce all the mutants used in this study^14^. All *S*. *marinoi* cultures were maintained in enriched growth media (EGM), which is a modified version of f/2^14^ using filtered, autoclaved sea water at 26 PSU, with the addition of the antibiotic zeocin (0.2 µg mL^−1^) for all mutants to maintain selection. Standard growth conditions were 16 °C with a 16 h photoperiod at an irradiance of 70 µmol photons m^−2^ s^−1^.

### Screening conditions

Pre-cultures of *S*. *marinoi* mutants and wild type were grown in EGM for five days, in triplicates, under standard growth conditions, with 0.2 µg mL^−1^ zeocin added to the mutant cultures (Supplementary Fig. 1). Cell density of pre-cultures was determined by chlorophyll fluorescence^14^ and then used to transfer an equal number of cells for each replicate into fresh EGM without zeocin in a 48-well microwell plate (Corning, USA). Separate plates were prepared and placed at each respective temperature (8, 16 and 24 °C), with multiple wild type replicates on all plates for each temperature treatment. Growth as measured by chlorophyll fluorescence was monitored daily for six days at each respective temperature.

### DNA extractions

DNA extractions were performed on *S*. *marinoi* cultures (150 mL) grown under standard conditions for 10–14 d as previously described^14^. In short, pelleted cells were ground in liquid N_2_ and the powder transferred to pre-chilled microcentrifuge tubes. Plant DNAzol (ThermoScientific, USA) supplemented with RNaseA (100 µg mL^−1^) was then added and DNA extracted according to the manufacturer’s instructions.

### Different PCR methods

Unless stated otherwise, standard PCR amplification of DNA was performed on a S1000 Thermal cycler (Bio-Rad, USA) in 25 µL reactions using 0.75 U SuperFi polymerase (ThermoScientific, USA). Primers (Supplementary Fig. 2) were used at a concentration of 0.5 µM, with 100 ng genomic DNA as template. Annealing temperature was 65°C with 35 cycles performed. For genomic mapping of each transformant, thermal asymmetric interlaced-PCR (TAIL-PCR) was performed according to Johansson *et al*.^14^ with modifications to the cycling program as shown in Supplementary Fig. 3. Bands from the tertiary reaction were purified using a Wizard DNA purification kit (Promega, USA), cloned into the Zero blunt-TOPO plasmid (ThermoScientific, USA) and transformed into *E*. *coli* DH5α. Plasmid extractions were done using the GeneJet plasmid purification kit according to the manufacturer’s instructions (ThermoScientific, USA). Sequencing was performed by Eurofins Genomics (Ebergsberg, Germany) using vector-specific primers.

### RNA extraction and RT-PCR

Total RNA was extracted from cultures grown for 5 d under standard conditions in EGM supplied with 0.2 µg mL^−1^ zeocin, using the Trizol method (ThermoScientific, USA), with polyA-RNA then extracted using the PureLink kit (ThermoScientific, USA). Contaminating DNA was removed at this step using an on-column DNaseI treatment according to the manufacturer’s instructions. Control for genomic DNA contamination was performed by running standard PCR on the purified RNA using the BLEO1 and BLEO2 primers specific to the antibiotic resistance gene^14^. RT-PCR was performed by first converting the purified RNA (250 ng) to cDNA using SuperScript IV (ThermoScientific, USA) with a mix of polyT and random hexameric oligonucleotides. The cDNAs corresponding to the *fat*1 and *lsu4e* (control) transcripts were then PCR amplified (5 ng cDNA, 28–35 cycles, 60 °C annealing temperature, 1 min extension) using the q_fat1_Fwd/Rev and q_Lsu4e_Fwd/Rev primers (Supplementary Fig. 2), respectively. RT-PCR products were separated by agarose gel electrophoresis and visualized by GelStar staining (Lonza, Switzerland). Quantification of original TIFF images was performed in Adobe Photoshop CC 2017 by integrating the band intensity histogram and subtracting the gel background. Each replicate was normalized against their respective integrated band intensity of the *lsu4e* reaction. Significance (p-value) level was determined using a standard two-tailed *t*-test in GraphPad Prism 8.0 (GraphPad Inc, USA).

### Fatty acid analysis

Pre-cultures of *S*. *marinoi* were grown in four or six replicates for 7 d in EGM under standard conditions, and then diluted into fresh EGM to grow under standard conditions. 10 mL of each culture were transferred to screw-cap glass tubes and pelleted, and the medium removed. Internal standard (di- nonadecanoyl phosphatidylcholine, 5 µg) was added to each tube. A total lipid extract was obtained as described (https://www.ncbi.nlm.nih.gov/pubmed/17951463) with the exception that K_2_SO_4_ was substituted for 5% acetic acid in the extraction. The lipid extract was transmethylated using 0.5 M sodium methoxide and the FA methyl esters extracted into heptane. These were then analyzed by GC-MS using an Agilent 6820 GC coupled to an Agilent 5975 mass selective detector. The FA methyl esters were separated on a 30 m, 0.25 mm DB-23 column (Agilent) using helium as the carrier gas at a constant flow of 0.62 mL/min. GC oven was set to 1 min at 135 °C, followed by a linear increase of 2 °C/min to 150 °C (2 °C/min), 1 °C/min to 175 °C, 2 °C/min to 210 °C, and finally 70 °C/min to 250 °C that was maintained for 2 min. The FA methyl esters were identified from mass spectra and compared to authentic standards. The FA analysis was performed three times with similar results and all 14 biological replicates from all three experiments were used to calculate averages and standard error. Statistical significance was calculated using two-tailed unpaired *t*-tests with Sidak-Bonferroni correction for multiple comparisons.

### Phylogenetic analysis

The three regions FAB1, FAB2 and ABC transporter where identified by the Conserved Domains search tool^15^ implemented in the blastp function of the NCBI web server (https://blast.ncbi.nlm.nih.gov/). Whole genome sequences from the species presented in Supplemental Tables 1 and 2 where downloaded from Phytozome v.13 (https://phytozome.jgi.doe.gov) and the Joint Genome Initiative (https://jgi.doe.gov/), and queried using the three domain sequences and blastp v2.2.29^16^. Each genome sequence was analysed independently and the ten first significant matches (as implemented in blastp^17^) where saved and aligned to the respective query sequences using mafft-linsi v7.294b^18^. Sequences that did not appear to be homologous where removed from the analysis and the alignments were then trimmed to the length of the respective query sequences. The resulting matches from the two FAB regions where combined and analysed together and the ABC sequences where analysed independently using MrBayes v3.2.6^19^ and the mixed protein model for two million generations. Trees where sampled every 1000 generations, after which the first 500 were discarded as burn-in, and the remaining 1500 trees where summarised in a 50% consensus tree.

## Results

### Screening of *S*. *marinoi* transformants for low and high temperature acclimation

**Figure 1:**
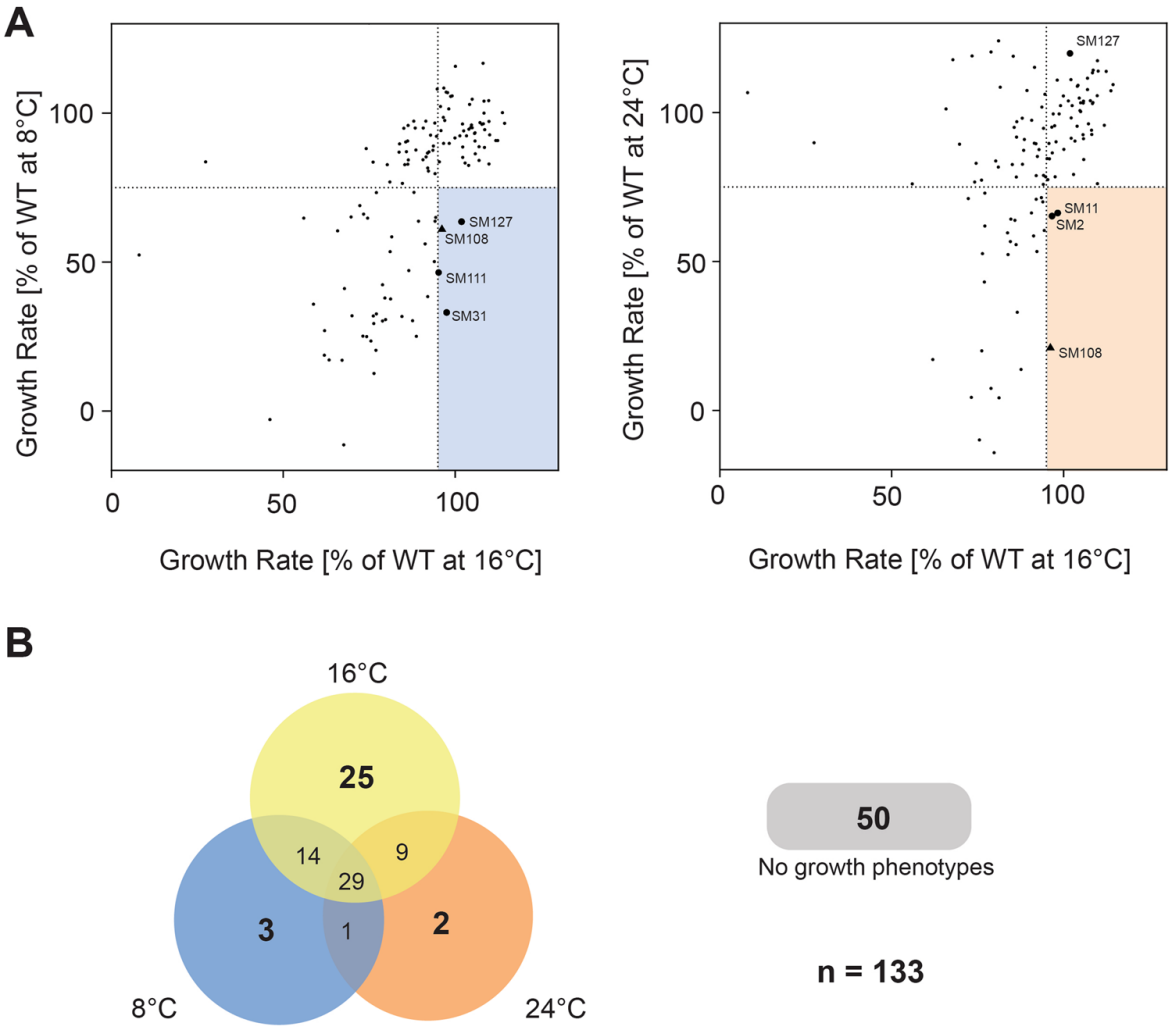
Screening for low and high temperature acclimation. Results of temperature screen. Shown in (**A**) are average growth rates (*n* = 3) for the 133 *S*. *marinoi* mutants included in the screen, displayed as percent of wild type growth rate at the respective temperature. Dashed lines indicate the 75% cutoff value considered a true phenotype at each respective temperature. Colored rectangles (8 °C, blue and 24 °C, red) mark the harsher selection criteria of 95% of wild type at 16 °C, applied to reduce the number of mutants for mapping. The dots representing the mutants passing the harsher selection criteria have been enlarged and labelled with their SMMC classification, with a black triangle representing the mutant exhibiting a compromised phenotype at both temperatures. In (**B**) a Venn diagram is shown of the distribution of phenotypes at the <75% level at 8 or 24 °C but with a 95% level for 16 °C.

In order to evaluate the feasibility of a phenomics screening approach of the *S*. *marinoi* mutant collection (SMMC) a screen was performed on 133 transformants, a number that was chosen based on those available at the time. Acclimation to different naturally-occurring temperature extremes was selected for phenotyping as this is known to affect the expression of many genes in various photosynthetic organisms^20^ and thereby increase the likelihood of identifying affected mutants within a limited collection size. Screening was performed by shifting the SMMC along with wild type controls from the standard temperature of 16 °C to either low (8 °C) or high temperatures (24 °C), two temperature extremes ecologically relevant for this diatom species^21^. A third set of the SMMC and wild type replicates were also maintained at 16 °C throughout the screening period as a control. The growth rate for each individual mutant was measured after six days and expressed as percent of wild type growth (Fig. 1A). The criteria for being assigned a temperature phenotype was set at <75% of wild type growth rate at the high or low temperatures to account for any stochastic variation of the mutant’s growth. The distribution of the mutant phenotypes at the various temperatures is shown in Fig. 1B. Two conditions were then applied for those mutants chosen for mapping to ensure a specific low and/or low temperature phenotype. Mutants should firstly display a phenotype at either the high or low temperature, or both, and then secondly, they should not have a phenotype or be subjected to stochastic variation at the standard 16 °C (>95% of wild type growth rate as indicated by a vertical line in Fig. 1A). Six mutants in total fulfilled these criteria, three only at the lower temperature (SM31, −111 and −127), two only at the higher temperature (SM2 and −8), and one with a phenotype at both temperatures (SM108). It should be noted that the SM127 not only exhibited compromised acclimation to low temperatures but also acclimated better to high temperatures relative to the wild type (Fig. 1A).

### Identification of the genomic insertion sites

**Figure 2:**
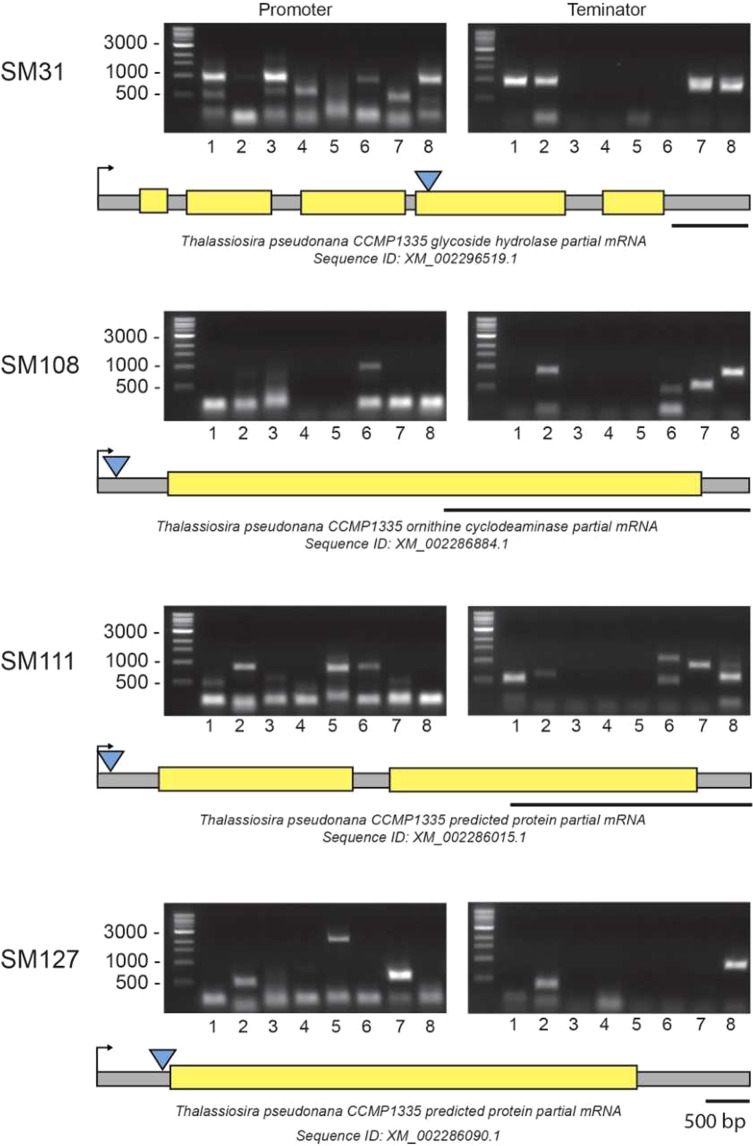
Mapping the genomic insertion sites in those transformants less able to acclimate to low or high temperatures. TAIL-PCR was performed on genomic DNA extracted from the transformants SM31, −108, −111 and −127 using the degenerate primers in each reaction in combination with the construct-specific primers for either the promoter or terminator ends. The number under each lane indicates the degenerate primer used in that reaction (Fusion 1–8, Supplementary Fig. 2). The final PCR products were separated and visualized by gel electrophoresis as shown, with selected individual products then purified and sequenced. Shown under each gel picture is the predicted gene model as identified by TAIL-PCR, with the yellow regions representing exons and the gray regions representing the non-coding sequences including introns and the 5′- and 3′ untranslated regions. The transcriptional start site for each gene is shown by the right-pointing arrow, with the genomic insertion site as mapped being indicated by the blue triangle. Underneath each gene map is shown the closest match in GenBank to the predicted *S*. *marinoi* protein along with the corresponding sequence identification number.

For each of the mutants identified from the temperature screens, we used a recently developed variation of the TAIL-PCR method^14^ to identify the genomic insertion site. This TAIL-PCR approach uses a combination of nested primers specific to either end of the construct to map the insertion site at both ends, with a set of short arbitrary degenerate primers that anneal randomly within the flanking regions. The resulting PCR products of sufficient length (i.e., ≥500 bp) are then sequenced and the flanking genomic regions identified using the newly available reference genome and transcriptomes (Töpel *et al*. in preparation). Sequencing of all PCR products revealed a single insertion site for each of the six mutants. Of the six mutants identified, the two that were compromised at high temperature had already been mapped in an earlier study, one encoding a hypothetical protein (SM11) and the other a putative G protein-coupled GABA receptor (SM2)^14^. In comparison, the three mutants mapped in this study that were compromised in low temperature acclimation were traced to genes encoding a hypothetical protein (SM111) and two putative enzymes, a glycoside hydrolase in the case of SM31 and an ornithine cyclodeaminase in SM108 (Fig. 2). The remaining mutant (SM127) exhibiting improved and impaired high and low temperature acclimation, respectively, was mapped to a gene encoding another hypothetical protein. In most cases, the closest orthologs to all the predicted proteins were found in another centric diatom species *T*. *pseudonana* with varying degrees of similarity, except for SM2 that best matched an ortholog in the pennate species *Phaeodactylum tricornutum*^14^.

### A new putative chloroplast fatty acid transporter in diatoms

**Figure 3:**
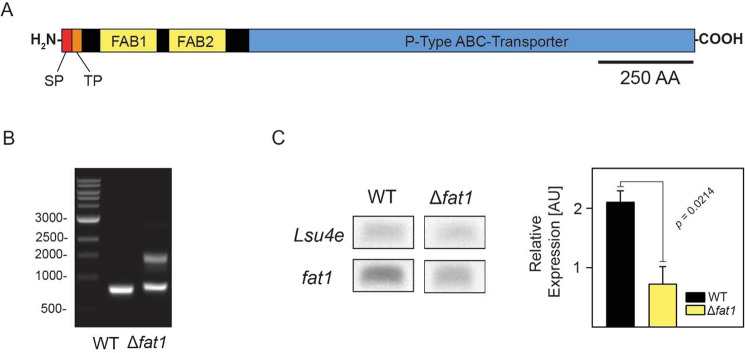
Identification of a novel chloroplast fatty acid transporter. (**A**) Schematic representation of the FAT1 polypeptide, with the position of the predicted signal peptide (SP) and chloroplast transit peptide (TP) shown in red and orange, respectively, followed by the two FA-binding domains (FAB1–2) shaded in yellow and the P-type ATPase region in blue. (**B**) Confirmation of the heterozygosity of the Δ*fat*1 knock-down mutant. Primers specific for the regions flanking ca. 250 bp either side of the inserted construct were used to determine the segregation state of the mutation. PCR was performed on genomic DNA extracted from wild type *S*. *marinoi* and the Δ*fat*1 mutant. The amplified products were separated and visualized by gel electrophoresis. The identity of all PCR products (i.e., 480 bp wild type allele, 1010 bp mutant allele) was confirmed by DNA sequencing. (**C**) Confirmation of the Δ*fat*1 knock-down mutation. Expression levels of the FAT1 transcript in wild type *S*. *marinoi* (WT) and SM127 mutant relative to that of the control Lsu4e were determined by RT-PCR (*n* = 3, SEM). Reactions were performed with equal amounts of total RNA using the gene-specific primers (i.e., q_fat1_Fwd and q_fat1_rev, q_Lsu4e_Fwd and q_Lsu4e_Rev). The resulting RT-PCR products were separated by agarose gel electrophoresis and visualized by staining with GelStar, with a representative replicate shown on the left.

Of particular interest among the mutants identified from the phenotyping screens was SM127, which in addition to being less able to acclimate to low temperature also exhibited improved high temperature acclimation compared to the wild type. The insertional disruption in this mutant was mapped to 14 bp upstream of a long, uninterrupted 4,926 bp ORF encoding a 176 kDa polypeptide (Fig. 2). Analysis of the amino acid sequence revealed several functional domains (Fig. 3A, Supplementary Figs 5–8). Within the first 40 amino acids are high-probability signal and transit peptides consistent with nuclear-encoded proteins transported into diatom chloroplasts^22^. This is then followed by two similar domains, each ca. 200 amino acids long and separated by 50 amino acids, which include the substrate-binding sites from the family of FA-binding proteins present in different photosynthetic organisms^23^. The remaining downstream sequence consists of an 1100 amino acid region with high sequence similarity to P-type ATPases, which function as transmembrane, active transporters for various ions and phospholipids in bacteria and in different membranes within eukaryotes^24^. Altogether, the above characteristics suggest the gene encodes a chloroplast-localized, FA transporter, and as such will now be referred to by the abbreviation FAT1. Searching all available genomic sequences revealed only one match to the full-length FAT1 protein in the centric diatom species, *Thalassiosira oceanica*, with another match in *T*. *pseudonana* to a region corresponding to a predicted polypeptide with only one of the two FA-binding domains in the N-terminal region (Supplementary Fig. 4).

**Figure 4:**
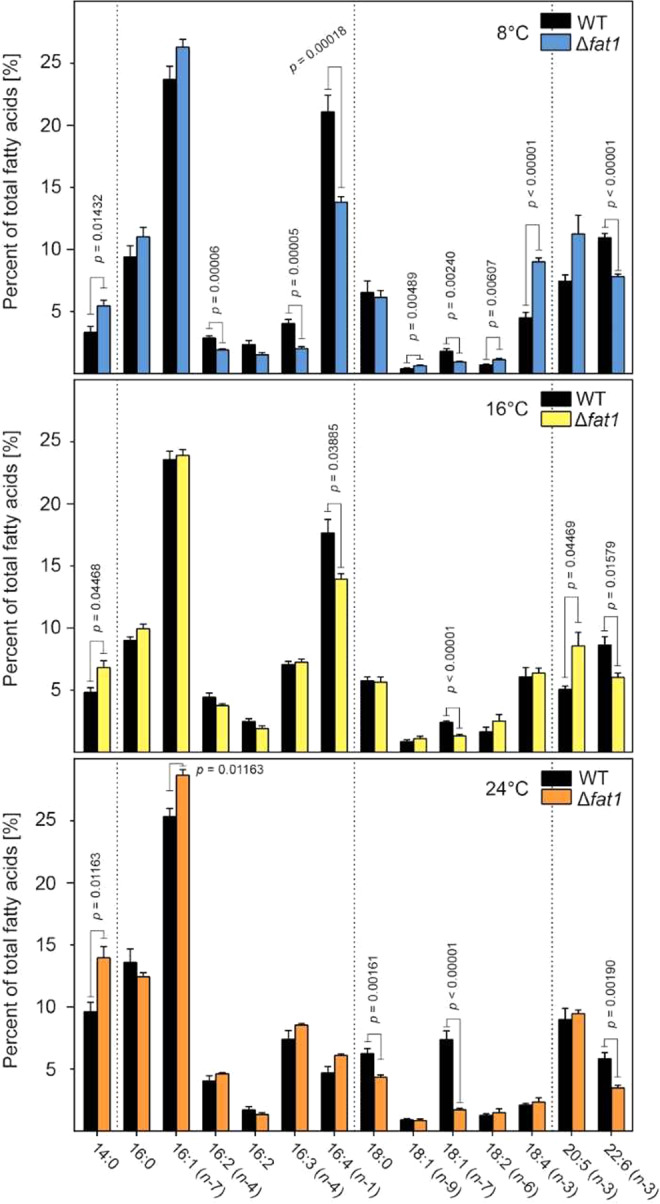
Changes to the fatty acid composition in the FAT1 knock-down mutant (SM127/Δ*fat*1) and wild type *S*. *marinoi* when grown at different temperatures. Changes in FA composition (total number of carbons: number of double bonds) in *S*. *marinoi* WT and Δ*fat*1 mutant were determined from cultures grown for 6 d under standard conditions at either 8, 16 or 24 °C. Pooled FA data from three separate experiments, totaling 14 biological replicates, were averaged and plotted as a percentage of total fatty acids (*n* = 14, SEM). Significance levels were calculated using t-tests for each FA species, with the *p*-values being corrected for comparisons using the Sidak-Bonferroni method.

We next examined if the mutation in the FAT1 gene had segregated to homogeneity given that the initial insertion of the construct would have occurred in a single allele. PCR amplification on DNA extracted from the mutant and using oligonucleotides that anneal ca. 250 bp on either side of the insertion site clearly revealed the mutant to be heterozygous, with PCR products for both the wild type and mutant alleles (Fig. 3B). Expression analysis of the FAT1 gene by RT-PCR also revealed a more than 50% loss in FAT1 transcript levels in the SM127 mutant relative to the wild type, confirming the knock-down mutation (Fig. 3C). Given the FA-binding domains in the FAT1 protein, we then compared the cellular FA composition between wild type and SM127 at the control temperature of 16 °C and found quantitative changes to five specific FAs; myristic acid (14:0), hexadecatetraenoic acid (16:4), 18:1 (*n*-7) and the omega-3, long-chain polyunsaturated FAs (LC-PUFA) eicosapentaenoic (EPA, 20:5 *n*-3) and docosahexaenoic acid (DHA, 22:6 *n*-3) (Fig. 4). Changes to the level of the latter two FAs were of particular note given that EPA is the precursor to DHA, with the relative accumulation of EPA and corresponding loss of DHA in SM127 suggesting this conversion is directly compromised by the loss of FAT1 protein.

Given the high and low temperature phenotypes of the SM127 mutant, we then compared its cellular FA composition with the wild type at both temperatures. At 8 °C, the wild type increased the proportion of certain PUFAs (16:4, 20:5 and 22:6) while decreasing that of the more saturated FAs (SFA; 14:0, 16:2, 16:3, 18:1 and 18:2), thus leading to an overall increase in desaturation at low temperature. In comparison, the SM127 mutant maintained the quantitative changes to the five specific FAs as observed at 16 °C, although the differences became more pronounced in the case of the PUFAs. Additional differences in the composition of the less abundant FAs also occurred between the wild type and SM127 at the low temperature, with the most significant being drops in 16:2 *n*-4, 16:3 and 18:1 *n*-7 combined with a doubling of 18:4 (Fig. 4). At 24 °C, the wild type accumulated more of the SFAs (14:0, 16:0, 16:1, and 18:1 *n*-7) compared to the control temperature and less of most PUFAs (16:4, 18:4 and 22:6). The most significant changes in the SM127 mutant at 24 °C were the dramatic reduction in 18:1 *n*-7 along with that of 18:0 and the continued inability to produce 22:6. In contrast, the mutant also had a higher proportion of the abundant short-chain SFAs 14:0 and 16:1.

## Discussion

Despite diatoms being the most ecologically successful eukaryotic marine phytoplankton contributing to global photosynthetic carbon fixation and biogeochemical cycling of different nutrients and minerals, much remains unknown in this group about the specific gene functions that underlie these important characteristics. In this age of post-genomics, the evolutionary genetic complexity of diatoms as revealed by comparative genomics remains a significant barrier as does the large proportion of genes in diatoms genomes encoding hypothetical protein (e.g., 79% for *S*. *marinoi*). Despite mutagenesis being one of the most informative methods for determining specific gene functions, such studies for diatoms have so far been limited to single genes within only a few species. To this end, we recently developed a genetic transformation procedure for *S*. *marinoi* based on nonhomologous integration of a linear DNA construct randomly within the nuclear genome, together with a PCR-based approach to reliably map the genomic insertion site of this construct in different transformants^14^. We have now undertaken a study as proof-of-concept to assess the suitability of *S*. *marinoi* for phenomics-based screening. Temperature acclimation was chosen for the phenotyping conditions, and although only a limited number of transformants were included, several mutants with clear low or high temperature phenotypes were identified.

Of the genes identified from the temperature acclimation screening, certain examples encoding proteins with predicted functions were consistent with the temperature phenotypes. For those linked to low temperature acclimation, the enzyme ornithine cyclodeaminase catalyzes the single‐step formation of proline from ornithine that derives from the urea cycle in diatoms^25^. The synthesis and accumulation of proline is well known to occur during cold acclimation to low, non-freezing temperatures and improve freezing tolerance in plants^26^. Indeed, proline plays several roles during plant stress tolerance including as a mediator of osmotic adjustment and a stabilizer of proteins and membranes^27^. Although its importance for cold acclimation in diatoms remains unclear, the fact that proline is the principal osmolyte^28^ suggests that any compromise in its synthesis could well affect the ability of an ornithine cyclodeaminase mutant to acclimate to low temperatures. The other gene linked to low temperature acclimation encodes a glycoside hydrolase, one of a large (>100 subfamilies) and diverse family of enzymes found in most domains of life. In general, glycoside hydrolases hydrolyze the glycosidic bond between two or more carbohydrates, or between a carbohydrate and a non-carbohydrate group^29^. In plants, they are located in the endoplasmic reticulum and Golgi apparatus and are involved in the processing of N-linked glycoproteins^30^. Despite the commonality of glycoside hydrolases in nature, however, little is yet known about their specific role in diatoms and other microalgae, nor their possible involvement in stress acclimation.

The one identified gene with known function from the high temperature phenotyping encoded a putative G protein-coupled GABA receptor. In general, G protein-coupled receptors are a highly conserved superfamily of cell-surface receptors that play a crucial role in the signal transduction pathways that perceive and respond to different environmental cues^31^. Although well characterized in plants and other eukaryotes, their function in diatoms remains unclear despite the fact that whole genome sequencing has revealed them in several different species^32^. Equally unknown in diatoms is the role of the subfamily of G protein-coupled receptors specific for the signaling molecule γ-aminobutyric acid (GABA). However, GABA concentrations in plants increase several-fold under various stress conditions including high temperatures^33^, and it has been proposed that G protein-coupled GABA receptors could help mediate the sensing and eventual acclimation of diatoms to such stresses^32^. As such, a possible role for the G protein-coupled GABA receptor identified in this study in the acclimation of *S*. *marinoi* to high temperatures is plausible given the limited information to date. Further investigations of this type of receptor including a more detailed characterization of the SM2 mutant could help resolve the regulatory mechanisms that instigate high temperature acclimation in diatoms.

In addition to the three mutants affected in genes encoding proteins with predicted functions, three others were identified from the temperature phenotyping encoding hypothetical proteins. A more detailed analysis of these sequences revealed one with several conserved functional domains. A large proportion of the protein has high sequence similarity and is structurally related to P-type ATPases, which in eukaryotes typically function as ABC transporters that pump ions and lipids across membranes^24^. The protein, which we now call FAT1, is unique among other known P-type ATPases in that it has two FA-binding domains positioned upstream^23^, although it was unclear from sequence conservation alone which specific types of FAs are transported.

Most of our knowledge about FA and lipid biosynthesis in diatoms and how these are regulated remains very limited, with many of the current models derived from those in green algae and plants and based upon genome predictions. In regards to FA biosynthesis, much of the experimental support for the diatom model comes from the pennate *P*. *tricornutum*^34^, although how applicable this is in bipolar centric species such as *S*. *marinoi* remains far from certain. Similar to that in plants, FA biosynthesis in diatoms is thought to occur mostly in plastids, beginning with the primary substrate acetyl-CoA that is eventually converted to malonyl-ACP. Malonyl-ACP is then converted by the enzymes of the type II FA synthase complex to 16:0-ACP. Unlike in plant plastids, there is so far no evidence for 18:0-ACP synthesis in diatom plastids, with 16:0-ACP being either incorporated into plastid lipids or exported as the 16:0 FA by an as yet unknown mechanism to support the synthesis of longer-chain FAs in the ER via a series of desaturation and elongation reactions. In the cytosol, excess 16C-FA can also be incorporated into triacylglycerols (TAGs) that act as a carbon store. The most common long-chain FAs in *P*. *tricornutum* that are incorporated into various glycerolipids are 20:5 (EPA), 22:6 (DHA) and 24:0^35^, although this differs significantly in different diatom species^36^. Of the LC-PUFAs, a proportion of EPA is thought to be imported from the ER into plastids to support galactolipid synthesis, although the mechanism for this transport remains unknown and is presumably complicated further by the unusual membrane composition of diatom plastids and their close association to the ER. A more detailed description of the current models for FA and lipid biosynthesis in diatom can be found here^34,37^.

Of the FAs that quantitatively changed in the mutant, only one (EPA) is known to be transported between the two main sites of FA synthesis in photosynthetic organisms, namely the chloroplast and ER. EPA is an omega-3 PUFA that is typically synthesized in the ER membrane of lower plants and microalgae, as well as in *P*. *tricornutum*^37^, with some of the EPA then imported into plastids for galactolipid biosynthesis. However, it has been proposed in the oleaginous diatom *Fistulifera sp*. that the final step of EPA synthesis might instead occur inside plastids^38^, opening up the possibility of EPA export from plastids, although as yet there is no experimental evidence to support such a mechanism. As an omega-3 LC-PUFA, EPA is important for human nutrition and has numerous health benefits including reduced risk of cardiovascular disease, prevention of several cognitive disorders and protection from various cancers^39,40^. Historically, the main source of these LC-PUFA has been fish but changes to modern dietary habits have seen a drop in their intake in most industrial countries. This has placed increasing pressure on aquaculture to compensate for shrinking wild fish populations and the need to provide fish feeds with relatively high concentrations of EPA and other omega-3 LC-PUFAs^41,42^. As a consequence, diatoms have attracted much interest from industry on account of their unusually high proportion of LC-PUFA, in particular EPA and DHA. Based on bioinformatic analyses, the genes encoding the necessary desaturases and elongases for EPA synthesis exist within the sequenced diatom genomes^43^, although the exact subcellular site of EPA synthesis remains unclear^44^. EPA serves two main functions, as a precursor for the synthesis of another omega-3 LC-PUFA, DHA, in the ER, and being incorporated into chloroplast galactolipids. Although the current model for FA biosynthesis in diatoms states that EPA is synthesized in the ER and transported only into plastids, the relative accumulation of EPA and corresponding decrease in DHA within the SM127 mutant (now referred to as Δ*fat*1) is consistent with FAT1 having a role in *S*. *marinoi* in EPA export from plastids to the ER. If so, the accumulation of EPA within the chloroplasts of the Δ*fat*1mutant might also have led to the observed decrease of another PUFA (i.e., 16:4) given that EPA is preferentially incorporated into plastid lipids, thereby reducing the need for the shorter-chain PUFA in the mutant. Such a possibility is supported by the known incorporation of 16:4 and EPA in both MGDG and DGDG in a closely related *Skeletonema* sp. (*S*. *costatum*)^45^, and contrasting further with that in the pennate *P*. *tricornutum* in which the 16:4 FA is unique to MGDG.

The fact that this type of putative ABC transporter is unique to diatoms could well be related to the unusual membrane organization surrounding diatom plastids, which consists of four membranes originating from the engulfment of a red algal ancestor and subsequent reduction of the symbiont subcellular structures^46^. That orthologs of FAT1 appear to exist only in centric diatoms further suggests that not only might the location of EPA synthesis differ between the two groups of diatoms but that more studies on the FA and lipid biosynthetic pathways need to be done on different centric diatoms. This is particularly important given that our knowledge of the FA biosynthetic pathways in diatoms remain fragmentary and so far restricted to a limited number of mostly pennate species. Because of this uncertainty, it is also possible that the FAT1 protein is involved in the transport of other FAs between the ER and chloroplast, whose disruption in the Δ*fat*1 mutant leads to the observed changes in FA content including the accumulation of EPA and corresponding decrease in DHA. Indeed, we plan to further investigate the precise role of this unusual FA transporter in *S*. *marinoi* by comparing the FA compositions of all the different classes of lipids in both wild type *S*. *marinoi* and the Δ*fat*1 mutant, and if indeed EPA might be a possible substrate. We are also preparing a recombinant protein in which the FA-binding domains from the FAT1 protein are fused to the maltose-binding protein in order to test *in vitro* which specific FAs are specifically bound.

It is known that temperature is one of the main environmental conditions that can alter FA composition in diatoms and other microalgae^47^. In *P*. *tricornutum*, increases in PUFAs along with decreases in SFAs at low temperatures enhance membrane fluidity, which is a well-characterized cellular biophysical acclimation to cold conditions^48–52^. The increases in the PUFAs we observed in *S*. *marinoi* at low temperatures is therefore consistent with similar FA modifications in other microalgae, although interestingly there was no corresponding decrease in SFAs in *S*. *marinoi*. Indeed, reduced synthesis of the two most abundant PUFAs (16:4 and 22:6) likely contributes to the compromised ability of the Δ*fat*1 mutant to acclimate to low temperatures, with corresponding increases in other PUFAs such as 18:4 being unable to adequately compensate.

In contrast to low temperatures, acclimation to high temperatures involves opposing changes in FA composition in microalgae and vascular plants: increases in SFAs with concomitant decreases in PUFAs^20,47,53^. Such changes in FA composition were also observed for wild type *S*. *marinoi* during acclimation to 24 °C, with increases in the SFAs 14:0, 16:0 and 16:1 and corresponding drops in PUFAs such as 16:4, 18:4 and 22:6. Heat accelerates lipid movement within membranes and thereby their fluidity, leading to potential loss of metabolic activities such as photosynthesis and possible cell rupture due to ion leakages and denaturation of embedded proteins^54^. Chloroplast membranes are especially prone to this type of damage due to their high proportion of PUFAs^55^. Increases in the proportion of SFAs confer heat tolerance by avoiding the fluidization of the membranes under conditions of high temperatures and thereby increasing their thermal resistance^54^. The improved tolerance of the Δ*fat*1 mutant could therefore be related to the elevation of the more abundant SFAs such as 14:0 and 16:1, which would most affect the FA composition of heat-sensitive chloroplast membranes.

In conclusion, we believe *S*. *marinoi* will become an invaluable addition to the functional genomic and phenomic studies of marine diatoms that have so far been mostly focused on *P*. *tricornutum* and *T*. *pseudonana*. This will be of particular importance in elucidating those genes responsible for the global ecological success of marine diatoms and how this might be impacted upon by future climate change. We are now in the process of scaling up the *S*. *marinoi* transformations to produce a more extensive collection of independent lines suitable for future large-scale phenotyping. Such large-scale insertional mutagenesis programs have been especially successful in other model organisms, including *Drosophila*^56^, mouse^57^, zebrafish^58^, the green algae *C*. *reinhardtii*^13^ and the vascular plant *Arabidopsis thaliana*^12^. The immense value of such genomic scale mutagenesis approaches remains true today despite the development of new approaches for targeted mutagenesis such as CRISPR and TALENs, for which concerns persist over specificity and the extent of off-target effects^59,60^. We believe that this extensive random mutagenesis platform for *S*. *marinoi* will quickly become a key resource for elucidating gene functions in diatoms, and complement the application of other more targeted approaches to diatoms that develop in the future.

## Supplementary information

Supplementary figures

Supplementary dataset
