# Supplementary material for: Phenomics reveals a novel putative chloroplast fatty acid transporter in the marine diatom Skeletonema marinoi involved in temperature acclimation
Source: Sci Rep. 2019 Oct 22;9:15143. doi: 10.1038/s41598-019-51683-y (PMC6805942; doi:10.1038/s41598-019-51683-y)
Supplement: Supplementary file 1 — Supplementary figures [file 41598_2019_51683_MOESM1_ESM.pdf]

Phenomics reveals a novel chloroplast fatty acid transporter in the marine diatom *Skeletonema marinoi* involved in temperature acclimation

Oskar N. Johansson[1], Mats Töpel[2,3], Jenny Egardt[1], Matthew I.M. Pinder[2], Mats X. Andersson[1], Anna Godhe[2], and Adrian K. Clarke[1*]

[1]Department of Biological and Environmental Sciences, University of Gothenburg, Box 461, 40530 Gothenburg, Sweden.

[2]Department of Marine Sciences, University of Gothenburg, Box 462, 40530 Gothenburg, Sweden.

[3]Gothenburg Global Biodiversity Center (GGBC), Box 461, 40530 Gothenburg, Sweden.

[*]Materials and correspondence: Adrian K. Clarke. Department of Biological and Environmental Sciences, University of Gothenburg, Box 461, 40530 Gothenburg, Sweden (adrian.clarke@bioenv.gu.se).

Correspondence should be addressed to A.K.C. (adrian.clarke@bioenv.gu.se).

Supplementary Figure 1. Overview of the temperature screening for *S. marinoi* mutant collection.

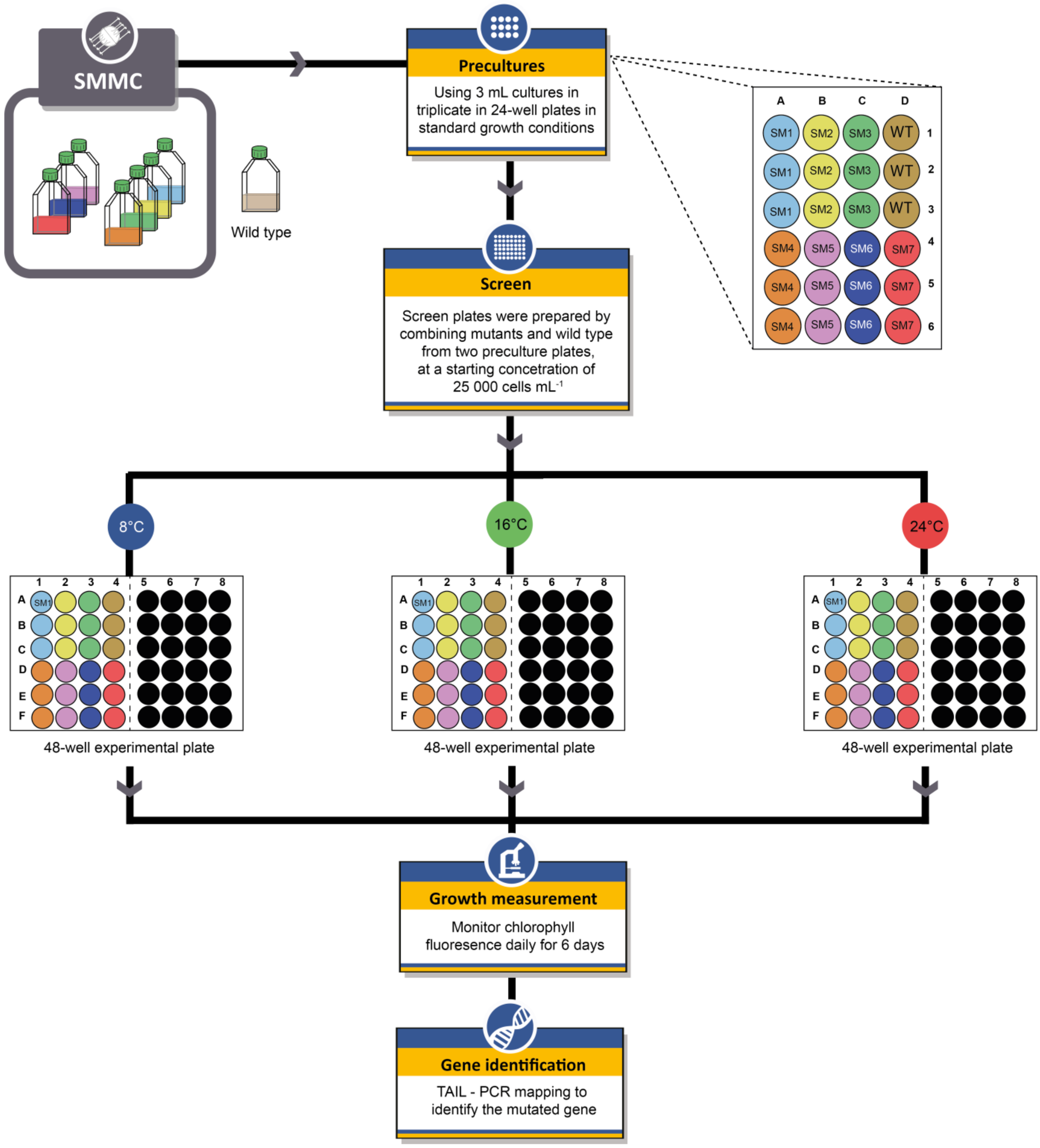

Supplementary Figure 2. List of primers

Primer	Sequence (5’->3’)
**TAIL-PCR**	
	Insert specific primers
**Promoter**	
pISP[1]	CCTGCGAAATCATCCTCCACAAAATCTCG
pISP[2]	GAGAATCCGAGACGATCTGTCCAGAACTC
pISP[3]	AGCACTGGAACAGCACTTGTCAACTTTGC
**Terminator**	
tISP[1]	CGAGATTTTGTGGAGGATGATTTCGCAGG
tISP[2]	GAATGGTCTGAAGTAGTGTCTACAAATTTCCGTG
tISP[3]	GCGTGCATTTCGTGGCAGAAGAGCAAG
	Degenerate primers
Fusion-1	GGGCACGTCTACTATAGGGCACGCGTGGT**NTCGASTWTSGWGTT**
Fusion-2	GGGCACGTCTACTATAGGGCACGCGTGGT**NGTCGASWGANAWGAA**
Fusion-3	GGGCACGTCTACTATAGGGCACGCGTGGT**WGTGNAGWANCANAGA**
Fusion-4	GGGCACGTCTACTATAGGGCACGCGTGGT**AGWGNAGWANCAWAGG**
Fusion-5	GGGCACGTCTACTATAGGGCACGCGTGGT**NGTAWAASGTNTSCAA**
Fusion-6	GGGCACGTCTACTATAGGGCACGCGTGGT**NGACGASWGANAWGAA**
Fusion-7	GGGCACGTCTACTATAGGGCACGCGTGGT**GTNCGASWCANAWGTT**
Fusion-8	GGGCACGTCTACTATAGGGCACGCGTGGT**NCAGCTWSCTNTSCTT**
	Amplicon specific primer
ASP1	GGGCACGTCTACTATAGGGC
ASP2	ACTATAGGGCACGCGTGGT
**Genotyping**	
SM127-fwd	ACTGGACAAAAATTACTGCAAAGCAAGAGACGGC
SM127-rev	ATCCTATCTCCGTTCCATCAACAGATACTTTGACTCCC
**qRT-PCR**	
q_Lsu4e_Fwd	CATGGGGAACTGGACGTGCCG
q_Lsu4e_Rev	GGTGTACTTACGGCCACGGGC
q_fat1_Fwd	AACAACTTATTCTTGATGGCGTAGCGG
q_fat1_Rev	CGC TCCTGCGATAGTTTTTGCATCC

Supplementary Figure 3. Thermal conditions for the modified TAIL-PCR approach used to map the genomic insertion sites in *S. marinoi* transformants.

Primary reaction (1°)			Secondary reaction (2°)			Tertiary reaction (3°)		
**Step**	**Temp.**	**Time**	**Step**	**Temp.**	**Time**	**Step**	**Temp.**	**Time**
1	96°C	5 min	1	98°C	20 s	1	98°C	30 s
2	98°C	10 s	2	65°C	1 min	2	98°C	10 s
3	70°C	15 s	3	72°C	2 min	3	57°C	15 s
4	72°C	2 min	4	Go to 1	1 x	4	72°C	2 min
5	Go to 2	30 x	5	94°C	20 s	5	Go to 2	14 x
6	98°C	10 s	6	68°C	1 min	6	72°C	5 min
7	25°C	1 min	7	72°C	2 min	7	4°C	∞
8	72°C<br>ramp 0.5 °C / sec	2 min	8	94°C	20 s			
9	Go to 6	1 x	9	68°C	1 min			
10	98°C	10 s	10	72°C	2 min			
11	70°C	15 s	11	94°C	20 s			
12	72°C	2 min	12	55°C	1 min			
13	98°C	10 s	13	72°C	2 min			
14	70°C	15 s	15	72°C	5 min			
15	72°C	2 min	16	4°C	∞			
16	98°C	10 s						
17	40°C	15 s						
18	72°C	2 min						
19	Go to 10	10 x						
21	72°C	5 min						
22	4°C	∞						

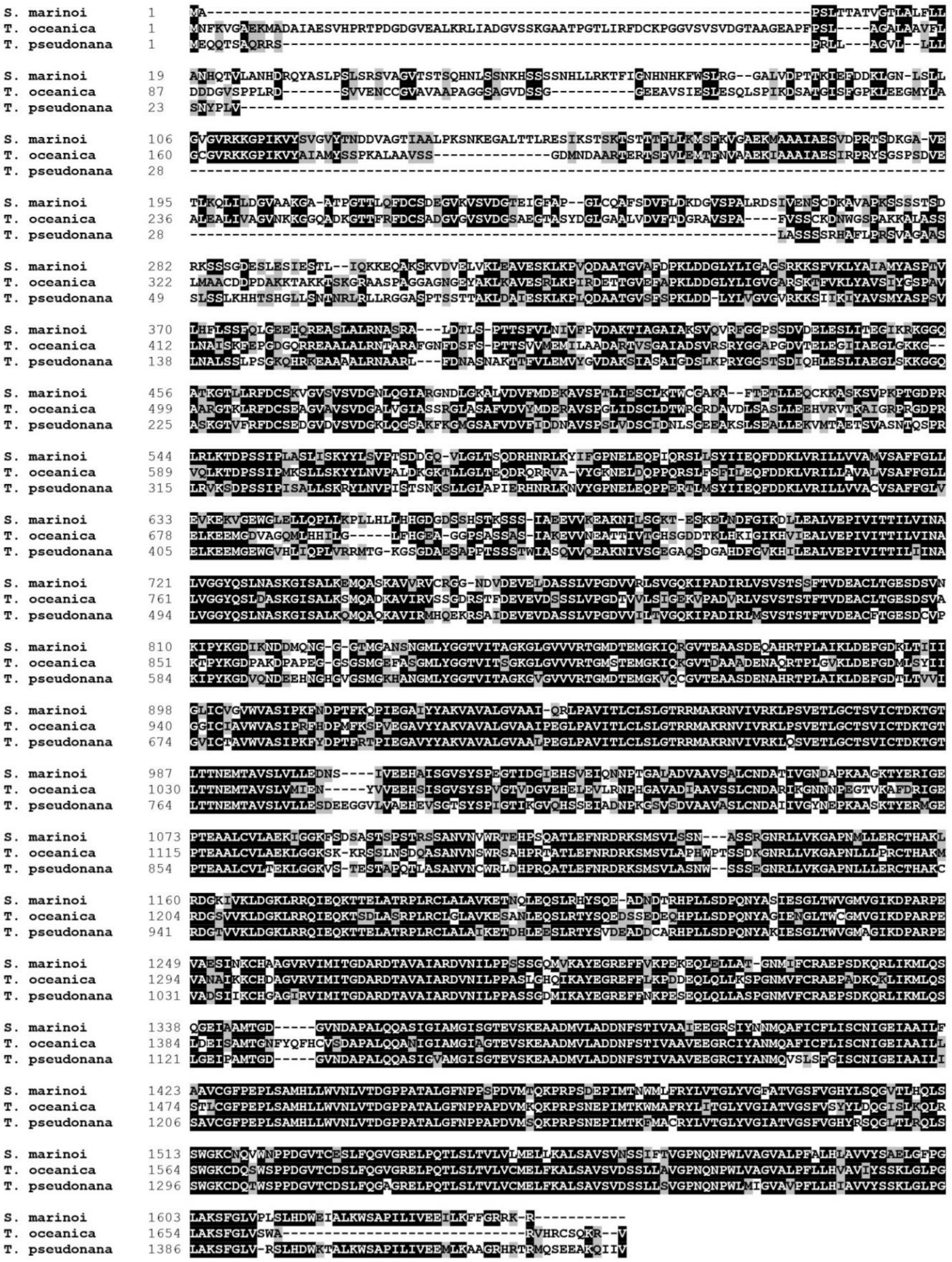

Supplementary Figure 4. Amino acid sequence alignment of the *S. marinoi* FAT1 protein with the orthologs in *T. oceanica* and *T. pseudonana*. The sequence from *T. pseudonana* consists of several ORF predictions (generated using the program getorf in the EMBOSS v6.3.1 package) grafted together after putative intron sequences where removed. Conserved sites are indicated by a black background, and similar amino acids by a grey background.

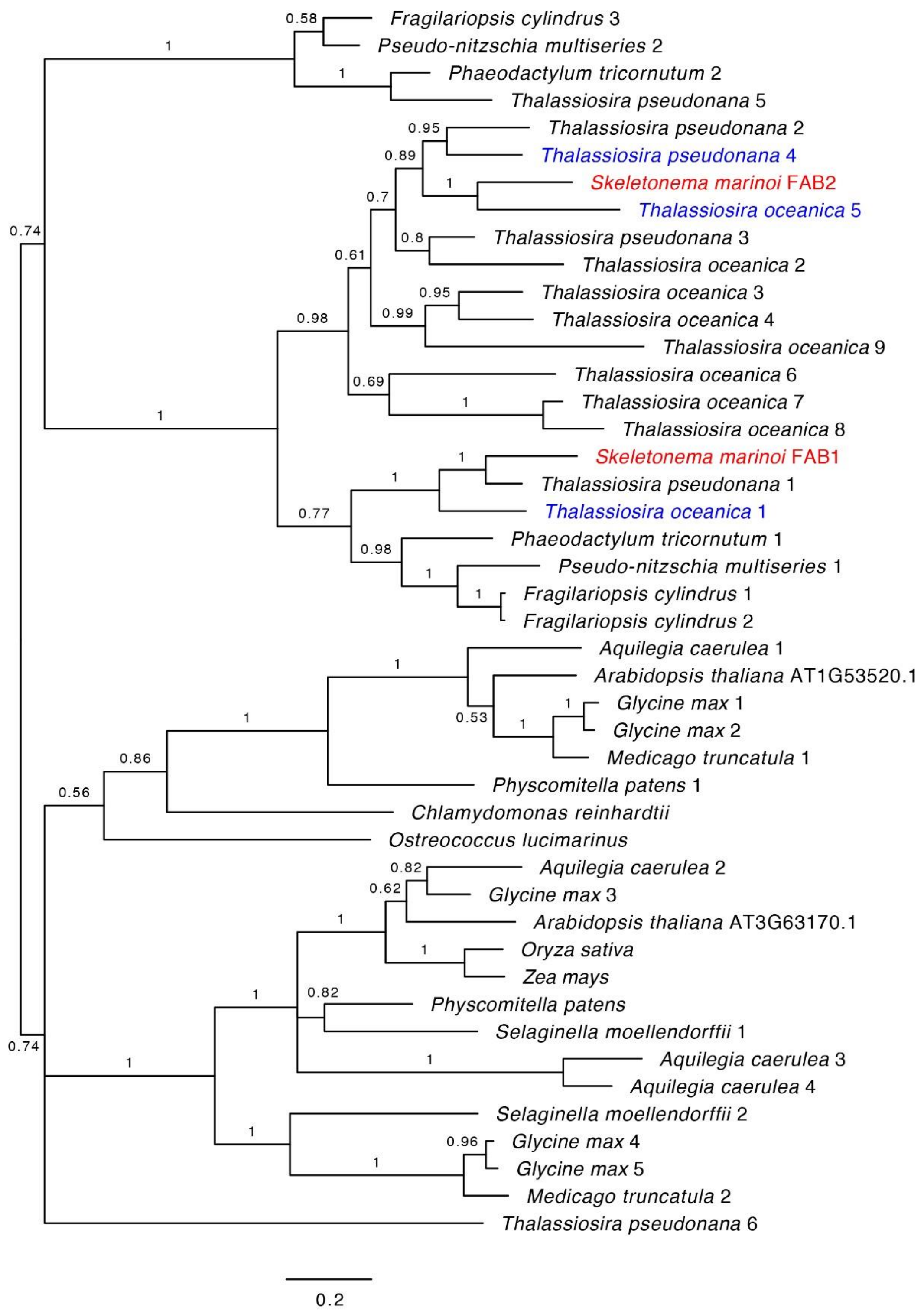

Supplementary Figure 5. Unrooted phylogenetic tree of the fatty acid-binding (FAB) domains presented in supplementary table SX. Highlighted in red are the two FAB regions found in the *S. marinoi* FAT1 protein. Highlighted in blue are the three domains from *T. oceanica* and *T. pseudonana* that are part of the proteins shown in Supplementary Figure 4. Posterior probability values appear above the branches, and the expected number of changes per site along the branches is indicated by the scale bar.

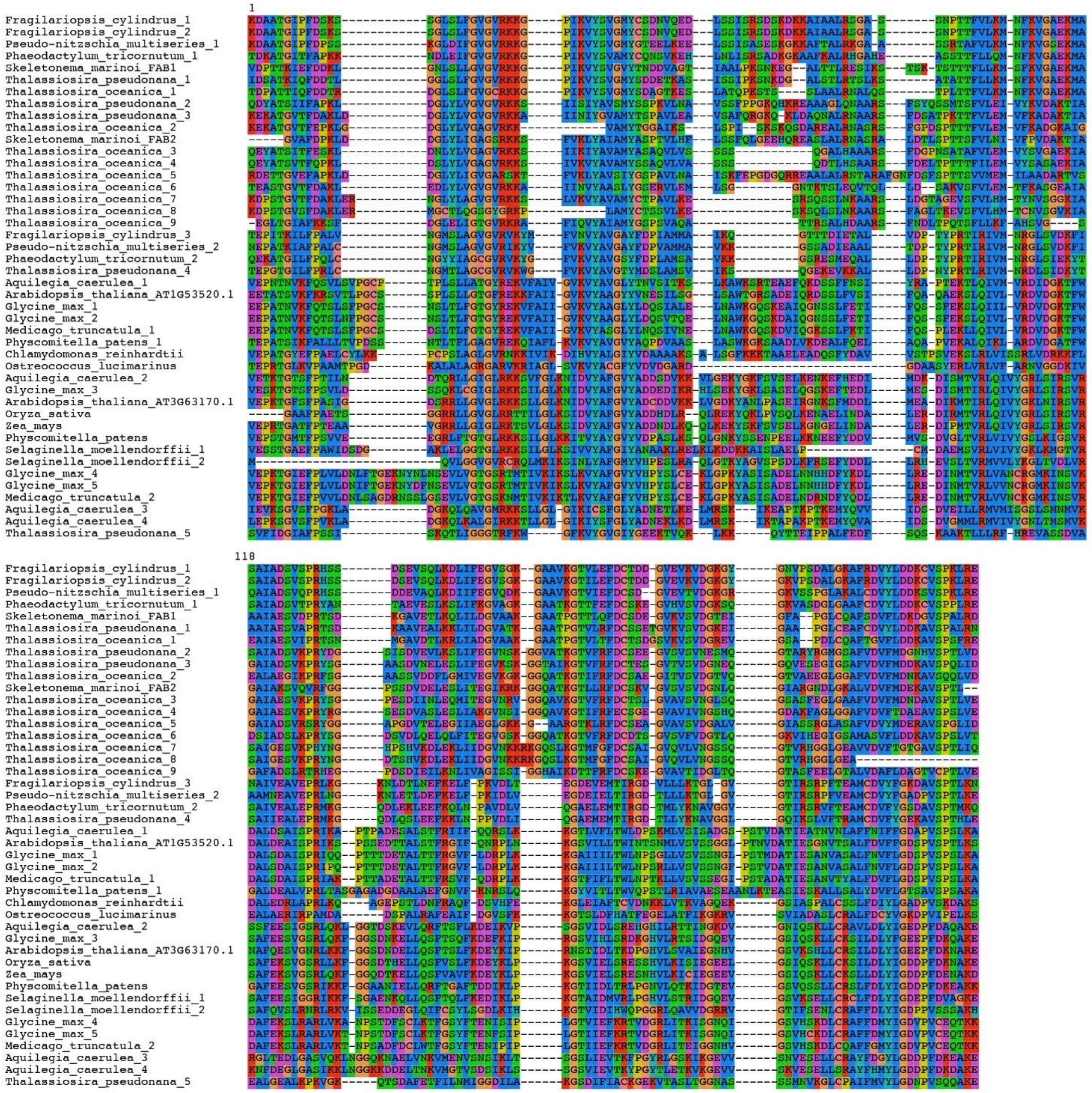

Supplementary Figure 6. Alignment of the FAB domains identified in green algae, land plants and diatoms and used for the phylogenetic analysis presented in Supplementary Figure 5. The length of the sequences has been adjusted to the length of the two domains found in *S. marinoi*. Numbers above the sequences indicate the positions in the alignment and the background color of the letters indicates size, shape, solubility, and ionization properties of the different amino acids.

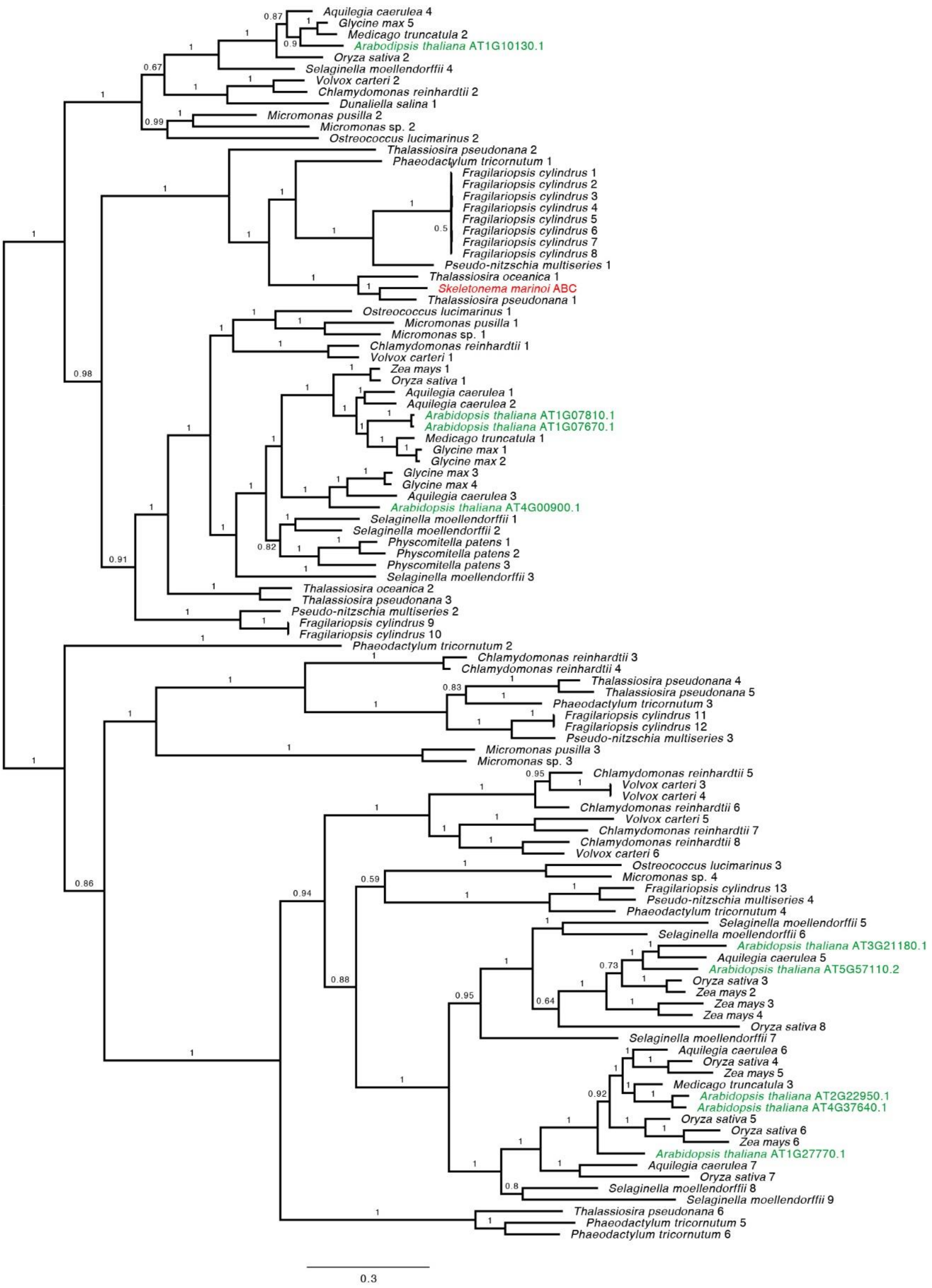

Supplementary Figure 7. Unrooted phylogenetic tree of the ABC transporter domains presented in Supplementary Table SX. The position of the *S. marinoi* sequence is highlighted in red and eight homologous domains found in *Arabidopsis thaliana* are highlighted in green. Posterior probability values appear above the branches, and the expected number of changes per site along the branches is indicated by the scale bar.

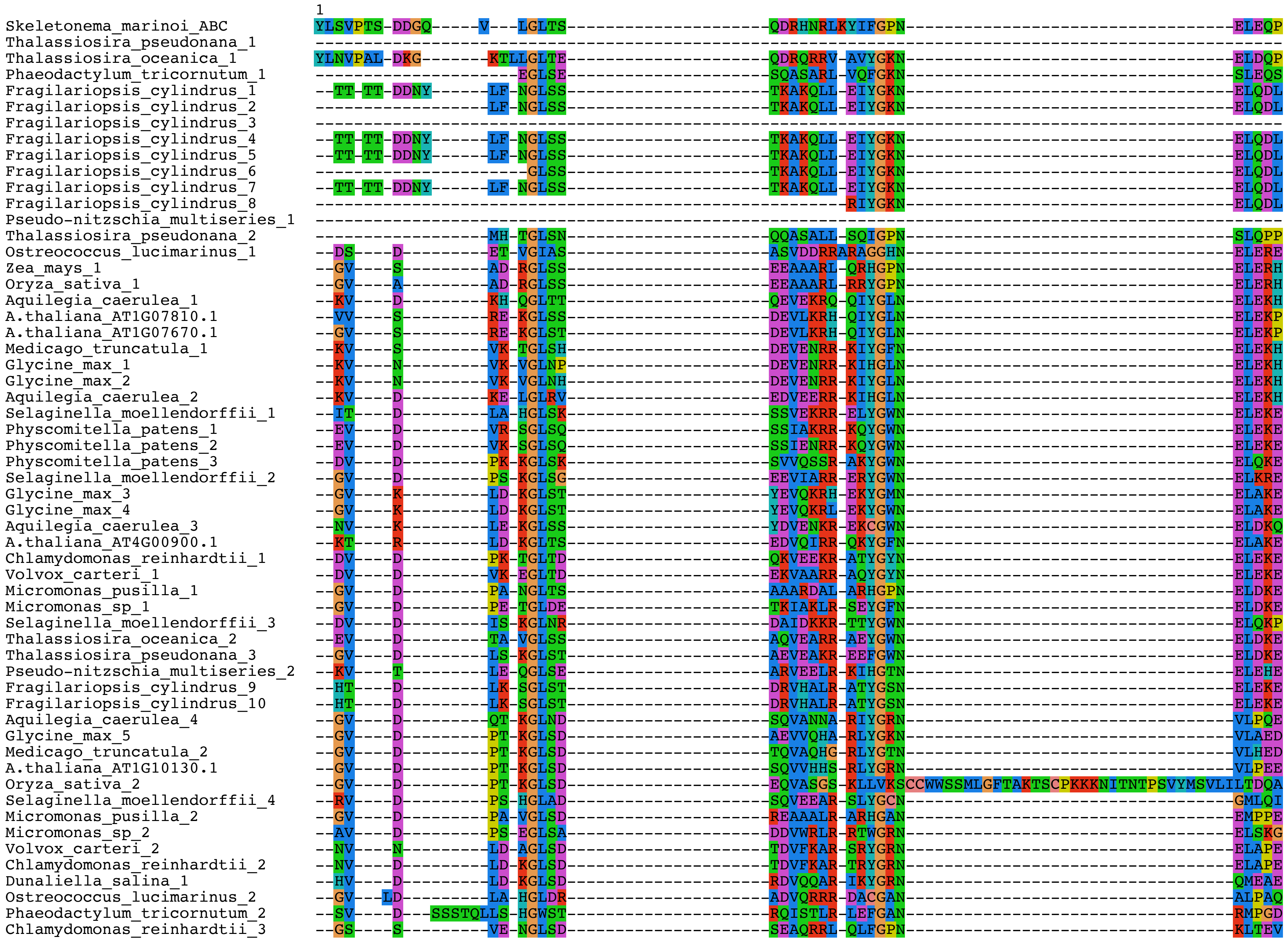
1
Skeletonema_marinoi_ABC
Thalassiosira_pseudonana_1
Thalassiosira_oceanica_1
Phaeodactylum_tricornutum_1
Fragilariopsis_cylindrus_1
Fragilariopsis_cylindrus_2
Fragilariopsis_cylindrus_3
Fragilariopsis_cylindrus_4
Fragilariopsis_cylindrus_5
Fragilariopsis_cylindrus_6
Fragilariopsis_cylindrus_7
Fragilariopsis_cylindrus_8
Pseudo-nitzschia_multiseries_1
Thalassiosira_pseudonana_2
Ostreococcus_lucimarinus_1
Zea_mays_1
Oryza_sativa_1
Aquilegia_caerulea_1
A.thaliana_AT1G07810.1
A.thaliana_AT1G07670.1
Medicago_truncatula_1
Glycine_max_1
Glycine_max_2
Aquilegia_caerulea_2
Selaginella_moellendorffii_1
Physcomitella_patens_1
Physcomitella_patens_2
Physcomitella_patens_3
Selaginella_moellendorffii_2
Glycine_max_3
Glycine_max_4
Aquilegia_caerulea_3
A.thaliana_AT4G00900.1
Chlamydomonas_reinhardtii_1
Volvox_carteri_1
Micromonas_pusilla_1
Micromonas_sp_1
Selaginella_moellendorffii_3
Thalassiosira_oceanica_2
Thalassiosira_pseudonana_3
Pseudo-nitzschia_multiseries_2
Fragilariopsis_cylindrus_9
Fragilariopsis_cylindrus_10
Aquilegia_caerulea_4
Glycine_max_5
Medicago_truncatula_2
A.thaliana_AT1G10130.1
Oryza_sativa_2
Selaginella_moellendorffii_4
Micromonas_pusilla_2
Micromonas_sp_2
Volvox_carteri_2
Chlamydomonas_reinhardtii_2
Dunaliella_salina_1
Ostreococcus_lucimarinus_2
Phaeodactylum_tricornutum_2
Chlamydomonas_reinhardtii_3

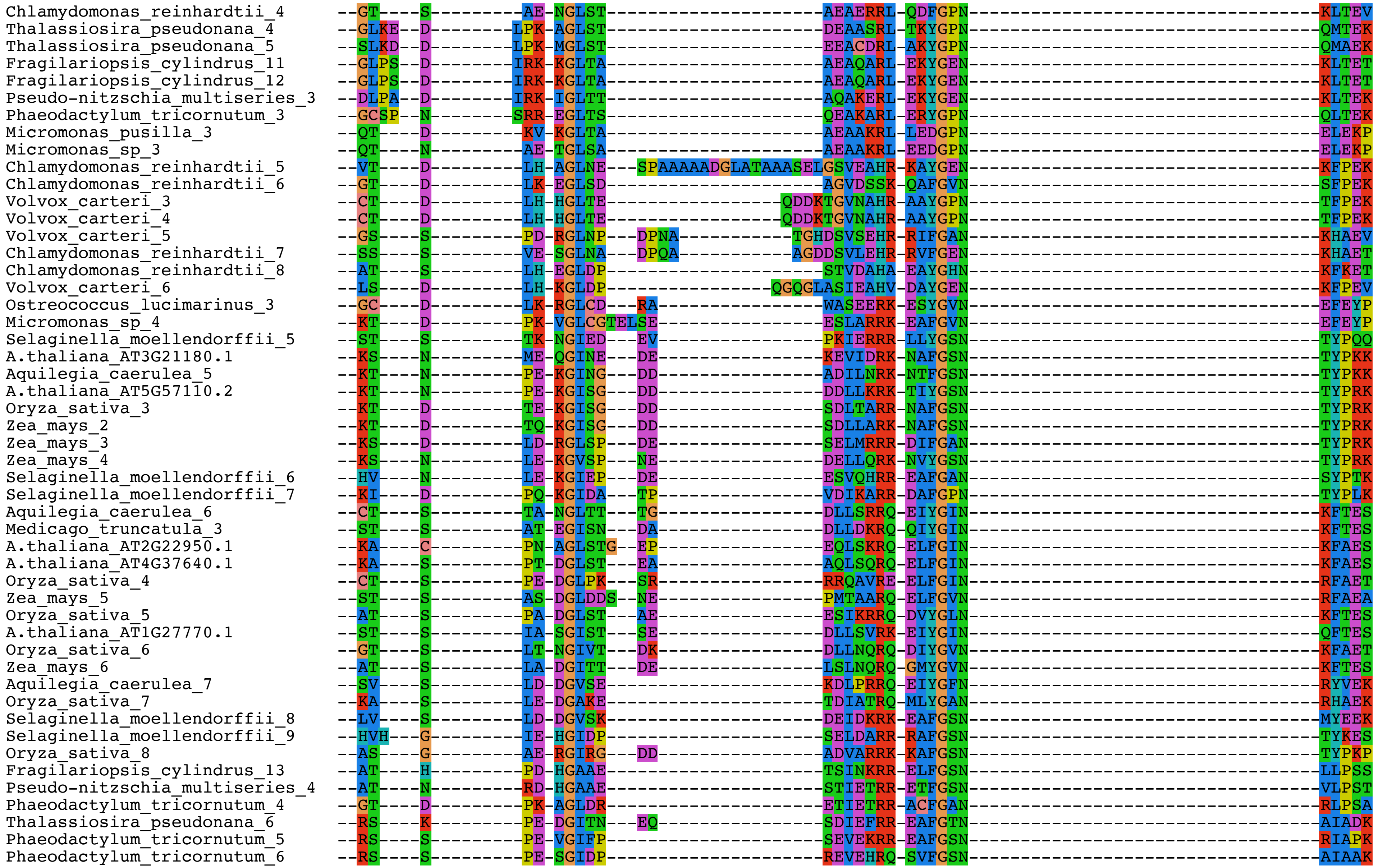
Chlamydomonas_reinhardtii_4 GT S AE NGLST AEAERRL QDFGPN KLTEV
Thalassiosira_pseudonana_4 GLKE D LPK AGLST DEAASRL TKYGPN QMTEK
Thalassiosira_pseudonana_5 SLKD D LPK MGLST EEACDRL AKYGPN QMAEK
Fragilariopsis_cylindrus_11 GLPS D IRK KGLTA AEAQARL EKYGEN KLTET
Fragilariopsis_cylindrus_12 GLPS D IRK KGLTA AEAQARL EKYGEN KLTET
Pseudo-nitzschia_multiseries_3 DLPA D IRK IGLTT AQAKERL EKYGEN KLTEK
Phaeodactylum_tricornutum_3 GCSP N SRR EGLTS QEAKARL ERYGPN QLTEK
Micromonas_pusilla_3 QT D KV KGLTA AEAAKRL LEDGPN ELEKP
Micromonas_sp_3 QT N AE TGLSA AEAAKRL EEDGPN ELEKP
Chlamydomonas_reinhardtii_5 VT D LH AGLNE SPAAAAADGLATAAASELGSVEAHR KAYGEN KFPEK
Chlamydomonas_reinhardtii_6 GT D LK EGLSD AGVDSSK QAFGVN SFPEK
Volvox_carteri_3 CT D LH HGLTE QDDKTGVNAHR AAYGPN TFPEK
Volvox_carteri_4 CT D LH HGLTE QDDKTGVNAHR AAYGPN TFPEK
Volvox_carteri_5 GS S PD RGLNP DPNA TGHDSVSEHR RIFGAN KHAEV
Chlamydomonas_reinhardtii_7 SS S VE SGLNA DPQA AGDDSVLEHR RVFGEN KHAET
Chlamydomonas_reinhardtii_8 AT S LH EGLDP STVDAHA EAYGHN KFKET
Volvox_carteri_6 LS D LH KGLDP QGQGLASIEAHV DAYGEN KFPEV
Ostreococcus_lucimarinus_3 GC D LK RGLCD RA WASEERK ESYGVN EFEYP
Micromonas_sp_4 KT D PK VGLCGTELSE ESLARRK EAFGVN EFEYP
Selaginella_moellendorffii_5 ST S TK NGIED EV PKIERRR LLYGSN TYPQQ
A.thaliana_AT3G21180.1 KS N ME QGINE DE KEVIDRK NAFGSN TYPKK
Aquilegia_caerulea_5 KT N PE KGING DD ADILNRK NTFGSN TYPKK
A.thaliana_AT5G57110.2 KT N PE KGISG DD DDLLKRK TIYGSN TYPRK
Oryza_sativa_3 KT D TE KGISG DD SDLTARR NAFGSN TYPRK
Zea_mays_2 KT D TQ KGISG DD SDLLARK NAFGSN TYPRK
Zea_mays_3 KS D LD RGLSP DE SELMRRR DIFGAN TYPRK
Zea_mays_4 KS N LE KGVSP NE DELLQRK NVYGSN TYPRK
Selaginella_moellendorffii_6 HV N LE KGIEP DE ESVQHRR EAFGAN SYPTK
Selaginella_moellendorffii_7 KI D PQ KGIDA TP VDIKARR DAFGPN TYPLK
Aquilegia_caerulea_6 CT S TA NGLTT TG DLLSRRQ EIYGIN KFTES
Medicago_truncatula_3 ST S AT EGISN DA DLLDKRQ QIYGIN KFTES
A.thaliana_AT2G22950.1 KA C PN AGLSTG EP EQLSKRQ ELFGIN KFAES
A.thaliana_AT4G37640.1 KA S PT DGLST EA AQLSQRQ ELFGIN KFAES
Oryza_sativa_4 CT S PE DGLPK SR RRQAVRE ELFGIN RFAET
Zea_mays_5 ST S AS DGLDDS NE PMTAARQ ELFGVN RFAEA
Oryza_sativa_5 AT S PA DGLST AE ESIKRRQ DVYGLN KFTES
A.thaliana_AT1G27770.1 ST S IA SGIST SE DLLSVRK EIYGIN QFTES
Oryza_sativa_6 GT S LT NGIVT DK DLLNQRQ DIYGVN KFAET
Zea_mays_6 AT S LA DGITT DE LSLNQRQ GMYGVN KFTES
Aquilegia_caerulea_7 SV S LD DGVSE KDLPRRQ EIYGFN RYVEK
Oryza_sativa_7 KA S LE DGAKE TDIATRQ MLYGAN RHAEK
Selaginella_moellendorffii_8 LV S LD DGVSK DEIDKRK EAFGSN MYEEK
Selaginella_moellendorffii_9 HVH G IE HGIDP SELDARR RAFGSN TYKES
Oryza_sativa_8 AS G AE RGIRG DD ADVARRK KAFGSN TYPKP
Fragilariopsis_cylindrus_13 AT H PD HGAAE TSINKRR ELFGSN LLPSS
Pseudo-nitzschia_multiseries_4 AT N RD HGAAE STIETRR ETFGSN VLPST
Phaeodactylum_tricornutum_4 GT D PK AGLDR ETIETRR ACFGAN RLPSA
Thalassiosira_pseudonana_6 RS K PE DGITN EQ SDIEFRR EAFGTN AIADK
Phaeodactylum_tricornutum_5 RS S PE VGIFP SEVEKRR EAFGSN RIAPK
Phaeodactylum_tricornutum_6 RS S PE SGIDP REVEHRQ SVFGSN AIAAK

```
                                   101
Skeletonema_marinoi_ABC            IQ--------------RSLLSYIIEQFDDKLVRILLVVAMVSAFFG----LLEVKEKVGEWGLELLQPLLKPLLHLLHHGDGDSSHSTKSSSIAEEVVKE
Thalassiosira_pseudonana_1         -------------------MSYIIEQFDDKLVRILLVVACVSAFFG----LVELKEEMGEW---------------------------------------
Thalassiosira_oceanica_1           PQ--------------RSLFSFILEQFDDKLVRILLAVALVSAFFG----LLELKEEMGDVAGQMLHHILG-----LFHGEA-GGPSASSASIAKEVVNE
Phaeodactylum_tricornutum_1        KS--------------KSTWQLILEQFEDRLVQILLVVALLSGVFS----YFEVRQ---------------------------------SATAAAELATD
Fragilariopsis_cylindrus_1         PP--------------PSYYELVKEQFEDQLVRILVFVAILSAILE----YSTSSG---------------------------------SGNGNG-ITTE
Fragilariopsis_cylindrus_2         PP--------------PSYYELVKEQFEDQLVRILVFVAILSAILE----YSTSSG---------------------------------SGNGNG-ITTE
Fragilariopsis_cylindrus_3         ----------------------VKEQFEDQLVRILVFVAILSAILE----YSTSSG---------------------------------SGNGNG-ITTE
Fragilariopsis_cylindrus_4         PP--------------PSYYELVKEQFEDQLVRILVFVAILSAILE----YSTSSG---------------------------------SGNGNG-ITTE
Fragilariopsis_cylindrus_5         PP--------------PSYYELVKEQFEDQLVRILVFVAILSAILE----YSTSSG---------------------------------SGNGNG-ITTE
Fragilariopsis_cylindrus_6         PP--------------PSYYELVKEQFEDQLVRILVFVAILSAILE----YSTSSG---------------------------------SGNGNG-ITTE
Fragilariopsis_cylindrus_7         PP--------------PSYYELVKEQFEDQLVRILVFVAILSAILE----YSTSSG---------------------------------SGNGNG-ITTE
Fragilariopsis_cylindrus_8         PP--------------PSYYELVKEQFEDQLVRILVFVAILSAILE----YSTSSG---------------------------------SGNGNG-ITTE
Pseudo-nitzschia_multiseries_1     ----------------------IKEQFEDRLVRILMGVAVLSALLE----YTSSGG---------------------------------GG------AVA
Thalassiosira_pseudonana_2         RK--------------KTMWELWLHQFDDGLVKILVSVAFASAAFI-----------------------------------------------------R
Ostreococcus_lucimarinus_1         PG--------------KALWRLVLEQFDDALVKVLLAAAAVSLALA----YAEG--------------------------------------------GA
Zea_mays_1                         AP--------------PSVWKLVLEQFNDTLVRILLLAAVVSFVLA----LYDG------------------------------------------AEGG
Oryza_sativa_1                     AA--------------PSVWKLVLEQFDDTLVRILLAAAVVSFVLA----LYDG------------------------------------------AEGG
Aquilegia_caerulea_1               DG--------------PSIWKLVLDQFNDTLVRILLVAAVISFVLA----WYDG------------------------------------------DEGG
A.thaliana_AT1G07810.1             EG--------------TSIFKLILEQFNDTLVRILLAAAVISFVLA----FFDG------------------------------------------DEGG
A.thaliana_AT1G07670.1             EG--------------TSIFKLILEQFNDTLVRILLAAAVISFVLA----FFDG------------------------------------------DEGG
Medicago_truncatula_1              DG--------------QSIWKLVLEQFNDTLVRILLAAAIISFVLA----WYDG------------------------------------------DEGG
Glycine_max_1                      DG--------------QSIWSLVLEQFNDTLVRILLVAAIISFVLA----WYDG------------------------------------------DEGG
Glycine_max_2                      EG--------------QSIWSLILEQFNDTLVRILLAAAIISFVLA----WYDG------------------------------------------DEGG
Aquilegia_caerulea_2               DG--------------VSIWSLILEQFNDTLVRILLAAAVISFVLA----WYDG------------------------------------------DEGG
Selaginella_moellendorffii_1       NG--------------KPMWKLVLEQFDDMLVKILLVAAVLSFFLT----YLDG------------------------------------------QKSG
Physcomitella_patens_1             PG--------------KPMWKLVLEQFDDMLVKILLVAALISFILA----YIDG------------------------------------------HSPD
Physcomitella_patens_2             PG--------------KPMWKLVLEQFDDMLVKILLVAALISFILA----YTDG------------------------------------------YSAD
Physcomitella_patens_3             PG--------------KPMWKLVLEQFDDTLVKILLFAAFVSLVLA----YID-------------------------------------------DHSA
Selaginella_moellendorffii_2       RG--------------KPLWKLVLEQFDDTLVRILLGAAVVSFLLT----YIDGK-----------------------------------------NKGA
Glycine_max_3                      KG--------------KPLWELVLEQFDDMLVKILLAAAFISFLLA----YFHG------------------------------------------SDSG
Glycine_max_4                      KG--------------KPLWELVLEQFDDMLVKILLAAAFISFLLA----YFHG------------------------------------------SDSE
Aquilegia_caerulea_3               KG--------------KPLWKLVLEQFDDMLVKILLVAAFISFVLA----YLHG------------------------------------------HESG
A.thaliana_AT4G00900.1             KG--------------KPLWHLVLEQFDDTLVKILLGAAFISFVLA----FLGE------------------------------------------EHGS
Chlamydomonas_reinhardtii_1        SK--------------QSIWAMIVEQFEDTLVRILLLAAVVSFALA----YFEE-------------------------------------------GAH
Volvox_carteri_1                   AK--------------QSIWAMIAEQFEDTLVRILLLAAIVSFGLA----WFEE-------------------------------------------DSH
Micromonas_pusilla_1               DG--------------KPLWKLVLEQFDDALVKILLAAAAVSFALV----WVEDR-----------------------------------------APGA
Micromonas_sp_1                    EG--------------KPLWKLVLEQFDDALVKILLVAALVSFVLA----FTEER-----------------------------------------APGV
Selaginella_moellendorffii_3       EG--------------TSFWKLVLEQFDDTLVQILLAAAGVSFALA----FSEL------------------------------------------EPGS
Thalassiosira_oceanica_2           DP--------------TPLWELVLEQFDDTLVKILLAAAGVSFALA----YFDDAG----------------------------------------EHAD
Thalassiosira_pseudonana_3         ES--------------TPLWKLVLEQFDDTLVKILLAAAAVSFALA----FFDDSG----------------------------------------SHSD
Pseudo-nitzschia_multiseries_2     EK--------------TPLWQLVLEQFDDKLVQILLGAALLSFILA----FFEE---------------------------------------------V
Fragilariopsis_cylindrus_9         ER--------------TPLWKLVLEQFDDKLVQILLFAALLSFVLA----FFEDI-------------------------------------------EG
Fragilariopsis_cylindrus_10        ER--------------TPLWKLVLEQFDDKLVQILLFAALLSFVLA----FFEDI-------------------------------------------EG
Aquilegia_caerulea_4               ES--------------TPFWRLVLKQFDDLLVKILIAAALVSFILA----LVDG----------------------------------------------
Glycine_max_5                      QR--------------VPFWKMVLKQFDDLLVKILIAAALISFILA----LING----------------------------------------------
Medicago_truncatula_2              QR--------------APFWKLVLKQFDDLLVKILIAAALISFILA----LING----------------------------------------------
A.thaliana_AT1G10130.1             KR--------------TPFWKLVLKQFDDLLVKILIVAAIVSFVLA----LANG----------------------------------------------
Oryza_sativa_2                     RG--------------TPFWKLVLKQFDDLLVKILIAAAVISFLLA----RMNG----------------------------------------------
Selaginella_moellendorffii_4       AG--------------ASFWKLVLKQFDDLLVKILIFAAITSLVLA----VVDG----------------------------------------------
Micromonas_pusilla_2               LG--------------TPFWKLVLKQFDDLLVKTLIAAAVVSFVLG----VVDG----------------------------------------------
Micromonas_sp_2                    QK--------------KSFLKLVLKQFDDVLVKILLAAALTSLILG----MANS----------------------------------------------
Volvox_carteri_2                   EA--------------TPLWKLILKQFDDLLVKILLAAAVADFIIA----LSDG----------------------------------------------
Chlamydomonas_reinhardtii_2        EA--------------TPLWKLILKQFDDLLVKILLAAAVADFIIA----MSDG----------------------------------------------
Dunaliella_salina_1                QS--------------TPLWKLILKQFDDLLVKILLGAAIVDFIIA----MSEG----------------------------------------------
Ostreococcus_lucimarinus_2         RE--------------TSFASLVIKQFDDTMVKVLLLAAFVSLALA----LWDG----------------------------------------------
Phaeodactylum_tricornutum_2        DGDKHKTFMSQYTACVSPIFTALVSQLKEPLILMLLGSAAVSIALG------------------------------------------------------
Chlamydomonas_reinhardtii_3        EK--------------ISFWRRLWNQLNNMIIGLLFAAAIVEG---------------------------------------------------------
Chlamydomonas_reinhardtii_4        EK--------------QGFWRRLWNQVNNMIIGLLFAAAIVEG---------------------------------------------------------
Thalassiosira_pseudonana_4         VK--------------TTLLQRIWHQINNVLVYVLITVAVVSLIQA----VTTNN--------------------------------------------S
Thalassiosira_pseudonana_5         IK--------------KTIWQRIWHQLNNVLVYVLITVAVVSL---------------------------------------------------------
```

```
Fragilariopsis_cylindrus_11       EK--------------EGLLKKIWNQVNNILVLILVIVAVISVVTA----FVIPS-------------------------------------------AV
Fragilariopsis_cylindrus_12       EK--------------EGLLKKIWNQVNNILVLILVIVAVISVVTA----FVIPS-------------------------------------------AV
Pseudo-nitzschia_multiseries_3    EK--------------ETLLQKIWNQVNNILVLILVIVAVISLISA----FVIPP-------------------------------------------DI
Phaeodactylum_tricornutum_3       EK--------------VTLLQRIWKQVNNVLVGILVFVAVVSLAKG----IST---------------------------------------------SG
Micromonas_pusilla_3              PR--------------VSLLVLFLIQLNSVIMYLLMGAVVASAAIKATG-------------------------------------------------DD
Micromonas_sp_3                   PR--------------VSLLVLFLIQLNSVIMYLLMAAVVASAAIKATG-------------------------------------------------DD
Chlamydomonas_reinhardtii_5       PP--------------PNFFMMLLEAAKDPMIIILLIVAIVTIVLGAA--VP-----------------------------------------------E
Chlamydomonas_reinhardtii_6       PP--------------PSFLSMLLEAAKDPMIVILLIVAIITIVLGAA--VP-----------------------------------------------E
Volvox_carteri_3                  PP--------------PSFLSMLLEASKDPMIIILLVVALVTIVLGAA--VP-----------------------------------------------E
Volvox_carteri_4                  PP--------------PSFLSMLLEASKDPMIIILLVVALVTIVLGAA--VP-----------------------------------------------E
Volvox_carteri_5                  PP--------------KNFFVLVWEVVQDPILILLIAAAAVSTILGSA--IP-----------------------------------------------E
Chlamydomonas_reinhardtii_7       PP--------------KNFFFLVWEVVQDPILILLIAAATVSTVLGAA--IP-----------------------------------------------E
Chlamydomonas_reinhardtii_8       PP--------------KSFFSLVWENLQDPVIIILCVAAAVSTALGAA--IP-----------------------------------------------E
Volvox_carteri_6                  PP--------------KSFLALVWGNLQDPVIIILIIAALVSTILGAA--IA-----------------------------------------------E
Ostreococcus_lucimarinus_3        PP--------------KSFWELCKDALGDLTVRILIAASVVSLAVGAG--MK-----------------------------------------------S
Micromonas_sp_4                   PP--------------KSFLQLCRDALDDLTVQILCVAAIISLGIGAG--LP-----------------------------------------------K
Selaginella_moellendorffii_5      SP--------------KGFLAFLWEACQDLTLVILGVCAVVSLALA----LA-----------------------------------------------T
A.thaliana_AT3G21180.1            KG--------------KNFFMFLWEAWQDLTLIILIIAAVTSLALG----IK-----------------------------------------------T
Aquilegia_caerulea_5              KG--------------RSFLNFLWEAWQDLTLIILMVAAAASLALG----IK-----------------------------------------------T
A.thaliana_AT5G57110.2            KG--------------KGFLRFLWDACHDLTLIILMVAAVASLALG----IK-----------------------------------------------T
Oryza_sativa_3                    KG--------------RSFLAFLWDACKDLTLIILMVAAAVSLALG----IT-----------------------------------------------T
Zea_mays_2                        KG--------------RSFLAFVWDACKDLTLIILMVAAAVSLALG----IT-----------------------------------------------T
Zea_mays_3                        ER--------------RSIWHFVFEACQDLTLVILMVAAAISFSLG----MA-----------------------------------------------T
Zea_mays_4                        KR--------------KNILRFVFEACQDLTLVILMIAAAISLTLG----MT-----------------------------------------------T
Selaginella_moellendorffii_6      AG--------------KSFW--------------------------------------------------------------------------------
Selaginella_moellendorffii_7      KR--------------TPFYMYVWEALQDETLMILILCAIVSLAVG----LT-----------------------------------------------T
Aquilegia_caerulea_6              EV--------------RSFWVFVWEALQDMTLMILGVCAFVSLIVG----LI-----------------------------------------------M
Medicago_truncatula_3             QA--------------KSFWVFVWEALQDMTLMILGVCALVSLIVG----IA-----------------------------------------------T
A.thaliana_AT2G22950.1            EL--------------RSFWVFVWEALQDMTLMILGVCAFVSLIVG----IA-----------------------------------------------T
A.thaliana_AT4G37640.1            EM--------------RGFWVFVWEALQDMTLMILGVCAFVSLIVG----IA-----------------------------------------------T
Oryza_sativa_4                    ES--------------RSFWVFVWEALQDMTLMILAACAFFSLVVG----IA-----------------------------------------------T
Zea_mays_5                        EP--------------RSFWVFVWEALQDMTLMILAACALVSLVVG----IA-----------------------------------------------T
Oryza_sativa_5                    EV--------------RSFWVFVWEALQDTTLIILAVCAFVSLVVG----IA-----------------------------------------------M
A.thaliana_AT1G27770.1            PS--------------RGFWLFVWEALQDTTLMILAACAFVSLIVG----IL-----------------------------------------------M
Oryza_sativa_6                    EI--------------RSFWEFVWEALEDTTLIILSACAIFSLVVG----IT-----------------------------------------------T
Zea_mays_6                        EA--------------RSLWEFVWEALQDTTLVILIACALVSFVVG----IA-----------------------------------------------T
Aquilegia_caerulea_7              PS--------------KGFFMFVWEALQDLTLMILMVCAVVSIGVG----LA-----------------------------------------------T
Oryza_sativa_7                    PP--------------RSFWMFVWDALHDLTLIILVVCALVSIVVG----LA-----------------------------------------------T
Selaginella_moellendorffii_8      PP--------------KGFWVFVWEAMHDLTLAILGFCAILSLVIG----VL-----------------------------------------------T
Selaginella_moellendorffii_9      PQ--------------RSVFSYILDASQDLTLLILVVCALVSIAVG----IA-----------------------------------------------T
Oryza_sativa_8                    KP--------------KGFFRHVWDALADVFLIVLLVCAAVSLAFG----IK-----------------------------------------------E
Fragilariopsis_cylindrus_13       PR--------------KSFYQLFIDTFDDATLQILIVAAIVSLCVG----MY------------------------------------------------
Pseudo-nitzschia_multiseries_4    PR--------------QSFWQLFVDTFDDATLQILIVAAIVSLAIG----IY------------------------------------------------
Phaeodactylum_tricornutum_4       PR--------------KTFGQLFLDTFDDATLQILIVAALVSLAVG----LY------------------------------------------------
Thalassiosira_pseudonana_6        KL--------------DSFLKLCWNAVQDFVLIMLIVLGVIGIVVETTIGLD----------------------------------------------PG
Phaeodactylum_tricornutum_5       KI--------------ESFCKLCWHAIQDFVLVMLIVLGFISIGVEIYS-LE----------------------------------------------ND
Phaeodactylum_tricornutum_6       SL--------------DSFFRLCWEAVQDFVLIMLIVLGIVSIVIEVGT-LE----------------------------------------------DG
```

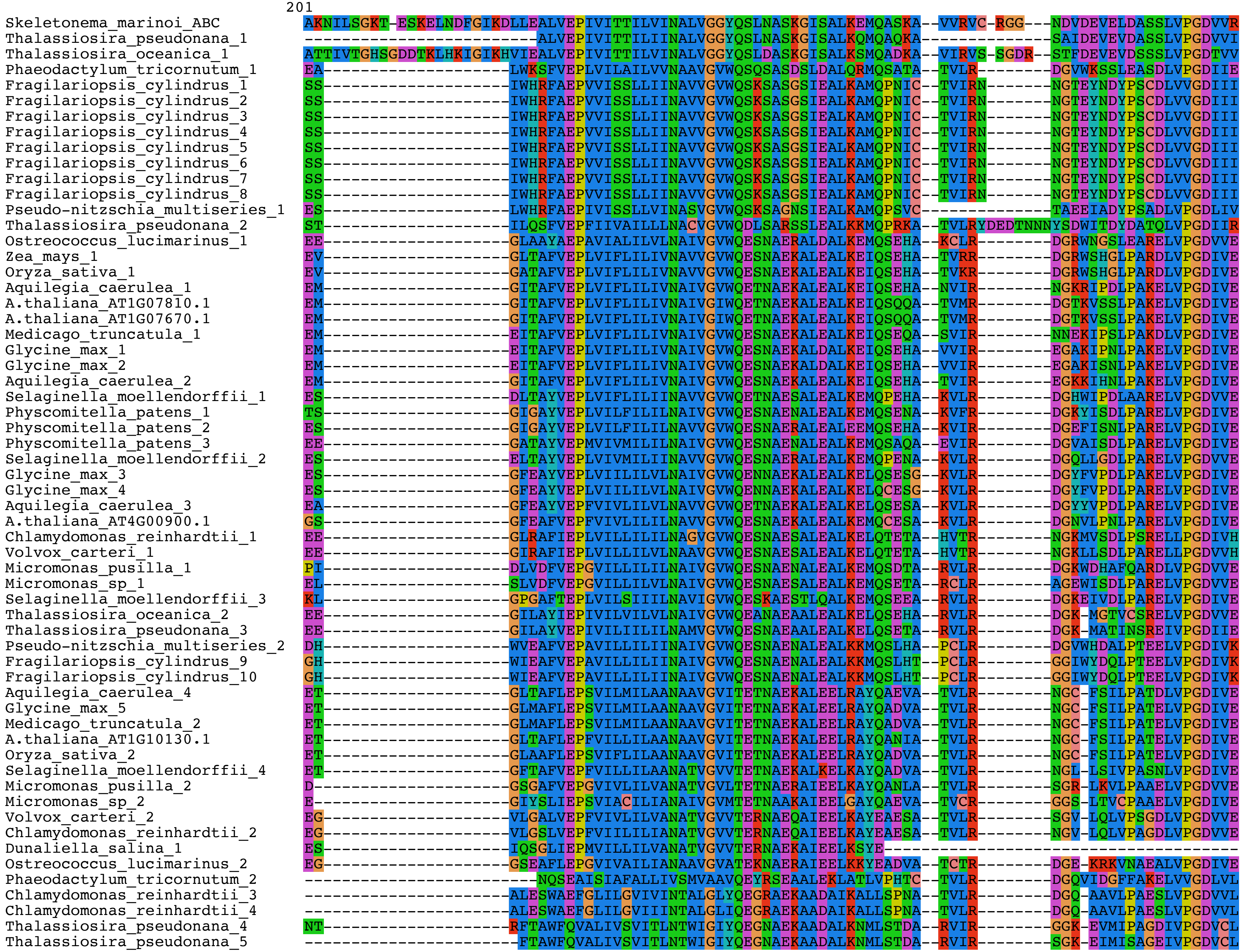
201
Skeletonema_marinoi_ABC AKNILSGKT-ESKELNDFGIKDLLEALVEPIVITTILVINALVGGYQSLNASKGISALKEMQASKA--VVRVC-RGG---NDVDEVELDASSLVPGDVVR
Thalassiosira_pseudonana_1 -------------------------ALVEPIVITTILIINALVGGYQSLNASKGISALKQMQAQKA--------------SAIDEVEVDASSLVPGDVVI
Thalassiosira_oceanica_1 ATTIVTGHSGDDTKLHKIGIKHVIEALVEPIVITTILVINALVGGYQSLDASKGISALKSMQADKA--VIRVS-SGDR--STFDEVEVDSSSLVPGDTVV
Phaeodactylum_tricornutum_1 EA--------------------LWKSFVEPLVILAILVVNAAVGVWQSQSASDSLDALQRMQSATA--TVLR---------DGVWKSSLEASDLVPGDIIE
Fragilariopsis_cylindrus_1 SS--------------------IWHRFAEPVVISSLLIINAVVGVWQSKSASGSIEALKAMQPNIC--TVIRN--------NGTEYNDYPSCDLVVGDIII
Fragilariopsis_cylindrus_2 SS--------------------IWHRFAEPVVISSLLIINAVVGVWQSKSASGSIEALKAMQPNIC--TVIRN--------NGTEYNDYPSCDLVVGDIII
Fragilariopsis_cylindrus_3 SS--------------------IWHRFAEPVVISSLLIINAVVGVWQSKSASGSIEALKAMQPNIC--TVIRN--------NGTEYNDYPSCDLVVGDIII
Fragilariopsis_cylindrus_4 SS--------------------IWHRFAEPVVISSLLIINAVVGVWQSKSASGSIEALKAMQPNIC--TVIRN--------NGTEYNDYPSCDLVVGDIII
Fragilariopsis_cylindrus_5 SS--------------------IWHRFAEPVVISSLLIINAVVGVWQSKSASGSIEALKAMQPNIC--TVIRN--------NGTEYNDYPSCDLVVGDIII
Fragilariopsis_cylindrus_6 SS--------------------IWHRFAEPVVISSLLIINAVVGVWQSKSASGSIEALKAMQPNIC--TVIRN--------NGTEYNDYPSCDLVVGDIII
Fragilariopsis_cylindrus_7 SS--------------------IWHRFAEPVVISSLLIINAVVGVWQSKSASGSIEALKAMQPNIC--TVIRN--------NGTEYNDYPSCDLVVGDIII
Fragilariopsis_cylindrus_8 SS--------------------IWHRFAEPVVISSLLIINAVVGVWQSKSASGSIEALKAMQPNIC--TVIRN--------NGTEYNDYPSCDLVVGDIII
Pseudo-nitzschia_multiseries_1 ES--------------------LWHRFAEPIVISSLLVINASVGVWQSKSAGNSIEALKAMQPSVC--------------TAEEIADYPSADLVPGDLIV
Thalassiosira_pseudonana_2 ST--------------------ILQSFVEPFIIVAILLLNACVGVWQDLSARSSLEALKKMQPRKA--TVLRYDEDTNNNYSDWITDYDATQLVPGDIIR
Ostreococcus_lucimarinus_1 EE--------------------GLAAYAEPAVIALILVLNAIVGVWQESNAERALDALKEMQSEHA--KCLR---------DGRWNGSLEARELVPGDVVE
Zea_mays_1 EV--------------------GLTAFVEPLVIFLILIVNAVVGVWQESNAEKALEALKEIQSEHA--TVRR---------DGRWSHGLPARDLVPGDIVE
Oryza_sativa_1 EV--------------------GATAFVEPLVIFLILIVNAVVGVWQESNAEKALEALKEIQSEHA--TVKR---------DGRWSHGLPARDLVPGDIVE
Aquilegia_caerulea_1 EM--------------------GITAFVEPLVIFLILIVNAIVGVWQETNAEKALEALKEIQSEHA--NVIR---------NGKRIPDLPAKELVPGDIVE
A.thaliana_AT1G07810.1 EM--------------------GITAFVEPLVIFLILIVNAIVGIWQETNAEKALEALKEIQSQQA--TVMR---------DGTKVSSLPAKELVPGDIVE
A.thaliana_AT1G07670.1 EM--------------------GITAFVEPLVIFLILIVNAIVGIWQETNAEKALEALKEIQSQQA--TVMR---------DGTKVSSLPAKELVPGDIVE
Medicago_truncatula_1 EM--------------------EITAFVEPLVIFLILIVNAIVGVWQESNAEKALEALKEIQSEQA--SVIR---------NNEKIPSLPAKDLVPGDIVE
Glycine_max_1 EM--------------------EITAFVEPLVIFLILIVNAIVGVWQESNAEKALDALKEIQSEHA--VVIR---------EGAKIPNLPAKELVPGDIVE
Glycine_max_2 EM--------------------EITAFVEPLVIFLILIVNAIVGVWQESNAEKALDALKEIQSEHA--VVIR---------EGAKISNLPAKELVPGDIVE
Aquilegia_caerulea_2 EM--------------------GITAFVEPLVIFLILIVNAIVGVWQESNAEKALEALKEIQSEHA--TVIR---------EGKKIHNLPAKELVPGDIVE
Selaginella_moellendorffii_1 ES--------------------DLTAYVEPLVIFLILIINAVVGVWQETNAESALEALKEMQPEHA--KVLR---------DGHWIPDLAARELVPGDVVE
Physcomitella_patens_1 TS--------------------GIGAYVEPLVILFILILNAIVGVWQESNAENALEALKEMQSENA--KVFR---------DGKYISDLPARELVPGDIVE
Physcomitella_patens_2 ES--------------------GIGAYVEPLVILFILILNAVVGVWQESNAERALEALEEMQSEHA--KVIR---------DGEFISNLPARELVPGDIVE
Physcomitella_patens_3 EE--------------------GATAYVEPMVIVMILILNAIVGVWQESNAENALEALKEMQSAQA--EVIR---------DGVAISDLPARELVPGDIVE
Selaginella_moellendorffii_2 ES--------------------ELTAYVEPLVIVMILIINAVVGVWQESNAERALEALKEMQPENA--KVLR---------DGQLLGDLPARELVPGDVVE
Glycine_max_3 ES--------------------GFEAYVEPLVIILILVLNAIVGVWQENNAEKALEALKELQSESG--KVLR---------DGYFVPDLPAKELVPGDIVE
Glycine_max_4 ES--------------------GFEAYVEPLVIILILVLNAIVGVWQENNAEKALEALKELQCESG--KVLR---------DGYFVPDLPARELVPGDIVE
Aquilegia_caerulea_3 EA--------------------GFEAYVEPFVIVLILVLNAVVGVWQETNAEKALEALKELQSESA--KVLR---------DGYYVPDLPARELVPGDIVE
A.thaliana_AT4G00900.1 GS--------------------GFEAFVEPFVIVLILILNAVVGVWQESNAEKALEALKEMQCESA--KVLR---------DGNVLPNLPARELVPGDIVE
Chlamydomonas_reinhardtii_1 EE--------------------GLRAFIEPLVILLILILNAGVGVWQESNAESALEALKELQTETA--HVTR---------NGKMVSDLPSRELLPGDIVH
Volvox_carteri_1 EE--------------------GIRAFIEPLVILLILVLNAAVGVWQESNAESALEALKELQTETA--HVTR---------NGKLLSDLPARELLPGDVVH
Micromonas_pusilla_1 PI--------------------DLVDFVEPGVILLILILNAIVGVWQESNAENALEALKEMQSDTA--RVLR---------DGKWDHAFQARDLVPGDVVE
Micromonas_sp_1 EL--------------------SLVDFVEPGVILLILVLNAIVGVWQESNAESALEALKEMQSETA--RCLR---------AGEWISDLPARELVPGDVVE
Selaginella_moellendorffii_3 KL--------------------GPGAFTEPLVILSIIILNAVIGVWQESKAESTLQALKEMQSEEA--RVLR---------DGKEIVDLPARELVPGDIVE
Thalassiosira_oceanica_2 EE--------------------GILAYIEPIVILVILVLNAIVGVWQEANAEAALEALKELQSEHA--RVLR---------DGK-MGTVCSRELVPGDVVE
Thalassiosira_pseudonana_3 EE--------------------GILAYVEPIVILIILILNAMVGVWQESNAEAALEALKELQSETA--RVLR---------DGK-MATINSREIVPGDIIE
Pseudo-nitzschia_multiseries_2 DH--------------------WVEAFVEPAVILLILIINAIVGVWQESNAENALEALKKMQSLHA--PCLR---------DGVWHDALPTEELVPGDIVK
Fragilariopsis_cylindrus_9 GH--------------------WIEAFVEPAVILLILIINAIVGVWQESNAENALEALKKMQSLHT--PCLR---------GGIWYDQLPTEELVPGDIVK
Fragilariopsis_cylindrus_10 GH--------------------WIEAFVEPAVILLILIINAIVGVWQESNAENALEALKKMQSLHT--PCLR---------GGIWYDQLPTEELVPGDIVK
Aquilegia_caerulea_4 ET--------------------GLTAFLEPSVILMILAANAAVGVITETNAEKALEELRAYQAEVA--TVLR---------NGC-FSILPATDLVPGDIVE
Glycine_max_5 ET--------------------GLMAFLEPSVILMILAANAAVGVITETNAEKALEELRAYQADVA--TVLR---------NGC-FSILPATELVPGDIVE
Medicago_truncatula_2 ET--------------------GLMAFLEPSVILMILAANAAVGVITETNAEKALEELRAYQADVA--TVLR---------NGC-FSILPATELVPGDIVE
A.thaliana_AT1G10130.1 ET--------------------GLTAFLEPFVILLILAANAAVGVITETNAEKALEELRAYQANIA--TVLR---------NGC-FSILPATELVPGDIVE
Oryza_sativa_2 ET--------------------GLAAFLEPSVIFLILAANAAVGVITETNAEKALEELRAYQADVA--TVLR---------NGC-FSILPATELVPGDIVE
Selaginella_moellendorffii_4 ET--------------------GFTAFVEPFVILLILAANATVGVVTETNAEKALKELKAYQADVA--TVLR---------NGL-LSIVPASNLVPGDIVE
Micromonas_pusilla_2 D---------------------GSGAFVEPGVIVLILVANATVGVLTETNAERAIEELKAYQANLA--TVLR---------SGR-LKVLPAAELVPGDVVE
Micromonas_sp_2 E---------------------GIYSLIEPSVIACILIANAIVGVMTETNAAKAIEELGAYQAEVA--TVCR---------GGS-LTVCPAAELVPGDIVE
Volvox_carteri_2 EG--------------------VLGALVEPFVIVLILVANATVGVVTERNAEQAIEELKAYEAESA--TVLR---------SGV-LQLVPSGDLVPGDVVE
Chlamydomonas_reinhardtii_2 EG--------------------VLGSLVEPFVIILILIANATVGVVTERNAEQAIEELKAYEAESA--TVLR---------NGV-LQLVPAGDLVPGDVVE
Dunaliella_salina_1 ES--------------------IQSGLIEPMVILLILVANATVGVVTERNAEKAIEELKSYE--------------------------------------
Ostreococcus_lucimarinus_2 EG--------------------GSEAFLEPGVIVAILIANAAVGVATEKNAERAIEELKKYEADVA--TCTR---------DGE-KRKVNAEALVPGDIVE
Phaeodactylum_tricornutum_2 -------------------------NQSEAISIAFALLIVSMVAAVQEYRSEAALEKLATLVPHTC--TVLR---------DGQVIDGFFAKELVVGDLVL
Chlamydomonas_reinhardtii_3 ----------------------ALESWAEFGLILGVIVINTALGLYQEGRAEKAADAIKALLSPNA--TVLR---------DGQ-AAVLPAESLVPGDVVL
Chlamydomonas_reinhardtii_4 ----------------------ALESWAEFGLILGVIIINTALGLIQEGRAEKAADAIKALLSPNA--TVLR---------DGQ-AAVLPAESLVPGDVVL
Thalassiosira_pseudonana_4 NT--------------------RFTAWFQVALIVSVITLNTWIGIYQEGNAEKAADALKNMLSTDA--RVIR---------GGK-EVMIPAGDIVPGDVCL
Thalassiosira_pseudonana_5 -----------------------FTAWFQVALIVSVITLNTWIGIYQEGNAEKAADALKNMLSTDA--RVIR---------SGK-EIMISAGEIVPGDVCL

```
Fragilariopsis_cylindrus_11        NP--------------------TYTNWIQVAIIVGVIVANTVIGIVQEGSAENAAEALKNMLSSDA--VLVR--------DGV-ETKVPSQFIVPGDVVV
Fragilariopsis_cylindrus_12        NP--------------------TYTNWIQVAIIVGVIVANTVIGIVQEGSAENAAEALKNMLSSDA--VLVR--------DGV-ETKVPSQFIVPGDVVV
Pseudo-nitzschia_multiseries_3     NP--------------------RYTNFIQIGIILGVIVINTIIGLIQEGSAEEAAEALKNMLSSDA--IVIR--------DGV-TTKVPAQLLVPGDAVI
Phaeodactylum_tricornutum_3        ED--------------------RVTNFIEVGLITFVITLNTTIGIYQEGSAEKAAEALKNMLSSDA--ILIR--------DGK-EVKIPASDVVPGDVVV
Micromonas_pusilla_3               KD--------------------QFLSYIDSIAITIIVFINASIAAKAENNANDALEALSSLQAPIS--TLIR--------DGE-EIRVESSDIVRGDLVK
Micromonas_sp_3                    KD--------------------KFLSYVDSIAISIIILINATIAAVTENSANDALEALSSLQSPMC--TVIR--------GGE-EVSIESKNMVRGDIVK
Chlamydomonas_reinhardtii_5        QR--------------------AHQGWSEGLAVLGTALIVIFLGAGQDFSKERQFQKLNALKDVIDV-KVTR--------GGK-QVLVPNTEVVVGDIMF
Chlamydomonas_reinhardtii_6        QR--------------------AHQGWSEGLAVLGTALIVVFIGAGQDYSKERQFQKLNALKDNIEV-KVTR--------GGK-QVLVPNTEIVVGDVMF
Volvox_carteri_3                   QR--------------------AHDGWSEGLAVLGTALIVIFIGAGQDYSKELQFQKLNKLKDNIDV-KVTR--------SGR-QVLIPNTDVVVGDILF
Volvox_carteri_4                   QR--------------------AHDGWSEGLAVLGTALIVIFIGAGQDYSKELQFQKLNKLKDNIDV-KVTR--------SGR-QVLIPNTDVVVGDILF
Volvox_carteri_5                   ER--------------------KKNHWIEGVAIWVAVIVVTLVGAGNDYSKDLQFRKLNAQKDRIQI-KVIR--------GGE-QILVENTDLVVGDIVI
Chlamydomonas_reinhardtii_7        ER--------------------AKSAWVEGVAIWVAVIVVTLVGAGNDYSKDLQFRKLNAQKDRIEI-KVVR--------GGQ-QILVPNTDLVVGDVML
Chlamydomonas_reinhardtii_8        QR--------------------KHGEWIEGVAIWVAIILVVSVGAGNDYQKDKQFRKLNAQKDKIMV-KVVR--------GHQ-TLLVENVELVVGDVYL
Volvox_carteri_6                   QR--------------------KHGEWIEGVAIWVAIIIVVSVSAGNDYQKDRQFRKLNAQKDKIMV-KVVR--------GGH-TELIENTQLVVGDVYL
Ostreococcus_lucimarinus_3         HR--------------------EEYGYLEGIAIVLVVFVVVFLQAFIDYAKEMKFRQLNSVKDNYQV-KVHR--------DGK-SVAVPAGEIMVGDLVE
Micromonas_sp_4                    HR--------------------EEYGYLEGIAIVIVVFVVVFLQAYIDYVKEQKFRQLNSIKDNYAV-KVVR--------NGE-VHAVTAGEVLVGDVVE
Selaginella_moellendorffii_5       KV--------------------KFASWYDGASIAFTVILVVCVTACSDYKQSLQFQRLNAEKRKIHV-EVLR--------GGR-RIGVSIFELVVGDVVP
A.thaliana_AT3G21180.1             EG--------------------LKEGWLDGGSIAFAVLLVIVVTAVSDYRQSLQFQNLNDEKRNIQL-EVMR--------GGR-TVKISIYDVVVGDVIP
Aquilegia_caerulea_5               EG--------------------IKEGWYDGGSIAFAVLLVIVVTAISDYRQSLQFQNLNDEKRNIHL-EIVR--------GGK-RVEVSIFDIVVGDVIP
A.thaliana_AT5G57110.2             EG--------------------IKEGWYDGGSIAFAVILVIVVTAVSDYKQSLQFQNLNDEKRNIHL-EVLR--------GGR-RVEISIYDIVVGDVIP
Oryza_sativa_3                     EG--------------------IKEGWYDGASIAFAVLLVVVVTATSDYKQSLQFQNLNEEKQNIKL-EVVR--------GGR-RISVSIYDLVAGDVVP
Zea_mays_2                         EG--------------------IKEGWYDGASIAFAVLLVVFVTAISDYKQSLQFQNLNEEKQNIRL-EVVR--------GGR-RITVSIYDLVVGDVVP
Zea_mays_3                         EG--------------------VKDGWYDGGSIFFAVFLVIFVTATSDYRQSLQFQHLNEEKRNIQV-EVIR--------GGK-RLVASIFDLVVGDVVP
Zea_mays_4                         EG--------------------VDEGWYDGGSIFLAVFLVILVTATSDYRQSLQFRHLNEEKQNIQV-EVVR--------GGK-RFGASIFDLVVGDVVP
Selaginella_moellendorffii_6       ----------------------VKEGWYDGASIGFAVLVVIFVTAFSDYRQSLQFRSLSQEKRNIQI-QVVR--------GGR-RFTTSIFDLVVGDIVP
Selaginella_moellendorffii_7       EA-----------------------RWYDGGGICFAIVVCVMVASLSDYNQANQFQKLSAEKRKIYI-NVTR--------GGH-RTKVSIFELVVGDMVH
Aquilegia_caerulea_6               EG--------------------WPKGAHDGLGIAASILLVVFVTATSDYRQSLQFKDLDKEKKKITI-QVTR--------NGY-RQKISIYDLLPGDIVH
Medicago_truncatula_3              EG--------------------WPKGAHDGLGIVASILLVVFVTATSDYRQSLQFKDLDKEKKKISI-QVTR--------NGY-RQKMSIYELLPGDIVH
A.thaliana_AT2G22950.1             EG--------------------WPQGSHDGLGIVASILLVVFVTATSDYRQSLQFRDLDKEKKKITV-QVTR--------NGF-RQKMSIYDLLPGDVVH
A.thaliana_AT4G37640.1             EG--------------------WPKGSHDGLGIAASILLVVFVTATSDYRQSLQFRDLDKEKKKITV-QVTR--------NGF-RQKLSIYDLLPGDIVH
Oryza_sativa_4                     EG--------------------WPKGAHDGLGIVASILLVVFVTATSDYRQSLQFKDLDKEKKKITV-QVSR--------NGY-RQKLSIYDLLAGDIVH
Zea_mays_5                         EG--------------------WPHGAHDGLGIVASILLVVFVTATSDYRQSLQFKDLDKEKKKIAV-QVTR--------RGY-RQRLSIYDLLAGDIVH
Oryza_sativa_5                     EG--------------------WPKGAHDGLGIVASILLVVFVTATSDYRQSLQFKDLDKEKKKIQV-QVTR--------NGF-RQRLSIYDLLPGDVVH
A.thaliana_AT1G27770.1             EG--------------------WPIGAHDGLGIVASILLVVFVTATSDYRQSLQFKDLDAEKKKIVV-QVTR--------DKL-RQKISIYDLLPGDVVH
Oryza_sativa_6                     EG--------------------WPQGAHDGVGIVASILLVVSVTGTSNYQQSLQFRDLDKEKRKILV-QVTR--------NGL-RQRVLIDDLLPGDAVH
Zea_mays_6                         EG--------------------WPSGAHDGIGIFTSILLVVSVTATSNYQQSLQFRDLDREKRKISV-QVTR--------DGF-RQRILIDDLLPGDVVH
Aquilegia_caerulea_7               EG--------------------WPKGMYDGLGILLSILLVVMVTAVSDYKQSLQFKDLDKEKKKIMV-QVTR--------NGY-RQKVSIFDLVVGDIVH
Oryza_sativa_7                     KG--------------------WPMGIYDGFGIILSILLVVLVTATSDYQQARKFMELDREKQKIYI-RVTR--------DKK-TKEVLVHDLVVGDILH
Selaginella_moellendorffii_8       EG--------------------WKEGWYDGTGIALSIILVVFVTAASDYQQSLQFRDLDKEKKNILV-QVTR--------NHK-RQKVSIFDLVVGDVVH
Selaginella_moellendorffii_9       KG--------------------FRDGWCDGAGILVSVVLVITVSASSDYQQAVQFRALDKEKGKVYI-QVTR--------SAK-RRRILASELVVGDIVH
Oryza_sativa_8                     HG--------------------IKDGWYDGVSIFLAVFLVAAVSAVSNHSQGKRFDKLARESENIMV-SVVR--------AAR-RQEVSIFDVVVGDVVV
Fragilariopsis_cylindrus_13        DD--------------------PAAGYVEGMAILSACLIVSVVTALNDYQKETQFRELSVANDEIDV-LVIR--------SGK-FQKIPVGELVVGDLVS
Pseudo-nitzschia_multiseries_4     DD--------------------PEVGYVEGMAILAACLVVSVVTALNDYQKETQFRELSAANDEVDV-LVIR--------SGK-FQKVPVGDLVVGDLIS
Phaeodactylum_tricornutum_4        DD--------------------PATGYVEGCAILAAVLVVSFVTAVNDFQKESQFRELSAANDAVDV-LVVR--------NNV-HWQIPVDELVVGDVVC
Thalassiosira_pseudonana_6         EK--------------------CGMCWLEGAAILASVCIVVLVTAGIDYAKQFAFIRLTRSLNDTNTKMVIR--------NGH-QMSVTDDEIVVGDILS
Phaeodactylum_tricornutum_5        EE--------------------CTTCWIEGAAILASVCIVVFVSAGIDYAKQFAFLRLTRSLHETNTKQVIR--------EGK-QVSIIDDDLVVGDILS
Phaeodactylum_tricornutum_6        ED--------------------CKTCWIEGAAILVSVCIVVLVTASIDYAKQFAFIRLTRSLHDTNTKQVIR--------DAK-QVSVIDDDIVVGDILS
```

```
                                301
Skeletonema_marinoi_ABC         LSVGQ--KIPADIRLVSV-ST--SS-FTVDEACLTGESDSVNKIPYK----GDIKNDDMQ---NG--------------GGTMGANSNGMLYG-------
Thalassiosira_pseudonana_1      LTVGQ--KIPADIRLMSV-ST--ST-FTVDEACLTGESDSVPKIPYK----GDVQNDEEH----------------------NGHHANGMLYG-------
Thalassiosira_oceanica_1        LSIGE--KVPADVRLVSV-ST--ST-FTVDEACLTGESDSVAKTPYK----GDPAKDPAP---EG-------------GSGSMGEFASGMLYG-------
Phaeodactylum_tricornutum_1     LRVGD--KIPADSRLLSL-QS--SS-LQIDEGSLTGESVTVGKLP-----------GDEG---RA------------DSPNRPVQDQKGMLYS-------
Fragilariopsis_cylindrus_1      LKTGD--KIPADARLVSY-YKGRRT-LMVDEASLTGESISVEKLICDNNNDGTTTQSKEE---SN-------------NSISNHQFNDGMCYS-------
Fragilariopsis_cylindrus_2      LKTGD--KIPADARLVSY-YKGRRT-LMVDEASLTGESISVEKLICDNNNDGTTTQSKEE---SN-------------NSISNHQFNDGMCYS-------
Fragilariopsis_cylindrus_3      LKTGD--KIPADARLVSY-YKGRRT-LMVDEASLTGESISVEKLICDNNNDGTTTQSKEE---SN-------------NSISNHQFNDGMCYS-------
Fragilariopsis_cylindrus_4      LKTGD--KIPADARLVSY-YKGRRT-LMVDEASLTGESISVEKLICDNNNDGTTTQSKEE---SN-------------NSISNHQFNDGMCYS-------
Fragilariopsis_cylindrus_5      LKTGD--KIPADARLVSY-YKGRRT-LMVDEASLTGESISVEKLICDNNNDGTTTQSKEE---SN-------------NSISNHQFNDGMCYS-------
Fragilariopsis_cylindrus_6      LKTGD--KIPADARLVSY-YKGRRT-LMVDEASLTGESISVEKLICDNNNDGTTTQSKEE---SN-------------NSISNHQFNDGMCYS-------
Fragilariopsis_cylindrus_7      LKTGD--KIPADARLVSY-YKGRRT-LMVDEASLTGESISVEKLICDNNNDGTTTQSKEE---SN-------------NSISNHQFNDGMCYS-------
Fragilariopsis_cylindrus_8      LKTGD--KIPADARLVSY-YKGRRT-LMVDEASLTGESISVEKLICDNNNDGTTTQSKEE---SN-------------NSISNHQFNDGMCYS-------
Pseudo-nitzschia_multiseries_1  LKTGD--KIPADCRLVRY-YQGRRS-LAVDETCLTGESIAVEKLL-----------GQEG---LA-------------PSKAPVQSQSGMVFS-------
Thalassiosira_pseudonana_2      LRVGE--YIPADARLASL-TS--ST-MYVDESSLTGESVSVGKLP-----------GDEG---LPAG---------DDKKTIPIQDQSSMLFS-------
Ostreococcus_lucimarinus_1      LKTGD--RVPADCRVIRL-KT--AT-VRVEQASLTGESVAVDK-------------RVES---VK-------------DEDIELQGKTCMLFA-------
Zea_mays_1                      LRVGD--KVPADMRVLQL-IS--ST-LRVEQGSLTGETASVNK-------------TSHK---IE-------------LEDTDIQGKECMVFA-------
Oryza_sativa_1                  LRVGD--KVPADMRVLQL-IS--ST-LRVEQGSLTGETASVNK-------------TSHK---IE-------------LEDTDIQGKECMVFA-------
Aquilegia_caerulea_1            LRVGD--KIPADLRLLTL-IT--ST-VRVEQSSLTGESEAVNK-------------TNKP---VS-------------V-DTDIQGKRCMLFA-------
A.thaliana_AT1G07810.1          LRVGD--KVPADMRVVAL-IS--ST-LRVEQGSLTGESEAVSK-------------TTKH---VD-------------E-NADIQGKKCMVFA-------
A.thaliana_AT1G07670.1          LRVGD--KVPADMRVVAL-IS--ST-LRVEQGSLTGESEAVSK-------------TTKH---VD-------------E-NADIQGKKCMVFA-------
Medicago_truncatula_1           LKVGD--KVPADMRVVEL-IS--ST-LRLEQGSLTGESEAVNK-------------TNKP---VA-------------E-DADIQGKKCIVFA-------
Glycine_max_1                   LKVGD--KVPADMRVVEL-IS--ST-LRLEQGSLTGESEAVNK-------------TNKR---VD-------------E-DADIQGKRCMVFA-------
Glycine_max_2                   LKVGD--KVPADMRVVEL-IS--ST-LRSEQGSLTGESEAVNK-------------TNKR---VD-------------E-DADIQGKRCMVFA-------
Aquilegia_caerulea_2            LRVGD--KVPADMRVLSL-IS--ST-IRIEQCSLTGESEAVNK-------------TNKA---VA-------------V-ETDIQGKKCMVFA-------
Selaginella_moellendorffii_1    LRVGD--KVPADMRIAVL-RT--ST-LRVEQSSLTGESAAVVK-------------STQA---VE-------------LADVELQAKECMVFS-------
Physcomitella_patens_1          LRVGD--KVPADMRIVKL-KT--ST-VRVEQSSLTGESMSVTK-------------TSYA---VE--------------ENIELQGKECMVFA-------
Physcomitella_patens_2          LRVGD--KVPADMRIVKL-KT--ST-VRVEQSSLTGESMSVVK-------------TTHS---VK--------------ENIELQGKECMVFA-------
Physcomitella_patens_3          LRVGD--KVPADMRVISL-KT--ST-VRVEQSSLTGESMAVLK-------------SNQN---VE--------------EEIELQGKECMVFA-------
Selaginella_moellendorffii_2    LRVGD--KVPADMRIASL-KT--ST-FRIEQSSLTGESVPVIK-------------GLQA---VE-------------SEDIELQGKDCMVFA-------
Glycine_max_3                   LHVGD--KVPADMRVAAL-KT--ST-LRVEQSSLTGEAMPVLK-------------GTNP---VF-------------LDDCELQAKENMVFA-------
Glycine_max_4                   LHVGD--KAPADMRVAAL-KT--SI-LRVEQSSLTGEAMPVLK-------------GTNP---VF-------------LDDCELQAKENMVFA-------
Aquilegia_caerulea_3            LRVGD--KVPADMRVAVL-KT--SI-LRVEQSSLTGEAMPVIK-------------STNP---VF-------------MDDCELQAKECMVFA-------
A.thaliana_AT4G00900.1          LNVGD--KVPADMRVSGL-KT--ST-LRVEQSSLTGEAMPVLK-------------GANL---VV-------------MDDCELQGKENMVFA-------
Chlamydomonas_reinhardtii_1     LHVGD--RVPADCRVLAL-RT--AT-CRVEQASLTGESVAVNK-------------GSDP---VA-------------DPNCELQSKECMLFA-------
Volvox_carteri_1                LHVGD--KVPADCRMVAL-KT--AT-VRAEQASLTGESVAVNK-------------STDP---VA-------------DPNCELQAKECMVFA-------
Micromonas_pusilla_1            VRTGD--RVPADARVVTL-KT--AT-IRLEQASLTGESVAVNK-------------DIDA---ID-------------DPDAELQAKGCMLFG-------
Micromonas_sp_1                 VRTGD--RVPADCRVIRL-KT--AT-IRLEQASLTGESVAVNK-------------TTEP---VA-------------DAGCELQGKECVLFG-------
Selaginella_moellendorffii_3    LRAGD--KASADMRVAFL-KS--GT-IRLQQAALTGESQPVLK-------------QPDS---ES-------------DEEVEIQGKDNMVFA-------
Thalassiosira_oceanica_2        VKVGD--RVPADTRVSEL-RT--TS-LRIDQSQLTGESQSVAK-------------DPAAP-NVK-------------DDELVVQAKTNIMFA-------
Thalassiosira_pseudonana_3      VKVGD--RVPADTRVTEL-KT--TS-LRIDQSQLTGESQSVAK-------------FPEVP-NVG-------------EDELVVQAKTNIMFA-------
Pseudo-nitzschia_multiseries_2  ISVGD--RAPADVRLCKL-IT--AT-LRSDEGALTGESETVLK-------------TSNA---IA-------------S-GEKISDQKNMVFA-------
Fragilariopsis_cylindrus_9      ISVGD--RSPADCRLVSF-IT--AT-LRADEGALTGESETVLK-------------QLEP---TSSN----------SSNKSSISDQKNMVFA-------
Fragilariopsis_cylindrus_10     ISVGD--RSPADCRLVSF-IT--AT-LRADEGALTGESETVLK-------------QLEP---TSSN----------SSNKSSISDQKNMVFA-------
Aquilegia_caerulea_4            VGVGC--KVPADMRTVEM-LG--NQ-LRVDQAILTGESCSVAK-------------ELES---TS-------------ATNAVFQDKTNILFS-------
Glycine_max_5                   VSVGC--KIPADMRMIEM-LS--NQ-VRVDQAILTGESSSVEK-------------ELKT---TT-------------TTNAVYQDKTNILFS-------
Medicago_truncatula_2           VSVGC--KIPADMKMIDM-LS--NE-VRVDQAILTGESSSVEK-------------ELKT---TT-------------AANAVYQDKTNILFS-------
A.thaliana_AT1G10130.1          VTVGC--KIPADLRMIEM-SS--NT-FRVDQAILTGESCSVEK-------------DVDC---TL-------------TTNAVYQDKKNILFS-------
Oryza_sativa_2                  VGVGC--KVPADMRTIEM-LS--HQ-LRVDQAILTGESCSVAK-------------ELES---TS-------------TMNAVYQDKTNILFSNITNRDN
Selaginella_moellendorffii_4    VAVGC--KVPADMRVIDM-LS--SSALRVDQAILTGESSSVAK-------------ELEE---NP-------------VINPVYQDKTSILFS-------
Micromonas_pusilla_2            CVVGN--KVPADVRLVSI-AS--ST-FRVDQSILTGESGSVSK-------------ELTP---CA-------------SAKAVVQDKTCMLYS-------
Micromonas_sp_2                 LAVGD--RIPADIRLSGI-VG--ST-FRVDQAPLTGESESVTK-------------TIEK---IA-------------ATKAVLQDKTCIAFS-------
Volvox_carteri_2                VAVGA--KVPADIRLTAL-IG--SV-LRADQSILTGESHTVDK-------------QVRP---VL-------------KDNPVYQDKTNMLFS-------
Chlamydomonas_reinhardtii_2     VAVGA--KVPADTRLAGL-LG--NV-LRVDQSILTGESHSVDK-------------QVRP---VL-------------KDNPVYQDKTNMMFS-------
Dunaliella_salina_1             -VLGN--KVPADTRVAHV-YT--SS-LKTDQSLLTGESHAVEK-------------HTEV---VH-------------NKQAVYQDKLNMLFS-------
Ostreococcus_lucimarinus_2      IATGE--KVPADCRLVKI-HS--NV-LRCDQALLTGESGSVAK-------------TERA---VE------------HLGECVLQDKTCMVYS-------
Phaeodactylum_tricornutum_2     LATGD--RVPADCRVV---DS--VE-LILDESSLTGENHPVAK-------------TGEG---VVL------------GASPPLTQQKNVVFA-------
Chlamydomonas_reinhardtii_3     LKSGD--KVPADVRLL---SA--VN-LQVQEAMLTGESVPVSK-------------VLHP-----------------APAQAGLGDRKCMCFS-------
Chlamydomonas_reinhardtii_4     LKSGD--KVPADVRLL---TA--VN-LQVQEAMLTGESVPVSK-------------VLHP-----------------APAQAGLGDRKCMCFS-------
Thalassiosira_pseudonana_4      LGLGD--KIPADLRLI---SV--SN-LATGEAALTGESVPIDK-------------VRMS---V------------ANPDQVPLGDRKNMAYS-------
Thalassiosira_pseudonana_5      LGLGD--KVPADMRLI---SV--SN-LATGEAALTGESVPIDK-------------VTDA---IPCENG-------MDPDQVPLGDRKNMSYS-------
```

```
Fragilariopsis_cylindrus_11      LALGD--KIPCDLRVI---EV--SN-MASAEAALTGESVPIEK-------------TPLP---IELNEG-------QLPKQVPLGDRKNMCFS-------
Fragilariopsis_cylindrus_12      LALGD--KIPCDLRVI---EV--SN-MASAEAALTGESVPIEK-------------TPLP---IELNEG-------QLPKQVPLGDRKNMCFS-------
Pseudo-nitzschia_multiseries_3   LGLGD--RIPADLRVI---EV--SN-MASAEAALTGESVPIEK-------------TPQA---IPLNEG-------QLAKQVPLGDRKNMCFS-------
Phaeodactylum_tricornutum_3      LGTGD--RVPGDIRML---EV--NN-LACQEAALTGESVPIEK-------------VTDA---IDCGSG--------KPEQVPLGDRKNMCFS-------
Micromonas_pusilla_3             LGTGD--VVPADVRCI---TA--ND-LRVNEMLLTGEPEDVAK-------------STKVKP--------------RVPGHPEKLTADNMAFS-------
Micromonas_sp_3                  LGTGD--VVPADCRCI---KA--ND-FRVNEMLLTGEPEDVAK-------------NTKIKA--------------RVPGQPEKLTADNMAFS-------
Chlamydomonas_reinhardtii_5      LDTGD--KVIADGIVI---DS--QG-LVLDEASLTGESDPIKK-------------D---------------------------PVSDPWVRS-------
Chlamydomonas_reinhardtii_6      LDTGD--KVIADGVVI---DS--QG-IVLDEASLTGESDPIKK-------------D---------------------------AVSDPWIRS-------
Volvox_carteri_3                 LDTGD--KVIADGIVI---DS--QG-LVLDEASLTGESDPIKK-------------D---------------------------PLNDPWVRS-------
Volvox_carteri_4                 LDTGD--KVIADGIVI---DS--QG-LVLDEASLTGESDPIKK-------------D---------------------------PLNDPWVRS-------
Volvox_carteri_5                 LDTGD--KVVADAIVL---DS--QG-LTMDEASLTGESDPMKK-------------N---------------------------INEDPWVMS-------
Chlamydomonas_reinhardtii_7      LDTGD--KVVADAIVI---DS--QG-LTMDEASLTGESDPMKK-------------N---------------------------TTADPWVMS-------
Chlamydomonas_reinhardtii_8      LDTGD--KVVADGVCF---DC--QG-LVIDEASLTGESDPIKK-------------N---------------------------TDEDPWVRS-------
Volvox_carteri_6                 LDTGD--KVVADGICF---DS--QG-LVVDEASLTGESDPIKK-------------N---------------------------PEDDCWVRS-------
Ostreococcus_lucimarinus_3       LAAGD--KVPADALFV---EG--SK-FKANEAAMTGEPIDISK-------------S---------------------------REKDPWVLS-------
Micromonas_sp_4                  LSAGD--KVPADGVFL---EG--SK-LRADESAMTGEPIGIAK-------------S---------------------------HDKDPFLLS-------
Selaginella_moellendorffii_5     LKTGD--QIPADGVLV---DG--YS-LVVDESSLTGESDPVSM-------------PK--------------------------GLDHPFFMS-------
A.thaliana_AT3G21180.1           LRIGD--QVPADGVLI---SG--HS-LAIDESSMTGESKIVHK-------------D----------------------------QKSPFLMS-------
Aquilegia_caerulea_5             LKIGD--QVPADGVLV---VG--HS-LAIDESSMTGEAKIVHK-------------D---------------------------HRKDPFLMS-------
A.thaliana_AT5G57110.2           LNIGN--QVPADGVLI---SG--HS-LALDESSMTGESKIVNK-------------D---------------------------ANKDPFLMS-------
Oryza_sativa_3                   LKIGD--QVPADGILI---SG--HS-LSVDESSMTGESKIVHK-------------D----------------------------QKSPFLMS-------
Zea_mays_2                       LKIGD--QVPTDGILI---SG--HS-LSIDESSMTGESKIVHK-------------D----------------------------QKSPFLMS-------
Zea_mays_3                       LKIGD--QVPADGILI---YG--HS-LAIDESSMTGESKIVNK-------------D----------------------------QRAPFLMS-------
Zea_mays_4                       LKIGD--QVPADGVLI---SG--HS-LAIDESSMTGESKVVHK-------------D----------------------------QKAPFLMS-------
Selaginella_moellendorffii_6     LNIGD--QVPADGVLV---SG--HS-LSIDESSMTGESEPVHV-------------D----------------------------GKSPFLHS-------
Selaginella_moellendorffii_7     LAIGD--QIPADGLVY---VG--HS-LIVDESSMTGESDPLPK-------------D---------------------------EEEKPFLMS-------
Aquilegia_caerulea_6             LAIGD--QVPADGLFV---SG--FS-LSINESSLTGESDPVMV-------------N----------------------------AQNPYMLS-------
Medicago_truncatula_3            LAIGD--QVPADGLFV---SG--FS-LLIDESSLTGESEPVVV-------------N----------------------------TENPFLLS-------
A.thaliana_AT2G22950.1           LAIGD--QVPADGLFL---SG--FS-VVIDESSLTGESEPVMV-------------T----------------------------AQNPFLLS-------
A.thaliana_AT4G37640.1           LAIGD--QVPADGLFL---SG--FS-VVIDESSLTGESEPVMV-------------N----------------------------AQNPFLMS-------
Oryza_sativa_4                   LSIGD--QVPADGLFL---SG--FS-LLINESSLTGESEPVAV-------------N----------------------------AENPFLLS-------
Zea_mays_5                       LSIGD--QVPADGLFV---SG--FS-MLIDESSLTGESEPVAV-------------S----------------------------AENPFLLS-------
Oryza_sativa_5                   LAIGD--QVPADGLFI---SG--FS-LLINESSLTGESEPVVV-------------N----------------------------EDNPFLLS-------
A.thaliana_AT1G27770.1           LGIGD--QIPADGLFI---SG--FS-VLINESSLTGESEPVSV-------------S----------------------------VEHPFLLS-------
Oryza_sativa_6                   LAVGD--QVPADGLFI---SG--FS-VLVDESSLTGESEPVFV-------------N----------------------------EDNPYLLS-------
Zea_mays_6                       LGVGD--QVPADGLFV---SG--YS-VLVNESSLTGESEPVVI-------------S----------------------------EDNPFLLS-------
Aquilegia_caerulea_7             LSIGD--QVPADGFFL---SG--YS-LVIDESSLSGESEPVNI-------------N----------------------------QRNPFLLA-------
Oryza_sativa_7                   LSIGD--VVPADGLFI---SG--DC-LMIDESSLSGESEPVNI-------------S----------------------------EERPFLHA-------
Selaginella_moellendorffii_8     LSIGD--QVPADGLFI---SG--YS-LVIDESSMTGESEPQHV-------------G----------------------------KNKPFLLS-------
Selaginella_moellendorffii_9     LGIGD--QIPADGLLL---YG--QS-LLVDESCMTGESEMRAK-------------S----------------------------AEQPFLIS-------
Oryza_sativa_8                   LKIGD--VVPADGVFL---DG--HA-LQVDESSMTGEPHPVEV-------------D---------------------------AVKSPFLAS-------
Fragilariopsis_cylindrus_13      LEAGD--SIPCDGVLV---NY--DG-LEVDESALTGEPEDIDK-------------D---------------------------FENDPFLLS-------
Pseudo-nitzschia_multiseries_4   LEAGD--SIPCDGVVV---QY--DG-LTVDESALTGEPEDIEK-------------D---------------------------FENDPFLLS-------
Phaeodactylum_tricornutum_4      VEAGD--QIPCDGVLL---VA--DD-VQVDESALTGEPTDVDK-------------S---------------------------LQNDPFVLS-------
Thalassiosira_pseudonana_6       INAHNLASIPADCVVL--GPS--GG-LKMDESSLTGESVLIAK------------------------------------------NPGDVVLS-------
Phaeodactylum_tricornutum_5      INAHNLASIPADCVLL--GPA--TD-LKMDESTLTGESKAVSK------------------------------------------KPGDVVLS-------
Phaeodactylum_tricornutum_6      VNAHNLASIPADCVLL--GPA--GD-LKMDESTLTGESKAVSK------------------------------------------KPGDIILS-------
```

```
                                401
Skeletonema_marinoi_ABC         ------------------------------GTVITAGKGLGVVVRTGMDTEMGKIQRGVT--EA-AS-------DEQ-AHRTPLAIKLDEFGDKLTIIIG
Thalassiosira_pseudonana_1      ------------------------------GTVITAGKGVGVVVRTGMDTEMGKIQCGVT--EA-AS-------DEN-AHRTPLAIKLDEFGDTLTVVIG
Thalassiosira_oceanica_1        ------------------------------GTVITSGKGLGVVVRTGMSTEMGKIQKGVT--DA-AA-------DEN-AQRTPLGVKLDEFGDMLSYIIG
Phaeodactylum_tricornutum_1     ------------------------------GTMVTSGSGKAVVVQTGMTTQFGKIQQGVT--AA-KA-------EQ---PKTPLAIKLDEFGETLTIIIG
Fragilariopsis_cylindrus_1      ------------------------------GTTIVNGGGIAIVVATGSQTEFGKIQSGVM--DA-KK-------DNKTENKTPLTKQLDEFGNQLTKLIG
Fragilariopsis_cylindrus_2      ------------------------------GTTIVNGGGIAIVVATGSQTEFGKIQSGVM--DA-KK-------DNKTENKTPLTKQLDEFGNQLTKLIG
Fragilariopsis_cylindrus_3      ------------------------------GTTIVNGGGIAIVVATGSQTEFGKIQSGVM--DA-KK-------DNKTENKTPLTKQLDEFGNQLTKLIG
Fragilariopsis_cylindrus_4      ------------------------------GTTIVNGGGIAIVVATGSQTEFGKIQSGVM--DA-KK-------DNKTENKTPLTKQLDEFGNQLTKLIG
Fragilariopsis_cylindrus_5      ------------------------------GTTIVNGGGIAIVVATGSQTEFGKIQSGVM--DA-KK-------DNKTENKTPLTKQLDEFGNQLTKLIG
Fragilariopsis_cylindrus_6      ------------------------------GTTIVNGGGIAIVVATGSQTEFGKIQSGVM--DA-KK-------DNKTENKTPLTKQLDEFGNQLTKLIG
Fragilariopsis_cylindrus_7      ------------------------------GTTIVNGGGIAIVVATGSQTEFGKIQSGVM--DA-KK-------DNKTENKTPLTKQLDEFGNQLTKLIG
Fragilariopsis_cylindrus_8      ------------------------------GTTIVNGGGIAIVVATGSQTEFGKIQSGVM--DA-KK-------DNKTENKTPLTKQLDEFGNQLTKLIG
Pseudo-nitzschia_multiseries_1  ------------------------------GTTVVSGAAVALVVATGESTEFGRIQSGVL--EA-KR-------DSH-DQKTPLAQQLDDFGNQLTGLIG
Thalassiosira_pseudonana_2      ------------------------------GSLVTRGSGTALVVRTGTSTQMGKIQSTLA--EA-QS-------ETD-ERKTPLGEQLDQFGTTLSYVIG
Ostreococcus_lucimarinus_1      ------------------------------GTAVSNGCCLCVVNSTGMSTEIGKIQSQIK--EA-SE-------EE---EDTPLKQKLDRFGESLTMMIG
Zea_mays_1                      ------------------------------GTTVVNGSAVCVVTGTGMATEIGKIHAQIQ--EA-SQ-------EE---DDTPLKKKLNEFGEALTAIIG
Oryza_sativa_1                  ------------------------------GTTIVNGSAVCVVTGTGMDTEIGKIHAQIQ--EA-SQ-------EE---DDTPLKKKLNEFGEALTAIIG
Aquilegia_caerulea_1            ------------------------------GTTVVNGNCICLVIQTGMNTEIGSLHSQIQ--EA-SQ-------SE---EDTPLKKKLNEFGEMLTAIIG
A.thaliana_AT1G07810.1          ------------------------------GTTVVNGNCICLVTDTGMNTEIGRVHSQIQ--EA-AQ-------HE---EDTPLKKKLNEFGEVLTMIIG
A.thaliana_AT1G07670.1          ------------------------------GTTVVNGNCICLVTDTGMNTEIGRVHSQIQ--EA-AQ-------HE---EDTPLKKKLNEFGEVLTMIIG
Medicago_truncatula_1           ------------------------------GTTVVNGHCFCLVTQTGMDTEIGKVHNQIH--EA-SQ-------SE---EDTPLKKKLNEFGERLTMMIG
Glycine_max_1                   ------------------------------GTTVVNGNSICLVTQTGMDTEIGKVHMQIH--VA-SQ-------SE---EDTPLKKKLNEFGEKLTLIIG
Glycine_max_2                   ------------------------------GTTVVNGNCICLVTQTGMDTEIGKVHMQIH--VA-SQ-------SE---EDTPLKKKLNEFGEKLTMIIG
Aquilegia_caerulea_2            ------------------------------GTTVVNGNCICLVTQTGMNTEIGKVHSQIH--AA-AQ-------EE---EDTPLKKKLNEFGESLTIIIG
Selaginella_moellendorffii_1    ------------------------------GTTIVNGCCVGVVVSTGMFTEIGKIQAQIQ--EA-SM-------QD---DDTPLKKKLDEFGERLTVVIG
Physcomitella_patens_1          ------------------------------GTTIVNGCFLSVVITTGMKTEIGKIQSQIQ--EA-SL-------EE---ADTPLKKKLDEFGELLTKVIG
Physcomitella_patens_2          ------------------------------GTTIVNGSFLSMVMTTGMKTEIGKIQSQIQ--EA-SK-------EE---ADTPLKKKLDEFGELLTKVIG
Physcomitella_patens_3          ------------------------------GTVVVNGCFVSVVVSTGMNTEIGKIQSQIA--EA-SL-------EE---ADTPLKKKLDEFGERLTKVIG
Selaginella_moellendorffii_2    ------------------------------GTTVVNGSCFCIVTSTGMNTEIGKIQAQIQ--EA-AL-------EE---DDTPLKKKLDEFGERLTFVIG
Glycine_max_3                   ------------------------------GTTVVNGSCVCIVITTGMDTEIGKIHKQIH--EA-SQ-------EE---SDTPLRKKLDEFGNRLTTAIG
Glycine_max_4                   ------------------------------GTTVVNGSCVCIVITTGMDTEIGKIQKQIH--EA-SQ-------EE---SDTPLKKKLDEFGNRLTTAIG
Aquilegia_caerulea_3            ------------------------------GTTIMNGSCICVVVSTGMSTEIGKIQTQIH--EA-SL-------EE---TDTPLKKKLDEFGGRLTTAIG
A.thaliana_AT4G00900.1          ------------------------------GTTVVNGSCVCIVTSIGMDTEIGKIQRQIH--EA-SL-------EE---SETPLKKKLDEFGSRLTTAIC
Chlamydomonas_reinhardtii_1     ------------------------------GTAIANGSCSAVVTSIGMGTEIGKIQEQIS--AA-AK-------ED---DDTPLKKKLDEFGEMLAKVIA
Volvox_carteri_1                ------------------------------GTAIANGSCTAVVTSIGMATEIGKIQAQIS--EA-AK-------ED---DDTPLKKKLNEFGEMLAKVIA
Micromonas_pusilla_1            ------------------------------GTAASQGACVAIVTHTGMRTEIGKIQAQIQ--AA-SE-------EE---EDTPLKQKLDRFGDQLTWGIG
Micromonas_sp_1                 ------------------------------GTSVSQGACVAAVIDTGMRTEIGKIQAQIQ--AA-AE-------EE---DDTPLKQKLDTFGDQLTLMIG
Selaginella_moellendorffii_3    ------------------------------GTTVTNGSCICIVTDTGMATEIGKIQTQIQ--DA-SL-------SD---YDSPLTRKLDEFADVLTKVVA
Thalassiosira_oceanica_2        ------------------------------TTTVVGGIARGIVTDIGMATEIGKIQQAVQ--SA-GE-------DE---EDTPLKKKLNEFGDMLSQVIG
Thalassiosira_pseudonana_3      ------------------------------TTTVVGGIARGIVTDIGMKTEIGKIQLAVQ--GA-AE-------DE---EDTPLKKKLDEFGDMLSQVIG
Pseudo-nitzschia_multiseries_2  ------------------------------GTTISGGKAIGVVTATGMATEIGKIQSAVT--EA-SE-------DA---EKTPLNQKLDEFGDLLTQVIT
Fragilariopsis_cylindrus_9      ------------------------------GTTISGGKAIGIVCSIGMKTEIGKIQSAVS--EA-SE-------EQ---EKTPLTQKLDEFGDLLTKVIT
Fragilariopsis_cylindrus_10     ------------------------------GTTISGGKAIGIVCSIGMKTEIGKIQSAVS--EA-SE-------EQ---EKTPLTQKLDEFGDLLTKVIT
Aquilegia_caerulea_4            ------------------------------GTVVVAGRARAVVVGVGSSTAMGGIRDAML--RT-TD------------EVTPLKKKLDEFGTFLAKVIA
Glycine_max_5                   ------------------------------GTVMVAGRARAVVVGVGPNTAMGSIRDSML--RT-ED------------EVTPLKKKLDEFGTFLAKVIA
Medicago_truncatula_2           ------------------------------GTVVVAGRARAVVVGVGPNTAMGSIRDSML--QT-ED------------EVTPLKKKLDEFGTFLAKVIA
A.thaliana_AT1G10130.1          ------------------------------GTDVVAGRGRAVVIGVGSNTAMGSIHDSML--QT-DD------------EATPLKKKLDEFGSFLAKVIA
Oryza_sativa_2                  LHLNEYGHFLLSIRFIAVILYLLSAFFASKGTVVVAGRARAVVIGVGSNTAMGSIRDAML--RT-ED------------EATPLKKKLDEFGTFLAK---
Selaginella_moellendorffii_4    ------------------------------GTNVTAGRARAIVVATGSETAMGKIHSAMS--EV-TE------------EMTPLKKQLDNFGEFLSKVIA
Micromonas_pusilla_2            ------------------------------GTVVTVGRCRGVVVGTGLNTAIGKIRDAMT--EA-AA-------EE---EMTPLKKKLDEFGTLLSKVIA
Micromonas_sp_2                 ------------------------------GTIVTAGRAQGVVVATGMSTAIGQIQNAVT--EV-DC-------MD---ETTPLKRKLDEFGTFLSKVIA
Volvox_carteri_2                ------------------------------GTLVTSGRARGVVVGTGASTAIGRIRDALA--SA-DE--------D---QRTPLKQKLDEFGTLLSKVIA
Chlamydomonas_reinhardtii_2     ------------------------------GTLVTSGRGRGIVVGTGASTAIGRIRDALS--AA-DG-------AD---EKTPLKQKLDEFGELLSKVIA
Dunaliella_salina_1             ------------------------------GTLVVAGRARGIVVGTGSSTAIGKIRDAMS--VE-ED------------VVTPLKAKLDEFGALLSKVIA
Ostreococcus_lucimarinus_2      ------------------------------GTTVTVGKATCVVVGTGSNTAMGKIQHTLE--QT-EE------------ELTPLKKKLDEFGNLLGKIIA
Phaeodactylum_tricornutum_2     ------------------------------GSLVNAGRGRALVIAVGVSTEFGKVATELS--SV-AS------------RKSPLQIKIDELGQRLAGLSS
Chlamydomonas_reinhardtii_3     ------------------------------ATNVVSGQGRGVVVATGDSAEIGQINKMVG--TV-ES------------ARNNLVHQLEVLGRWLVLLVL
Chlamydomonas_reinhardtii_4     ------------------------------ATNVVSGQGRGVVVATGDSAEIGQINKMVG--TV-ES------------ARNNLVHQLEVLGRWLVLLVL
Thalassiosira_pseudonana_4      ------------------------------ATLVAQGSGIGIAISTGDFTQIGTINRLVN--NT-ET------------IKTDVLKQIDMVSKYLFIAIC
Thalassiosira_pseudonana_5      ------------------------------ATLVAQGSGIGVAIATGDFTQIGTINLLVN--NT-QS------------IETDVLKQIDQISKYLFVAIC
```

```
Fragilariopsis_cylindrus_11        ------------------------------ATLIATGSGVGVATSTGDNTEIGTINKLVN--NV-EK------------KKTAVLEQIDTISIYLAVFIL
Fragilariopsis_cylindrus_12        ------------------------------ATLIATGSGVGVATSTGDNTEIGTINKLVN--NV-EK------------KKTAVLEQIDTISIYLAVFIL
Pseudo-nitzschia_multiseries_3     ------------------------------ATLIAQGSGVGIAVATGDNTEIGTINKLVN--SQ-EK------------TKTAVLEQIDTISLYLAGFIM
Phaeodactylum_tricornutum_3        ------------------------------ATLVAQGYGVGVVVCTGDNTEIGTINSLVN--KV-ET------------KRTAVLEQIDFVSKVLACFIF
Micromonas_pusilla_3               ------------------------------SCNVKAGACLGLVVATGMRTRKKK-EPCLP--DT-KS------------GQSPLQENLEKLAVKLGYMAI
Micromonas_sp_3                    ------------------------------SCNVKAGTCIGLVVATGMRTRAKK-EGCLP--DT-KA------------GQTPLQANLEALSIKLGYMAI
Chlamydomonas_reinhardtii_5        ------------------------------GTTVNEGSGHMLVLAVGVHSEWGKTMALVN--EA-GD------------DETPLQEQLTDVAAKVSKMGV
Chlamydomonas_reinhardtii_6        ------------------------------GTTVNEGSGHMLVVAVGVNSEWGKTMALVS--EA-GD------------DETPLQEQLTDVAAKVSKMGV
Volvox_carteri_3                   ------------------------------GTTVNEGSGHVLIVAVGPHSEWGKTMALVS--EA-GD------------DQTPLQEQLTDVAAKVSKMGV
Volvox_carteri_4                   ------------------------------GTTVNEGSGHVLIVAVGPHSEWGKTMALVS--EA-GD------------DQTPLQEQLTDVAAKVSKMGV
Volvox_carteri_5                   ------------------------------GTQVTEGSGHVLVIAVGPNSTWGKTMALVT--EA-GD------------DETPLQEKLGVLATAIGKIGF
Chlamydomonas_reinhardtii_7        ------------------------------GTQVTEGSGRVLVTAVGPNSTWGKTMALVS--EA-GD------------DETPLQQKLEVLAGAIGKVGF
Chlamydomonas_reinhardtii_8        ------------------------------GTQVTEGSGKLLVVAVGENSEWGKTMALVG--EA-GD------------DETPLQVKLTWVASTVGKIGF
Volvox_carteri_6                   ------------------------------GTQVTEGSGKLLIVAVGENSEWGKTMALVG--EA-GD------------DETPLQVKLTWVASTVGKVGF
Ostreococcus_lucimarinus_3         ------------------------------GTSISEGSGKALIIAVGSRSQWGVILKTLIV-EP---------------SDTPLQERLERLVLLIGNFGI
Micromonas_sp_4                    ------------------------------GTTISEGSGRMVVIAVGSSSQWGVILKTLIV-EP---------------SSTPLQDRLDVLVVTVGNFGI
Selaginella_moellendorffii_5       ------------------------------GCKVVDGYGTILITSVGINTEWGRAMAALT--DDISD------------EETPLQMRLAGAATVIGAIGL
A.thaliana_AT3G21180.1             ------------------------------GCKVADGVGNMLVTGVGINTEWGLLMASIS--ED-TG------------EETPLQVRLNGLATFIGIVGL
Aquilegia_caerulea_5               ------------------------------GCKVADGYGTMLVTGVGINTEWGLLMASIS--ED-TG------------EETPLQVRLNGVATFIGIVGL
A.thaliana_AT5G57110.2             ------------------------------GCKVADGNGSMLVTGVGVNTEWGLLMASIS--ED-NG------------EETPLQVRLNGVATFIGSIGL
Oryza_sativa_3                     ------------------------------GCKVADGYGTMLVTAVGINTEWGLLMASIS--ED-SG------------EETPLQVRLNGVATFIGMVGL
Zea_mays_2                         ------------------------------GCKVADGYGTMLVTAVGINTEWGLLMASIS--ED-SG------------EETPLQVRLNGVATFIGMVGL
Zea_mays_3                         ------------------------------GCKVADGYGSMLVTGVGINTEWGMLMANLS--ED-IG------------EETPLQVRLNGVATLIGIVGL
Zea_mays_4                         ------------------------------GCKVADGYGSMLVTGVGTNTEWGQLMANLS--ED-NG------------EETPLQVRLNGVATFIGLVGL
Selaginella_moellendorffii_6       ------------------------------GCKVVDGYGSMLITGVGINTEWGQVMATLD--DD-SS------------EETPLQVRLNGIATFVGKIGL
Selaginella_moellendorffii_7       ------------------------------GTKVLDGFGTMLVTAVGMRTEWGRVMATLS--ED-ND------------EETPLQVRLNNLATIIGKVGL
Aquilegia_caerulea_6               ------------------------------GTKVQDGSCKMLVTTVGMRTQWGKLLATLS--EG-GD------------DETPLQVKLNGVATIIGKIGL
Medicago_truncatula_3              ------------------------------GTKVQDGSCKMLVTTVGMRTQWGKLMATLS--EG-GD------------DETPLQVKLNGVATIIGKIGL
A.thaliana_AT2G22950.1             ------------------------------GTKVQDGSCKMLVTTVGMRTQWGKLMATLS--EG-GD------------DETPLQVKLNGVATIIGKIGL
A.thaliana_AT4G37640.1             ------------------------------GTKVQDGSCKMMITTVGMRTQWGKLMATLT--EG-GD------------DETPLQVKLNGVATIIGKIGL
Oryza_sativa_4                     ------------------------------GTKVQDGSCKMLVTTVGMRTQWGKLMATLS--EG-GD------------DETPLQVKLNGVATIIGKIGL
Zea_mays_5                         ------------------------------GTKVQDGACKMLVTTVGMRTQWGKLMATLS--EG-GD------------DETPLQVKLNGVATIIGKIGL
Oryza_sativa_5                     ------------------------------GTKVQDGSCKMLITTVGMRTQWGKLMATLS--EG-GD------------DETPLQVKLNGVATIIGKIGL
A.thaliana_AT1G27770.1             ------------------------------GTKVQDGSCKMLVTTVGMRTQWGKLMATLS--EG-GD------------DETPLQVKLNGVATIIGKIGL
Oryza_sativa_6                     ------------------------------GTKVLDGSCKMLVTAVGMRTQWGKLMAVLT--DG-GD------------DETPLQTRLNGVANTIGKIGL
Zea_mays_6                         ------------------------------GTKVLDGSCIMLVTAVGMRTQWGKLMAAIT--ES-GD------------DETPLQGKLNGVANTIGNIGL
Aquilegia_caerulea_7               ------------------------------GTKVQDGSGKMLIASVGMRTEWGRLMETLS--EG-GE------------DETPLQVKLNGVATIIGKIGL
Oryza_sativa_7                     ------------------------------GNKVVDGAAKMLVTAVGTRTEWGKIMGTLN--GD-GV------------DETPLQVKLNGVATIIGQIGL
Selaginella_moellendorffii_8       ------------------------------GTKVQDGSALMLVTGVGMNTEWGHLMAVLG--EG-GD------------DETPLQVRLNGVATLIGKIGL
Selaginella_moellendorffii_9       ------------------------------GTKIGDGSGVMIVTGVGMNTEWGHSMSILSGEDS-GQ------------SETPLQHKLQDLATLIGKIGL
Oryza_sativa_8                     ------------------------------GVKVVDGYGKMVVTAVGTDTAWGEMMRTIT--RE-NT------------DPTPLQERLEGLTSSIGKVGI
Fragilariopsis_cylindrus_13        ------------------------------GCTAVAGSGRFVAIAVGPNSQWGVIKSHLEQ----EQ------------EETPLQEKLDIMAANIGYVGM
Pseudo-nitzschia_multiseries_4     ------------------------------GCTVVAGSTRFVATAVGPNSQWGVIKSHLEK----EQ------------EETPLQEKLDDMAAKIGYVGM
Phaeodactylum_tricornutum_4        ------------------------------GCTMEAGTARFLAIAVGKDSQWGIIKAHLDK----EH------------SQTPLQEKLDDMAAMIGYIGM
Thalassiosira_pseudonana_6         ------------------------------GTTAVQGSAKMVVIAVGINSVAGKIKAHVY--DS-SDHEGEGLEGD---DESPLFTKLEKIAKQIGIAGT
Phaeodactylum_tricornutum_5        ------------------------------GTNVQQGSAKLVVIAVGIHSVAGKIRAQVY--ES-EDHRDDLGGDD---EESPLFVKLNVLAKRIGIAGT
Phaeodactylum_tricornutum_6        ------------------------------GTTASQGSGKMVVIAVGIHSVAGKIKARVY--ES-EDHEDELGDDG---EHSPLFFKINAIAKRVGIAGT
```

```
                                 501
Skeletonema_marinoi_ABC          LICVGVWVASIPKF---------------NDPTFKQ-----------PIEGAIYYAKVAVALGVAAI-QRLPAVITLCLSLGTRRMAKRNVIVRKLPSV
Thalassiosira_pseudonana_1       VICTAVWVASIPKF---------------YDPTFKT-----------PIEGAVYYAKVAVALGVAALPEGLPAVITLCLSLGTRRMAKRNVIVRKLQSV
Thalassiosira_oceanica_1         GICIAVWVASIPRF---------------HDPMFKS-----------PVEGAVYYAKVAVALGVAAIPEGLPAVITLCLSLGTRRMAKRNVIVRKLPSV
Phaeodactylum_tricornutum_1      VICLAVWIVSIPKM---------------NDPSFGS-----------VWVGAVYYAKVAVALGVAAIPEGLPAVITLCLSLGTRRMAERNVIVRKLQSV
Fragilariopsis_cylindrus_1       LICGLVWIASIPKMLT--------------SNIFNS-----------KLEGIIYYTKVAVALGVAAIPEGLPAVITLCLSLGTRRMAQRNVIVRHLPSV
Fragilariopsis_cylindrus_2       LICGLVWIASIPKMLT--------------SNIFNS-----------KLEGIIYYTKVAVALGVAAIPEGLPAVITLCLSLGTRRMAQRNVIVRHLPSV
Fragilariopsis_cylindrus_3       LICGLVWIASIPKMLT--------------SNIFNS-----------KLEGIIYYTKVAVALGVAAIPEGLPAVITLCLSLGTRRMAQRNVIVRHLPSV
Fragilariopsis_cylindrus_4       LICGLVWIASIPKMLT--------------SNIFNS-----------KLEGIIYYTKVAVALGVAAIPEGLPAVITLCLSLGTRRMAQRNVIVRHLPSV
Fragilariopsis_cylindrus_5       LICGLVWIASIPKMLT--------------SNIFNS-----------KLEGIIYYTKVAVALGVAAIPEGLPAVITLCLSLGTRRMAQRNVIVRHLPSV
Fragilariopsis_cylindrus_6       LICGLVWIASIPKMLT--------------SNIFNS-----------KLEGIIYYTKVAVALGVAAIPEGLPAVITLCLSLGTRRMAQRNVIVRHLPSV
Fragilariopsis_cylindrus_7       LICGLVWIASIPKMLT--------------SNIFNS-----------KLEGIIYYTKVAVALGVAAIPEGLPAVITLCLSLGTRRMAQRNVIVRHLPSV
Fragilariopsis_cylindrus_8       LICGLVWIASIPKMLT--------------SNIFNS-----------KLEGIIYYTKVAVALGVAAIPEGLPAVITLCLSLGTRRMAQRNVIVRHLPSV
Pseudo-nitzschia_multiseries_1   VICAAVWFASIPKMMA------------KDSTVFSS-----------PFEGMVYYAKVAVALGVAAIPEGLPAVITLCLSLGTRRMAQRNVIVRNLPSV
Thalassiosira_pseudonana_2       GICLAVWIASVPRF---------------SDSAFST-----------WLEGAIYYAKVGVALGVAAIPEGLPAVITLCLSLGTRRMAERNVIVRKLPSV
Ostreococcus_lucimarinus_1       VICLLVWLINYRHFVQ-------------FEFKAGS----TIPSVAFDLVKCTYYFKIAIALAVAAIPEGLPAVITTCLALGTRKMAKKNAIVRKLPSV
Zea_mays_1                       VICALVWLINLKYFLS-------------WEYVDG----W-PTNFKFSFEKCTYYFEIAVALAVAAIPEGLPAVITTCLALGTRKMAQKNALVRKLPSV
Oryza_sativa_1                   VICALVWLINVKYFLT-------------WEYVDG----W-PRNFKFSFEKCTYYFEIAVALAVAAIPEGLPAVITTCLALGTRKMAQKNALVRKLPSV
Aquilegia_caerulea_1             VICALVWLINVKYFLT-------------WEYVDG----W-PTNFHFSFEKCTYYFEIAVALAVAAIPEGLPAVITTCLALGTRKMAQKNALVRKLPSV
A.thaliana_AT1G07810.1           LICALVWLINVKYFLS-------------WEYVDG----W-PRNFKFSFEKCTYYFEIAVALAVAAIPEGLPAVITTCLALGTRKMAQKNALVRKLPSV
A.thaliana_AT1G07670.1           LICALVWLINVKYFLS-------------WEYVDG----W-PRNFKFSFEKCTYYFEIAVALAVAAIPEGLPAVITTCLALGTRKMAQKNALVRKLPSV
Medicago_truncatula_1            LICILVWLINVKYFLT-------------WDYVDDG--WW-PTNFKFSFEKCTYYFEIAVALAVAAIPEGLPAVITTCLALGTRKMAQKNALVRKLPSV
Glycine_max_1                    LICILVWLINVKYFLS-------------WEYVDG----W-PRNFKFSFEKCTYYFEIAVALAVAAIPEGLPAVITTCLALGTRKMAQKNALVRKLPSV
Glycine_max_2                    LICILVWLINVKYFLS-------------WEYVDG----W-PRNFKFSFEKCTYYFEIAVALAVAAIPEGLPAVITTCLALGTRKMAQKNALVRKLPSV
Aquilegia_caerulea_2             IICALVWLINVKYFLT-------------WEYVDG----W-PANFKFSFEKCTYYFEIAVALAVAAIPEGLPAVITTCLALGTRKMAQKNALVRKLPSV
Selaginella_moellendorffii_1     VICLLVWIINYKYFLT-------------WDYADG----W-PTNVRFSFEKCTYYFEIAVALAVAAIPEGLPAVITTCLALGTRKMAQKNAIVRKLPSV
Physcomitella_patens_1           VICLLVWIINYKYFLS-------------WEMKDG----W-PTNFQFSFEKCTYYFKIAVALAVAAIPEGLPAVITTCLALGTRKMAAKNAIVRKLPSV
Physcomitella_patens_2           VICLLVWVINYKYFLS-------------WEMKDG----W-PRNFQFSFEKCTYYFKIAVALAVAAIPEGLPAVITTCLALGTRKMAAKNAIVRKLPSV
Physcomitella_patens_3           VICVLVWMINYEHFLS-------------WDMKNG----F-PTNIRFSFEKCTYYFKIAVALAVAAIPEGLPAVITTCLALGTKKMAAKNAIVRKLPSV
Selaginella_moellendorffii_2     MICLLVWVINYKYFLS-------------WDVVGG----W-PRNVRFSFEKCTYYFEIAVALAVAAIPEGLPAVITTSLALGTRKMAQKNAIVRKLPSV
Glycine_max_3                    LVCLIVWVINYKNFIS-------------WEVVDG----W-PSNINFSFQKCTYYFKIAVSLAVAAIPEGLPAVITTCLALGTRKMAQKNAIVRKLPSV
Glycine_max_4                    LVCLIVWVINYKNFIS-------------WDVVDG----W-PSNIKFSFQKCTYYFKIAVALAVAAIPEGLPAVITTCLALGTRKMAQKNAIVRKLPSV
Aquilegia_caerulea_3             IVCLIVWVINYKNFLT-------------WDVVNG----W-PTNFRFSFEKCTYYFKIAVALAVAAIPEGLPAVITTCLALGTRKMAKKNAIVRKLPSV
A.thaliana_AT4G00900.1           IVCVLVWMINYKNFVS-------------WDVVDG----YKPVNIKFSFEKCTYYFKIAVALAVAAIPEGLPAVITTCLALGTRKMAQKNAIVRKLPSV
Chlamydomonas_reinhardtii_1      AICVVVWLINYEHFVT-------------FTWKPEG----GLPGVAFNLSKATYYFKIAVALAVAAIPEGLPAVITTCLALGTRTMAKKNAIVRKLPSV
Volvox_carteri_1                 AICVIVWLINYHHFLT-------------ITFKPEG----GLPTFSFNLSKATYYFKIAVALAVAAIPEGLPAVITTCLALGTRTMAKKNAIVRKLPSV
Micromonas_pusilla_1             LVCLFVWLMNYQFFIS-------------WKRAPGS---FVPYDVEFNFAKCTFYFKIAVALAVAAIPEGLPAVITTCLALGTRKMAKKNAIVRKLQSV
Micromonas_sp_1                  VICLLVWLMNYHFFIS-------------WKWGGLSDP-FSITEVDFNFAKCTFYFKIAVALAVAAIPEGLPAVITTCLALGTRKMAKKNAIVRKLQSV
Selaginella_moellendorffii_3     AICGIVWVVNYKYFLT-------------WDVSNGL-----PSNVQFDVGQATYYFKVAVALAVAAIPEGLPAVITTCLALGTRRMAEENAIVRKLPSV
Thalassiosira_oceanica_2         VICILVWAINYSHF---------------FDPVHGS-----------VFKGCIYYFKIAVALAVAAIPEGLPTVITTCLALGTRKMAAKNAIVRKLPSV
Thalassiosira_pseudonana_3       VICLLVWAINYNHF---------------FDPVHGS-----------VFNGCIYYFKIAVALAVAAIPEGLPTVITTCLALGTRKMAAKNAIVRKLPSV
Pseudo-nitzschia_multiseries_2   GICVLVWVMNFNQF---------------SDPVFGG-----------VFKGCIYYLKIAVALGVAAIPEGLPAVITLCLALGTKKMVKRNAIVRKLPSV
Fragilariopsis_cylindrus_9       AICVLVWVMNYNKF---------------FDPVFGG-----------IFKGCIYYLKIAVALGVAAIPEGLPAVITLCLALGTKKMVKRNAIVRRLPSV
Fragilariopsis_cylindrus_10      AICVLVWVMNYNKF---------------FDPVFGG-----------IFKGCIYYLKIAVALGVAAIPEGLPAVITLCLALGTKKMVKRNAIVRRLPSV
Aquilegia_caerulea_4             GICVLVWIVNIGHF---------------RDPAHGG-----------FLRGAIHYFKIAVALAVAAIPEGLPAVVTTCLALGTKRMARLNAIVRSLPSV
Glycine_max_5                    GICVLVWIVNIGHF---------------RDPSHGG-----------FLRGAIHYFKIAVALAVAAIPEGLPAVVTTCLALGTKRMAKLNAIVRSLPSV
Medicago_truncatula_2            GICVLVWIVNIGHF---------------RDPSHGG-----------FVHGAIHYFKIAVALAVAAIPEGLPAVVTTCLALGTKRMARLNAIVRSLPSV
A.thaliana_AT1G10130.1           GICVLVWVVNIGHF---------------SDPSHGG-----------FFKGAIHYFKIAVALAVAAIPEGLPAVVTTCLALGTKKMARLNAIVRSLPSV
Oryza_sativa_2                   --------------------------------KHRF----------LKLHRTLFSFQVAVALAVAAIPEGLPAVVTTCLALGTKRMARLNAIVRSLPSV
Selaginella_moellendorffii_4     GICVLVWVVNIGHF---------------RDPAHGG-----------ILRGAIYYLKIAVALAVAAIPEGLPAVVTTCLALGTKRMASLNAIVRSLPAV
Micromonas_pusilla_2             VVCVLVWVVNIGHF---------------ADKAHGG-----------MLRGAIYYFKIAVALAVAAIPEGLPAVVTTCLALGTRKMAKQNAIVRSLPSV
Micromonas_sp_2                  AICILVWLINIRHF---------------RDYAHGG-----------IFRGAIHYFKVAVALAVAAIPEGLPAVVTTCLALGTRKLAKQKAIVRTLSSV
Volvox_carteri_2                 AICVIVWLMNIRRF---------------SDPALGG-----------WLSGALYYLKIAVALAVAAIPEGLPAVVTTCLALGTRKMAKQNAIVRSLPSV
Chlamydomonas_reinhardtii_2      TICVIVWLMNINRF---------------KDPALGG-----------WLSGALYYLKIAVALAVAAIPEGLPAVVTTCLALGTRKMAKQNAIVRSLPSV
Dunaliella_salina_1              GICVLVWVVNINRF---------------NDPALGG-----------WFQGAIHYFKIAVALAVAAIPEGLPAVVTTCLALGTRKMARHNAIVRTLPSV
Ostreococcus_lucimarinus_2       VICILVWVVNIGHF---------------ADKAHGG-----------LLRGAVYYFKIAVALAVAAIPEGLPAVVTTCLALGTRRMAKKNALVRTLPSV
Phaeodactylum_tricornutum_2      IAISIIAMLGWI-----------------------------------LGRPFLETLTVAVSLAVAAIPEGLPICVTVTLALGVLRMARRNAIIKKLPVV
Chlamydomonas_reinhardtii_3      AIGLLAFLLALLHA----------------------------------DQGFKEAFESAVSIAVAIVPEGLPAMVTIVLAIGTTVMARNNAIIRQLPAV
Chlamydomonas_reinhardtii_4      AIGLVAFLLALLHA----------------------------------DQGFKEAFESAVSIAVAIVPEGLPAVVTIVLAIGTTVMARNNAIIRQLPAV
Thalassiosira_pseudonana_4       TMCLATFFVAFFYSDN-------------------------------YKKDALGSINIALTCAVAMVPEGLEAIVTLTYSYAVKVMASQNAIVRALPAV
Thalassiosira_pseudonana_5       TMCLGTFFVAFYYSDS-------------------------------YYKNALSSVNIALTCAVAMVPEGLEAIVTLVYSYAVKVMATQNAIVRALPAV
```

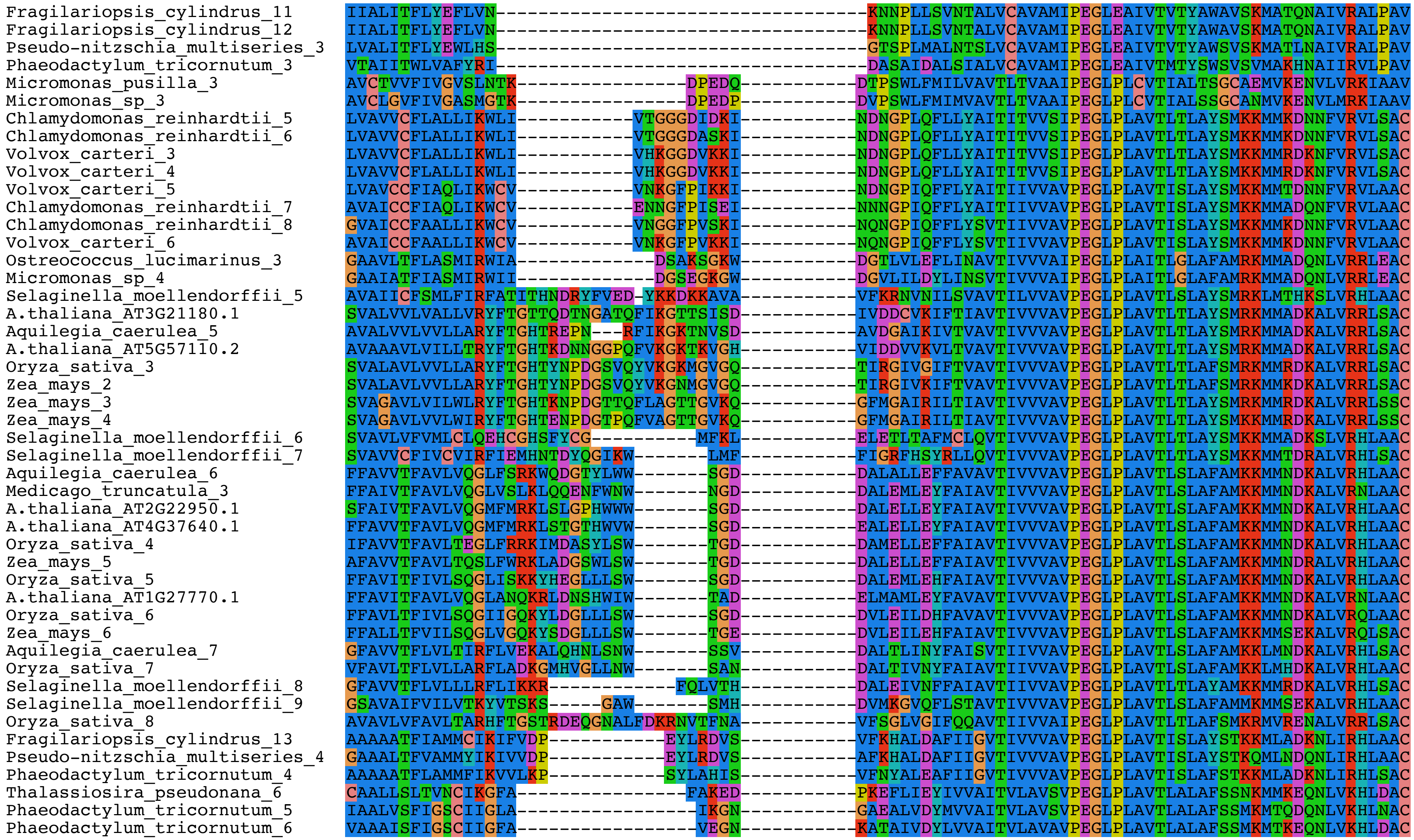
Fragilariopsis_cylindrus_11
Fragilariopsis_cylindrus_12
Pseudo-nitzschia_multiseries_3
Phaeodactylum_tricornutum_3
Micromonas_pusilla_3
Micromonas_sp_3
Chlamydomonas_reinhardtii_5
Chlamydomonas_reinhardtii_6
Volvox_carteri_3
Volvox_carteri_4
Volvox_carteri_5
Chlamydomonas_reinhardtii_7
Chlamydomonas_reinhardtii_8
Volvox_carteri_6
Ostreococcus_lucimarinus_3
Micromonas_sp_4
Selaginella_moellendorffii_5
A.thaliana_AT3G21180.1
Aquilegia_caerulea_5
A.thaliana_AT5G57110.2
Oryza_sativa_3
Zea_mays_2
Zea_mays_3
Zea_mays_4
Selaginella_moellendorffii_6
Selaginella_moellendorffii_7
Aquilegia_caerulea_6
Medicago_truncatula_3
A.thaliana_AT2G22950.1
A.thaliana_AT4G37640.1
Oryza_sativa_4
Zea_mays_5
Oryza_sativa_5
A.thaliana_AT1G27770.1
Oryza_sativa_6
Zea_mays_6
Aquilegia_caerulea_7
Oryza_sativa_7
Selaginella_moellendorffii_8
Selaginella_moellendorffii_9
Oryza_sativa_8
Fragilariopsis_cylindrus_13
Pseudo-nitzschia_multiseries_4
Phaeodactylum_tricornutum_4
Thalassiosira_pseudonana_6
Phaeodactylum_tricornutum_5
Phaeodactylum_tricornutum_6

```
601
Skeletonema_marinoi_ABC ETLGCTSVICTDKTGTLTTNEMTAVSLVLL-EDN-----------------------SIVEEHAISGVSYS----------P-----------EGTIDGI
Thalassiosira_pseudonana_1 ETLGCTSVICTDKTGTLTTNEMTAVSLVLL-ESDEEGG-------------------VLVAEHEVSGTSYS----------P-----------IGTIKGV
Thalassiosira_oceanica_1 ETLGCTSVICTDKTGTLTTNEMTAVSLVMI-EN------------------------YVVEEHSISGVSYS----------P-----------VGTVDGV
Phaeodactylum_tricornutum_1 ETLGCTSVICTDKTGTLTTNEMTVVSLVLL-EHDEVGE-------------------VSIRERIVEGFSYS----------P-----------VGEVEGI
Fragilariopsis_cylindrus_1 ETLGCVSVICSDKTGTLTTNEMTVISLLLF-QKNQDQKQKQNKRSKKNDKNNKNDSLSCLTEHTIDGVSYS----------P-----------IGKINDL
Fragilariopsis_cylindrus_2 ETLGCVSVICSDKTGTLTTNEMTVISLLLF-QKNQDQKQKQNKRSKKNDKNNKNDSLSCLTEHTIDGVSYS----------P-----------IGKINDL
Fragilariopsis_cylindrus_3 ETLGCVSVICSDKTGTLTTNEMTVISLLLF-QKNQDQKQKQNKRSKKNDKNNKNDSLSCLTEHTIDGVSYS----------P-----------IGKINDL
Fragilariopsis_cylindrus_4 ETLGCVSVICSDKTGTLTTNEMTVISLLLF-QKNQDQKQKQNKRSKKNDKNNKNDSLSCLTEHTIDGVSYS----------P-----------IGKINDL
Fragilariopsis_cylindrus_5 ETLGCVSVICSDKTGTLTTNEMTVISLLLF-QKNQDQKQKQNKRSKKNDKNNKNDSLSCLTEHTIDGVSYS----------P-----------IGKINDL
Fragilariopsis_cylindrus_6 ETLGCVSVICSDKTGTLTTNEMTVISLLLF-QKNQDQKQKQNKRSKKNDKNNKNDSLSCLTEHTIDGVSYS----------P-----------IGKINDL
Fragilariopsis_cylindrus_7 ETLGCVSVICSDKTGTLTTNEMTVISLLLF-QKNQDQKQKQNKRSKKNDKNNKNDSLSCLTEHTIDGVSYS----------P-----------IGKINDL
Fragilariopsis_cylindrus_8 ETLGCVSVICSDKTGTLTTNEMT------------------------------NDSLSCLTEHTIDGVSYS----------P-----------IGKINDL
Pseudo-nitzschia_multiseries_1 ETLGCVSVICSDKTGTLTTNEMTVKSLVLL-EKSKT---------------------NPIVEHSVEGVSYS----------P-----------YGSIGGI
Thalassiosira_pseudonana_2 ETLGCTSVICTDKTGTLTSNQMTSVSLVLL-ETTENG--------------------IELVEHEITGSSYN----------P-----------FGSAVGI
Ostreococcus_lucimarinus_1 ETLGCTSVICSDKTGTLTTNQMSAVKLITV-RDET-----------------------HLTTYTVEGTTYN----------PT----------EGGVVG-
Zea_mays_1 ETLGCTTVICSDKTGTLTTNQMSAVKLVAI-GRWP----------------------DTLRSFKVDGTTYD----------PT----------DGKIHD-
Oryza_sativa_1 ETLGCTTVICSDKTGTLTTNQMSAVKLVAI-GRWP----------------------DTLRSFKVDGTTYD----------PS----------DGKINE-
Aquilegia_caerulea_1 ETLGCTTVICSDKTGTLTTNQMAVSKLVAL-GTRK----------------------DMIRTFRVDGTTYS----------PG----------DGKIHN-
A.thaliana_AT1G07810.1 ETLGCTTVICSDKTGTLTTNQMAVSKLVAM-GSRI----------------------GTLRSFNVEGTSFD----------PR----------DGKIED-
A.thaliana_AT1G07670.1 ETLGCTTVICSDKTGTLTTNQMAVSKLVAM-GSRI----------------------GTLRSFNVEGTSFD----------PR----------DGKIED-
Medicago_truncatula_1 ETLGCTTVICSDKTGTLTTNQMAVSKLVAI-GTNV----------------------DALRAFKVEGTTYN----------PN----------DGQIEN-
Glycine_max_1 ETLGCTTVICSDKTGTLTTNQMAVAKLVAV-GHNV----------------------DTLRAFKVEGTTYN----------PA----------DGQIEN-
Glycine_max_2 ETLGCTTVICSDKTGTLTTNQMAVAKLVAI-GHNV----------------------DTLRAFKVEGTTYN----------PA----------DGQIEN-
Aquilegia_caerulea_2 ETLGCTTVICSDKTGTLTTNQMAVAKLVAM-GPST----------------------NAVRSFKVDGTTYS----------PI----------DGKIHN-
Selaginella_moellendorffii_1 ETLGCTTVICSDKTGTLTTNQMSVMQLVGS-GIHP----------------------ADMEEFRVEGTTYN----------PE----------DGGITG-
Physcomitella_patens_1 ETLGCTTVICSDKTGTLTTNQMSVTDLVLN-GPAA----------------------GVTRQFHVEGTTYS----------FL----------DGKIAG-
Physcomitella_patens_2 ETLGCTTVICSDKTGTLTTNQMSVTELILN-GPAA----------------------GVTRDFHVEGTTYS----------FL----------DGKIEG-
Physcomitella_patens_3 ETLGCTTVICSDKTGTLTTNQMSVTELVLN-GAEP----------------------GVTRNFHVGGTSYD----------FQ----------DGVIES-
Selaginella_moellendorffii_2 ETLGCTTVICSDKTGTLTTNQMSVMEFAGN-GGSA----------------------GEVREFSVEGTTYN----------PD----------DGEILG-
Glycine_max_3 ETLGCTTVICSDKTGTLTTNQMAVTEFFTL-GGKT----------------------TASRLISVEGTTYD----------PK----------DGGILD-
Glycine_max_4 ETLGCTTVICSDKTGTLTTNQMAVTEFFTL-GGKT----------------------TASRLIGVEGTTYD----------PK----------DGGIVD-
Aquilegia_caerulea_3 ETLGCTTVICSDKTGTLTTNQMSVSQFFTL-GGKT----------------------TISRIFNVEGTTYD----------PK----------DGGILD-
A.thaliana_AT4G00900.1 ETLGCTTVICSDKTGTLTTNQMSATEFFTL-GGKT----------------------TTTRVFSVSGTTYD----------PK----------DGGIVD-
Chlamydomonas_reinhardtii_1 ETLGCTTVICSDKTGTLTTNQMSAVALAAM-GSDG----------------------ASVRRWAVAGHTYC----------PD----------DGEVVG-
Volvox_carteri_1 ETLGCTTVICSDKTGTLTTNQMSAVALTAFEGPNG----------------------TSMRRWVVAGHTYN----------PD----------EGEVEG-
Micromonas_pusilla_1 ETLGCTSVICSDKTGTLTTNNMSAVKLVVP-TIKP----------------------DVLKTYDVTGTSYD----------AS----------DGAVVGA
Micromonas_sp_1 ETLGCTSVICSDKTGTLTTNNMSAVRLV------------------------------------VQGTSYD----------PS----------DGGVVG-
Selaginella_moellendorffii_3 ETLGCTTVICSDKTGTLTTNQMSVVQLLAV-EGP-----------------------DELRTFRVTGTSYD----------PD----------DGHVIG-
Thalassiosira_oceanica_2 ETLGCTNVICSDKTGTLTTNEMSCVEIVLP-GSKA-----------------------EMSAHAVSGITYA----------PV-----------GTIEPA
Thalassiosira_pseudonana_3 ETLGCTNVICSDKTGTLTTNEMSCVEVVLP-EGKN-----------------------EMTTRVVSGITYA----------PT-----------GEISPP
Pseudo-nitzschia_multiseries_2 ETLGCCTVICSDKTGTLTTNQMVVEALVVP-DAEE----------------------GAIEEYRVTGTEYA----------PT----------DGEIQNY
Fragilariopsis_cylindrus_9 ETLGCCTVICSDKTGTLTTNQMVVESI--D-DYSG----------------------NNIREYHITGTEYS----------PT----------DGAIQDY
Fragilariopsis_cylindrus_10 ETLGCCTVICSDKTGTLTTNQMVVESI--D-DYSG----------------------NNIREYHITGTEYS----------PT----------DGAIQDY
Aquilegia_caerulea_4 ETLGCTTVICSDKTGTLTTNMMSVSKICVI-SSVNHG--------------------AITAEYRVSGTTYA----------P-----------EGIILDG
Glycine_max_5 ETLGCTTVICSDKTGTLTTNMMSVAKVCVV-ESAKRG--------------------PVVSEYSVSGTTYA----------P-----------EGIIFDS
Medicago_truncatula_2 ETLGCTTVICSDKTGTLTTNMMSVAKICIV-ESSKSS--------------------PFVTEYGVSGTTYA----------P-----------EGIIFDK
A.thaliana_AT1G10130.1 ETLGCTTVICSDKTGTLTTNMMSVSKICVV-QSAEHG--------------------PMINEFTVSGTTYA----------P-----------EGTVFDS
Oryza_sativa_2 ETLGCTTVICSDKTGTLTTNMMSVSKVCVV-RSVHQR--------------------PITDEYSISGTTFA----------P-----------DGFIYDA
Selaginella_moellendorffii_4 ETLGCTTVICSDKTGTLTTNMMSVTKICVV-ESA-----------------------ATLSEFTVTGTSYA----------P-----------DGVIQNA
Micromonas_pusilla_2 ETLGCTSVICSDKTGTLTTNAMLATRVCVV-DASEGAAGAAAARVG----------DACLAEYEVTGDGYS----------P-----------DGVVTEA
Micromonas_sp_2 ETLGCTSVICSDKTGTVTTNIMTITHVCAV-NFVEAAADDKISLA------------DCLTDYKVTGNGCA----------P-----------EGDISEV
Volvox_carteri_2 ETLGCTTVICSDKTGTLTTNQMSAVGCSVV-QSCAAGG-------------------ASLMEFEVTGTTYS----------P-----------EGMILGP
Chlamydomonas_reinhardtii_2 ETLGCTTVICSDKTGTLTTNQMSAVGCAVV-QSCAAGG-------------------SALMEFSITGTTYS----------P-----------DGMILGP
Dunaliella_salina_1 ETLGCTTVICSDKTGTLTTNQMSVIKVAAV-QSSS----------------------SQLAEFDVTGTTFS----------P-----------EGMVLGP
Ostreococcus_lucimarinus_2 ETLGCTSVICSDKTGTLTCNVMTVMRMCVI-ENSST---------------------AEVTNYGIRGEAYA----------Q-----------NGEILDS
Phaeodactylum_tricornutum_2 ESLGCATAVASDKTGTLTQNEMTVRTLFAL-AYPK-------------------------AKFGFTGIGYG-SK-------------------SGNLVYL
Chlamydomonas_reinhardtii_3 ETLGSLNVICSDKTGTLTKNEMTVVALRTA-----------------------------ATEYTVSGVGYE---------------------PIGEFAML
Chlamydomonas_reinhardtii_4 ETLGSLNVICSDKTGTLTKNEMTVVALRTA-----------------------------ATEYTVSGVGYE---------------------PVGEFAML
Thalassiosira_pseudonana_4 ETLGSVTVICSDKTGTLTQNIMSLTAFVTS------------------------------------NARYKNNN--------------------------
Thalassiosira_pseudonana_5 ETLGSVTVICSDKTGTLTQNIMSLTAFVTS------------------------------------NARYKNN---------------------------
```

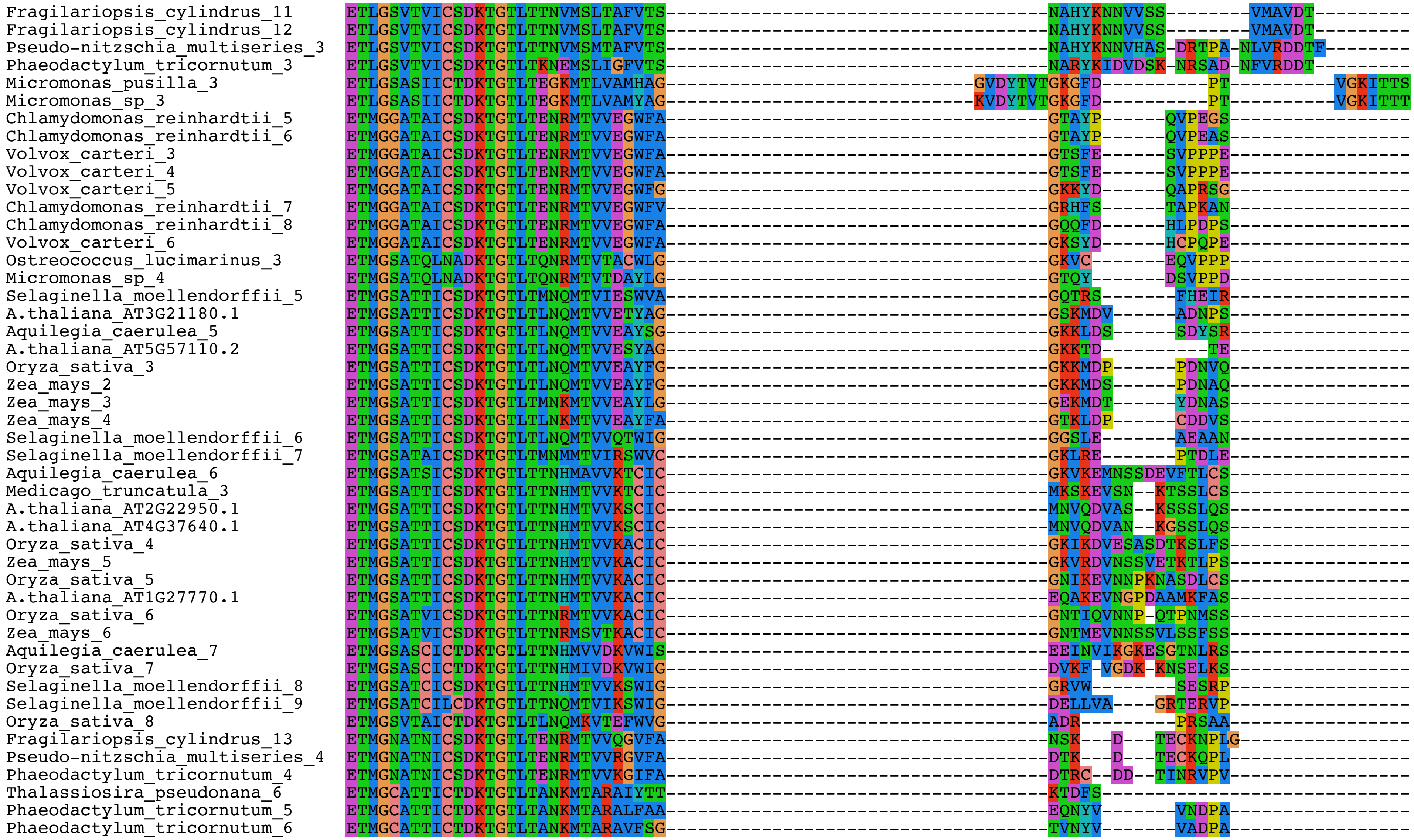
Fragilariopsis_cylindrus_11
Fragilariopsis_cylindrus_12
Pseudo-nitzschia_multiseries_3
Phaeodactylum_tricornutum_3
Micromonas_pusilla_3
Micromonas_sp_3
Chlamydomonas_reinhardtii_5
Chlamydomonas_reinhardtii_6
Volvox_carteri_3
Volvox_carteri_4
Volvox_carteri_5
Chlamydomonas_reinhardtii_7
Chlamydomonas_reinhardtii_8
Volvox_carteri_6
Ostreococcus_lucimarinus_3
Micromonas_sp_4
Selaginella_moellendorffii_5
A.thaliana_AT3G21180.1
Aquilegia_caerulea_5
A.thaliana_AT5G57110.2
Oryza_sativa_3
Zea_mays_2
Zea_mays_3
Zea_mays_4
Selaginella_moellendorffii_6
Selaginella_moellendorffii_7
Aquilegia_caerulea_6
Medicago_truncatula_3
A.thaliana_AT2G22950.1
A.thaliana_AT4G37640.1
Oryza_sativa_4
Zea_mays_5
Oryza_sativa_5
A.thaliana_AT1G27770.1
Oryza_sativa_6
Zea_mays_6
Aquilegia_caerulea_7
Oryza_sativa_7
Selaginella_moellendorffii_8
Selaginella_moellendorffii_9
Oryza_sativa_8
Fragilariopsis_cylindrus_13
Pseudo-nitzschia_multiseries_4
Phaeodactylum_tricornutum_4
Thalassiosira_pseudonana_6
Phaeodactylum_tricornutum_5
Phaeodactylum_tricornutum_6

```
                                701
Skeletonema_marinoi_ABC         EH--------------SVEI------------QNNPTG-ALADVAAVSALCNDATIVGN----------DAPKAA------------------GKT--YE
Thalassiosira_pseudonana_1      QH--------------SSEI------------ADNPKG-SVSDVAAVASLCNDAIIAA-----------------------------------SKT--YE
Thalassiosira_oceanica_1        EH--------------ELEV------------LRNPHG-AVADIAAVSSLCNDARIKGN----------NNPEGT------------------VKA--FD
Phaeodactylum_tricornutum_1     QY--------------NKEV------------KEDPLG-SVADVAAVCALCNDAKILGI----------DS----------------------EKA--FQ
Fragilariopsis_cylindrus_1      VFDK-----------YDKEL-------------YSSSG-SINDVIAVASLCNDARIIGN----------NNNNDNNE--ADGKNGKNKN-TQRQRQ--YE
Fragilariopsis_cylindrus_2      VFDK-----------YDKEL-------------YSSSG-SINDVIAVASLCNDARIIGN----------NNNNDNNE--ADGKNGKNKN-TQRQRQ--YE
Fragilariopsis_cylindrus_3      VFDK-----------YDKEL-------------YSSSG-SINDVIAVASLCNDAH--------------------------GKNGKNKN-TQRQRQ--YE
Fragilariopsis_cylindrus_4      VFDK-----------YDKEL-------------YSSSG-SINDVIAVASLCNDAH--------------------------GKNGKNKN-TQRQRQ--YE
Fragilariopsis_cylindrus_5      VFDK-----------YDKEL-------------YSSSG-SINDVIAVASLCNDARIIGN----------NNNNDNNE--ADGKNGKNKN-TQRQRQ--YE
Fragilariopsis_cylindrus_6      VFDK-----------YDKEL-------------YSSSG-SINDVIAVASLCNDARIIGN----------NNNNDNNE--ADGKNGKNKN-TQRQRQ--YE
Fragilariopsis_cylindrus_7      VFDK-----------YDKEL-------------YSSSG-SINDVIAVASLCNDARIIGN----------NNNNDNNE--ADGKNGKNKN-TQRQRQ--YE
Fragilariopsis_cylindrus_8      VFDK-----------YDKEL-------------YSSSG-SINDVIAVASLCNDARIIGN----------NNNNDNNE--ADGKNGKNKN-TQRQRQ--YE
Pseudo-nitzschia_multiseries_1  V--------------------------------SDGFA-LLSDVAAVAALCNDARIMGN----------DH---------DGKDSSN------LHD--FE
Thalassiosira_pseudonana_2      DR--------------SETV-------------RLPNG-AVKDACDIMTLCNDARLIGN----------DVLADNTEK-KNSSGGST------TQQ--YS
Ostreococcus_lucimarinus_1      -------------------------------APKKLDA-SLRAIAKISALCNGAQLEY------------K----------------------NDA--YK
Zea_mays_1                      -------------------W-----------PSLSMDE-NLQMIGKIAAVCNDASIAH------------S----------------------EHQ--YV
Oryza_sativa_1                  -------------------W-----------PSLSMDE-NLQMIAKIAAVCNDASIAH------------S----------------------EHQ--YV
Aquilegia_caerulea_1            -------------------W-----------PAGRMDA-NLQMMAKIASLCNDAGITY------------S----------------------GHQ--YV
A.thaliana_AT1G07810.1          -------------------W-----------PMGRMDA-NLQMIAKIAAICNDANVEQ------------S----------------------DQQ--FV
A.thaliana_AT1G07670.1          -------------------W-----------PTGRMDA-NLQMIAKIAAICNDANVEK------------S----------------------DQQ--FV
Medicago_truncatula_1           -------------------W-----------PAGQLDA-NLQTMAKIAAVCNDAGISQ------------S----------------------EHK--FV
Glycine_max_1                   -------------------W-----------PTGGLDA-NLQMIAKIAAVCNDAGVAQ------------S----------------------EHK--FV
Glycine_max_2                   -------------------W-----------PTSGLDA-NLQMIAKIAAVCNDAGVAQ------------S----------------------EHK--FV
Aquilegia_caerulea_2            -------------------F-----------PSGRMDV-NLQMIAKIAAVCNDAGVSQ------------S----------------------GNH--YV
Selaginella_moellendorffii_1    -------------------Y-----------NPGMMSK-NLQSLAEIAAVCNDAGIMC------------K----------------------GNQ--FR
Physcomitella_patens_1          -------------------L-----------RIGQLDP-NIQSFAEIASLCNDAGILY------------Q----------------------GNT--FK
Physcomitella_patens_2          -------------------L-----------SVGQLDP-NMLSFAEIASLCNDAGIEY------------Q----------------------GNG--FK
Physcomitella_patens_3          -------------------L-----------EPGNLDR-NLETFAEIAARCNDARVSL------------K----------------------NGV--FK
Selaginella_moellendorffii_2    -------------------L-----------PV-SMDR-NLQTIAQIAAVCNDAAITY------------K----------------------GGQ--FR
Glycine_max_3                   -------------------W-----------GCYNMDA-NLQVMAEICAVCNDAGIYF------------D----------------------GRL--FR
Glycine_max_4                   -------------------W-----------GCYNMDV-NLQVMAEICAVCNDAGIYF------------D----------------------GRL--FR
Aquilegia_caerulea_3            -------------------W-----------TCYNMDA-NLQSMAEICAVCNDAGVFR------------K----------------------GRL--FQ
A.thaliana_AT4G00900.1          -------------------W-----------GCNNMDA-NLQAVAEICSICNDAGVFY------------E----------------------GKL--FR
Chlamydomonas_reinhardtii_1     -------------------LG----------HAAALDK-ALQTVAEVCAVCNEAHLEF------------K----------------------GSA--FR
Volvox_carteri_1                -------------------LA----------PGAALDK-ALQTIAEVCAVCSEAQLEF------------K----------------------GSA--FR
Micromonas_pusilla_1            PKP----------------T-----------KSKPLDA-SLAAVSKVCRGCNDAVIEM------------D------------------A---HGH--AK
Micromonas_sp_1                 -------------------L-----------VGGSLDA-CVTAVSRVCVMCNESTVEL------------K----------------------DGA--FR
Selaginella_moellendorffii_3    -------------------L------------PSELDH-NLRTLARICALCNDAGIQF------------K----------------------NGS--YS
Thalassiosira_oceanica_2        -------------------V-----------DFGKSPA-QLGMLSSIASLCNSSGIEYD----------EK----------------------GSK--YV
Thalassiosira_pseudonana_3      -------------------V-----------DFVKSSA-QLSMLSSIASLCNASSIEYD----------TK----------------------GKK--YV
Pseudo-nitzschia_multiseries_2  ---------------------------------TSTTG-NLEQLANVCALCNEATIRY------------L----------------------EGQ--YV
Fragilariopsis_cylindrus_9      NTS----------------------------ADTSNGS-SLEMMALVCTLCNEAIIKY------------K----------------------DGQ--YV
Fragilariopsis_cylindrus_10     NTS----------------------------ADTSNGS-SLEMMALVCTLCNEAIIKY------------K----------------------DGQ--YV
Aquilegia_caerulea_4            S---------------GLQL-----------EFPAQCP-CLLHIAMCSALCNESMLQYN----------PD----------------------KGN--YE
Glycine_max_5                   T---------------GLQL-----------DFPAQLP-CLLHMAMCSALCNESTLQYN----------PD----------------------KGN--YE
Medicago_truncatula_2           A---------------GVQL-----------DTPAQLQ-CLLHMAMCSALCNESTLQYN----------PD----------------------KGK--YE
A.thaliana_AT1G10130.1          N---------------GMQL-----------DLPAQSP-CLHHLAMCSSLCNDSILQYN----------PD----------------------KDS--YE
Oryza_sativa_2                  G---------------GLQL-----------EFPPQSS-CLLHIAMCSALCNESTLQYN----------PD----------------------KKC--YE
Selaginella_moellendorffii_4    N---------------NQQI-----------DYPAALP-SLVQAAICSSLCNDCSIQYN----------AE----------------------KDI--YE
Micromonas_pusilla_2            NS--------------GKVV-----------EHPAERA-SVLHMSICASLCNDASLTYN----------GK----------------------TRA--YE
Micromonas_sp_2                 LT--------------EKVV-----------DRPANLP-SILHLAICSSLCNDSSLSYN----------GK----------------------THS--FD
Volvox_carteri_2                S---------------GAVL-----------QRPADSP-CLLHLAMASSLCNDSALVYR----------PD----------------------KGT--YQ
Chlamydomonas_reinhardtii_2     G---------------GAVL-----------QRPADSP-CLLHLAMGAALCNDSALVYR----------PD----------------------KGT--YQ
Dunaliella_salina_1             G---------------GVVL-----------RQPADTP-CLAHAAQCAALCNDSQVFVA----------QK----------------------TGK--LQ
Ostreococcus_lucimarinus_2      R---------------GLVV-----------HEPADAA-AVAYVAMCCSMCNDSTLNFD----------KE----------------------KGS--FE
Phaeodactylum_tricornutum_2     DADGS------TGPKAPS---------GKVTSECDEYA-ALSALLNTACLCNNATLLQS----------LDSELS------------------EGHTGGA
Chlamydomonas_reinhardtii_3     VPDAA-------ATDKGAAVANGSGTRSQP-LDGAQRT-ALQQLLKGTVLCNDSSLIKEGPGEVAISIGDDGAAADVAGAADKGGKDKG-GGGGVR--YS
Chlamydomonas_reinhardtii_4     VPDAA-------ATDKGAAIANGSGTRSQP-LDGAQRT-ALQQLLKGTVLCNDSSLVKEGPGEVAISIGDDGAAADVAGAADKGEKDKG-GGKGVR--YS
Thalassiosira_pseudonana_4      -----PEGVKPAHPDDNFPVSNG---------ESPTKD-WIQSALACGVLCSKCVLGEG----------------------------------GGR--AG
Thalassiosira_pseudonana_5      -------GIKSAHPDDSFPVKNG---------ESPSKD-WIRSALACGVLCSKCVLGEG----------------------------------GGR--AG
```

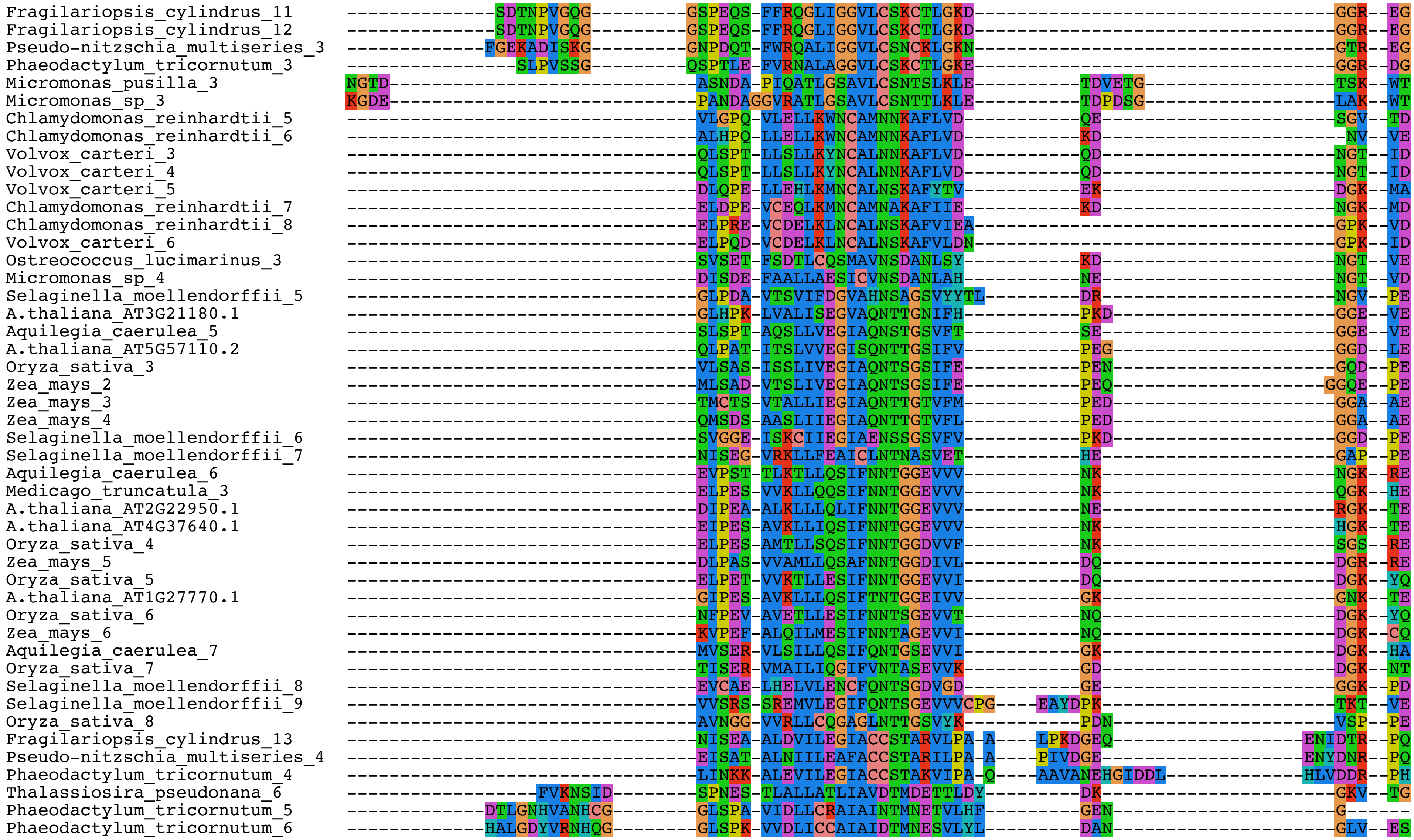

Fragilariopsis_cylindrus_11
Fragilariopsis_cylindrus_12
Pseudo-nitzschia_multiseries_3
Phaeodactylum_tricornutum_3
Micromonas_pusilla_3
Micromonas_sp_3
Chlamydomonas_reinhardtii_5
Chlamydomonas_reinhardtii_6
Volvox_carteri_3
Volvox_carteri_4
Volvox_carteri_5
Chlamydomonas_reinhardtii_7
Chlamydomonas_reinhardtii_8
Volvox_carteri_6
Ostreococcus_lucimarinus_3
Micromonas_sp_4
Selaginella_moellendorffii_5
A.thaliana_AT3G21180.1
Aquilegia_caerulea_5
A.thaliana_AT5G57110.2
Oryza_sativa_3
Zea_mays_2
Zea_mays_3
Zea_mays_4
Selaginella_moellendorffii_6
Selaginella_moellendorffii_7
Aquilegia_caerulea_6
Medicago_truncatula_3
A.thaliana_AT2G22950.1
A.thaliana_AT4G37640.1
Oryza_sativa_4
Zea_mays_5
Oryza_sativa_5
A.thaliana_AT1G27770.1
Oryza_sativa_6
Zea_mays_6
Aquilegia_caerulea_7
Oryza_sativa_7
Selaginella_moellendorffii_8
Selaginella_moellendorffii_9
Oryza_sativa_8
Fragilariopsis_cylindrus_13
Pseudo-nitzschia_multiseries_4
Phaeodactylum_tricornutum_4
Thalassiosira_pseudonana_6
Phaeodactylum_tricornutum_5
Phaeodactylum_tricornutum_6

```
                                801
Skeletonema_marinoi_ABC         RIGEPTEAALC-----------VLAE-KI------------GGKFSD----------------------------------------------SASTS--
Thalassiosira_pseudonana_1      RMGEPTEAALC-----------VLTE-KL------------GGKVS-----------------------------------------------TESTA--
Thalassiosira_oceanica_1        RIGEPTEAALC-----------VLAE-KL------------GGKSK-----------------------------------------------KRSSL--
Phaeodactylum_tricornutum_1     RVGEPTEAALC-----------ILAE-KL------------GGMSHY-------------------LEKGRLD----------------KKGLHFDVP--
Fragilariopsis_cylindrus_1      RTGEPTEAALC-----------ILAE-KI------------GTASSA-------------------SASSSATSS-SASPSSGNDSFPLKQQQQQQLQ--
Fragilariopsis_cylindrus_2      RTGEPTEAALC-----------ILAE-KI------------GTASSA-------------------SASSSATSS-SASPSSGNDSFPLKQQQQQQLQ--
Fragilariopsis_cylindrus_3      RTGEPTEAALC-----------ILAE-KI------------GTASSA-------------------SASSSATSS-SASPSSG-----------------
Fragilariopsis_cylindrus_4      RTGEPTEAALC-----------ILAE-KI------------GTASSA-------------------SASSSATSS-SASPSSG-----------------
Fragilariopsis_cylindrus_5      RTGEPTEAALC-----------ILAE-KI------------GTASSA-------------------SASSSATSS-SASPSSGNDSFPLKQQQQQQLQ--
Fragilariopsis_cylindrus_6      RTGEPTEAALC-----------ILAE-KI------------GTASSA-------------------SASSSATSS-SASPSSGNDSFPLKQQQQQQLQ--
Fragilariopsis_cylindrus_7      RTGEPTEAALC-----------ILAE-KI------------GTASSA-------------------SASSSATSS-SASPSSGNDSFPLKQQQQQQLQ--
Fragilariopsis_cylindrus_8      RTGEPTEAALC-----------ILAE-KI------------GTASSA-------------------SASSSATSS-SASPSSGNDSFPLKQQQQQQLQ--
Pseudo-nitzschia_multiseries_1  RTGEPTEAALC-----------VLAE-KL------------GALQDK-------------------KSTED------------------DEQQQQKKP--
Thalassiosira_pseudonana_2      IEGEPTEAALL-----------VLVE-KL------------GSISAD----------------------------------------------ADES--
Ostreococcus_lucimarinus_1      CVGEPTEGALK-----------VLCE-KI------------GLDDMR-------------------GVAK-----------------------KRSSK--
Zea_mays_1                      ATGMPTEAALK-----------VLVE-KM------------GLPGG-----------------------------------------------YTPSL--
Oryza_sativa_1                  ATGMPTEAALK-----------VLVE-KM------------GLPGG-----------------------------------------------YTPSL--
Aquilegia_caerulea_1            SNGMPTEAALK-----------VLVE-KM------------GLPEGF---------------------------------------------DRSSTA--
A.thaliana_AT1G07810.1          SRGMPTEAALK-----------VLVE-KM------------GFPEG-----------------------------------------------LNEAS--
A.thaliana_AT1G07670.1          SRGMPTEAALK-----------VLVE-KM------------GFPEG-----------------------------------------------LNEAS--
Medicago_truncatula_1           AHGMPTEAALKARYIFSCSLACVLVE-KM------------GLPEGS---------------------------------------------KNVQSG--
Glycine_max_1                   AHGMPTEAALK-----------VLVE-KM------------GLPEGS---------------------------------------------KVAPSA--
Glycine_max_2                   AHGMPTEAALK-----------VLVE-KM------------GLPEGS---------------------------------------------KVAQSA--
Aquilegia_caerulea_2            ANGMPTEAALK-----------VLVE-KM------------GNPDGS----------------------------------------------HHSSS--
Selaginella_moellendorffii_1    ATGLPTEAALK-----------VLVE-KM------------KLPHNY-------------------NSDY----------------------QNHIVD--
Physcomitella_patens_1          ATGMPTEAALK-----------VVVE-KM------------GVPDPA-------------------TQSIITN-QRSS---------------EALAL--
Physcomitella_patens_2          AIGMPTEAALK-----------VVVE-KM------------GVPDFA-------------------AQSVINN-QRSR---------------EAIAL--
Physcomitella_patens_3          AVGMPTEAALK-----------VVVE-KM------------GVPDTD-------------------AQRFIME-QRASV--------------DPSAD--
Selaginella_moellendorffii_2    ASGMPTEAALK-----------VLVE-KI------------GLPDAK---------------------EYCLN-RRA-----------------------
Glycine_max_3                   ATGLPTEAALK-----------VLVE-KM------------GVPDAK-------------------ARNKIRNNTELAA--------------NNMMN--
Glycine_max_4                   ATGLPTEAALK-----------VLVE-KM------------GVPDAK-------------------SRNKIRDNTELAA--------------NNMMN--
Aquilegia_caerulea_3            ATGLPTEAALK-----------VLAE-KM------------GVPDVK-------------------ARNRIRD-AQIAA--------------DCSID--
A.thaliana_AT4G00900.1          ATGLPTEAALK-----------VLVE-KM------------GIPEKK-------------------NSENIEE----VT--------------NFSDN--
Chlamydomonas_reinhardtii_1     AVGAPTEAALL-----------VLAE-KL------------GVADPA-------------------ETAAARK--------------------KRTAT--
Volvox_carteri_1                AVGAPTEAALL-----------VLAE-KL------------GLQDPR-------------------ETAAARK--------------------KRTAT--
Micromonas_pusilla_1            CAGQPTEGALR-----------VLAS-KLE----------RG----------------------------------------------------------
Micromonas_sp_1                 CAGEPTEGALK-----------VLAE-KI------------GVADAA-------------------ANAKIVK--------------------LRAAD--
Selaginella_moellendorffii_3    ATGMPTEAAML-----------VLVE-KL------------GVPDKQ-------------------SLQKFKA--------------------KRMAD--
Thalassiosira_oceanica_2        RVGEPTEASLK-----------VLVE-KI------------GLPDGS-------------------EQAALLE--------------------KRESD--
Thalassiosira_pseudonana_3      RVGEPTEASLK-----------VLVE-KM------------G----------------------------------------------------------
Pseudo-nitzschia_multiseries_2  RVGEPTEAALK-----------CLVE-KM------------GLPGQS-------------------APSNEVE---------------------------
Fragilariopsis_cylindrus_9      RIGEPTEAALK-----------CLVE-KM------------GLPSFT-------------------PPSNEVE---------------------------
Fragilariopsis_cylindrus_10     RIGEPTEAALK-----------CLVE-KM------------GLPSFT-------------------PPSNEVE---------------------------
Aquilegia_caerulea_4            KIGESTEVALR-----------VLTE-KV------------GLPGYD-------------------SMPSSLN----------------------MLS--
Glycine_max_5                   KIGESTEVALR-----------VLAE-KV------------GLPGFN-------------------SMPSSLN----------------------MLT--
Medicago_truncatula_2           KIGESTEVALR-----------VLVE-KV------------GLPGYN-------------------SMPSALN----------------------MLS--
A.thaliana_AT1G10130.1          KIGESTEVALR-----------VLAE-KV------------GLPGFD-------------------SMPSALN----------------------MLS--
Oryza_sativa_2                  KIGESTEVALR-----------VLVE-KV------------GLPGFD-------------------SMPSALN----------------------MLT--
Selaginella_moellendorffii_4    KIGESTEVALR-----------VFSE-KV------------GLPGFD-------------------SMPTSLS----------------------VLS--
Micromonas_pusilla_2            KIGESTEVALR-----------VLTE-KI------------GLPGFD-------------------AMPSALT----------------------RLS--
Micromonas_sp_2                 KIGESTEVALR-----------VLAE-KI------------GLPGFD-------------------DMPRALT----------------------YLS--
Volvox_carteri_2                RIGEATELALR-----------VFAE-KV------------GLPASVGDHPGPLYVAG--------SGPAAVS---------------------- MG--
Chlamydomonas_reinhardtii_2     RIGEATELALR-----------VFAE-KV------------GLPASVADHPHVYVPAGSAAAAGLGGGPAAGG---------------------- SGRG
Dunaliella_salina_1             RIGESTEIALR-----------VFAE-KI------------GLPSSV-------------------RPDRPIS---------------------------
Ostreococcus_lucimarinus_2      KIGEATEIALR-----------VLTE-KI------------GLPSDS-------------------    GIL----------------------GRA--
Phaeodactylum_tricornutum_2     LSGQPTELALL-----------VAAD-KA------------NLE--------------------------------------------------------
Chlamydomonas_reinhardtii_3     PLGAPTEVALL-----------TAAE-KA------------GLPPAA-----------------------------------------------------
Chlamydomonas_reinhardtii_4     PLGAPTEVALL-----------TAAE-KA------------GLPPAA-----------------------------------------------------
Thalassiosira_pseudonana_4      EIGNPTELSIL-----------RASY-FA------------GVDIE------------------------------------------------------
Thalassiosira_pseudonana_5      EIGNPTELSIL-----------RASY-FA------------GIDIE------------------------------------------------------
```

```
Fragilariopsis_cylindrus_11        EIGNPTELSIL-----------RASY-WA------------GIDVN------------------------------------------------------
Fragilariopsis_cylindrus_12        EIGNPTELSIL-----------RASY-WA------------GIDVN------------------------------------------------------
Pseudo-nitzschia_multiseries_3     EIGNPTELSIL-----------RATY-WS------------GVDVN------------------------------------------------------
Phaeodactylum_tricornutum_3        EIGNPTEISIL-----------RASY-FA------------DINVS------------------------------------------------------
Micromonas_pusilla_3               PRGNSSEAPLV-----------VAGQ-KV------------GIKLE------------------------------------------------------
Micromonas_sp_3                    PRGNSSEAPLV-----------VAGH-KC------------GIKLE------------------------------------------------------
Chlamydomonas_reinhardtii_5        FVGNRTECALL-----------VLLR-KL------------GFDYK------------------------------------------------------
Chlamydomonas_reinhardtii_6        FVGNRTECALL-----------VLLR-KL------------GHDYV------------------------------------------------------
Volvox_carteri_3                   FVGNRTECALL-----------VLLR-KL------------GFDYK------------------------------------------------------
Volvox_carteri_4                   FVGNRTECALL-----------VLLR-KL------------GFDYK------------------------------------------------------
Volvox_carteri_5                   FVGNRTECALL-----------LLMNRDL------------ESDYK------------------------------------------------------
Chlamydomonas_reinhardtii_7        FVGNRTECALL-----------LFMNKEL------------GSNYN------------------------------------------------------
Chlamydomonas_reinhardtii_8        FVGNRTECALL-----------MMIK-TW------------GCTYT------------------------------------------------------
Volvox_carteri_6                   FVGNRTECALL-----------MMLR-NW------------GCDYA------------------------------------------------------
Ostreococcus_lucimarinus_3         HLGSKTECALL-----------QLVE-QMQP------PTDDTQTYI------------------------------------------------------
Micromonas_sp_4                    HIGSKTECALL-----------QLVE-DLRSAGKGNLPESSNFAYV------------------------------------------------------
Selaginella_moellendorffii_5       VAGSPTEKALL-----------SWGL-QL------------GMDYS------------------------------------------------------
A.thaliana_AT3G21180.1             ISGSPTEKAIL-----------SWAY-KL------------GMKFD------------------------------------------------------
Aquilegia_caerulea_5               ISGSPTEKAIL-----------SWGV-KL------------GMKFE------------------------------------------------------
A.thaliana_AT5G57110.2             YSGSPTEKAIL-----------GWGV-KL------------GMNFE------------------------------------------------------
Oryza_sativa_3                     VTGSPTEKAIL-----------SWGL-KL------------GMRFN------------------------------------------------------
Zea_mays_2                         VTGSPTEKAIL-----------SWGL-KL------------GMKFS------------------------------------------------------
Zea_mays_3                         ITGSPTEKAIL-----------SWGL-MI------------GMDFK------------------------------------------------------
Zea_mays_4                         LTGSPTEKAIL-----------SWGL-KI------------GMDFD------------------------------------------------------
Selaginella_moellendorffii_6       VTGSPTEKAIL-----------GWGL-KA------------GMNFE------------------------------------------------------
Selaginella_moellendorffii_7       ITGTPTEVAVL-----------GWGV-KL------------GANFD------------------------------------------------------
Aquilegia_caerulea_6               ILGTPTETALL-----------EFGL-SL------------GGDFK------------------------------------------------------
Medicago_truncatula_3              ILGTPTETAIL-----------EFGL-SL------------GGDFQ------------------------------------------------------
A.thaliana_AT2G22950.1             ILGTPTETAIL-----------ELGL-SL------------GGKFQ------------------------------------------------------
A.thaliana_AT4G37640.1             LLGTPTETAIL-----------ELGL-SL------------GGKFQ------------------------------------------------------
Oryza_sativa_4                     ILGTPTETAIL-----------EFGL-SL------------GGDFL------------------------------------------------------
Zea_mays_5                         ILGTPTEAAIL-----------EFGL-SL------------GGDFA------------------------------------------------------
Oryza_sativa_5                     ILGTPTETALL-----------EFAL-SL------------GGNFK------------------------------------------------------
A.thaliana_AT1G27770.1             ILGTPTETALL-----------EFGL-SL------------GGDFQ------------------------------------------------------
Oryza_sativa_6                     ILGTPTETALL-----------EFAL-LL------------DGDCK------------------------------------------------------
Zea_mays_6                         ILGTPTEAALL-----------DFAL-TI------------GGDFK------------------------------------------------------
Aquilegia_caerulea_7               ILGTPTESALL-----------EFGL-LW------------GGDFG------------------------------------------------------
Oryza_sativa_7                     ILGLATETALL-----------EFGL-SL------------EEHLY------------------------------------------------------
Selaginella_moellendorffii_8       LIGTPTETAVL-----------SFGI-SL------------GGNFK------------------------------------------------------
Selaginella_moellendorffii_9       VIGTPTETALL-----------QFGL-DL------------AGNWQG-----------------------------------------------------
Oryza_sativa_8                     ITGSPTEKALL-----------SWAVEEL------------PMDAD------------------------------------------------------
Fragilariopsis_cylindrus_13        LIGSKTEAAMI-----------LLSQ-SE------------WGNNDD-----------------------------------------------------
Pseudo-nitzschia_multiseries_4     VIGSKTEGAMI-----------LLSQ-SE------------WGNHDD-----------------------------------------------------
Phaeodactylum_tricornutum_4        IIGNKTEAALL-----------ILAR-SS------------WTPHDD-----------------------------------------------------
Thalassiosira_pseudonana_6         STGNPTEVALL-----------VLAA-DL------------GKDYR------------------------------------------------------
Phaeodactylum_tricornutum_5        -------------------------D-DN------------VRQSH------------------------------------------------------
Phaeodactylum_tricornutum_6        SCGNPTEVALL-----------HLCH-DL------------GSNYE------------------------------------------------------
```

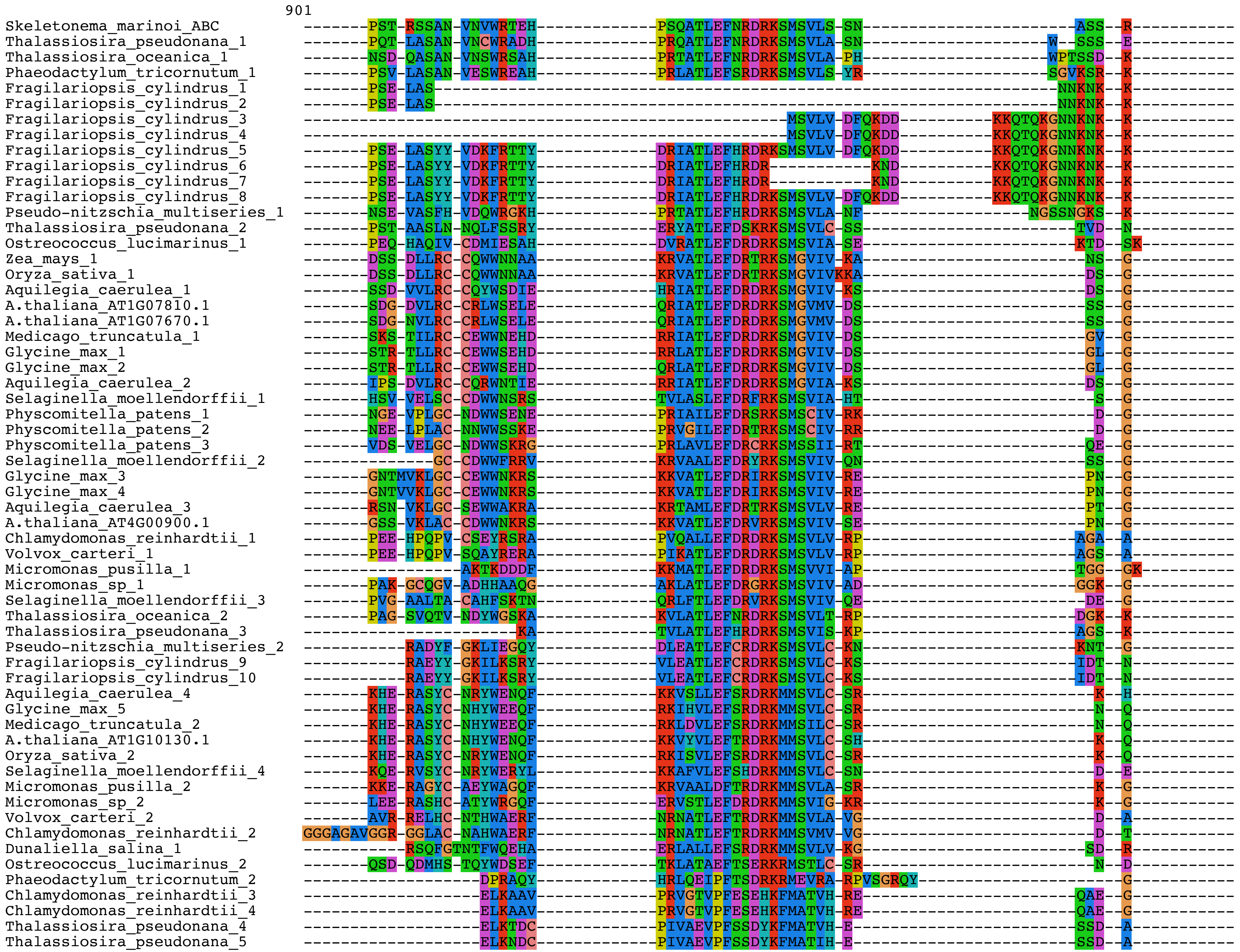
901
Skeletonema_marinoi_ABC
Thalassiosira_pseudonana_1
Thalassiosira_oceanica_1
Phaeodactylum_tricornutum_1
Fragilariopsis_cylindrus_1
Fragilariopsis_cylindrus_2
Fragilariopsis_cylindrus_3
Fragilariopsis_cylindrus_4
Fragilariopsis_cylindrus_5
Fragilariopsis_cylindrus_6
Fragilariopsis_cylindrus_7
Fragilariopsis_cylindrus_8
Pseudo-nitzschia_multiseries_1
Thalassiosira_pseudonana_2
Ostreococcus_lucimarinus_1
Zea_mays_1
Oryza_sativa_1
Aquilegia_caerulea_1
A.thaliana_AT1G07810.1
A.thaliana_AT1G07670.1
Medicago_truncatula_1
Glycine_max_1
Glycine_max_2
Aquilegia_caerulea_2
Selaginella_moellendorffii_1
Physcomitella_patens_1
Physcomitella_patens_2
Physcomitella_patens_3
Selaginella_moellendorffii_2
Glycine_max_3
Glycine_max_4
Aquilegia_caerulea_3
A.thaliana_AT4G00900.1
Chlamydomonas_reinhardtii_1
Volvox_carteri_1
Micromonas_pusilla_1
Micromonas_sp_1
Selaginella_moellendorffii_3
Thalassiosira_oceanica_2
Thalassiosira_pseudonana_3
Pseudo-nitzschia_multiseries_2
Fragilariopsis_cylindrus_9
Fragilariopsis_cylindrus_10
Aquilegia_caerulea_4
Glycine_max_5
Medicago_truncatula_2
A.thaliana_AT1G10130.1
Oryza_sativa_2
Selaginella_moellendorffii_4
Micromonas_pusilla_2
Micromonas_sp_2
Volvox_carteri_2
Chlamydomonas_reinhardtii_2
Dunaliella_salina_1
Ostreococcus_lucimarinus_2
Phaeodactylum_tricornutum_2
Chlamydomonas_reinhardtii_3
Chlamydomonas_reinhardtii_4
Thalassiosira_pseudonana_4
Thalassiosira_pseudonana_5

```
Fragilariopsis_cylindrus_11        -------------------AVKDAC-------------PIIAEVPFSSEYKFMATVH-ESN---------------------LDND--G-----------
Fragilariopsis_cylindrus_12        -------------------AVKDAC-------------PIIAEVPFSSEYKFMATVH-ESN---------------------LDND--G-----------
Pseudo-nitzschia_multiseries_3     -------------------EVKEAC-------------PIIAEVPFSSEYKFMATVH-ESS---------------------PEND--G-----------
Phaeodactylum_tricornutum_3        -------------------GMKEEA-------------PIVAEVPFSSEYKFMATVH-EPR---------------------VEND--G-----------
Micromonas_pusilla_3               -------------------DLEDAL-------------ERTAEIPFSSSRKMMVTVT-KTVKQ--------------TPLAHVAKI--G-----------
Micromonas_sp_3                    -------------------DLEASL-------------TRVYEIPFSSSRKMMVTIT-KTTGA--------------TSLSHVAAS--G-----------
Chlamydomonas_reinhardtii_5        -------------------QLREER-----------EADQIKLYGFSSARKMASVLL-RER----------------------AAG--G-----------
Chlamydomonas_reinhardtii_6        -------------------QLREQR-----------EADQVKLYGFSSARKMASVLL-RE-----------------------PSS--G-----------
Volvox_carteri_3                   -------------------QIREER-----------EQDQIKMYGFSSARKMASVLV-R------------------------EPS--G-----------
Volvox_carteri_4                   -------------------QIREER-----------EQDQIKMYGFSSARKMASVLV-R------------------------EPS--G-----------
Volvox_carteri_5                   -------------------AYRTQH-----------ERSVEKIYGFTSAKKMASVLV-R------------------------LGD--------------
Chlamydomonas_reinhardtii_7        -------------------DYRHKY-----------DKAVVKLYGFSSAKKMASVLI-Q------------------------LPD--------------
Chlamydomonas_reinhardtii_8        -------------------AVREEY-----------EASVYKMFGFSSSKKMASCSV-K------------------------FAD--------------
Volvox_carteri_6                   -------------------SVRDEY-----------DASVFKVFGFSSTKKMASATI-K------------------------FAD--------------
Ostreococcus_lucimarinus_3         -------------------KLREKF-------------PVAQLYHFTSARKRMSTAI-S------------------------NGS--------------
Micromonas_sp_4                    -------------------AGREKH-------------DVAQRYHFTSARKRMSTAV-P------------------------MN --------------
Selaginella_moellendorffii_5       -------------------TVRAAS-------------SIIAVEPFNSTKKMAGVAI-K------------------------RNN--G-----------
A.thaliana_AT3G21180.1             -------------------TIRSES-------------AIIHAFPFNSEKKRGGVAV-L------------------------RGD--S-----------
Aquilegia_caerulea_5               -------------------VVRSEA-------------SILHVFPFNSEKKRGGLAV-K------------------------LPN--N-----------
A.thaliana_AT5G57110.2             -------------------TARSQS-------------SILHAFPFNSEKKRGGVAV-K------------------------TAD--G-----------
Oryza_sativa_3                     -------------------DTRTKS-------------SILHVFPFNSEKKRGGVAV-H------------------------LGGSES-----------
Zea_mays_2                         -------------------ETRSKS-------------SILHVFPFNSEKKRGGVAV-Y------------------------LAG--S-----------
Zea_mays_3                         -------------------DVRSKS-------------SVLHVVPFNSEKKRGGVAL-Q------------------------VSD--T-----------
Zea_mays_4                         -------------------DVRTKS-------------SVIHVFPFNSEKKRGAVAV-Q------------------------LDD--G-----------
Selaginella_moellendorffii_6       -------------------EVRSSN-------------TVMHVETFNSTKKRAGVAF-K------------------------RKD--G-----------
Selaginella_moellendorffii_7       -------------------RVKKSA-------------TVTEVDAFNSTKKRMAVIA-K------------------------TED--G-----------
Aquilegia_caerulea_6               -------------------AERESA-------------KLVKVEPFNSEKKRMGVVL-E------------------------LPE--G-----------
Medicago_truncatula_3              -------------------GERQAC-------------KLVKVEPFNSTKKRMGAVV-E------------------------LPS--G-----------
A.thaliana_AT2G22950.1             -------------------EERQSN-------------KVIKVEPFNSTKKRMGVVI-E------------------------LPEG-G-----------
A.thaliana_AT4G37640.1             -------------------EERKSY-------------KVIKVEPFNSTKKRMGVVI-E------------------------LPEG-G-----------
Oryza_sativa_4                     -------------------AVRKAS-------------TLVKVEPFNSAKKRMGVVI-Q------------------------LPG--G-----------
Zea_mays_5                         -------------------AVRKAS-------------TLLKVEPFNSARKRMGVVI-Q------------------------LPG--G-----------
Oryza_sativa_5                     -------------------AKRDET-------------KIVKMEPFNSTKKRMCVVL-K------------------------LPG--G-----------
A.thaliana_AT1G27770.1             -------------------EVRQAS-------------NVVKVEPFNSTKKRMGVVI-E------------------------LPE--R-----------
Oryza_sativa_6                     -------------------EKQLGS-------------KIVKVEPFNSTKKRMSTIL-E------------------------LPG--G-----------
Zea_mays_6                         -------------------EKRQET-------------KIVKVEPFNSTKKRMGIIL-E------------------------LPG--G-----------
Aquilegia_caerulea_7               -------------------SQRQES-------------KIVKVEPFNSDKKKMSVLV-S------------------------LST--G-----------
Oryza_sativa_7                     -------------------DDYNKL-------------TRIKVDPFNSVKKKMSVTI-Q------------------------LPN--G-----------
Selaginella_moellendorffii_8       -------------------DVRSQS-------------SILKVEPFNSAKKRMGVLV-K------------------------GGH--G-----------
Selaginella_moellendorffii_9       --L--------------VSEVRSRS-------------RVIRVEPFNSVKKMMGVLV-A------------------------VNG--GG----------
Oryza_sativa_8                     -------------------ALKRKC-------------KVVRVEAFNSDKKRSGVML-RD-----------------------AAT--G-----------
Fragilariopsis_cylindrus_13        ------------------TDKRREA--ANFGK-----ESGSRLFPFSSSRKRMTVLV-K------------------------KGG--N-----------
Pseudo-nitzschia_multiseries_4     ------------------TDMRREA--ANFGK-----EGGSRLFPFSSSRKRMTVLV-K------------------------KGG--D-----------
Phaeodactylum_tricornutum_4        ------------------TDQRRVD--ANFGA-----EGGSRLFPFSSSRKCMTVFV-T------------------------KDE--A-AVSDTSIRTR
Thalassiosira_pseudonana_6         -------------------DIRDSTRGRSDKGELAEYLVEGKQIGFSSARKMMSWAV-P------------------------SEG--G-----------
Phaeodactylum_tricornutum_5        -------------------RIRASTKGRSDQASLAEFFSEGKRFDFSSARKMMSWAI-P------------------------Y E--G-----------
Phaeodactylum_tricornutum_6        -------------------NIRNRTRGRSDQGSLGAFLCQGKQYGFSSARKMMSWAV-P------------------------LAD--G-----------
```

```
                                   1001
Skeletonema_marinoi_ABC            -------GNRLLVKGAPNMLLERCTHAKL-RDGKI----VKLDGKLRRQIEQKTTE---------LA-TRPLRCLALAVKETNQLEQSLRHYSQE-----
Thalassiosira_pseudonana_1         -------GNRLLVKGAPNLLLERCTHAKC-RDGTV----VKLDGKLRRQIEQKTTE---------LA-TRPLRCLALAIKETDHLEE--------------
Thalassiosira_oceanica_1           -------GNRLLVKGAPNLLLPRCTHAKM-RDGSV----VKLDGKLRRQIEQKTSD---------LA-SRPLRCLGLAVKESANLEQSLRTYSQE-----
Phaeodactylum_tricornutum_1        ------AGNRLLIKGAPNLLIERCTNVKF-RDGTI----APMTGALRRSIEDQVSK---------MA-ARPLRCLALAIKDQDELDDSLKSFEPD-----
Fragilariopsis_cylindrus_1         ------PCNRLLVKGAANMVVDRCTHIKY-RDGSI----GRMTNVLKKEIDSKITD---------MA-TRPLRCIALAIKEESQLPKSLKNFQSN-----
Fragilariopsis_cylindrus_2         ------PCNRLLVKGAANMVVDRCTHIKY-RDGSI----GRMTNVLKKEIDSKITD---------MA-TRPLRCIALAIKEESQLPKSLKNFQSN-----
Fragilariopsis_cylindrus_3         ------PCNRLLVKGAANMVVDRCTHIKY-RDGSI----GRMTNVLKKEIDSKITD---------MA-TRPLRCIALAIKEESQLPKSLKNFQSN-----
Fragilariopsis_cylindrus_4         ------PCNRLLVKGAANMVVDRCTHIKY-RDGSI----GRMTNVLKKEIDSKITD---------MA-TRPLRCIALAIKEESQLPKSLKNFQSN-----
Fragilariopsis_cylindrus_5         ------PCNRLLVKGAANMVVDRCTHIKY-RDGSI----GRMTNVLKKEIDSKITD---------MA-TRPLRCIALAIKEESQLPKSLKNFQSN-----
Fragilariopsis_cylindrus_6         ------PCNRLLVKGAANMVVDRCTHIKY-RDGSI----GRMTNVLKKEIDSKITD---------MA-TRPLRCIALAIKEESQLPKSLKNFQSN-----
Fragilariopsis_cylindrus_7         ------PCNRLLVKGAANMVVDRCTHIKY-RDGSI----GRMTNVLKKEIDSKITD---------MA-TRPLRCIALAIKEESQLPKSLKNFQSN-----
Fragilariopsis_cylindrus_8         ------PCNRLLVKGAANMVVDRCTHIKY-RDGSI----GRMTNVLKKEIDSKITD---------MA-TRPLRCIALAIKEESQLPKSLKNFQSN-----
Pseudo-nitzschia_multiseries_1     ------AHNRLLVKGAANMVLDRCTHVKY-RDGSV----AKISGSLRRELEKKITD---------MA-TRPLRCLALAVKDESKLQSSLRHFQPR-----
Thalassiosira_pseudonana_2         -------QNKLFVKGAPSMLLRRCSHAKL-RDGKV----VPLTPQLRSQIEDEISS---------IG-DRALRCISLAFKDDSLAPQLQN---------
Ostreococcus_lucimarinus_1         ----RGSANELLVKGAPEVLLERCAFVQM-PDGAT----APLSATMRNVILNEQAT---------MA-RDALRCLAFAKKV---SL-GDLSSYDGS-----
Zea_mays_1                         -------KNLLLVKGAVENLLERCTHIQL-LDGSV----VLLDDGAKALILSTLRD---------MS-ASALRCLGFAYKE--EL-SEFATYDG------
Oryza_sativa_1                     -------KNLLLVKGAVENLLERSGYIQL-LDGSV----VLLDEGAKALILSTLRE---------MS-ASALRCLGFAYKE--DL-AEFATYDG------
Aquilegia_caerulea_1               -------RNSLLVKGAVENLLERSKYIQL-LDGSV----ISLDPNCRELILQALHE---------MS-STALRCLGFAYKD--DL-AEFATYNGD-----
A.thaliana_AT1G07810.1             -------NKLLLVKGAVENVLERSTHIQL-LDGSK----RELDQYSRDLILQSLRD---------MS-LSALRCLGFAYSD--VP-SDFATYDGS-----
A.thaliana_AT1G07670.1             -------KKLLLVKGAVENVLERSTHIQL-LDGST----RELDQYSRDLILQSLHD---------MS-LSALRCLGFAYSD--VP-SDFATYDGS-----
Medicago_truncatula_1              ------KKKSLLVKGAVENVLDRSSKVQL-RDGSV----VKLDNNAKNLILQALHE---------MS-TSALRCLGFAYKD--EL-TNFENYNGN-----
Glycine_max_1                      -------KRSLLVKGAVENVLDRSSKIQL-RDGSI----VNLDDNARNLVLQALHE---------MS-TSALRCLGFAYKD--EL-PKFENYSGN-----
Glycine_max_2                      -------KRSLLVKGAVENVLDRSSKIQL-RDGSI----VNLDDNARNLVLQALHE---------MS-TSALRCLGFAYKD--EL-PKFENYSGN-----
Aquilegia_caerulea_2               -------RNSLLVKGAVENLLERSSFLQL-LDGSI----VELDQNSRKLILQSLHE---------MS-TTALRCLGFAYKD--NL-SEFATYDGD-----
Selaginella_moellendorffii_1       -------KNRLLVKGAVENLLERSSYLQL-KDGSI----VSLDSNSRDAWIKKLDS---------MS-SKALRCLGFAYKD--NL-GDFSSYNG------
Physcomitella_patens_1             -------VNRLLVKGAVENILERSTRVQL-SDGSV----VKMTQSARDDLLAKLDS---------LS-AKALRCLGLAYKD--DL-QDLGDYDG------
Physcomitella_patens_2             -------VNRLLVKGAVENILERCTRVQL-LDGSV----ANMTEGARDALLGKLNG---------LS-ARALRCLGLAYKD--DL-QELSDYDG------
Physcomitella_patens_3             -------TNKLLVKGAVENVLERSTHVQL-LDGTV----KEMTEEARSVLLSKIYS---------MS-TKCLRCLGLAYTD--DL-GDLSDYDG------
Selaginella_moellendorffii_2       -------QSTLLVKGAVENVLERCSFMQL-MDGTV----VELDNASRAAILSKLNT---------MS-SKALRCLGLAYKD--DL-GELSTYDG------
Glycine_max_3                      -------QNRLLVKGAVESLLERSSHVQL-ADGSL----VPIDDQCRELLLRRLQE---------MS-SKGLRCLGFAYND--EL-GEFSDYYA------
Glycine_max_4                      -------QNRLLVKGAVESLLERSSHVQL-ADGSV----VPIDDQCRELLLQRLQE---------MS-SKGLRCLGFAYND--DL-GEFSDYYA------
Aquilegia_caerulea_3               -------FNRLLVKGAVESVLERSLHVQL-ADGSI----APMDEPCRQLLLLRHME---------MS-SKGLRCLALAYKD--EL-GEFSDYYS------
A.thaliana_AT4G00900.1             -------QNRLLVKGAAESILERSSFAQL-ADGSL----VALDESSREVILKKHSE---------MT-SKGLRCLGLAYKD--EL-GEFSDYSS------
Chlamydomonas_reinhardtii_1        -------RNALLVKGAAECVIDRCNRMML-PDGRV----VPLTPVARAAVLGAVKD---------MA-RDALRCLALAVKP--DPPAPLSDWDGSD----
Volvox_carteri_1                   -------KNSLLVKGAAECVIERSTRMML-PDGRI----VPLTDAARSAVLGAVQG---------MA-RDALRCLAIAVKP--DVPSPLAEYNGD-----
Micromonas_pusilla_1               ---ANANANELLVKGAPEHVLERCAFVQL-PNGDV----VPLTKAARAAVVKRAET---------MS-ADALRCLALATKSGASL-GALASYDGA-----
Micromonas_sp_1                    -----SNKNSLLVKGAPECVLDRCSKVLL-PDGSV----TTLSPALREEIVATVAE---------MS-SSALRCLGFALKTGAEL-GKLGGYDGG-----
Selaginella_moellendorffii_3       --------NSLLVKGAAEFVLERCTSVQL-KDGSV----VPLTPSFRENIISCINA---------MT-SKGLRVLALASKS--DL-GPLSDYTG------
Thalassiosira_oceanica_2           -------TNQLLVKGAPEGLLARCTHIMQ-ANGKV----VKLDKASADAVSAQQQR---------MS-GRALRVLALAYKD--LS-GDLGSYDGT-----
Thalassiosira_pseudonana_3         -------TNQLLVKGAPEGLISRCNKIML-GNGKV----VSLDKDGVNAILNQQQR---------MA-GRALRVLALAYKD--LS-GDLGSYDGT-----
Pseudo-nitzschia_multiseries_2     -------DRALFVKGAAELLLNRCTSIMR-PDGSV----QALSAKDRVAFAARITD---------MS-KRPLRVLGMAIKT--NVP------------
Fragilariopsis_cylindrus_9         -------KRTLFVKGAAELLLQRCTTIMR-PNGEV----VKLTSIERNALAKQITT---------MS-QRPLRVLGMAIKQ--NMN------------
Fragilariopsis_cylindrus_10        -------KRTLFVKGAAELLLQRCTTIMR-PNGEV----VKLTSIERNALAKQITT---------MS-QRPLRVLGMAIKQ--NMN------------
Aquilegia_caerulea_4               -------QEIVFSKGAPESIISRCTNILCNDDGCT----APLTADIRAELMARFHS---------FAGKDTLRCLALALKE---MPFGQQTLAF-----
Glycine_max_5                      -------MHVLFSKGAPESIISRCTSILCNDDGSI----VSLTADIRAELDSRFHS---------FAGKETLRCLALALKW---MPSTQQSLSF-----
Medicago_truncatula_2              -------LHVLFSKGAPESIISKCTTILCNGDGSV----MPLTADIRAELDSKFNS---------FAGKETLRCLALALKW---MPSDQQKLSF-----
A.thaliana_AT1G10130.1             -------MDVMFSKGAPESIIARCNKILCNGDGSV----VPLTAAGRAELESRFYS---------FG-DETLRCLALAFKT---VPHGQQTISY-----
Oryza_sativa_2                     -------QEIMFSKGAPESVMARCTHILCNDDGSS----VPLTMDIRNELEARFQS---------FAGKDTLRCLALALKR---MPEGQQSLSY-----
Selaginella_moellendorffii_4       -------RTVLLSKGAPEVILQRSSYVVCNQDGSV----TKLSPELKLSLEEKFHRQDLISLDAFFASQETLRCIALAYKD---VPTGQQTLTE-----
Micromonas_pusilla_2               -------QSILFTKGAAETVLAKCTQALTNASGAA----EPLTDAMRAALSDKLQK---------FA-ASSLRVLALAMRP---TPPKTTKVSV-----
Micromonas_sp_2                    -------QSILFTKGSPEAVLLRCTRVLTNSKGIA----EPISTQVRDALTEKYRT---------YA-RRSLRVLALAMRP---ISSDQCHISP-----
Volvox_carteri_2                   -------RSVLWSKGAPESILARCSSVLANNGEGV----VPLTDAARAALTASVKR---------YG-RRALRTLALAYKP---MPSGTKTLAP-----
Chlamydomonas_reinhardtii_2        -------RSIMWSKGAPESILARCTSVLANNGEGV----VPLTDAARGALAAATRR---------FG-HRALRTLALAYKP---LPGGSKALSP-----
Dunaliella_salina_1                -------QHNIWSKGAPEFVLRKCSHVLANNGEGA----VPLTDSMRQAILSDMQA---------FGSRQALRCLALAFKS---VPTSTTKLGY-----
Ostreococcus_lucimarinus_2         -------ESILFVKGAPESVLSLCTSVMSNRNGRA----ERMTDQVREQVAEQMRG---------YA-NDALRVLALAMRP---MGRGVTTCSE-----
Phaeodactylum_tricornutum_2        --------SLYFVKGMPEKILGECSTYGQ-SNGSA----ELLVEDDRAEVLLQARR---------MA-AGGLRVLAFAYGQ----------------
Chlamydomonas_reinhardtii_3        ----GGSELIMYVKGAPDRLLPLCNSQV--VDNDL----SRTAALDPGFWKAQQAA---------LS-SRGLRVLALCRTV---LPEDTDLSVLSP----
Chlamydomonas_reinhardtii_4        ----GGSELIMYVKGAPDRLLPLCNSQV--VDNDL----SRTAALDPGFWKAQQAA---------LS-SRGLRVLALCRTV---LPEDTDLSVLSP----
Thalassiosira_pseudonana_4         --P--DGKYTCFVKGAPDRMVKLCKYQA--KGGVAGE--ENLEEIDEAYWIEQIAV---------LS-SHGLRVLGLCRAY---IDKSSVQAGAQ-----
Thalassiosira_pseudonana_5         --P--EGKYTAFVKGAPDRMVKFCKYQA--KGGATGD--DNYEDIDEAYWIEQIAI---------LS-SHGLRVLGL--------------FGAQ-----
```

```
Fragilariopsis_cylindrus_11         --PGLEDKLIIHVKGAPDRMVKLCNTQA--VAGELG----KTEPINAEFWQEQIAI---------LS-SHGLRVLALLRGT---LEKGSVTPGDS-----
Fragilariopsis_cylindrus_12         --PGLEDKLIIHVKGAPDRMVKLCNTQA--VAGELG----KTEPINAEFWQEQIAI---------LS-SHGLRVLALLRGT---LEKGSVTPGDS-----
Pseudo-nitzschia_multiseries_3      --PGLENKNIIHVKGAPDRMVKLCNTQA--VAGQLG----KTEPINADFWQEQIAI---------LS-SHGLRVLALLRGT---VEKGSVKQGDA-----
Phaeodactylum_tricornutum_3         --AGNDGSLVVHVKGAPDRMVKLCSTQA--KAGLLGE--GNKEPINMGYWTEQIAI---------LS-SHGLRVLALCRGS--E----------------
Micromonas_pusilla_3                -------EQTAHVKGAPNYIMEKCTKYIT-ADGSV----KPLDDAVKKKIMSTVDD---------LS-EQALRVLAVATKN---VGGKLPF-----DSEQ
Micromonas_sp_3                     -------EHTAHVKGAPNYIMEKCTSYMA-ADGSI----VDFDEAAKAEFTAKVDE---------LS-SQALRVLAIATKD---LGAKLPY-----GEEE
Chlamydomonas_reinhardtii_5         -------NLRLYNKGAAEWVLRRCVGLAR-PDGST----EPMTPAKLEEMNALVTG---------MA-KRGLRCICLSYRD---YAGSDP----------
Chlamydomonas_reinhardtii_6         -------NLRLYNKGAAEWVLRRCSSLMR-PDGST----EPMTEARLAEMIELVTS---------MA-KRGLRCICLSYRD---YAGSDP----------
Volvox_carteri_3                    -------NLRLYNKGAAEWVLRRCSAMLQ-PDGST----IPMDKVILEEMNSLVTA---------MA-MRGLRCICLSYRD---YPANDL----------
Volvox_carteri_4                    -------NLRLYNKGAAEWVLRRCSAMLQ-PDGST----IPMDKVILEEMNSLVTA---------MA-MRGLRCICLSYRD---YPANDL----------
Volvox_carteri_5                    -------KYRLYNKGAAEWVLKRCSRCIT-QAGVV----E-MTPALRGQLLDEVTA---------MA-KRGLRCICLTYAD---FALVDP----------
Chlamydomonas_reinhardtii_7         -------KLRLYNKGAAEWVLKRCIRCHT-EAGIV----E-MTPALRGKLLDEVTN---------MA-KRGLRCICLSYTD---YPISDP----------
Chlamydomonas_reinhardtii_8         -------KFRHYNKGAAEWVLKRCTSMYN-GSQIV----Q-MGDAEREKLVEVVTG---------MA-KRGLRCICLTYTD---YPLVDD----------
Volvox_carteri_6                    -------KFRHYNKGAAEWVLKRCTSMYD-GARVI----E-MTEVERARLMEVVTG---------MA-KRGLRCICLTYTD---YPLVDD----------
Ostreococcus_lucimarinus_3          -------GTRLHVKGASEIVVKLCTKMMG-ADGKV----SSLTTPMLKEAEAAIEA---------FA-RQGLRTLCIAYTD---LGKSPS----------
Micromonas_sp_4                     ------GGVRLHVKGASEIVVKLCTKMMK-TDGSV----EDFSPEDLAAAEKAITA---------MA-STGLRTLCIAYVD---LDTAPS----------
Selaginella_moellendorffii_5        -------TLCALWKGAAEIILDLCENWLD-GEGTE----KVLSSEMVSSIHGTLTH---------MA-ASSLRCLAFAIKT---YN--------------
A.thaliana_AT3G21180.1              -------EVFIHWKGAAEIVLACCTQYMD-SNGTL----QSIESQK-EFFRVAIDS---------MA-KNSLRCVAIACRT---QELNQV----------
Aquilegia_caerulea_5                -------EVHIHWKGAAEIVLASCSSFLD-ADGAV----KPLDEDKVAYHKKAIGD---------MA-EGSLRCVALAYRL---FDINKV----------
A.thaliana_AT5G57110.2              -------EVHVHWKGASEIVLASCRSYID-EDGNV----APMTDDKASFFKNGIND---------MA-GRTLRCVALAFRT---YEAEKV----------
Oryza_sativa_3                      -------EVHIHWKGAAEIILDSCKSWLA-ADGSK----HSMTPEKISEFKKFIED---------MA-ASSLRCVAFAYRT---YEMVDV----------
Zea_mays_2                          -------EVHIHWKGAAEIILDSCTSWVD-TGGSK----HSMTPEKVAEFKKFIED---------MA-AASLRCVAFAYRT---HEMDDV----------
Zea_mays_3                          -------EVHIHWKGAAELLLASCRSWLS-TDGSI----QQMNSIKHNEFKERIDD---------MA-MSSLRCVAFAYCP---WEPKMV----------
Zea_mays_4                          --------VHIHWKGAAEIVLSSCKSWLS-VDGSV----QSMSAEKHDEFKRSIED---------MG-ANSLRCVAFAYCS---FDIEKI----------
Selaginella_moellendorffii_6        -------NAYVHWKGAAEIILDLCTKWMG-SDGSE----NQLSETKKVEIQNAIGD---------MA-SRSLRCVALAYRP---ISANQI----------
Selaginella_moellendorffii_7        -------KAWIHWKGASEVVLAQCSNFMD-EQGNV----SPLTPEKLQELQEIIDT---------FA-NAALRTLCLACKE---FPQNEFLAR-------
Aquilegia_caerulea_6                -------GLRAHCKGASEIILDACDKFID-SNGEV----VRLDEATLNHLNNTIEQ---------FA-NEALRTLCLAYME---LGNDY-----------
Medicago_truncatula_3               -------GLRAHCKGASEIVLAACDKVLN-SNGEV----VPLDEESTNHLTNTINQ---------FA-NEALRTLCLAYME---LENGF-----------
A.thaliana_AT2G22950.1              -------RIRAHTKGASEIVLAACDKVIN-SSGEV----VPLDDESIKFLNVTIDE---------FA-NEALRTLCLAYMD---IESGF-----------
A.thaliana_AT4G37640.1              -------RMRAHTKGASEIVLAACDKVVN-SSGEV----VPLDEESIKYLNVTINE---------FA-NEALRTLCLAYMD---IEGGF-----------
Oryza_sativa_4                      -------AMRAHSKGASEIILASCSKYLN-DQGNV----VPLDDATVAHLNATINS---------FA-NEALRTLCLAYVD---VGDGF-----------
Zea_mays_5                          -------ALRAHCKGASEIVLASCTRYLD-ERGSA----VALDGATADRLRATIDS---------FA-NEALRTLCLAYVD---VGDGF-----------
Oryza_sativa_5                      -------GCRAHCKGASEIVLAACDKFMD-ETGAV----VPLDKTTADKLNGIIES---------FA-NEALRTLCLGYRE---MEEGF-----------
A.thaliana_AT1G27770.1              -------HFRAHCKGASEIVLDSCDKYIN-KDGEV----VPLDEKSTSHLKNIIEE---------FA-SEALRTLCLAYFE---IGDEF-----------
Oryza_sativa_6                      -------GYRAHCKGASEIVLAACDKFID-ERGCI----VPLDDKTSSKLNDIIKA---------FS-SEALRTLCLAYRE---MEEGF-----------
Zea_mays_6                          -------GYRAHCKGASEVVLAACDNFID-ARGTI----VALDKTATKKLSDVIET---------FS-KEALRTLCLAYRE---MDDSF-----------
Aquilegia_caerulea_7                -------GFRVFCKGASEIVLRMCDKIVD-KNGKP----ILLSEAQIENVMGIING---------FA-NEALRTLCLAFKD---MDKC------------
Oryza_sativa_7                      -------GIRTFCKGASEIILEQCNTIHN-TDGNI----VPLSEMQKHNVLNIINS---------FA-SEALRTLCIAFKD---MD--------------
Selaginella_moellendorffii_8        -------TIRAHWKGASEIVLGMCDKYLD-TEGNV----CPIDEKKYRELKGIITT---------FA-DEALRTLCMAFRE---LESE------------
Selaginella_moellendorffii_9        --EQSAPSYRVHWKGASEIVMGMCDWYLD-SQGRK----VALDDSKNWELRGIIRR---------FA-DEGLRTLCLAYRD---LEIAP-----------
Oryza_sativa_8                      -------AVTAHWKGAAEMVLARCTVYVG-ADGAA----RELGVEQRRKLEQVIND---------MA-AASLRCIAFAYKQ---VVDGGD----------
Fragilariopsis_cylindrus_13         -------DVIMYHKGAAEIILKNCTQYLD-VDGTQ----KPMTAEKRKYYENIISE---------FA-GDALRCIASAHRN---QIESITK---------
Pseudo-nitzschia_multiseries_4      -------NVTMYHKGAAEIILKNCTKYLD-ANGSQ----MPLTPEKRQYFESIIQE---------FA-EDALRCIAIAHRN---EIQTITK---------
Phaeodactylum_tricornutum_4         RATKNVQSYTLYHKGAAEIVLDKCTKYLD-IDGTE----KEMSDQKREEFAKLIRE---------FA-SQALRCVALAHRR---DIQNVV----------
Thalassiosira_pseudonana_6          -------GYRIYCKGASEVLVVRCNQHLV-KSGND--TSEELNNETRQDILNVAEM---------YA-RRGMRTLALAYRD---LPSGSD----------
Phaeodactylum_tricornutum_5         -------GYRLYCKGAPEVLLARSKSYIN-ALSEV----VDMTEDFRREFLSVSEG---------YA-RRGMRCLALSYRD---VPMEFDIEGKS-----
Phaeodactylum_tricornutum_6         -------GYRLYCKGAPEVLMSRCVLFVN-ASGED----ETLTEHVLGEFQEVSVG---------YG-RRGMRCLALAYRD---IPAGFDLESLS-----
```

```
                                 1101
Skeletonema_marinoi_ABC          ---------ADNDTRHPLL-SDPQNYAS--IESGLTWVGMVGIKDPARPEVAESINKCHAAGVRVIMITGDARDTAVAIARDVNIL-PPS----------
Thalassiosira_pseudonana_1       ---------TDDCARHPLL-SDPQNYAK--IESGLTWVGMAGIKDPARPEVADSIIKCHGAGIRVIMITGDARDTAVAIARDVNIL-PPA----------
Thalassiosira_oceanica_1         --------DSSEDEQHPLL-SDPQNYAG--IENGLTWCGMVGIKDPARPEVANAIKKCHDAGVRVIMITGDARDTAVAIARDVNIL-PPA----------
Phaeodactylum_tricornutum_1      --------NDRAVSRHPLL-SDPTNYRS--VESGLTLVGIVGIKDPARPEVAESMKQCTRAGIRVMMITGDAKDTAIAIARDVNIF-SPV----------
Fragilariopsis_cylindrus_1       --------D--DIRNHPLL-KDPNKYED--IESGLTLVGIVGIKDPARPEVADSILKCTQAGIRVMMITGDARDTAIAIAKDVNIF-NPL----------
Fragilariopsis_cylindrus_2       --------D--DIRNHPLL-KDPNKYED--IESGLTLVGIVGIKDPARPEVADSILKCTQAGIRVMMITGDARDTAIAIAKDVNIF-NPL----------
Fragilariopsis_cylindrus_3       --------D--DIRNHPLL-KDPNKYED--IESGLTLVGIVGIKDPARPEVADSILKCTQAGIRVMMITGDARDTAIAIAKDVNIF-NPL----------
Fragilariopsis_cylindrus_4       --------D--DIRNHPLL-KDPNKYED--IESGLTLVGIVGIKDPARPEVADSILKCTQAGIRVMMITGDARDTAIAIAKDVNIF-NPL----------
Fragilariopsis_cylindrus_5       --------D--DIRNHPLL-KDPNKYED--IESGLTLVGIVGIKDPARPEVADSILKCTQAGIRVMMITGDARDTAIAIAKDVNIF-NPL----------
Fragilariopsis_cylindrus_6       --------D--DIRNHPLL-KDPNKYED--IESGLTLVGIVGIKDPARPEVADSILKCTQAGIRVMMITGDARDTAIAIAKDVNIF-NPL----------
Fragilariopsis_cylindrus_7       --------D--DIRNHPLL-KDPNKYED--IESGLTLVGIVGIKDPARPEVADSILKCTQAGIRVMMITGDARDTAIAIAKDVNIF-NPL----------
Fragilariopsis_cylindrus_8       --------D--DIRNHPLL-KDPNKYED--IESGLTLVGIVGIKDPARPEVADSILKCTQAGIRVMMITGDARDTAIAIAKDVNIF-NPL----------
Pseudo-nitzschia_multiseries_1   --------DDNDVRKHPLL-KDPSKYQD--IESGLTLVGLVGIKDPARPEVADSIKKCTDAGIRVIMITGDARDTAVAIAKDVNIF-DKDA---------
Thalassiosira_pseudonana_2       ----------ENHQYNDYL-KDSSIFEV--IESDLVFVGITAIRDPPRDGVAESIDLCKQAGIRVVMITGDSKSTSVAIAKDVHIF-KENH---------
Ostreococcus_lucimarinus_1       ----------EKHKAHKVL-KDPSAYAS--IESDLIFVGMTGLRDPPRPEVAGAIKACHTAGIRVIVITGDNKLTAEAICTEIGVF-KS-----------
Zea_mays_1                       ----------EEHAAHKYL-LDPSYYSS--IESNMIFCGFVGLRDPPREEVHKAIEDCRAAGIRVMVITGDNKETAEAICREIGVF-GP-----------
Oryza_sativa_1                   ----------EEHAAHKYL-LDPSYYSS--IESNLIFCGFVGLRDPPREEVHKAIEDCRAAGIRVMVITGDNKETAEAICREIGVF-GS-----------
Aquilegia_caerulea_1             ----------EEHPAHDLL-LKPSNYSS--IESDLIFVGFVGLRDPPRAEVYKAIEDCRAAGIRVMVITGDNKNTAEAICREIGVF-GP-----------
A.thaliana_AT1G07810.1           ----------EDHPAHQQL-LNPSNYSS--IESNLIFVGFVGLRDPPRKEVRQAIADCRTAGIRVMVITGDNKSTAEAICREIGVF-EA-----------
A.thaliana_AT1G07670.1           ----------EDHPAHQQL-LNPSNYSS--IESNLVFVGFVGLRDPPRKEVRQAIADCRTAGIRVMVITGDNKSTAEAICREIGVF-EA-----------
Medicago_truncatula_1            ----------EDHPAHQLL-LDPNNYSS--IEDELIFVGLVGLRDPPREEVYQAIEDCRAAGIRVMVITGDNKNTAEAICREIGVF-AP-----------
Glycine_max_1                    ----------DDHPAHQLM-LNPSNYSS--IESELIFVGLVGLRDPPREEVYQAIEDCRDAGIRVMVITGDNKNTAEAICREIGVF-SP-----------
Glycine_max_2                    ----------EDHPAHQLL-LNPSNYSS--IESELIFVGLVGLRDPPREEVYQAIEDCREAGIRVMVITGDNKNTAEAICREIGVF-SP-----------
Aquilegia_caerulea_2             ----------EDHPAHDLL-LNPANYSN--IESGLTFVGLVGLRDPPREEVHKAIEDCREAGIRVMVITGDNKHTAEAICREIGVF-GP-----------
Selaginella_moellendorffii_1     ----------ATHPAHVVL-QDPANYPD--IESDLIFVGMVGLRDPPRGEVKAAIEDCKEAGIQVMVITGDNKKTAEAICREIGIF-YS-----------
Physcomitella_patens_1           ----------DHHPGHARL-LDTSNYDK--IESDLIFVGMAGIRDPPREEVRGAIEDCNEAGIRVMVITGDNKNTAEAICSEIGIF-KD-----------
Physcomitella_patens_2           ----------ENHPGHGRL-LDTENYEK--IESNLIFVGMVGIRDPPRQEVRGAIEDCCEAGVRVMVITGDNKNTAEAICREIGIF-ND-----------
Physcomitella_patens_3           ----------ESHSAHKLL-LDPMNYDD--IESRLIFVGMAGLRDPPREEVHSAIQDCSEAGIRVIVITGDNKNTAESICREIGVF-SK-----------
Selaginella_moellendorffii_2     ----------ESHPAHKKL-LDPSNYSA--IESDLVYCGMVGLRDPPRDEVKSAMDDCREAGIRVMVITGDNKNTAEAICREIGVF-YN-----------
Glycine_max_3                    ----------DTHPAHKKL-LDPTYYSS--IESDLVFVGIVGLRDPPREEVHKAIEDCKEAGIRVMVITGDNKSTAEAICREIKLF-SK-----------
Glycine_max_4                    ----------DTHPAHKKL-LDPTHYSS--IESDLVFVGIIGLRDPPREEVHKAIEDCKEAGIRVMVITGDNKSTAEAICREIKLF-SK-----------
Aquilegia_caerulea_3             ----------DSHPAHKKL-LDPANYSA--IESNLVFVGVVGLRDPPRAEVHEAIEACRDAGIKVMVITGDNKSTAETICQEIGLF-CG-----------
A.thaliana_AT4G00900.1           ----------EEHPSHKKL-LDPSSYSN--IETNLIFVGVVGLRDPPREEVGRAIEDCRDAGIRVMVITGDNKSTAEAICCEIRLF-SE-----------
Chlamydomonas_reinhardtii_1      ---------AEHSPAGRLL-RDPATYAA--VESELVLVGLTGLQDPPRPEVRPAIESCKAAGIRVMVITGDNKDTAEAICGKIGVF-EA-----------
Volvox_carteri_1                 ----------SHHPAMKLL-RDPATYAS--VESDLVLVGLAGLQDPPRPEVRPAIENCKQAGIRVMVITGDNKDTAEAICTKIGVF-QP-----------
Micromonas_pusilla_1             ----------TTHAAHASL-ADASGYAA--IESDLVFVGLAGLRDPPRPEVRGAVAACASAGIRVVVITGDNRLTAEAICVDIGVF-DS-----------
Micromonas_sp_1                  ----------EQHPAHKDL-MDPGKYES--IESDLTFCGLAGLRDPPRPEVRGAIDACKTAGIRVVVITGDNKLTAEAICADIGIF-DS-----------
Selaginella_moellendorffii_3     ----------PDHPAQNIL-VKPESYVL--VESQLTFVGLAGLQDPPRPEVKEAIDDCKRAGIRVIVITGDNKNTAEAICCEIGLF-SS-----------
Thalassiosira_oceanica_2         ----------PGHPATKILGQDTSAFAE--IESGLTFVGLVGIIDPPREEIAPMVQQCKTAGIRIMMITGDNKLTAEAIATEIGIL-DE-----------
Thalassiosira_pseudonana_3       ----------REHKATAILSQDTSTFSS--IESELTFVGLVGIIDPPREEIAPMVKICKTAGIRIMMITGDNKLTAEAIAVDIGIL-DK-----------
Pseudo-nitzschia_multiseries_2   ----------PKEELPSQL-LNLNEYAQ--VESGLTFVGLTGIKDPPRPEVRSAIKRCQDAGIRVIMITGDNKTTAEAIARDVGIF-HE-----------
Fragilariopsis_cylindrus_9       ----------SKEKLPSEL-KNINEYEA--VESELTFIGLTGIKDPPRPEVRGAIQRCQDAGIRVIMITGDNKVTAEAIARDVGIF-TK-----------
Fragilariopsis_cylindrus_10      ----------SKEKLPSEL-KNINEYEA--VESELTFIGLTGIKDPPRPEVRGAIQRCQDAGIRVIMITGDNKVTAEAIARDVGIF-TK-----------
Aquilegia_caerulea_4             ---------------------------E--DEKDLTFIGLVGMLDPPRDEVRSAMLSCMTAGIRVIVVTGDNKSTAESLCRKIGAF-DH-----------
Glycine_max_5                    ---------------------------D--DEKDLTFIGLVGMLDPPRDEVRNAMLSCMTAGIRVIVVTGDNKSTAESLCRKIGAF-DQ-----------
Medicago_truncatula_2            ---------------------------D--DEKDLTFIGLVGMLDPPRDEVRNAMLSCMTAGIRVIVVTGDNKSTAESLCRKIGAF-DH-----------
A.thaliana_AT1G10130.1           ---------------------------D--NENDLTFIGLVGMLDPPREEVRDAMLACMTAGIRVIVVTGDNKSTAESLCRKIGAF-DN-----------
Oryza_sativa_2                   ---------------------------D--DEANLTFIGLVGMLDPPREEVRNAIHSCMSAGIRVIVVTGDNKSTAESLCRQIGAF-EH-----------
Selaginella_moellendorffii_4     ---------------------------D--DENGLILIGMVGMMDPPRPEVKAAIATCKSAGIRVVVITGDNKKTAEALCHRIGVF-DK-----------
Micromonas_pusilla_2             ---------------------------D--DERDLTFLGFVGMLDPPRAEVARAISLCRQAGVRVVMVTGDNRSTAEAIAKRVGLG-DDD---GGRGSHP
Micromonas_sp_2                  ---------------------------S--DETGLTFLGFCGMLDPPRPEVKRAVDVCRGAGIRVVMVTGDNKLTAEAIAKQIGLD-DY----GG-----
Volvox_carteri_2                 ---------------------------A--DESGLTFLGLVAMHDPPRNECSRALQLCQQAGIRVVMVTGDNKATAEAVARQVGLL-PRE---SGSA---
Chlamydomonas_reinhardtii_2      ---------------------------A--DEAGLTFLGLVGMHDPPRPEAARALELCRQAGIRVVMVTGDNKSTAEAVARQVGML-PPLSRGGGAAS--
Dunaliella_salina_1              ---------------------------N--DESGLTFIGLLGMHDPPRPECRSALSTCHNAGIKVIMVTGDNKGTAEAVARQVGAL-SPSTALAGS----
Ostreococcus_lucimarinus_2       ---------------------------T--DENNLTFIGLVGMIDPPRPEVRYSLQTCKDAGIRVIMVTGDNQQTAEAIASQIGLS-NSIDPLTGG----
Phaeodactylum_tricornutum_2      ------------------------------SLGELVFAGLVGMEDPPREGVCDAVRQLRRGGVKVMMVTGDSKETALAIAQRCGIL-GS-----------
Chlamydomonas_reinhardtii_3      AWLLSGGVNPEAAAAAAAE-GKPPPAPA--AKLQLSMVLLVAILDPPREEAVRAVGVAHKAGITVKMITGDHALTAVAIGQMLGIV-PRAQEMPAAAD--
Chlamydomonas_reinhardtii_4      AWLLSGGVNPEAAAAAAAE-GKRPPAPA--AKLQLSMVLLVAILDPPREEAVRAVGVAHKAGITVKMITGDHALTAVAIGRMLGIV-PA-----------
Thalassiosira_pseudonana_4       --------------------LGQEFVNGRPEGKWLTMVGLCAIMDPPRPECVQAIKEAHGAGVRVAMITGDHKDTATAIGHMLGIV-DETY---------
Thalassiosira_pseudonana_5       --------------------LGQEFVNGRPERKWLTMVGLCAIMDPPRPECVQAIREAHGAGVRVAMITGDHKDTATAIGHMLGIV-DEKY---------
```

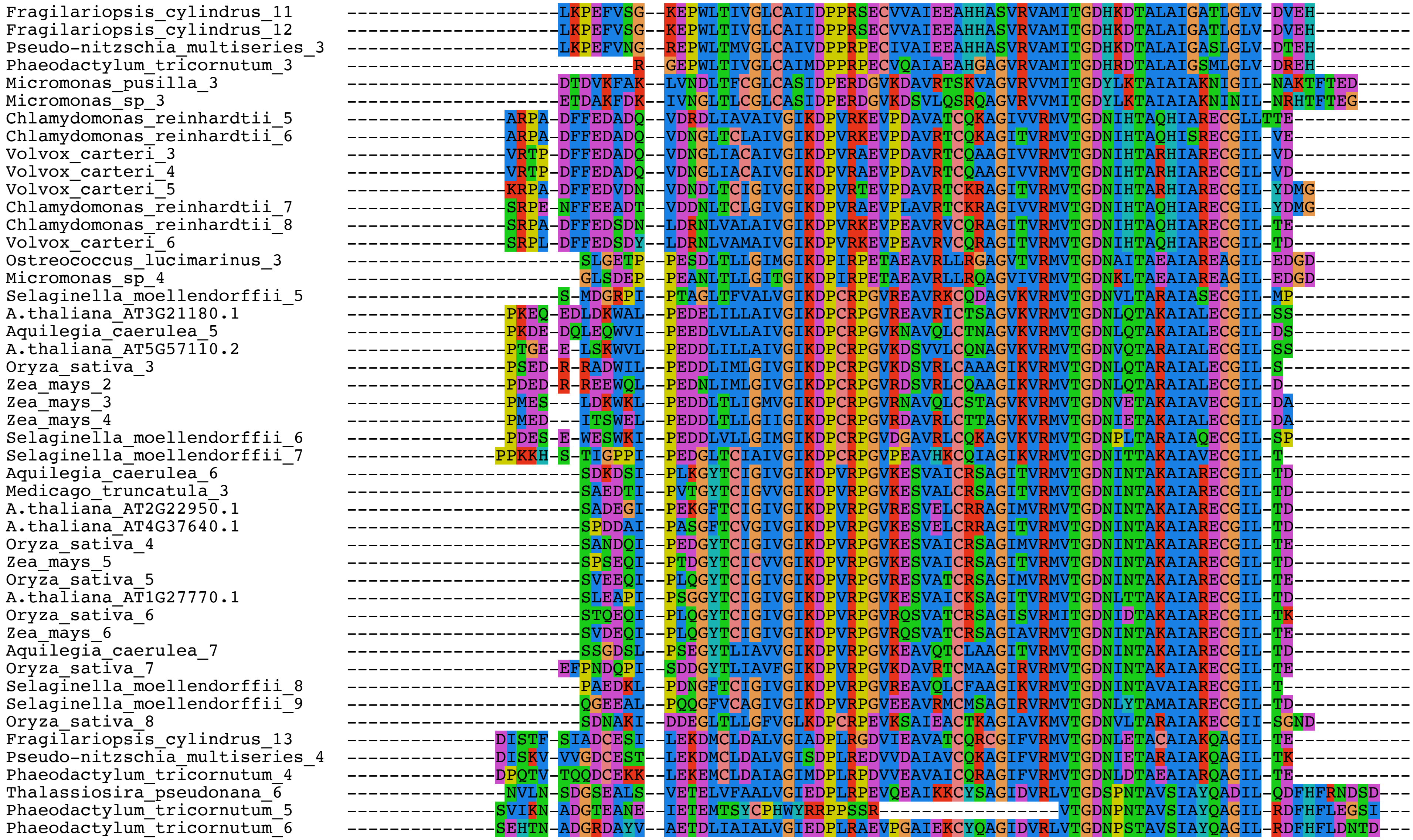
Fragilariopsis_cylindrus_11
Fragilariopsis_cylindrus_12
Pseudo-nitzschia_multiseries_3
Phaeodactylum_tricornutum_3
Micromonas_pusilla_3
Micromonas_sp_3
Chlamydomonas_reinhardtii_5
Chlamydomonas_reinhardtii_6
Volvox_carteri_3
Volvox_carteri_4
Volvox_carteri_5
Chlamydomonas_reinhardtii_7
Chlamydomonas_reinhardtii_8
Volvox_carteri_6
Ostreococcus_lucimarinus_3
Micromonas_sp_4
Selaginella_moellendorffii_5
A.thaliana_AT3G21180.1
Aquilegia_caerulea_5
A.thaliana_AT5G57110.2
Oryza_sativa_3
Zea_mays_2
Zea_mays_3
Zea_mays_4
Selaginella_moellendorffii_6
Selaginella_moellendorffii_7
Aquilegia_caerulea_6
Medicago_truncatula_3
A.thaliana_AT2G22950.1
A.thaliana_AT4G37640.1
Oryza_sativa_4
Zea_mays_5
Oryza_sativa_5
A.thaliana_AT1G27770.1
Oryza_sativa_6
Zea_mays_6
Aquilegia_caerulea_7
Oryza_sativa_7
Selaginella_moellendorffii_8
Selaginella_moellendorffii_9
Oryza_sativa_8
Fragilariopsis_cylindrus_13
Pseudo-nitzschia_multiseries_4
Phaeodactylum_tricornutum_4
Thalassiosira_pseudonana_6
Phaeodactylum_tricornutum_5
Phaeodactylum_tricornutum_6

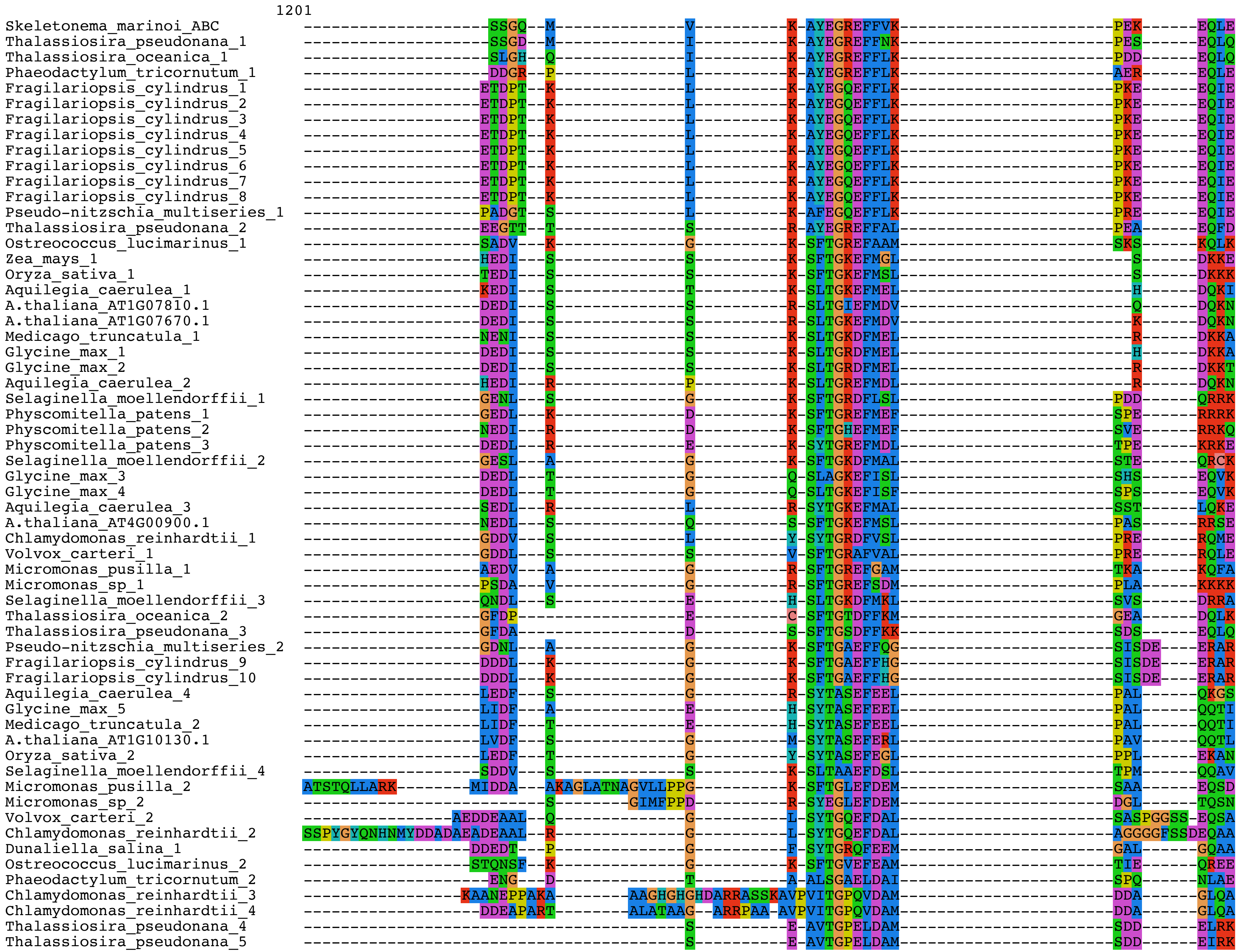
1201
Skeletonema_marinoi_ABC
Thalassiosira_pseudonana_1
Thalassiosira_oceanica_1
Phaeodactylum_tricornutum_1
Fragilariopsis_cylindrus_1
Fragilariopsis_cylindrus_2
Fragilariopsis_cylindrus_3
Fragilariopsis_cylindrus_4
Fragilariopsis_cylindrus_5
Fragilariopsis_cylindrus_6
Fragilariopsis_cylindrus_7
Fragilariopsis_cylindrus_8
Pseudo-nitzschia_multiseries_1
Thalassiosira_pseudonana_2
Ostreococcus_lucimarinus_1
Zea_mays_1
Oryza_sativa_1
Aquilegia_caerulea_1
A.thaliana_AT1G07810.1
A.thaliana_AT1G07670.1
Medicago_truncatula_1
Glycine_max_1
Glycine_max_2
Aquilegia_caerulea_2
Selaginella_moellendorffii_1
Physcomitella_patens_1
Physcomitella_patens_2
Physcomitella_patens_3
Selaginella_moellendorffii_2
Glycine_max_3
Glycine_max_4
Aquilegia_caerulea_3
A.thaliana_AT4G00900.1
Chlamydomonas_reinhardtii_1
Volvox_carteri_1
Micromonas_pusilla_1
Micromonas_sp_1
Selaginella_moellendorffii_3
Thalassiosira_oceanica_2
Thalassiosira_pseudonana_3
Pseudo-nitzschia_multiseries_2
Fragilariopsis_cylindrus_9
Fragilariopsis_cylindrus_10
Aquilegia_caerulea_4
Glycine_max_5
Medicago_truncatula_2
A.thaliana_AT1G10130.1
Oryza_sativa_2
Selaginella_moellendorffii_4
Micromonas_pusilla_2
Micromonas_sp_2
Volvox_carteri_2
Chlamydomonas_reinhardtii_2
Dunaliella_salina_1
Ostreococcus_lucimarinus_2
Phaeodactylum_tricornutum_2
Chlamydomonas_reinhardtii_3
Chlamydomonas_reinhardtii_4
Thalassiosira_pseudonana_4
Thalassiosira_pseudonana_5

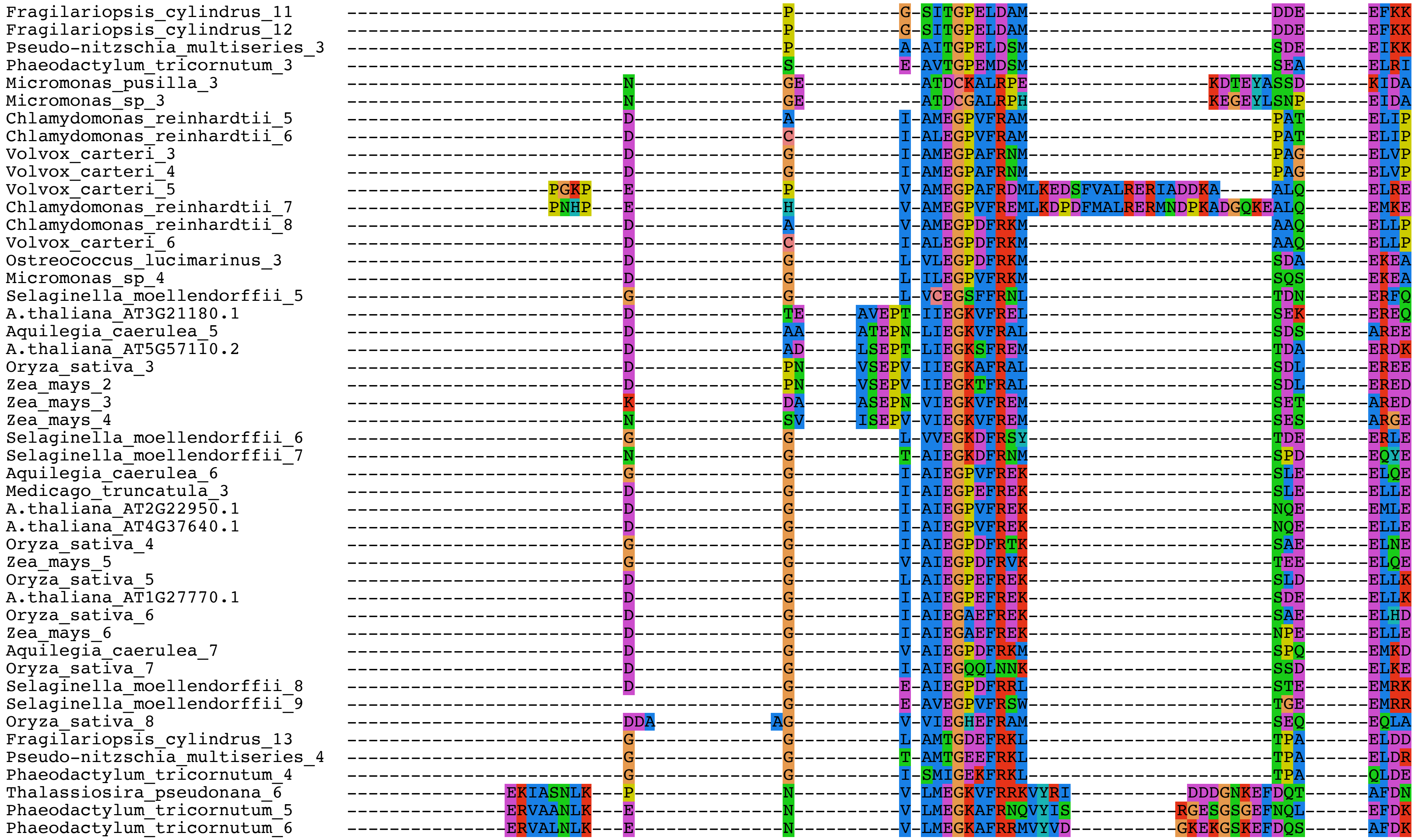
Fragilariopsis_cylindrus_11 P G SITGPELDAM DDE EFKK
Fragilariopsis_cylindrus_12 P G SITGPELDAM DDE EFKK
Pseudo-nitzschia_multiseries_3 P A AITGPELDSM SDE EIKK
Phaeodactylum_tricornutum_3 S E AVTGPEMDSM SEA ELRI
Micromonas_pusilla_3 N GE ATDCKALRPE KDTEYASSD KIDA
Micromonas_sp_3 N GE ATDCGALRPH KEGEYLSNP EIDA
Chlamydomonas_reinhardtii_5 D A I AMEGPVFRAM PAT ELIP
Chlamydomonas_reinhardtii_6 D C I ALEGPVFRAM PAT ELIP
Volvox_carteri_3 D G I AMEGPAFRNM PAG ELVP
Volvox_carteri_4 D G I AMEGPAFRNM PAG ELVP
Volvox_carteri_5 PGKP E P V AMEGPAFRDMLKEDSFVALRERIADDKA ALQ ELRE
Chlamydomonas_reinhardtii_7 PNHP E H V AMEGPVFREMLKDPDFMALRERMNDPKADGQKEALQ EMKE
Chlamydomonas_reinhardtii_8 D A V AMEGPDFRKM AAQ ELLP
Volvox_carteri_6 D C I ALEGPDFRKM AAQ ELLP
Ostreococcus_lucimarinus_3 D G L VLEGPDFRKM SDA EKEA
Micromonas_sp_4 D G L ILEGPVFRKM SQS EKEA
Selaginella_moellendorffii_5 G G L VCEGSFFRNL TDN ERFQ
A.thaliana_AT3G21180.1 D TE AVEPT IIEGKVFREL SEK ERFQ
Aquilegia_caerulea_5 D AA ATEPN LIEGKVFRAL SDS AREE
A.thaliana_AT5G57110.2 D AD LSEPT LIEGKSFREM TDA ERDK
Oryza_sativa_3 D PN VSEPV IIEGKAFRAL SDL EREE
Zea_mays_2 D PN VSEPV IIEGKTFRAL SDL ERED
Zea_mays_3 K DA ASEPN VIEGKVFREM SET ARED
Zea_mays_4 N SV ISEPV VIEGKVFREM SES ARGE
Selaginella_moellendorffii_6 G G L VVEGKDFRSY TDE ERLE
Selaginella_moellendorffii_7 N G T AIEGKDFRNM SPD EQYE
Aquilegia_caerulea_6 G G I AIEGPVFREK SLE ELQE
Medicago_truncatula_3 D G I AIEGPEFREK SLE ELLE
A.thaliana_AT2G22950.1 D G I AIEGPVFREK NQE EMLE
A.thaliana_AT4G37640.1 D G I AIEGPVFREK NQE ELLE
Oryza_sativa_4 G G I AIEGPDFRTK SAE ELNE
Zea_mays_5 G G V AIEGPDFRVK TEE ELQE
Oryza_sativa_5 D G L AIEGPEFREK SLD ELLK
A.thaliana_AT1G27770.1 D G I AIEGPEFREK SDE ELLK
Oryza_sativa_6 D G I AIEGAEFREK SAE ELHD
Zea_mays_6 D G I AIEGAEFREK NPE ELLE
Aquilegia_caerulea_7 D G V AIEGPDFRKM SPQ EMKD
Oryza_sativa_7 D G I AIEGQQLNNK SSD ELKE
Selaginella_moellendorffii_8 D G E AIEGPDFRRL STE EMRK
Selaginella_moellendorffii_9 G E AVEGPVFRSW TGE EMRR
Oryza_sativa_8 DDA AG V VIEGHEFRAM SEQ EQLA
Fragilariopsis_cylindrus_13 G G L AMTGDEFRKL TPA ELDD
Pseudo-nitzschia_multiseries_4 G G T AMTGEEFRKL TPA ELDR
Phaeodactylum_tricornutum_4 G G I SMIGEKFRKL TPA QLDE
Thalassiosira_pseudonana_6 EKIASNLK P N V LMEGKVFRRKVYRI DDDGNKEFDQT AFDN
Phaeodactylum_tricornutum_5 ERVAANLK E N V LMEGKAFRNQVYIS RGESGSGEFNQL EFDK
Phaeodactylum_tricornutum_6 ERVALNLK E N V LMEGKAFRRMVYVD GKEKGSKEFDQS AFDK

```
                                 1301
Skeletonema_marinoi_ABC          LL----AT--GNMIFC------RAEPSDKQRLIK---------MLQ----------------------------------------S--Q----------
Thalassiosira_pseudonana_1       LL----ASP-GNMVFC------RAEPSDKQRLIK---------MLQ----------------------------------------S--L----------
Thalassiosira_oceanica_1         LL----KSP-GNMVFC------RAEPADKQKLIK---------MLQ----------------------------------------S--L----------
Phaeodactylum_tricornutum_1      IL----RE--GNIVFC------RAEPADKQKLVK---------MLQ----------------------------------------N--L----------
Fragilariopsis_cylindrus_1       IL----KS--DNIVFC------RAQPSDKQKLIK---------MLQ----------------------------------------SKEL----------
Fragilariopsis_cylindrus_2       IL----KS--DNIVFC------RAQPSDKQKLIK---------MLQ----------------------------------------SKEL----------
Fragilariopsis_cylindrus_3       IL----KS--DNIVFC------RAQPSDKQKLIK---------MLQ----------------------------------------SKEL----------
Fragilariopsis_cylindrus_4       IL----KS--DNIVFC------RAQPSDKQKLIK---------MLQ----------------------------------------SKEL----------
Fragilariopsis_cylindrus_5       IL----KS--DNIVFC------RAQPSDKQKLIK---------MLQ----------------------------------------SKEL----------
Fragilariopsis_cylindrus_6       IL----KS--DNIVFC------RAQPSDKQKLIK---------MLQ----------------------------------------SKEL----------
Fragilariopsis_cylindrus_7       IL----KS--DNIVFC------RAQPSDKQKLIK---------MLQ----------------------------------------SKEL----------
Fragilariopsis_cylindrus_8       IL----KS--DNIVFC------RAQPSDKQKLIK---------MLQ----------------------------------------SKEL----------
Pseudo-nitzschia_multiseries_1   IL----SK--DNIVFC------RAQPSDKQALIK---------MLQ----------------------------------------N-DL----------
Thalassiosira_pseudonana_2       VL----KS--GNLVIC------RAEPSDKQRLVK---------MLQ----------------------------------------S--I----------
Ostreococcus_lucimarinus_1       AL----LGE-GGRVFS------RTEPKHKQDIVR---------LLR----------------------------------------D--S----------
Zea_mays_1                       LL----RQQ-GGLLFS------RAEPKHKQEIVR---------LLK----------------------------------------E--D----------
Oryza_sativa_1                   LL----RQT-GGLLFS------RAEPKHKQEIVR---------LLK----------------------------------------E--D----------
Aquilegia_caerulea_1             HL----RQS-GGLLFS------RAEPRHKQEIVR---------LLK----------------------------------------E--D----------
A.thaliana_AT1G07810.1           HL----RQT-GGLLFS------RAEPKHKQEIVR---------LLK----------------------------------------E--D----------
A.thaliana_AT1G07670.1           HL----RQT-GGLLFS------RAEPKHKQEIVR---------LLK----------------------------------------E--D----------
Medicago_truncatula_1            YL----RQT-GGLLFS------RAEPRHKQDIVR---------LLK----------------------------------------E--D----------
Glycine_max_1                    YL----RQH-GGLLFS------RAEPRHKQEIVR---------LLK----------------------------------------E--E----------
Glycine_max_2                    YL----RQP-GGLLFS------RAEPRHKQEIVR---------LLK----------------------------------------E--E----------
Aquilegia_caerulea_2             HL----RQS-GGLLFS------RAEPRHKQEIVR---------LLK----------------------------------------E--D----------
Selaginella_moellendorffii_1     VL----SGK-GGRVFS------RAEPRHKQEIVR---------MLK----------------------------------------E--A----------
Physcomitella_patens_1           IL----SGT-GGRVFS------RAEPKHKQDIVR---------ILK----------------------------------------E--A----------
Physcomitella_patens_2           IL----SGT-GGRVFS------RAEPKHKQDIVR---------ILK----------------------------------------D--A----------
Physcomitella_patens_3           IL----FGK-GGRVFS------RAEPKHKQDIVR---------ILK----------------------------------------D--G----------
Selaginella_moellendorffii_2     VL----SGS-GGRVFS------RAEPRHKQEIVR---------MLK----------------------------------------D--A----------
Glycine_max_3                    IL----LRP-GGKVFS------RAEPRHKQEIVR---------LLK----------------------------------------E--M----------
Glycine_max_4                    IL----LRP-GGKVFS------RAEPRHKQEIVR---------LLK----------------------------------------E--M----------
Aquilegia_caerulea_3             IL----SKP-GGMVFS------RAEPKHKQEIVR---------MLK----------------------------------------E--M----------
A.thaliana_AT4G00900.1           IL----SKS-GGKVFS------RAEPRHKQEIVR---------MLK----------------------------------------E--M----------
Chlamydomonas_reinhardtii_1      IL----ASA-PAMCFS------RAEPRHKQDIVR---------LLK----------------------------------------E--Q----------
Volvox_carteri_1                 LL----AAA-PAMCFS------RAEPRHKQDIVR---------LLK----------------------------------------E--Q----------
Micromonas_pusilla_1             AL----TAP-GGCVCS------RAEPKHKQDIVR---------LLK----------------------------------------E--R----------
Micromonas_sp_1                  LL----ATP-GGCVFS------RAEPKHKQDIVR---------LLK----------------------------------------E--A----------
Selaginella_moellendorffii_3     LLLGNQSDS-KGFVFS------RSEPIHKQEIVR---------VLK----------------------------------------D--G----------
Thalassiosira_oceanica_2         IL----AGD-GGLVFS------RTEPTHKQQLVK---------LLK----------------------------------------S--Q----------
Thalassiosira_pseudonana_3       IL----MKDNGGLVFS------RTEPRHKQQLVK---------LLK----------------------------------------S--Q----------
Pseudo-nitzschia_multiseries_2   LIM---QGG-GSRVFS------RTEPRDKQLLVK---------ILR----------------------------------------S--C----------
Fragilariopsis_cylindrus_9       LIM---VGG-GSRVFS------RTEPRDKQLLVK---------LLR----------------------------------------N--C----------
Fragilariopsis_cylindrus_10      LIM---VGG-GSRVFS------RTEPRDKQLLVK---------LLR----------------------------------------N--C----------
Aquilegia_caerulea_4             AL----Q---HMVLFT------RVEPSHKRMLVE---------ALQ----------------------------------------Q--Q----------
Glycine_max_5                    AL----Q---RMALFT------RVEPSHKRMLVE---------ALQ----------------------------------------H--Q----------
Medicago_truncatula_2            AL----Q---RMALFT------RVEPSHKRMLVE---------ALQ----------------------------------------H--Q----------
A.thaliana_AT1G10130.1           AL----R---RMTLFS------RVEPSHKRMLVE---------ALQ----------------------------------------K--Q----------
Oryza_sativa_2                   AL----Q---RMVLFSSFTGCCRVEPSHKRMLVE---------ALQ----------------------------------------L--H----------
Selaginella_moellendorffii_4     AV----Q---NLVLFS------RVNPSHKSMLVD---------VLK----------------------------------------R--H----------
Micromonas_pusilla_2             AV----A---NMAVFS------RVEPRHKSKLIE---------ILK----------------------------------------R--Q----------
Micromonas_sp_2                  AA----L---SMSVFS------RVEPLHKTRLVE---------LLK----------------------------------------A--H----------
Volvox_carteri_2                 AV----S---RLAVMS------RVEPMHKLRLVE---------LLR----------------------------------------S--Q----------
Chlamydomonas_reinhardtii_2      AV----D---NVVVLS------RVEPMHKLRLVE---------QLR----------------------------------------A--R----------
Dunaliella_salina_1              AT----R---NLVVLS------RVEPMHKLRLVE---------LLK----------------------------------------A--Q----------
Ostreococcus_lucimarinus_2       AA----R---TMCVFS------RVEPAQKSKLVE---------ILK----------------------------------------R--Q----------
Phaeodactylum_tricornutum_2      SI----S---GVRVFY------RVAPRHKLAIVR---------ALQ----------------------------------------E--R----------
Chlamydomonas_reinhardtii_3      VV----M---GCNVFA------RASPENKLRIVR---------ALQ----------------------------------------A--L----------
Chlamydomonas_reinhardtii_4      VV----M---GCNVFA------RASPENKLRIVK---------ALQ----------------------------------------A--L----------
Thalassiosira_pseudonana_4       CV----M---THNVFA------RASPQNKIRIVK---------ALQ----------------------------------------A--E----------
Thalassiosira_pseudonana_5       CV----L---THNVFA------RASPQNKIRIVK---------ALQ----------------------------------------A--E----------
```

```
Fragilariopsis_cylindrus_11        AV----M---TYNVFA------RASPENKIKIVK---------ALQ----------------------------------------S--Q----------
Fragilariopsis_cylindrus_12        AV----M---TYNVFA------RASPENKIKIVK---------ALQ----------------------------------------S--Q----------
Pseudo-nitzschia_multiseries_3     AV----M---TYNVFA------RASPENKIKIVK---------ALQ----------------------------------------S--Q----------
Phaeodactylum_tricornutum_3        AT----Q---KYNVFA------RASPQNKIRIVK---------ALQ----------------------------------------A--E----------
Micromonas_pusilla_3               LT----R---TTSVFA------RAKPEDKLEIVK---------SLQ----------------------------------------R--Q----------
Micromonas_sp_3                    LT----K---TTSVFA------RAKPEDKLEIVK---------SLQ----------------------------------------R--Q----------
Chlamydomonas_reinhardtii_5        LL----P---RLRVLA------RSSPEDKLTLVS---------LLK----------------------------------------K--Q----------
Chlamydomonas_reinhardtii_6        LL----P---RLRVLA------RSSPEDKLTLVA---------LLK----------------------------------------K--Q----------
Volvox_carteri_3                   LL----P---KLRVLA------RSSPEDKLTLVS---------LLK----------------------------------------K--Q----------
Volvox_carteri_4                   LL----P---KLRVLA------RSSPEDKLTLVS---------LLK----------------------------------------K--Q----------
Volvox_carteri_5                   KM----D---AVRV--------RPGRGGRLKVVRGEKNTTYSWLAS----------------------------------------Q--A----------
Chlamydomonas_reinhardtii_7        KI----N---HVRVLA------RSSPEDKLQLVR---------LLK----------------------------------------E--M----------
Chlamydomonas_reinhardtii_8        LL----P---KLRVLA------RSSPEDKLTLVS---------MLK----------------------------------------Q--H----------
Volvox_carteri_6                   LL----P---KLRVLA------RSSPEDKLTLVS---------MLK----------------------------------------Q--Q----------
Ostreococcus_lucimarinus_3         IA----M---RIRVLA------RSSPSDKLVLCN---------LQR----------------------------------------K--L----------
Micromonas_sp_4                    VA----V---KIRVLA------RSSPADKLMLCN---------LQK----------------------------------------S--L----------
Selaginella_moellendorffii_5       IV----P---KIDVLA------RSTPSDKLLLVK---------TLK----------------------------------------S--L----------
A.thaliana_AT3G21180.1             VA----K---KITVMG------RSSPNDKLLLVQ---------ALR----------------------------------------K--N----------
Aquilegia_caerulea_5               IA----D---KISVMG------RSSPNDKLLLVK---------ALK----------------------------------------R--R----------
A.thaliana_AT5G57110.2             IS----D---KISVMG------RSSPNDKLLLVQ---------SLR----------------------------------------R--Q----------
Oryza_sativa_3                     AA----E---KISVMG------RSSPNDKLLLVK---------ALR----------------------------------------K--R----------
Zea_mays_2                         AA----E---KISVMG------RSSPNDKLLLVK---------ALR----------------------------------------A--R----------
Zea_mays_3                         IA----D---KITVMG------RSSPNDKLLLVQ---------CLK----------------------------------------R--R----------
Zea_mays_4                         AA----D---KIIVMG------RSSPNDKLLLVQ---------ALK----------------------------------------R--K----------
Selaginella_moellendorffii_6       LV----P---KLEVMA------RSSPMDKLLLVK---------TLR----------------------------------------S--M----------
Selaginella_moellendorffii_7       IL----P---AIQVMA------RSSPTDKHTMVK---------RLL----------------------------------------E--M----------
Aquilegia_caerulea_6               II----P---KLQVMA------RSSPLDKHTLVK---------HLR----------------------------------------T--TF---------
Medicago_truncatula_3              LI----P---KIQVMA------RSSPLDKHTLVR---------HLR----------------------------------------T--TF---------
A.thaliana_AT2G22950.1             LI----P---KIQVMA------RSSPMDKHTLVK---------QLR----------------------------------------T--TF---------
A.thaliana_AT4G37640.1             LI----P---KIQVMA------RSSPMDKHTLVK---------QLR----------------------------------------T--TF---------
Oryza_sativa_4                     LI----P---KIQVMA------RSSPLDKHTLVK---------HLR----------------------------------------T--TF---------
Zea_mays_5                         LI----P---KIQVMA------RSSPLDKHTLVK---------HLR----------------------------------------T--TF---------
Oryza_sativa_5                     LI----P---KIQVMA------RSSPLDKHTLVK---------HLR----------------------------------------T--TF---------
A.thaliana_AT1G27770.1             LI----P---KLQVMA------RSSPMDKHTLVR---------LLR----------------------------------------T--MF---------
Oryza_sativa_6                     LI----P---KMQVLA------RSSPLDKHTLVK---------HLR----------------------------------------T--AF---------
Zea_mays_6                         LI----P---KMQVLA------RSSPLDKHALVK---------YLR----------------------------------------T--TF---------
Aquilegia_caerulea_7               LV----P---KIQVMA------RSMPMDKHTLVT---------HLR----------------------------------------T--VL---------
Oryza_sativa_7                     LL----P---KIQVIA------RSLPMDKYKLVT---------SLK----------------------------------------S--MY---------
Selaginella_moellendorffii_8       LI----P---SLQVMA------RSSPTDKHTLVR---------ELR----------------------------------------A--L----------
Selaginella_moellendorffii_9       RI----P---KMQILA------RSSPSDKHRLVK---------ELQ----------------------------------------A--M----------
Oryza_sativa_8                     IV----D---NIRVMA------RSLPLDKLVLVQ---------RLK----------------------------------------Q--K----------
Fragilariopsis_cylindrus_13        IL----P---TLQVLA------RSSPEDKHTLVQ---------RLNG-----------------------------------------------------
Pseudo-nitzschia_multiseries_4     VL----P---TLQVLA------RSSPEDKHTLVQ---------RLNGGLMPETEEEWLERHPGRDFDTEKDLLLPGYKEEWSRSRGG--V----------
Phaeodactylum_tricornutum_4        IL----P---RLQVLA------RSSPEDKHTLVQ---------RLNGAAIPSTESEWCEAHPNKDFATQRNLLLPGYKDEWAKSRFG--V----------
Thalassiosira_pseudonana_6         IW----P---HLRVLA------RSSPDDKLTLAH---------GLN----------------------------------------Q--STL--------
Phaeodactylum_tricornutum_5        IW----P---HLRILA------RSSPDDKLTLAH---------GLN----------------------------------------Q--SNLFVDKGLCQ
Phaeodactylum_tricornutum_6        IW----P---RLRVLA------RSSPDDKLTLAH---------GLN----------------------------------------K-------------
```

```
                                  1401
Skeletonema_marinoi_ABC           --------------GEI---AAMTGD-----GVNDAPALQQ------ASIGIAMGISGTEVSKEAADMVLADDNFSTIVAAIEEGRSIYNNMQAFICFLISC
Thalassiosira_pseudonana_1        --------------GEI---PAMTGD-----GVNDAPALQQ------ASIGVAMGISGTEVSKEAADMVLADDNFSTIVAAVEEGRCIYANMQAFICFLISC
Thalassiosira_oceanica_1          --------------DEI---SAMTGNFYQFHCVSDAPALQQ------ANIGIAMGIAGTEVSKEAADMVLADDNFSTIVAAVEEGRCIYANMQAFICFLISC
Phaeodactylum_tricornutum_1       --------------DEI---PAMTGD-----GVNDAPALQQ------AAIGIAMGITGTEVSKNAADMILADDNFSTIVSAVEEGRRIYSNMQAFICFLISC
Fragilariopsis_cylindrus_1        --------------NEI---PAMTGD-----GVNDAPALQQ------AAIGVAMG-TGTAVAKEAADMILADDDFSTIVSAVEEGRTIYANMQAFICFLISC
Fragilariopsis_cylindrus_2        --------------NEI---PAMTGD-----GVNDAPALQQ------AAIGVAMG-TGTAVAKEAADMILADDDFSTIVSAVEEGRTIYANMQAFICFLISC
Fragilariopsis_cylindrus_3        --------------NEI---PAMTGD-----GVNDAPALQQ------AAIGVAMG-TGTAVAKEAADMILADDDFSTIVSAVEEGRTIYANMQAFICFLISC
Fragilariopsis_cylindrus_4        --------------NEI---PAMTGD-----GVNDAPALQQ------AAIGVAMG-TGTAVAKEAADMILADDDFSTIVSAVEEGRTIYANMQAFICFLISC
Fragilariopsis_cylindrus_5        --------------NEI---PAMTGD-----GVNDAPALQQ------AAIGVAMG-TGTAVAKEAADMILADDDFSTIVSAVEEGRTIYANMQAFICFLISC
Fragilariopsis_cylindrus_6        --------------NEI---PAMTGD-----GVNDAPALQQ------AAIGVAMG-TGTAVAKEAADMILADDDFSTIVSAVEEGRTIYANMQAFICFLISC
Fragilariopsis_cylindrus_7        --------------NEI---PAMTGD-----GVNDAPALQQ------AAIGVAMG-TGTAVAKEAADMILADDDFSTIVSAVEEGRTIYANMQAFICFLISC
Fragilariopsis_cylindrus_8        --------------NEI---PAMTGD-----GVNDAPALQQ------AAIGVAMG-TGTAVAKEAADMILADDDFSTIVSAVEEGRTIYANMQAFICFLISC
Pseudo-nitzschia_multiseries_1    --------------GEI---AAMTGD-----GVNDAPALQQ------AAIGVAMG-TGTAVSKEAADMVLADDDFSTIVSAVEEGRTIYANMQAFICFLISC
Thalassiosira_pseudonana_2        --------------DEI---PAMTGD-----GVNDAPALQQ------ASIGVAMGISGTDVAKEASDMILVDDNFSTIVDAVEEGRCIYANMQAFINFLITC
Ostreococcus_lucimarinus_1        --------------GDV---VAMTGD-----GVNDAPALKL------ADIGIAMGIAGTEVAKEASDMVLADDNFSTIVEAVSEGRSIYNNMKAFIRYMISS
Zea_mays_1                        --------------GEV---VAMTGD-----GVNDAPALKL------ADIGVAMGITGTEVAKEASDMVLADDNFSTIVAAVGEGRSIYNNMKAFIRYMISS
Oryza_sativa_1                    --------------GEV---VAMTGD-----GVNDAPALKL------ADIGVAMGITGTEVAKEASDMVLADDNFSTIVAAVGEGRSIYDNMKAFIRYMISS
Aquilegia_caerulea_1              --------------GEV---VAMTGD-----GVNDAPALKL------ADIGIAMGIAGTEVAKEASDMVLADDNFSTIVAAVGEGRSIYNNMKAFIRYMISS
A.thaliana_AT1G07810.1            --------------GEV---VAMTGD-----GVNDAPALKL------ADIGVAMGISGTEVAKEASDMVLADDNFSTIVAAVGEGRSIYNNMKAFIRYMISS
A.thaliana_AT1G07670.1            --------------GEV---VAMTGD-----GVNDAPALKL------ADIGVAMGISGTEVAKEASDLVLADDNFSTIVAAVGEGRSIYNNMKAFIRYMISS
Medicago_truncatula_1             --------------GEV---VAMTGD-----GVNDAPALKL------ADIGIAMGIAGTEVAKEASDMVLADDNFSSIVAAVGEGRSIYNNMKAFIRYMISS
Glycine_max_1                     --------------GEV---VAMTGD-----GVNDAPALKL------ADIGIAMGIAGTEVAKEASDMVLADDNFSSIVAAVGEGRSIYNNMKAFIRYMISS
Glycine_max_2                     --------------GEV---VAMTGD-----GVNDAPALKL------ADIGIAMGIAGTEVAKEASDMVLADDNFSSIVAAVGEGRSIYNNMKAFIRYMISS
Aquilegia_caerulea_2              --------------GEV---VAMTGD-----GVNDGPALKL------ADIGIAMGIAGTEVAKEASDMVLADDNFSTIVSAVGEGRSIYNNMKAFIRYMISS
Selaginella_moellendorffii_1      --------------GEV---VAMTGD-----GVNDAPALKF------ADIGIAMGITGTEVAKEAADMVLADDNFSTIVSAVAEGRSIYSNMKAFIRYMISS
Physcomitella_patens_1            --------------GEV---VAMTGD-----GVNDAPALKL------ADIGVAMGIAGTEVAKEAADMVLADDNFSTIVAAVGEGRSIYNNMKAFIRYMISS
Physcomitella_patens_2            --------------GEV---VAMTGD-----GINDAPALKL------ADIGVAMGIAGTEVAKEAADMVLADDNFSTIVAAVGEGRSIYNNMKAFIRYMISS
Physcomitella_patens_3            --------------GEV---VAMTGD-----GVNDAPALKL------ADIGVAMGISGTEVAKEAADMVLADDNFSTIVAAVGEGRAIYNNMKAFIRYMISS
Selaginella_moellendorffii_2      --------------GEV---VAMTGD-----GVNDAPALKL------ADIGIAMGITGTEVAKEAADMVLADDNFSTIVSAVAEGRSIYNNMKAFIRYMISS
Glycine_max_3                     --------------GEI---VAMTGD-----GVNDAPALKL------ADIGIAMGITGTEVAKEASDMVLADDNFSTIVLAVAEGRSIYNNMKSFIRYMISS
Glycine_max_4                     --------------GEI---VAMTGD-----GVNDAPALKL------ADIGIAMGITGTEVAKEASDMVLADDNFSTIVSAVAEGRSIYNNMKSFIRYMISS
Aquilegia_caerulea_3              --------------GEI---VAMTGD-----GVNDAPALKL------ADIGIAMGITGTEVAKEASDMVLADDNFSTIVSAVAEGRSIYDNMKAFIRYMISS
A.thaliana_AT4G00900.1            --------------GEI---VAMTGD-----GVNDAPALKL------ADIGIAMGITGTEVAKEASDMVLADDNFSTIVSAVAEGRSIYNNMKAFIRYMISS
Chlamydomonas_reinhardtii_1       --------------GEV---TAMTGD-----GVNDAPALKL------ADIGVAMGIAGTEVAKEASDMVLADDNFSSIVAAVAEGRAIYNNMKAFIRYMISS
Volvox_carteri_1                  --------------GEV---AAMTGD-----GVNDAPALKL------ADIGVAMGIAGTEVAKEASDMVLADDNFSSIVAAVAEGRAIYNNMKAFIRYMISS
Micromonas_pusilla_1              --------------GEI---VAMTGD-----GVNDAPALKL------ADIGIAMGITGTAVAKEASDMVLADDNFSSIVDAISEGRSIYNNMKAFIRYMISS
Micromonas_sp_1                   --------------DEI---VAMTGD-----GVNDAPALKL------ADIGIAMGITGTAVAKEASDMVLADDNFSSIVDAISEGRSIYNNMKAFIRYMISS
Selaginella_moellendorffii_3      --------------GEI---VAMTGD-----GVNDAPALKL------ADIGIAMGLSGTEVAKEASDMVLADDDFATIVVAVREGRSIYDNMRAFIRYLISS
Thalassiosira_oceanica_2          --------------GCV---VAMTGD-----GVNDAPALKQ------ADIGIAMGLTGTEVAKEAADMILADDNFATIVLAVEEGRSIYNNMQAFIRYLISS
Thalassiosira_pseudonana_3        --------------GCV---VAMTGD-----GVNDAPALKQ------ADIGIAMGLTGTEVAKEASDMILADDNFATIVHAVEEGRSIYNNMQAFIRYLISS
Pseudo-nitzschia_multiseries_2    --------------GEV---PAMTGD-----GVNDAPALKQ------ASIGIAMGISGTEVAKEAADMILADDNFATIVSAVEEGRSIYSNMKAFIRYMISS
Fragilariopsis_cylindrus_9        --------------DEV---PAMTGD-----GVNDAPALKQ------ASIGIAMGISGTEVAKEASDMILADDNFATIVDAVEEGRSIYSNMKAFIRYMISS
Fragilariopsis_cylindrus_10       --------------DEV---PAMTGD-----GVNDAPALKQ------ASIGIAMGISGTEVAKEASDMILADDNFATIVDAVEEGRSIYSNMKAFIRYMISS
Aquilegia_caerulea_4              --------------NEV---VAMTGD-----GVNDAPALKK------ADIGIAMG-SGTAVAKSASDMVLADDNFASIVAAVAEGRAIYNNTKQFIRYMISS
Glycine_max_5                     --------------NEV---VAMTGD-----GVNDAPALKK------ADIGIAMG-SGTAVAKSASDMVLADDNFASIVAAVAEGRAIYNNTKQFIRYMISS
Medicago_truncatula_2             --------------NEV---VAMTGD-----GVNDAPALKK------ADIGIAMG-SGTAVAKSASDMVLADDNFASIVAAVAEGRAIYNNTKQFIRYMISS
A.thaliana_AT1G10130.1            --------------NEV---VAMTGD-----GVNDAPALKK------ADIGIAMG-SGTAVAKSASDMVLADDNFASIVAAVAEGRAIYNNTKQFIRYMISS
Oryza_sativa_2                    --------------NEV---VAMTGD-----GVNDAPALKK------ADIGIAMG-SGTAVAKSASDMVLADDNFATIVAAVSEGRAIYNNTKQFIRYMISS
Selaginella_moellendorffii_4      --------------KEV---VAMTGD-----GVNDAPALKQ------ADIGIAMG-SGTAVAKGASDMVLADDNFATVVAAVAEGRAIYNNTKQFIRYMISS
Micromonas_pusilla_2              --------------GHV---VAMTGD-----GVNDAPALKR------ADIGIAMG-SGTAVAKSSSDMVLADDNFATIVSAVAEGRAIYNNTKQFIRYMVSS
Micromonas_sp_2                   --------------GQV---VAMTGD-----GVNDAPALRL------ADIGIAMG-SGTAVAKNAADMVLADDNFATIVTAVAEGRGIFNNTKQFVRYMVSS
Volvox_carteri_2                  --------------GHV---VAMTGD-----GVNDAPALAR------ADIGVAMG-SGTAVAKGAADMVLADDNFATIVAAVAEGRAIYNNTKQFIRYMISS
Chlamydomonas_reinhardtii_2       --------------GHV---VAMTGD-----GVNDAPALSR------ADIGVAMG-SGTAVAKGASDMVLADDNFATIVSAVAEGRAIYNNTKQFIRYMISS
Dunaliella_salina_1               --------------GHV---VAMTGD-----GVNDAPALLR------ADIGIAMG-SGTAVAKHAADMVLGDDNFATIVFA---------------------
Ostreococcus_lucimarinus_2        --------------DNI---VAMTGD-----GVNDAPALKC------ADIGIAMG-SGTAVAKGAADMVLADDNFSTIVEAVAEGRAIYNNTKQFIRYMVSS
Phaeodactylum_tricornutum_2       --------------GDI---VAMTGD-----GVNDATALKG------ADIGIAMGLKGTDVAKEAADVVLADDNFRTITMAIAEGKGIFFNIRCFLAFQLST
Chlamydomonas_reinhardtii_3       --------------GQT---AAMTGD-----GVNDAPALKA------ADVGVAMGITGTDVSKEAAKMVLADDNFATIVAAVREGRRVWDNIRKILIFNLPV
Chlamydomonas_reinhardtii_4       --------------GQT---AAMTGD-----GVNDAPALKA------ADVGVAMGITGTDVSKEAAKMVLADDNFATIVAAVREGRRVWDNIRKILVFNLPV
Thalassiosira_pseudonana_4        --------------GQV---SSMTGD-----GVNDAPALKA------ANMGVAMGKEGTDVAREASEMILADDNFATIVFAVKQGRVVWDNLRKVLLVNTPI
Thalassiosira_pseudonana_5        --------------GQV---TSMTGD-----GVNDAPALKA------ANMGVAMGKEGTDVAREASEMILADDNFATIVYAVKQGRVVWDNLRKVLLVNTPI
```

```
Fragilariopsis_cylindrus_11         -------------GEV---ASMTGD-----GVNDAPALKA-----ANMGVAMGKEGTDVAREAADMILADDNFATILTAVKEGRVVWDNLRKVLMINTPI
Fragilariopsis_cylindrus_12         -------------GEV---ASMTGD-----GVNDAPALKA-----ANMGVAMGKEGTDVAREAADMILADDNFATILTAVKEGRVVWDNLRKVLMINTPI
Pseudo-nitzschia_multiseries_3      -------------GEV---ASMTGD-----GVNDAPALKA-----ANMGVAMGLEGTDVAREAAEMILADDNFATILSAVKEGRVVWDNLRKVLMINTPI
Phaeodactylum_tricornutum_3         -------------GEV---CGMTGD-----GVNDAPALKA-----ADMGVAMGKEGTDVAREAAEMILADDNFATIISAVREGRVVWDNLRKVLLINTPI
Micromonas_pusilla_3                -------------GWV---CAMTGD-----GVNDAPALQR-----ADIGVAMGLEGTEVAKGASDMILTDDNFCSIVKAIEKGRIIYAGIQKFVAFIMSV
Micromonas_sp_3                     -------------GFV---CAMTGD-----GVNDAPALNR-----ADIGVAMGLEGTEVAKGASDMILTDDNFCSIVKAIEKGRTIYAGIQKFVSFIMSV
Chlamydomonas_reinhardtii_5         -------------GEI---VAVTGD-----GTNDAPALKE-----SDVGLAMGIAGTEVAKEAADIIILDDNFSSIVKSVLWGRAVYMNIRKFLVFQLSI
Chlamydomonas_reinhardtii_6         -------------GEV---VAVTGD-----GTNDAPALKE-----SDVGLAMGIAGTEVAKEAADIIILDDNFSSIVKSVLWGRTVYMNIRKFLVFQLSV
Volvox_carteri_3                    -------------GEV---VAVTGD-----GTNDAPALKE-----SDVGLAMGIAGTEVAKEAADIIILDDNFSSIVKSVLWGRCVYMNIRKFLVFQLSI
Volvox_carteri_4                    -------------GEV---VAVTGD-----GTNDAPALKE-----SDVGLAMGIAGTEVAKEAADIIILDDNFSSIVKSVLWGRCVYMNIRKFLVFQLSI
Volvox_carteri_5                    -------------PDSTFGVQSNGG-----GGPRGERMQEGSTKGGDCALGEADGGTEVSKEAADIVILDDNFSSIVKSVLWGRSVFANIRKFLQFQLTV
Chlamydomonas_reinhardtii_7         -------------GDV---VAVTGD-----GTNDAPALKE-----SDVGLAMGIAGTEVAKEAADIVILDDNFSSIVKSVKWGRSVFANIRKFLQFQLTV
Chlamydomonas_reinhardtii_8         -------------GDV---VAVTGD-----GTNDAPALKE-----SDVGLAMGIAGTEVAKEAADIVILDDNFSSIVKSVLWGRSVFTNIRKFLMFQLTV
Volvox_carteri_6                    -------------GEV---VAVTGD-----GTNDAPALKE-----SDVGLAMGIAGTEVAKEAADIVIMDDNFSSIVKSVLWGRSVFTNIRKFLMFQLTV
Ostreococcus_lucimarinus_3          -------------GEV---VAVTGD-----GTNDAPALKD-----ADVGFALGIAGTEIAKEACDIVILDDNIKSMAKAVLWGRNVYQSIRKFLQFQLVV
Micromonas_sp_4                     -------------GEV---VSVTGD-----GTNDAPALKD-----ADVGFALGIAGTEIAKEACDIVILDDNIQSMAKAVLWGRNVFQSIRKFLQFQLVV
Selaginella_moellendorffii_5        -------------NEI---VAVTGD-----GTNDAPALRE-----AHIGLSMGIVGTEVAKESSDIIILDDNFASVVKVVHWGRSVYENIQKFIQFQLTV
A.thaliana_AT3G21180.1              -------------GDV---VAVTGD-----GTNDAPALHE-----ADIGLSMGISGTEVAKESSDIIILDDNFASVVKVVRWGRSVYANIQKFIQFQLTV
Aquilegia_caerulea_5                -------------GHV---VAVTGD-----GTNDAPALHE-----ADIGLAMGIAGTEVAKESSDIIILDDNFSSVVKVVRWGRSVYANIQKFIQFQLTV
A.thaliana_AT5G57110.2              -------------GHV---VAVTGD-----GTNDAPALHE-----ADIGLAMGIAGTEVAKESSDIIILDDNFASVVKVVRWGRSVYANIQKFIQFQLTV
Oryza_sativa_3                      -------------GHV---VAVTGD-----GTNDAPALHE-----ADIGLSMGIQGTEVAKESSDIIILDDNFASVVRVVRWGRSVYANIQKFIQFQLTV
Zea_mays_2                          -------------GHV---VAVTGD-----GTNDAPALHE-----ADIGLSMGIQGTEVAKESSDIIILDDNFASVVRVVRWGRSVYANIQKFIQFQLTV
Zea_mays_3                          -------------GHV---VAVTGD-----GTNDAPALHE-----ADIGLSMGISGTEVAKESSDIIILDDDFTSVVKVVRWGRSVYANIQKFIQFQLTV
Zea_mays_4                          -------------GHV---VAVTGD-----GTNDAPALHE-----ADIGLSMGISGTEVAKESSDIIILDDDFTSVVKVVRWGRSVYANIQKFIQFQLTV
Selaginella_moellendorffii_6        -------------NDV---VAVTGD-----GTNDAPALHE-----ADIGLSMGIQGTEVAKESSDIIILDDNFASVVKVVRWGRSVYANIQKFIQFQLTV
Selaginella_moellendorffii_7        -------------GEI---VAVTGD-----GTNDAPALHE-----ASIGLSMGIAGTEVAKESSDIIIMDDDFASIVKVVRWGRAVYANIQKFVQFQCTV
Aquilegia_caerulea_6                -------------GEV---VAVTGD-----GTNDAPALHE-----ADIGLAMGIAGTEVAKESADVIILDDNFSTIVTVAKWGRSVYINIQKFVQFQLTV
Medicago_truncatula_3               -------------GEV---VAVTGD-----GTNDAPALHE-----ADIGLAMGIAGTEVAKESADVIILDDNFSTIVTVAKWGRSVYINIQKFVQFQLTV
A.thaliana_AT2G22950.1              -------------DEV---VAVTGD-----GTNDAPALHE-----ADIGLAMGIAGTEVAKEIADVIILDDNFSTIVTVAKWGRSVYINIQKFVQFQLTV
A.thaliana_AT4G37640.1              -------------DEV---VAVTGD-----GTNDAPALHE-----ADIGLAMGIAGTEVAKESADVIILDDNFSTIVTVAKWGRSVYINIQKFVQFQLTV
Oryza_sativa_4                      -------------DEV---VAVTGD-----GTNDAPALHE-----ADIGLAMGIAGTEVAKESADVIILDDNFSTIVTVAKWGRSVYINIQKFVQFQLTV
Zea_mays_5                          -------------DEV---VAVTGD-----GTNDAPALHE-----ADIGLAMGIAGTEVAKESADVIILDDNFSTIVTVAKWGRSVYINIQKFVQFQLTV
Oryza_sativa_5                      -------------NEV---VAVTGD-----GTNDAPALHE-----ADIGLAMGIAGTEVAKESADVIILDDNFSTIVTVAKWGRSVYVNIQKFVQFQLTV
A.thaliana_AT1G27770.1              -------------QEV---VAVTGD-----GTNDAPALHE-----ADIGLAMGISGTEVAKESADVIILDDNFSTIVTVAKWGRSVYINIQKFVQFQLTV
Oryza_sativa_6                      -------------NEV---VAVTGD-----GTNDAPALRE-----ADIGLAMGIAGTEVAKESADVVILDDNFSTIVTVAKWGRSVYVNIQKFVQFQLTV
Zea_mays_6                          -------------NEV---VAVTGD-----GTNDAPALRE-----ADIGLAMGIAGTEVAKESADVVILDDNFSTIVTVAKWGRSVYVNIQKFVQFQLTV
Aquilegia_caerulea_7                -------------GEV---VAVTGD-----GTNDAPALHE-----SDIGLAMGIAGTEVAKENADIIIMDDNFSTIVNVAKWGRAVYINIQKFVQFQLTV
Oryza_sativa_7                      -------------QEV---VAVTGD-----GTNDAPALHE-----SDIGLAMGITGTEVAKESADVIIMDDNFETIVNVARWGRAVYLNIQKFVQFQLTV
Selaginella_moellendorffii_8        -------------DEV---VSVTGD-----GTNDAPALHE-----ADVGLAMGIAGTEVAKESADIVILDDKFNTIVVVAKWGRSVYTNIQKFVQFQLTV
Selaginella_moellendorffii_9        -------------GEV---VGVTGD-----GTNDAPALRE-----ADIGISMGIAGTEVAKESSDIIILDDNFASIVNVALWGRSVYTNIQKFVQFQATV
Oryza_sativa_8                      -------------GHV---VAVTGD-----GTNDAPALKE-----ADVGLSMGVQGTEVAKESSDIVILNDNFDTVVTATRWGRCVYNNIQKFIQFQLTV
Fragilariopsis_cylindrus_13         -------------GEV---VGVTGD-----GTNDGPALKA-----ADVGLSMGLSGTDVAKNASDIIIMDDKFSSIVKAVLWGRSVYDNIRKFLQFQLTV
Pseudo-nitzschia_multiseries_4      -------------GEV---VGVTGD-----GTNDGPALKA-----ADVGLSMGLSGTDVAKNASDIIIMDDKFSSIVKAVLWGRSVFDNIRKFLQFQLTV
Phaeodactylum_tricornutum_4         -------------GEV---VGVTGD-----GTNDAPALKA-----ADVGLSMGLSGTDVAKKASDIIIMDDNFASIVRAVLWGRSVFDNIRKFLQFQLTV
Thalassiosira_pseudonana_6          ----EDNIIIFPDRQV---VAMTGD-----GTNDAPALKR-----ADIGFAMGIAGTQIAKDAADIILLDDNFASIVTAAKWGRNVYASIQKFLQFQLTV
Phaeodactylum_tricornutum_5         KYNLEDGINIFPDRQV---IAMTGD-----GTNDAPALKR-----ADIGFAMGIAGTQIAKDAADIILLDDNFASIVTAAKWGRNIYASIQKFLQFQLTV
Phaeodactylum_tricornutum_6         -----EGIKVFPDRQV---IAMTGD-----GTNDAPALKR-----ADIGFAMGIAGTQIAKDAADIILLDDNFASIVTAAKWGRNVYASIQKFLQFQLTV
```

```
                                  1501
Skeletonema_marinoi_ABC           NIGEIAAILFAAVCGFPE--------PLSAMHLLWVNLVTDGPPATALGFNPPSPDVMTQKPR-------------------------------------
Thalassiosira_pseudonana_1        NIGEIAAILISAVCGFPE--------PLSAMHLLWVNLVTDGPPATALGFNPPAPDVMSQKPR-------------------------------------
Thalassiosira_oceanica_1          NIGEIAAILLSTLCGFPE--------PLSAMHLLWVNLVTDGPPATALGFNPPAPDVMKQKPR-------------------------------------
Phaeodactylum_tricornutum_1       NIGEICAIFFATLAGFPE--------PLTAMHLLWVNLVTDGPPATALGFNPPAPDLMEQPPR-------------------------------------
Fragilariopsis_cylindrus_1        NIGEICAIFFATLFGFPE--------PLTAMHLLWVNLVTDGPPATALGFNPPLPNIMKQPPR-------------------------------------
Fragilariopsis_cylindrus_2        NIGEICAIFFATLFGFPE--------PLTAMHLLWVNLVTDGPPATALGFNPPLPNIMKQPPR-------------------------------------
Fragilariopsis_cylindrus_3        NIGEICAIFFATLFGFPE--------PLTAMHLLWVNLVTDGPPATALGFNPPLPNIMKQPPR-------------------------------------
Fragilariopsis_cylindrus_4        NIGEICAIFFATLFGFPE--------PLTAMHLLWVNLVTDGPPATALGFNPPLPNIMKQPPR-------------------------------------
Fragilariopsis_cylindrus_5        NIGEICAIFFATLFGFPE--------PLTAMHLLWVNLVTDGPPATALGFNPPLPNIMKQPPR-------------------------------------
Fragilariopsis_cylindrus_6        NIGEICAIFFATLFGFPE--------PLTAMHLLWVNLVTDGPPATALGFNPPLPNIMKQPPR-------------------------------------
Fragilariopsis_cylindrus_7        NIGEICAIFFATLFGFPE--------PLTAMHLLWVNLVTDGPPATALGFNPPLPNIMKQPPR-------------------------------------
Fragilariopsis_cylindrus_8        NIGEICAIFFATLFGFPE--------PLTAMHLLWVNLVTDGPPATALGFNPPLPNIMKQPPR-------------------------------------
Pseudo-nitzschia_multiseries_1    NIGEICAIFFATLLGFPE--------PLTAMHLLWVNLVTDGPPATALGFNPPSPNSMKEKPR-------------------------------------
Thalassiosira_pseudonana_2        NIGEVIGVFLATILGFPQ--------LLTPLHLLWVNLVTDGPPATALGFNPPGPGVMAQKPR-------------------------------------
Ostreococcus_lucimarinus_1        NVGEVVSIFLTAALGMPE--------GLVPVQLLWVNLVTDGPPATALGFNPPDKDIMTKPPR-------------------------------------
Zea_mays_1                        NIGEVASIFLTSALGIPE--------GLIPVQLLWVNLVTDGPPATALGFNPPDKDIMKKPPR-------------------------------------
Oryza_sativa_1                    NIGEVASIFLTSALGIPE--------GLIPVQLLWVNLVTDGPPATALGFNPPDKDIMKKPPR-------------------------------------
Aquilegia_caerulea_1              NIGEVASIFLTAALGIPE--------GLIPVQLLWVNLVTDGPPATALGFNPPDRDIMKKPPR-------------------------------------
A.thaliana_AT1G07810.1            NIGEVASIFLTAALGIPE--------GMIPVQLLWVNLVTDGPPATALGFNPPDKDIMKKPPR-------------------------------------
A.thaliana_AT1G07670.1            NIGEVASIFLTAALGIPE--------GMIPVQLLWVNLVTDGPPATALGFNPPDKDIMKKPPR-------------------------------------
Medicago_truncatula_1             NIGEVASIFLTAALGIPE--------GLIPVQLLWVNLVTDGPPATALGFNPPDKDIMKKPPR-------------------------------------
Glycine_max_1                     NIGEVASIFLTAALGIPE--------GLIPVQLLWVNLVTDGPPATALGFNPPDKDIMKKPPR-------------------------------------
Glycine_max_2                     NIGEVASIFLTAALGIPE--------GLIPVQLLWVNLVTDGPPATALGFNPPDKDIMKKPPR-------------------------------------
Aquilegia_caerulea_2              NIGEVASIFLTAALGIPE--------GLIPVQLLWVNLVTDGPPATALGFNPPDKDIMKKPPR-------------------------------------
Selaginella_moellendorffii_1      NIGEVASIFLTAAVGMPE--------GLTPVQLLWVNLVTDGPPATALGFNPPDVDIMRKPPR-------------------------------------
Physcomitella_patens_1            NIGEVASIFMTAALGMPE--------GLVPVQLLWVNLVTDGPPATALGFNPPDLDIMQKPPR-------------------------------------
Physcomitella_patens_2            NIGEVASIFMTAALGMPE--------GLVPVQLLWVNLVTDGPPATALGFNPPDLDIMQKPPR-------------------------------------
Physcomitella_patens_3            NMGEVASIFLTAALGLPE--------GLIPVQLLWVNLVTDGPPATALGFNPPDPDIMKKPPR-------------------------------------
Selaginella_moellendorffii_2      NIGEVASIFLTAALGMPE--------GLIPVQLLWVNLVTDGPPATALGFNPPDLGIMKKPPR-------------------------------------
Glycine_max_3                     NIGEVISIFLTAALGIPE--------CMISVQLLWVNLVTDGPPATALGFNPADVDIMQKPPR-------------------------------------
Glycine_max_4                     NVGEVISIFLTAALGIPE--------CMIPVQLLWVNLVTDGPPATALGFNPADIDIMQKPPR-------------------------------------
Aquilegia_caerulea_3              NVGEVISIFLTAALGIPE--------CLIPVQLLWVNLVTDGPPATALGFNPADVDIMRKPPR-------------------------------------
A.thaliana_AT4G00900.1            NVGEVISIFLTAALGIPE--------CMIPVQLLWVNLVTDGPPATALGFNPADIDIMKKPPR-------------------------------------
Chlamydomonas_reinhardtii_1       NIGEVASIFLTAALGLPE--------GLIPVQLLWVNLVTDGPPATALGFNAPDKDIMSKPPR-------------------------------------
Volvox_carteri_1                  NIGEVASIFLTAALGLPE--------GLIPVQLLWVNLVTDGPPATALGFNPPDKDIMSRPPR-------------------------------------
Micromonas_pusilla_1              NVGEVVSIFLTAALGMPE--------GLIPVQLLWVNLVTDGPPATALGFNPPDVDIMTKTPR-------------------------------------
Micromonas_sp_1                   NVGEVVSIFLTAALGMPE--------GLIPVQLLWVNLVTDGPPATALGFNPPDVDIMTKKPR-------------------------------------
Selaginella_moellendorffii_3      NIGEVVAIFLTAILGMPQ--------GLIPVQLLWVNLVTDGAPATALGFNPPDTDIMDRPPR-------------------------------------
Thalassiosira_oceanica_2          NIGEVAAIFFTAALGLPE--------GLIPVQLLWVNLVTDGPPATALGFNPPDADIMKKLPR-------------------------------------
Thalassiosira_pseudonana_3        NIGEVAAIFFTAALGMPE--------GLIPVQLLWVNLVTDGPPATALGFNPADKDIMKKLPR-------------------------------------
Pseudo-nitzschia_multiseries_2    NIGEVVSIFLTSTIGCPE--------GLIPVQLLWVNLVTDGPPATALGFNPPDPDVMLKPPR-------------------------------------
Fragilariopsis_cylindrus_9        NIGEVVSIFLTSSLGVPE--------GLIPVQLLWVNLVTDGPPATALGFNPPDPDVMLKPPR-------------------------------------
Fragilariopsis_cylindrus_10       NIGEVVSIFLTSSLGVPE--------GLIPVQLLWVNLVTDGPPATALGFNPPDPDVMLKPPR-------------------------------------
Aquilegia_caerulea_4              NIGEVVCIFVAAVLGIPE--------TLVPVQLLWVNLVTDGLPATAIGFNKQDSDVMKAKPR-------------------------------------
Glycine_max_5                     NIGEVVCIFVAAVLGIPD--------TLAPVQLLWVNLVTDGLPATAIGFNKQDSDVMRAKPR-------------------------------------
Medicago_truncatula_2             NIGEVVCIFVAAVLGIPD--------TLAPVQLLWVNLVTDGLPATAIGFNKQDSDVMKVKPR-------------------------------------
A.thaliana_AT1G10130.1            NIGEVVCIFVAAVLGIPD--------TLAPVQLLWVNLVTDGLPATAIGFNKQDSDVMKAKPR-------------------------------------
Oryza_sativa_2                    NIGEVVCIFVAAVLGMPD--------TLVPVQLLWVNLVTDGLPATAIGFNKPDSNIMTVKPRLHHSATTTIRWLTDAHGKQCFGIAFIVDVMGVDGDLD
Selaginella_moellendorffii_4      NIGEVVCIFVAAALGLPE--------TLIPVQLLWVNLVTDGLPATALGFNKQDRNVMMAKPR-------------------------------------
Micromonas_pusilla_2              NIGEVVCIFIAAALGMPE--------TLCPVQLLWVNLVTDGLPATALGFNKPDRDIMRARPR-------------------------------------
Micromonas_sp_2                   NIGEVVCIFIAAALGLPE--------TLCPVQLLWVNLVTDGLPATALGFNKPEQDIMQRRPR-------------------------------------
Volvox_carteri_2                  NIGEVVAIFVAALLGVPE--------VLTPVQLLWVNLVTDGLPATALGFNKPDKDIMAVRPR-------------------------------------
Chlamydomonas_reinhardtii_2       NIGEVVAIFVAALLGVPE--------VLTPVQLLWVNLVTDGLPATALGFNRPDKDIMSRPPR-------------------------------------
Dunaliella_salina_1               --GGVV----------------------------------------------------------------------------------------------
Ostreococcus_lucimarinus_2        NIGEVVCIFIAAALGFPE--------TLVPVQLLWINLVTDGLPATALGFNRADGDIMRQRPR-------------------------------------
Phaeodactylum_tricornutum_2       SFAALTMASIATAFGLPS--------PLNATQILWINILMDGPPAQSLGVEPVDEKILRAKPR-------------------------------------
Chlamydomonas_reinhardtii_3       NLAQGFSVLWSYILSLDN-------VPLTALQVLLVNLITSVTLGLALAAEPPEPDIMERQPR-------------------------------------
Chlamydomonas_reinhardtii_4       NLAQGFSVLYSYILGFKD-------VPLTALQVLLVNLITSVTLGLALAAEPPEPDIMERQPR-------------------------------------
Thalassiosira_pseudonana_4        NNSQGLSVLVGLLVRLPN-------TPITTIQILYSNFICAVTLGFVCAIEPAEDGIMALPPR-------------------------------------
Thalassiosira_pseudonana_5        NNSQGLCVFFGMLVRLPN-------IPITTIQILYSNFICAVTLGFVCAVEPAEDGIMALPPR-------------------------------------
```

```
Fragilariopsis_cylindrus_11        NNAQGLSVLFGIFCGFSRGESAFDGVILTSIQVLYCNLICAVTLGFVAAVEPAEDGIMDVPPR------------------------------------
Fragilariopsis_cylindrus_12        NNAQGLSVLFGIFCGFSRGESAFDGVILTSIQVLYCNLICAVTLGFVAAVEPAEDGIMDVPPR------------------------------------
Pseudo-nitzschia_multiseries_3     NNAQGLSVLFGIICGFNRGTEQNDKVILTAIQVLYCNLICAVTLGFVAAVEPAEAGIMDVPPR------------------------------------
Phaeodactylum_tricornutum_3        NNAQGMSVLFGLLFGLKE-------TPLSSIQVLYCNLICAVTLGFVAAVEPAEDGIMELEPR------------------------------------
Micromonas_pusilla_3               HFAEVVQIFLCIVSSIPV--------MRQPLQILFLILVTDLPPSIALGFEPGEDLTMKRSPR------------------------------------
Micromonas_sp_3                    HFAEVLQIFLCIVSSIPV--------MRQPLQILFLILVTDLPPSIALGFEPGEALTMKRAPR------------------------------------
Chlamydomonas_reinhardtii_5        NLVAMISAAVGALYGGVP--------PLNVLQLLWVNMIMDTLAALALATEDPYPELLDDKPH------------------------------------
Chlamydomonas_reinhardtii_6        NLVAMISAAVGALYGGVP--------PLNVLQLLWVNMIMDTLAALALATENPYPELLDEMPH------------------------------------
Volvox_carteri_3                   NLVAMISAAVGALYGGVP--------PLNVLQLLWVNMIMDTLAALALATEDPYPELLQDTPH------------------------------------
Volvox_carteri_4                   NLVAMISAAVGALYGGVP--------PLNVLQLLWVNMIMDTLAALALATEDPYPELLQDTPH------------------------------------
Volvox_carteri_5                   NFVALVTAFVGAVAGGHE--------PLNILQLLWVNLIMDTMGALALATEDPHPALLLQRPN------------------------------------
Chlamydomonas_reinhardtii_7        NLVALVTAFIGAVVGGHE--------PLNILQLLWVNLIMDTMGALALATEAPHPTLLLQRPN------------------------------------
Chlamydomonas_reinhardtii_8        NFVALVIAFFGAVIGGQE--------PLNVLQLLWVNLIMDTMGALALATEDPNPELLLMKPY------------------------------------
Volvox_carteri_6                   NFVALVIAFFGAVIDGHE--------PLNVLQLLWVNLIMDTMGALALATEDPNPELLLMKPY------------------------------------
Ostreococcus_lucimarinus_3         NVVAVSLNLIAAIAGIKE-------LPLAAVPLLWVNMIMDSMGALALATEPPSPELMKRKPF------------------------------------
Micromonas_sp_4                    NVVAVSLNFISAAAGITE-------LPLAAVPLLWVNMIMDSMGALALATEPPSPHLMERKPF------------------------------------
Selaginella_moellendorffii_5       NLAALSTNLVAAGRSENV--------PLNTVQLLWVNLIMDTLGALALATEPPTEEMMERAPI------------------------------------
A.thaliana_AT3G21180.1             NVAALIINVVAAMSSGDV--------PLKAVQLLWVNLIMDTLGALALATEPPTDHLMHRTPV------------------------------------
Aquilegia_caerulea_5               NVAALIINVVAAVTTGEV--------PLNAVQLLWVNLIMDTLGALALATEPPTDHLMHRSPV------------------------------------
A.thaliana_AT5G57110.2             NVAALVINVVAAISSGDV--------PLTAVQLLWVNLIMDTLGALALATEPPTDHLMGRPPV------------------------------------
Oryza_sativa_3                     NVAALIINVVAAVSSGNV--------PLNAVQLLWVNLIMDTLGALALATEPPTDHLMQRPPV------------------------------------
Zea_mays_2                         NVAALIINVVAAVSSGNV--------PLNAVQLLWVNLIMDTLGALALATEPPTNHLMERPPV------------------------------------
Zea_mays_3                         NVAALVINVIAAVSSGDV--------PLNAVELLWVNLIMDTLGALALATEPPTDNLMKRHPV------------------------------------
Zea_mays_4                         NVAALVINVVAAVSSGDV--------PLNAVELLWVNLIMDTLGALALATEPPTDNLMKRNPV------------------------------------
Selaginella_moellendorffii_6       NVVALVLNVVAAAKSSQV--------PLTAVQLLWVNLIMDTLGALALATEPPTDDLMDRPPV------------------------------------
Selaginella_moellendorffii_7       NAVALMLNFISALSEGAA--------PLTAVQLLWVNLIMDTLGALALATEPPNDAVMYRPPI------------------------------------
Aquilegia_caerulea_6               NVVALIVNFSSACLTGSA--------PLTAVQLLWVNMIMDTLGALALATEPPNDELMKRTPV------------------------------------
Medicago_truncatula_3              NIVALIVNFTSACLTGTA--------PLTAVQLLWVNMIMDTLGALALATEPPNDDLMKRAPV------------------------------------
A.thaliana_AT2G22950.1             NVVALIVNFSSACLTGSA--------PLTAVQLLWVNMIMDTLGALALATEPPNNELMKRMPV------------------------------------
A.thaliana_AT4G37640.1             NVVALVVNFSSACLTGSA--------PLTAVQLLWVNMIMDTLGALALATEPPNDELMKRLPV------------------------------------
Oryza_sativa_4                     NVVALIVNFSSACLTGSA--------PLTAVQLLWVNMIMDTLGALALATEPPNDELMKRTPV------------------------------------
Zea_mays_5                         NVVALVVNFSSACLIGSA--------PLTAVQLLWVNMIMDTLGALALATEPPNDELMKRTPV------------------------------------
Oryza_sativa_5                     NVVALLVNFSSACFTGNA--------PLTAVQLLWVNMIMDTLGALALATEPPNDDLMKREPV------------------------------------
A.thaliana_AT1G27770.1             NVVALIVNFLSACLTGNA--------PLTAVQLLWVNMIMDTLGALALATEPPQDDLMKRSPV------------------------------------
Oryza_sativa_6                     NVVALLVNFTSACFTGDA--------PLTAVQLLWVNMIMDTLGALALATEPPNNNLMKKAPV------------------------------------
Zea_mays_6                         NVVALLVNFSSACFTGDA--------PLTAVQLLWVNMIMDTLGALALATEPPDDNLMKKSPV------------------------------------
Aquilegia_caerulea_7               NVVALMLNFVSACLSGSA--------PLTAVQLLWVNMIMDTLGALALATEPPNDRLMQKPPV------------------------------------
Oryza_sativa_7                     NIVALIVNFVSACIIGSA--------PLTAVQLLWVNMIMDTLGALALATEPPNDEMMKRPPV------------------------------------
Selaginella_moellendorffii_8       NLVALVLNFTSACITGTA--------PLTAVQLLWVNLIMDTLGALALATEPPTDDLMKRTPV------------------------------------
Selaginella_moellendorffii_9       NLVALALNFWSACSTGDV--------PLTVIQLLWVNLIMDTLGALALATESPHAGLMKRPPV------------------------------------
Oryza_sativa_8                     NVAALVINFVSAVTTGRM--------PLTTVQLLWVNLIMDTMGALALATDTPTAGLMRRPPI------------------------------------
Fragilariopsis_cylindrus_13        NVVALTITFLSALVGYDP--------PLNAIMMLWVNLIMDTMGALALGTEPPKLELLDRPPY------------------------------------
Pseudo-nitzschia_multiseries_4     NVVALTITFLSALVGYDP--------PLNAVMMLWVNLIMDTMGALALGTEPPKLELLDRPPY------------------------------------
Phaeodactylum_tricornutum_4        NVVALTITFLAAVVGYQP--------PLNAVMMLWVNLIMDTMGALALGTEPPLKELLDRRPY------------------------------------
Thalassiosira_pseudonana_6         NIAAVVTALVGSFAYAKS--------PLAAIQLLWVNLIMDSLASLALASEPPTEELLKRQPV------------------------------------
Phaeodactylum_tricornutum_5        NISAVVTALVGSFAYQKS--------PLAAIQLLWVNLLMDSLASLALASEPPVDDLLRKPPV------------------------------------
Phaeodactylum_tricornutum_6        NISAVATALVGSFAYQAS--------PLAAIQLLWVNLLMDSLASLALASEPPVDSLLQRPPV------------------------------------
```

```
                                  1601
Skeletonema_marinoi_ABC           -----------------------------------------------------------------------------------------------PSDEP
Thalassiosira_pseudonana_1        -----------------------------------------------------------------------------------------------PSNEP
Thalassiosira_oceanica_1          -----------------------------------------------------------------------------------------------PSNEP
Phaeodactylum_tricornutum_1       -----------------------------------------------------------------------------------------------PSDEP
Fragilariopsis_cylindrus_1        -----------------------------------------------------------------------------------------------SSSEP
Fragilariopsis_cylindrus_2        -----------------------------------------------------------------------------------------------SSSEP
Fragilariopsis_cylindrus_3        -----------------------------------------------------------------------------------------------SSSEP
Fragilariopsis_cylindrus_4        -----------------------------------------------------------------------------------------------SSSEP
Fragilariopsis_cylindrus_5        -----------------------------------------------------------------------------------------------SSSEP
Fragilariopsis_cylindrus_6        -----------------------------------------------------------------------------------------------SSSEP
Fragilariopsis_cylindrus_7        -----------------------------------------------------------------------------------------------SSSEP
Fragilariopsis_cylindrus_8        -----------------------------------------------------------------------------------------------SSSEP
Pseudo-nitzschia_multiseries_1    -----------------------------------------------------------------------------------------------PSNEP
Thalassiosira_pseudonana_2        -----------------------------------------------------------------------------------------------PTSEE
Ostreococcus_lucimarinus_1        -----------------------------------------------------------------------------------------------RKDED
Zea_mays_1                        -----------------------------------------------------------------------------------------------RSDDS
Oryza_sativa_1                    -----------------------------------------------------------------------------------------------RSDDS
Aquilegia_caerulea_1              -----------------------------------------------------------------------------------------------RSDDS
A.thaliana_AT1G07810.1            -----------------------------------------------------------------------------------------------RSDDS
A.thaliana_AT1G07670.1            -----------------------------------------------------------------------------------------------RSDDS
Medicago_truncatula_1             -----------------------------------------------------------------------------------------------RSDDS
Glycine_max_1                     -----------------------------------------------------------------------------------------------HSDDS
Glycine_max_2                     -----------------------------------------------------------------------------------------------HSDDS
Aquilegia_caerulea_2              -----------------------------------------------------------------------------------------------RSDDS
Selaginella_moellendorffii_1      -----------------------------------------------------------------------------------------------RSDDK
Physcomitella_patens_1            -----------------------------------------------------------------------------------------------KSDDV
Physcomitella_patens_2            -----------------------------------------------------------------------------------------------KSNDV
Physcomitella_patens_3            -----------------------------------------------------------------------------------------------KSTDV
Selaginella_moellendorffii_2      -----------------------------------------------------------------------------------------------KSNDK
Glycine_max_3                     -----------------------------------------------------------------------------------------------RSDDP
Glycine_max_4                     -----------------------------------------------------------------------------------------------RNDDP
Aquilegia_caerulea_3              -----------------------------------------------------------------------------------------------KSNDA
A.thaliana_AT4G00900.1            -----------------------------------------------------------------------------------------------KSDDC
Chlamydomonas_reinhardtii_1       -----------------------------------------------------------------------------------------------RANDQ
Volvox_carteri_1                  -----------------------------------------------------------------------------------------------RANDQ
Micromonas_pusilla_1              -----------------------------------------------------------------------------------------------KKDED
Micromonas_sp_1                   -----------------------------------------------------------------------------------------------RKDED
Selaginella_moellendorffii_3      -----------------------------------------------------------------------------------------------LPTEG
Thalassiosira_oceanica_2          -----------------------------------------------------------------------------------------------RTDDS
Thalassiosira_pseudonana_3        -----------------------------------------------------------------------------------------------RADDN
Pseudo-nitzschia_multiseries_2    -----------------------------------------------------------------------------------------------GKDDS
Fragilariopsis_cylindrus_9        -----------------------------------------------------------------------------------------------GKNDA
Fragilariopsis_cylindrus_10       -----------------------------------------------------------------------------------------------GKNDA
Aquilegia_caerulea_4              -----------------------------------------------------------------------------------------------KVNEA
Glycine_max_5                     -----------------------------------------------------------------------------------------------KVNEA
Medicago_truncatula_2             -----------------------------------------------------------------------------------------------KVNEA
A.thaliana_AT1G10130.1            -----------------------------------------------------------------------------------------------KVGEA
Oryza_sativa_2                    QCEHGLLCRLDGGGISSDEDVSAIQKIWHCLVQQTAALTNPLRHCLRLVQCIAAAVSPTFLKLLPIGWTACCASPSLLCEGDTVLAYQQLVLLLIEVNEA
Selaginella_moellendorffii_4      -----------------------------------------------------------------------------------------------KMDEA
Micromonas_pusilla_2              -----------------------------------------------------------------------------------------------RPDES
Micromonas_sp_2                   -----------------------------------------------------------------------------------------------RSDEP
Volvox_carteri_2                  -----------------------------------------------------------------------------------------------RLDEP
Chlamydomonas_reinhardtii_2       -----------------------------------------------------------------------------------------------RLDEP
Dunaliella_salina_1               -------------------------------------------------------------------------------------------------DDP
Ostreococcus_lucimarinus_2        -----------------------------------------------------------------------------------------------SPREQ
Phaeodactylum_tricornutum_2       -----------------------------------------------------------------------------------------------KADEP
Chlamydomonas_reinhardtii_3       -----------------------------------------------------------------------------------------------RRGKR
Chlamydomonas_reinhardtii_4       -----------------------------------------------------------------------------------------------RRGKR
Thalassiosira_pseudonana_4        -----------------------------------------------------------------------------------------------RVGKR
Thalassiosira_pseudonana_5        -----------------------------------------------------------------------------------------------RVGKR
```

Fragilariopsis_cylindrus_11	---RVGKR
Fragilariopsis_cylindrus_12	---RVGKR
Pseudo-nitzschia_multiseries_3	---RVGKR
Phaeodactylum_tricornutum_3	---RVGKR
Micromonas_pusilla_3	---PKTQP
Micromonas_sp_3	---PKTQP
Chlamydomonas_reinhardtii_5	---GRSEA
Chlamydomonas_reinhardtii_6	---GRSEP
Volvox_carteri_3	---GRTEP
Volvox_carteri_4	---GRTEP
Volvox_carteri_5	---GRTEH
Chlamydomonas_reinhardtii_7	---GRTEQ
Chlamydomonas_reinhardtii_8	---GRNEN
Volvox_carteri_6	---GRNEN
Ostreococcus_lucimarinus_3	---GRTAP
Micromonas_sp_4	---GRSAP
Selaginella_moellendorffii_5	---GLSEP
A.thaliana_AT3G21180.1	---GRREP
Aquilegia_caerulea_5	---GRREP
A.thaliana_AT5G57110.2	---GRKEP
Oryza_sativa_3	---GRREP
Zea_mays_2	---GRREP
Zea_mays_3	---GRREP
Zea_mays_4	---GRREP
Selaginella_moellendorffii_6	---GRREP
Selaginella_moellendorffii_7	---SKEAP
Aquilegia_caerulea_6	---GRKGN
Medicago_truncatula_3	---GRKGN
A.thaliana_AT2G22950.1	---GRRGN
A.thaliana_AT4G37640.1	---GRRGN
Oryza_sativa_4	---GRKGN
Zea_mays_5	---GRKGN
Oryza_sativa_5	---GRTGK
A.thaliana_AT1G27770.1	---GRKGN
Oryza_sativa_6	---GRKGK
Zea_mays_6	---GRTGR
Aquilegia_caerulea_7	---GKSVN
Oryza_sativa_7	---RRGDN
Selaginella_moellendorffii_8	---GRKGS
Selaginella_moellendorffii_9	---RRKEN
Oryza_sativa_8	---GRAAP
Fragilariopsis_cylindrus_13	---KRNSS
Pseudo-nitzschia_multiseries_4	---TRNSS
Phaeodactylum_tricornutum_4	---RRDSS
Thalassiosira_pseudonana_6	---NRSKS
Phaeodactylum_tricornutum_5	---NRTES
Phaeodactylum_tricornutum_6	---NRTDS

```
                                1701
Skeletonema_marinoi_ABC         IMTNWMLFRYLVTGLYVG-------FATVGSFVGHYLS--------------------------------------------------------------
Thalassiosira_pseudonana_1      IMTKFMACRYLVTGLYVG-------IATVGSFVGHYRS--------------------------------------------------------------
Thalassiosira_oceanica_1        IMTKWMAFRYLITGLYVG-------IATVGSFVSYYLD--------------------------------------------------------------
Phaeodactylum_tricornutum_1     IMTRWLLTRYCITGLYVG-------LATIGIFAQHYLS--------------------------------------------------------------
Fragilariopsis_cylindrus_1      IMTKWLLIRYMLTGLYVG-------IATIGVFAQHYIK--------------------------------------------------------------
Fragilariopsis_cylindrus_2      IMTKWLLIRYMLTGLYVG-------IATIGVFAQHYIK--------------------------------------------------------------
Fragilariopsis_cylindrus_3      IMTKWLLIRYMLTGLYVG-------IATIGVFAQHYIK--------------------------------------------------------------
Fragilariopsis_cylindrus_4      IMTKWLLIRYMLTGLYVG-------IATIGVFAQHYIK--------------------------------------------------------------
Fragilariopsis_cylindrus_5      IMTKWLLIRYMLTGLYVG-------IATIGVFAQHYIK--------------------------------------------------------------
Fragilariopsis_cylindrus_6      IMTKWLLIRYMLTGLYVG-------IATIGVFAQHYIK--------------------------------------------------------------
Fragilariopsis_cylindrus_7      IMTKWLLIRYMLTGLYVG-------IATIGVFAQHYIK--------------------------------------------------------------
Fragilariopsis_cylindrus_8      IMTKWLLIRYMLTGLYVG-------IATIGVFAQHYIK--------------------------------------------------------------
Pseudo-nitzschia_multiseries_1  IMTKWLLIRYMLTGLYVG-------VATIGIFAQHYLQ--------------------------------------------------------------
Thalassiosira_pseudonana_2      ILTPSLLLRYSTAGLYIG-------IATVGIYASYFVD--------------------------------------------------------------
Ostreococcus_lucimarinus_1      LLSNWVMFRYAVVGLYVG-------VATVGAFAIWFTR--------TSFMGIDL-----------------------SQD--------------------
Zea_mays_1                      LITPWILFRYMVIGLYVG-------VATVGIFIIWYTH--------DSFLGIDL-----------------------ASD--------------------
Oryza_sativa_1                  LITPWILFRYMVIGMYVG-------IATVGVFIIWYTH--------GSFLGIDL-----------------------AGD--------------------
Aquilegia_caerulea_1            LISPWILFRYLVIGLYVG-------VATVGVFIIWFTH--------ASFLGIDL-----------------------SGD--------------------
A.thaliana_AT1G07810.1          LITAWILFRYMVIGLYVG-------VATVGVFIIWYTH--------SSFMGIDL-----------------------SQD--------------------
A.thaliana_AT1G07670.1          LITAWILFRYMVIGLYVG-------VATVGVFIIWYTH--------NSFMGIDL-----------------------SQD--------------------
Medicago_truncatula_1           LINLWILFRYLVIGIYVG-------LATVGVFIIWYTH--------GSFMGIDL-----------------------SSD--------------------
Glycine_max_1                   LINLWILFRYLVIGIYVG-------LATVGIFIIWYTH--------GSFFGIDL-----------------------SGD--------------------
Glycine_max_2                   LINLWILFRYLVIGIYVG-------LATVGIFIIWYTH--------GSFFGIDL-----------------------SGD--------------------
Aquilegia_caerulea_2            LISAWILFRYLVIGLYVG-------IATVGVFIIWFTQ--------SSFLGIDL-----------------------SGD--------------------
Selaginella_moellendorffii_1    LINSWVFFRYMVIGLYVG-------LATVGIFALWYTH--------GSFLGLDI-----------------------SGD--------------------
Physcomitella_patens_1          LINAWVFFRYLVIGLYVG-------VATVGAFALWYTQ--------ASFLGINL-----------------------AAD--------------------
Physcomitella_patens_2          LINGWVFFRYLVIGLYVG-------IATVGAFALWYTH--------ASFLGINL-----------------------AAD--------------------
Physcomitella_patens_3          LINSWVLLRYLIIGLYVG-------IATVGAFSLWFTR--------FSFLGIDL-----------------------SSD--------------------
Selaginella_moellendorffii_2    LINAWVLLRYMVIGLYVG-------VATVGIFALWYTH--------DSFLGIDL-----------------------SSD--------------------
Glycine_max_3                   LISSWVLFRYLVIGSYVG-------LATVGIFVLWYTQ--------ASFLGINL-----------------------VSD--------------------
Glycine_max_4                   LISSWVLFRYLVIGSYVG-------LATVGIFVLWYTQ--------ASFLGINL-----------------------VSD--------------------
Aquilegia_caerulea_3            LIDSWVLFRYLVIGSYVG-------IATVGVFILWYTQ--------GSFLGINL-----------------------AED--------------------
A.thaliana_AT4G00900.1          LIDSWVLIRYLVIGSYVG-------VATVGIFVLWYTQ--------ASFLGISL-----------------------ISD--------------------
Chlamydomonas_reinhardtii_1     LITPWVFFRYMVIGGYVG-------VATVGAFVSWYMY--------DRFMGIDL-----------------------SRD--------------------
Volvox_carteri_1                LITPWVFFRYMVIGGYVG-------VATVGAFVSWYMF--------DHFVGIDL-----------------------SRD--------------------
Micromonas_pusilla_1            LISAWALVRYLVVGLYVG-------AATVGVFAVWYTR--------SSFLGIDL-----------------------SGD--------------------
Micromonas_sp_1                 LISTWAMVRYLVVGLYVG-------AATVGIFAVWYTK--------TEFLGIDL-----------------------AKD--------------------
Selaginella_moellendorffii_3    FISGWTLFRFLTIGLYVG-------LATIGIFGLWYLND-------DSFLGIDL-----------------------SRD--------------------
Thalassiosira_oceanica_2        LITPWVFFRYMVVGIYVG-------FACVAVFAYWYIY---------------------------------------HEG--------------------
Thalassiosira_pseudonana_3      LITPWVFFRYMVVGIYVG-------FACVGVFAYWYMY                                       YES--------------------
Pseudo-nitzschia_multiseries_2  LITNWVFFRYMVIGLYVG-------FATVGIFVFWYTEYES----FVSFLGPSLMETLG-----------------ATFD--------------------
Fragilariopsis_cylindrus_9      LINNWVFFRYMVIGLYVG-------FATVGIFIFWYIGYEP----FVNYLGPDLMNTLG-----------------STYD--------------------
Fragilariopsis_cylindrus_10     LINNWVFFRYMVIGLYVG-------FATVGIFIFWYIGYEP----FVNYLGPDLMNTLG-----------------STYD--------------------
Aquilegia_caerulea_4            VVSGWLFFRYLVIGAYVG-------LATVAGFIWWFVYSEN-----------------------------------------------------------
Glycine_max_5                   VVTGWLFFRYLVIGAYVG-------LATVAGFIWWFVYSDS-----------------------------------------------------------
Medicago_truncatula_2           VVTGWLFFRYLVIGAYVG-------LATVAGFIWWFVYADS-----------------------------------------------------------
A.thaliana_AT1G10130.1          VVTGWLFFRYLVIGVYVG-------LATVAGFIWWFVYSDG-----------------------------------------------------------
Oryza_sativa_2                  VVNGWLFFRYLIIGAYVG-------LATIAGFVWWFVYSED-----------------------------------------------------------
Selaginella_moellendorffii_4    IVNGWLFFRYMVVGAYVG-------LATVGGFIWWFLYHEE-----------------------------------------------------------
Micromonas_pusilla_2            IVDRWLFVRYLVVGMYVG-------FVTVGAFAWWYMSYLD-----------------------------------------------------------
Micromonas_sp_2                 IVDRWLFVRYIVVGIYVGLELPDVVFTLIGGFVWWYMFHSG-----------------------------------------------------------
Volvox_carteri_2                IVNGWLFIRYLVVGMYVG-------LVTVAGFLWWFLGYQG-----------------------------------------------------------
Chlamydomonas_reinhardtii_2     IVNGWLFVRYLVVGMYVG       LATVGGFLWWFLAHEG-----------------------------------------------------------
Dunaliella_salina_1             IVNGWLFLRYLIIGMYVG-------IVTVYGFIWWYISYPE-----------------------------------------------------------
Ostreococcus_lucimarinus_2      IVDRWLLIRYLIIGVYVG       IATVGSFGWWFMSYPG-----------------------------------------------------------
Phaeodactylum_tricornutum_2     IVTRALLLRATSSAALIV-------FLTLKVFS-------------------------------------------------------------------
Chlamydomonas_reinhardtii_3     LVGKLLLWRCFFVCNVVV-------ALVLGMFYWGGED--------------------------------------------------------------
Chlamydomonas_reinhardtii_4     LVGKLLLWRMFFVCHVVV-------ALVLGLFYWAGRG--------------------------------------------------------------
Thalassiosira_pseudonana_4      LIGRFLFLRIIVGTVLLT-------GCVVASAIIVDTI--PRYDY-------------------------------------------------------
Thalassiosira_pseudonana_5      LIGRYLFLRIILGTVILT-------GCVVGGALIVD----------------------------------------------------------------
```

```
Fragilariopsis_cylindrus_11        LIGRFLFLRIVLGTILLV-------LVTVASSIWAEYELENNYDY-------------------------------------------------------
Fragilariopsis_cylindrus_12        LIGRFLFLRIVLGTILLV-------LVTVASSIWAEYELENNYDY-------------------------------------------------------
Pseudo-nitzschia_multiseries_3     LIGRFLFLRIALGTVVLV-------FVTVGASLWAEHILKTEYGM-------------------------------------------------------
Phaeodactylum_tricornutum_3        LIGRFLALRIAIGTITLV-------GCTVGSVFWAK----------------------------------------------------------------
Micromonas_pusilla_3               VVQMWMWRGIVINGMILT-------VCIFCTYLIALHAYAGAF----------------------------------LTDDITDTSRSSCS---------
Micromonas_sp_3                    IVMNWMWRGIVANGLILT-------VCIFYTYMIALWAYAGAF----------------------------------TSDEITDPTRTSCA---------
Chlamydomonas_reinhardtii_5        IITGLMYTHIVVAALY--------KLFWLFACLYGLPR----------VLP---------------------AYATLTKGEYYQT---MCP---------
Chlamydomonas_reinhardtii_6        IITGYMYMHILAGATY--------KLIWLFACLYGLPR----------AIS---------------------AYAVLTPDEYYRE---DCN---------
Volvox_carteri_3                   IITGLMYMHIVAAALY--------KLFWLFACLYGLPR----------VID---------------------RYSVLGKDEYYQD---KCD---------
Volvox_carteri_4                   IITGLMYMHIVAAALY--------KLFWLFACLYGLPR----------VID---------------------RYSVLGKDEYYQD---KCD---------
Volvox_carteri_5                   LINWKMSKHIFVQGCY--------QMLWMFLCLYLLPM----------GPKRGRDA-VSYASSCRTVVMMLYSFSCSHAAQLLWQ---RAVPLISGVSSR
Chlamydomonas_reinhardtii_7        LINAKMTKHILVQGSY--------QMIWMFLCLYLLPQ----------GPPG------------------LEQYKVHNEAYMANQ---DCV---------
Chlamydomonas_reinhardtii_8        LITRIMWKHILVQGFY--------QIFWMFLILYGMPV----------FLNGAKYNG---------------RYDIEGREDWWKR---ECM---------
Volvox_carteri_6                   LITRIMWKHILVQGCY--------QLFWMFFILYGAPK----------ILTDA-------------------RYAIEPKEDFWMR---ECS---------
Ostreococcus_lucimarinus_3         LINKHMWRNIIGVSVY--------QLTVCMVFMFDGKR----------LLD-------------------------------------------------
Micromonas_sp_4                    LVNKEMWRNIVVMSLY--------QLIVCLVLLFAGQD----------LLG-------------------------------------------------
Selaginella_moellendorffii_5       LVTNVMWRNIFGQAAY--------QVAVLLVLYFRGDQ----------ILH-------------------------------------------------
A.thaliana_AT3G21180.1             LITNIMWRNLLVQSFY--------QVAVLLVLNFAGLS----------ILG-------------------------------------------------
Aquilegia_caerulea_5               LITNIMWRNLLVQALY--------QVIVLLVLNFMGKN----------ISF-------------------------------------------------
A.thaliana_AT5G57110.2             LITNIMWRNLLIQAIY--------QVSVLLTLNFRGIS----------ILG-------------------------------------------------
Oryza_sativa_3                     LITNVMWRNLIIMALF--------QVIVLLTLNFRGTS----------LLQ-------------------------------------------------
Zea_mays_2                         LVTNIMWRNLIIMATF--------QVSVLLSLNFKGIS----------LLQ-------------------------------------------------
Zea_mays_3                         LVTNVMWRNLFIQALY--------QIAVLLIFNFDGKR----------ILR-------------------------------------------------
Zea_mays_4                         LVTNIMWRNLFVQALY--------QVAILLIFDFDGVR----------ILR-------------------------------------------------
Selaginella_moellendorffii_6       LVTNIMWRNIFVQAIY--------QLSVLFTLFFGGLK----------ILK-------------------------------------------------
Selaginella_moellendorffii_7       LINNIMWRNLLGQSIY--------QLGLLLVLKFKGIE----------ILN-------------------------------------------------
Aquilegia_caerulea_6               FISNVMWRNILGQSLY--------QFVVIWYLQTRGKA----------LFS-------------------------------------------------
Medicago_truncatula_3              FISNVMWRNILGQSLY--------QFMVIWFLQSKGKT----------IFS-------------------------------------------------
A.thaliana_AT2G22950.1             FITNAMWRNILGQAVY--------QFIIIWILQAKGKS----------MFG-------------------------------------------------
A.thaliana_AT4G37640.1             FITNAMWRNILGQAVY--------QFIVIWILQAKGKA----------MFG-------------------------------------------------
Oryza_sativa_4                     FISNIMWRNILGQAFY--------QFIVIWYLQTEGKW----------LFG-------------------------------------------------
Zea_mays_5                         FISNVMWRNIMGQALY--------QFLVIWSLQSRGKS----------LFG-------------------------------------------------
Oryza_sativa_5                     FITNVMWRNILGQSFY--------QFIVMWYLQTQGKS----------MFG-------------------------------------------------
A.thaliana_AT1G27770.1             FISNVMWRNILGQSLY--------QLVIIWCLQTKGKT----------MFG-------------------------------------------------
Oryza_sativa_6                     FITNVMWRNIVGQSLY--------QFAVMWYLQTQGKH----------LFG-------------------------------------------------
Zea_mays_6                         FITNVMWRNIVGQSIF--------QFVVIWYLQTQGEY----------LFG-------------------------------------------------
Aquilegia_caerulea_7               FITGIMWRNIIGQSIY--------QLAVLGVLKFDGKH----------LLK-------------------------------------------------
Oryza_sativa_7                     FITRIMWRNILGQGLY--------QLLVLATLMVIGKK----------LLS-------------------------------------------------
Selaginella_moellendorffii_8       FISTVMWRNIAVQVVY--------QLVVLNVLLYKGKD----------ILG-------------------------------------------------
Selaginella_moellendorffii_9       FISPVMCRNVLAQSVF--------QLVVLIVLQYRGLE----------IFG-------------------------------------------------
Oryza_sativa_8                     LISNAMWRNLAAQAAY--------QVAVLLALQYRGFG--------------------------------------------------------------
Fragilariopsis_cylindrus_13        LISLPMWRNILIQSTF--------QLSLLVYLLKNGPK----------LFN-------------------------------------------------
Pseudo-nitzschia_multiseries_4     LISRPMWRNILIQSTY--------QLTLLVYLLNKGPT----------LYN-------------------------------------------------
Phaeodactylum_tricornutum_4        LISRPMWRNILCQAVF--------QLSLLVFLLNKGPA----------MFE-------------------------------------------------
Thalassiosira_pseudonana_6         IIATRMWANMLGQALY--------QIAVVMFLLFGGAE----------AFG-------------------------------------------------
Phaeodactylum_tricornutum_5        MITKHMWANMLGQAAY--------QITVIMVLLFAGPE----------LLG-------------------------------------------------
Phaeodactylum_tricornutum_6        MITKHMWANMLGQATY--------QITVVMVLLFPGPD----------LLD-------------------------------------------------
```

```
                                  1801
Skeletonema_marinoi_ABC           --------------------QGVTLHQL---------------------------------------------------------SSWGKC----NQVW-
Thalassiosira_pseudonana_1        --------------------QGLTLRQL---------------------------------------------------------SSWGKC----DQTW-
Thalassiosira_oceanica_1          --------------------QGISLKQL---------------------------------------------------------RSWGKC----DQSW-
Phaeodactylum_tricornutum_1       --------------------QGITLAQL---------------------------------------------------------ATWSQC----GEFW-
Fragilariopsis_cylindrus_1        --------------------QGITLKQL---------------------------------------------------------SNWSNC---------
Fragilariopsis_cylindrus_2        --------------------QGITLKQL---------------------------------------------------------SNWSNC---------
Fragilariopsis_cylindrus_3        --------------------QGITLKQL---------------------------------------------------------SNWSNC----GTTWM
Fragilariopsis_cylindrus_4        --------------------QGITLKQL---------------------------------------------------------SNWSNC----GTTWM
Fragilariopsis_cylindrus_5        --------------------QGITLKQL---------------------------------------------------------SNWSNC----GTTWM
Fragilariopsis_cylindrus_6        --------------------QGITLKQL---------------------------------------------------------SNWSNC----GTTWM
Fragilariopsis_cylindrus_7        --------------------QGITLKQL---------------------------------------------------------SNWSNC----GTTWM
Fragilariopsis_cylindrus_8        --------------------QGITLKQL---------------------------------------------------------SNWSNC---------
Pseudo-nitzschia_multiseries_1    --------------------QGISLKQL---------------------------------------------------------SNWSNC----GTDW-
Thalassiosira_pseudonana_2        --------------------QGINVQEL---------------------------------------------------------SSWSSCS--------
Ostreococcus_lucimarinus_1        ------------------GHTPVTFKQL---------------------------------------------------------TNWGEC-----ASW-
Zea_mays_1                        ------------------GHTLVSYSQL---------------------------------------------------------SNWDKC-----SSW-
Oryza_sativa_1                    ------------------GHSLVSYSQL---------------------------------------------------------SNWGQC-----SSW-
Aquilegia_caerulea_1              ------------------GHTLVTYSQL---------------------------------------------------------SNWGQC-----SSW-
A.thaliana_AT1G07810.1            ------------------GHSLVSYSQL---------------------------------------------------------AHWGQC-----SSW-
A.thaliana_AT1G07670.1            ------------------GHSLVSYSQL---------------------------------------------------------AHWGQC-----SSW-
Medicago_truncatula_1             ------------------GHTLVTYSQL---------------------------------------------------------ANWGQC-----SSW-
Glycine_max_1                     ------------------GHTLVTYTQL---------------------------------------------------------ANWGQC-----SSW-
Glycine_max_2                     ------------------GHSLVTYTQL---------------------------------------------------------ANWGQC-----SSW-
Aquilegia_caerulea_2              ------------------GHSLVTYSQL---------------------------------------------------------SNWGQC-----PSW-
Selaginella_moellendorffii_1      ------------------GHTLVTFSQL---------------------------------------------------------TSWGEC-----PSW-
Physcomitella_patens_1            ------------------GHTLVSFSQL---------------------------------------------------------THWGEC-----STW-
Physcomitella_patens_2            ------------------GHTLVSFSQL---------------------------------------------------------THWGEC-----STW-
Physcomitella_patens_3            ------------------GHTLVSFSQL---------------------------------------------------------RNWGDC-----PNW-
Selaginella_moellendorffii_2      ------------------GHSLVTFSQL---------------------------------------------------------THWGEC-----PTW-
Glycine_max_3                     ------------------GHTIIELSQL---------------------------------------------------------RNWGEC-----PSW-
Glycine_max_4                     ------------------GHTIIELSQL---------------------------------------------------------RNWGEC-----PSW-
Aquilegia_caerulea_3              ------------------GHTLVTLYQL---------------------------------------------------------RTWGQC-----PSW-
A.thaliana_AT4G00900.1            ------------------GHTLVSFTQL---------------------------------------------------------QNWSEC-----SSWG
Chlamydomonas_reinhardtii_1       ------------------GHSTVTWEQL---------------------------------------------------------TNWQSC-----REW-
Volvox_carteri_1                  ------------------GHTTVTWEQL---------------------------------------------------------TNWQQC-----REW-
Micromonas_pusilla_1              ------------------GHTTVTWHQL---------------------------------------------------------SHWGDC-----ASWG
Micromonas_sp_1                   ------------------GHTPVTWHQL---------------------------------------------------------THWGEC-----ETW-
Selaginella_moellendorffii_3      ------------------GHTAVSFQQL---------------------------------------------------------SHWGEC-----PLW-
Thalassiosira_oceanica_2          ------------------DHTNITWEQL---------------------------------------------------------SNWGHC-----STW-
Thalassiosira_pseudonana_3        ------------------DHTNISWEQL---------------------------------------------------------TGWGHC-----STW-
Pseudo-nitzschia_multiseries_2    ------------------GHSMVTYGQL---------------------------------------------------------TRWGQC-----RTGE
Fragilariopsis_cylindrus_9        ------------------GHSMVSYAQL---------------------------------------------------------THWGQC-----RTGS
Fragilariopsis_cylindrus_10       ------------------GHSMVSYAQL---------------------------------------------------------THWGQC-----RTGS
Aquilegia_caerulea_4              -------------------GPKLPYYEL---------------------------------------------------------INFDTC-----PARE
Glycine_max_5                     -------------------GPKLPYTEL---------------------------------------------------------MNFDTC-----PTRE
Medicago_truncatula_2             -------------------GPKLPYTEL---------------------------------------------------------MNFDTC-----PTRE
A.thaliana_AT1G10130.1            -------------------GPKLTYSEL---------------------------------------------------------MNFETC-----ALRE
Oryza_sativa_2                    -------------------GPRLPYSELARKPLLHALLLARKWDKMMWSNNHTSSYQQK---------PISLIKEI---PSIGPQVNFDSC-----STRQ
Selaginella_moellendorffii_4      -------------------GPRLTWKEL---------------------------------------------------------VSFNEC-----KDGT
Micromonas_pusilla_2              -------------------GPMLTWSEL---------------------------------------------------------TSFESC-----EEGK
Micromonas_sp_2                   -------------------GPLMTWHQL---------------------------------------------------------TSFTEC-----VEGV
Volvox_carteri_2                  -------------------GGNLTWSQL---------------------------------------------------------TAFQKCT--EPSAKA
Chlamydomonas_reinhardtii_2       -------------------GGGLTWGQL---------------------------------------------------------TSFQKCT--EASAKA
Dunaliella_salina_1               -------------------GGNMTWSQL---------------------------------------------------------THFQSC-----ASQP
Ostreococcus_lucimarinus_2        -------------------GPQMTWAEL---------------------------------------------------------TSASRCI--------
Phaeodactylum_tricornutum_2       -------------------------NEL------------------------------------------------------------------------
Chlamydomonas_reinhardtii_3       ----------------------------------------------------------------------------------------------------
Chlamydomonas_reinhardtii_4       ----------------------------------------------------------------------------------------------------
Thalassiosira_pseudonana_4        ----------------------------------------------------------------------------------------------------
Thalassiosira_pseudonana_5        ----------------------------------------------------------------------------------------------------
```

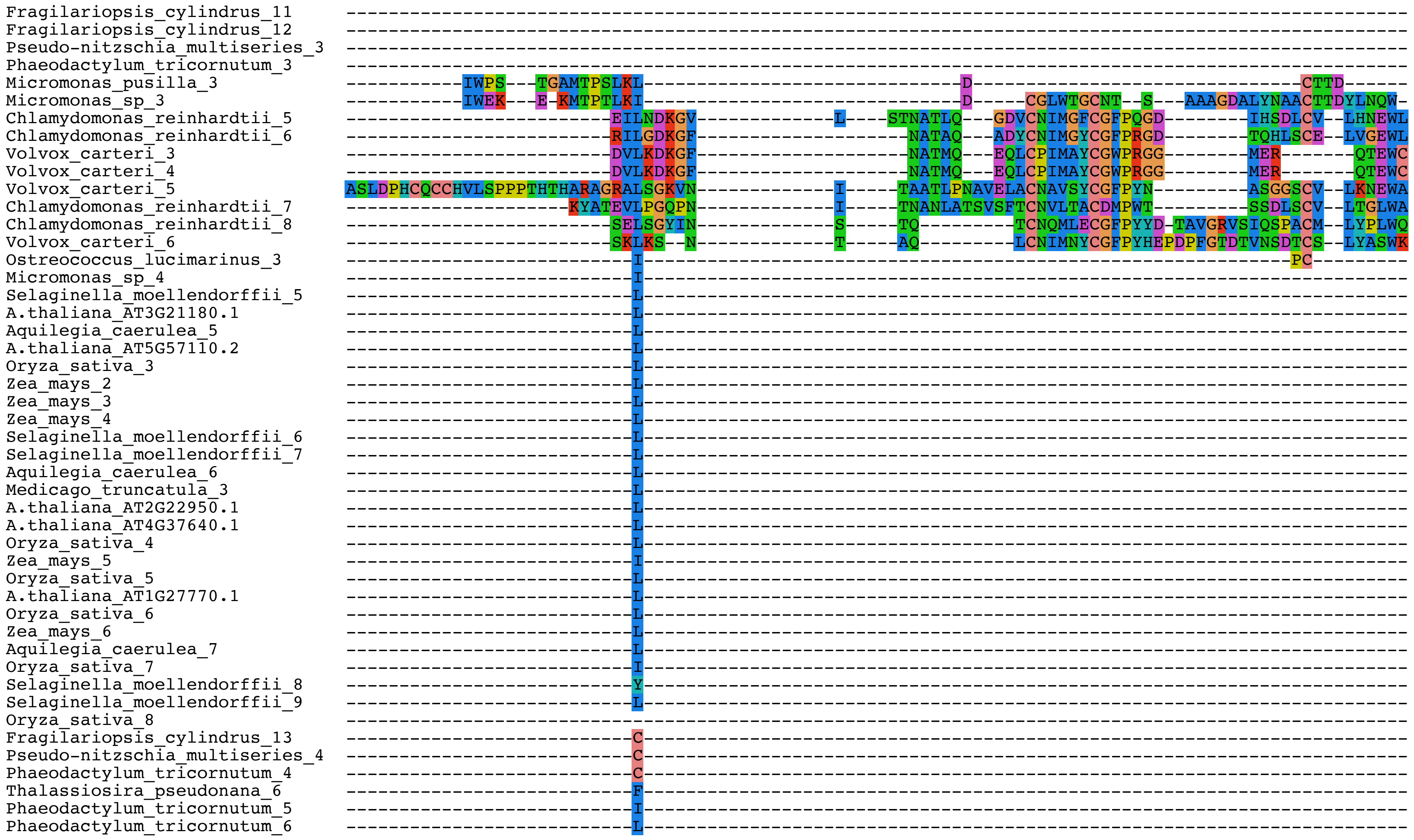
Fragilariopsis_cylindrus_11
Fragilariopsis_cylindrus_12
Pseudo-nitzschia_multiseries_3
Phaeodactylum_tricornutum_3
Micromonas_pusilla_3
Micromonas_sp_3
Chlamydomonas_reinhardtii_5
Chlamydomonas_reinhardtii_6
Volvox_carteri_3
Volvox_carteri_4
Volvox_carteri_5
Chlamydomonas_reinhardtii_7
Chlamydomonas_reinhardtii_8
Volvox_carteri_6
Ostreococcus_lucimarinus_3
Micromonas_sp_4
Selaginella_moellendorffii_5
A.thaliana_AT3G21180.1
Aquilegia_caerulea_5
A.thaliana_AT5G57110.2
Oryza_sativa_3
Zea_mays_2
Zea_mays_3
Zea_mays_4
Selaginella_moellendorffii_6
Selaginella_moellendorffii_7
Aquilegia_caerulea_6
Medicago_truncatula_3
A.thaliana_AT2G22950.1
A.thaliana_AT4G37640.1
Oryza_sativa_4
Zea_mays_5
Oryza_sativa_5
A.thaliana_AT1G27770.1
Oryza_sativa_6
Zea_mays_6
Aquilegia_caerulea_7
Oryza_sativa_7
Selaginella_moellendorffii_8
Selaginella_moellendorffii_9
Oryza_sativa_8
Fragilariopsis_cylindrus_13
Pseudo-nitzschia_multiseries_4
Phaeodactylum_tricornutum_4
Thalassiosira_pseudonana_6
Phaeodactylum_tricornutum_5
Phaeodactylum_tricornutum_6

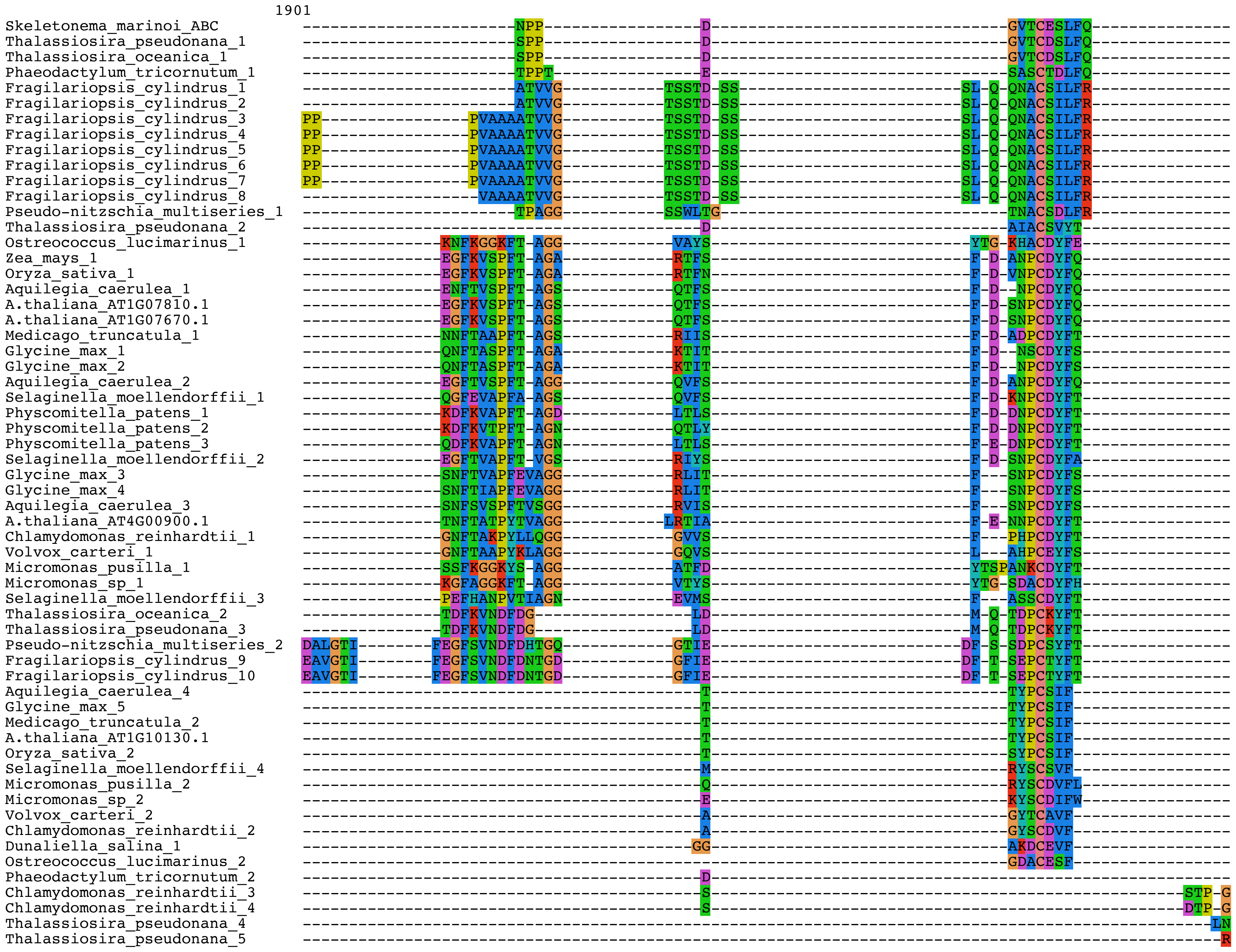
1901
Skeletonema_marinoi_ABC
Thalassiosira_pseudonana_1
Thalassiosira_oceanica_1
Phaeodactylum_tricornutum_1
Fragilariopsis_cylindrus_1
Fragilariopsis_cylindrus_2
Fragilariopsis_cylindrus_3
Fragilariopsis_cylindrus_4
Fragilariopsis_cylindrus_5
Fragilariopsis_cylindrus_6
Fragilariopsis_cylindrus_7
Fragilariopsis_cylindrus_8
Pseudo-nitzschia_multiseries_1
Thalassiosira_pseudonana_2
Ostreococcus_lucimarinus_1
Zea_mays_1
Oryza_sativa_1
Aquilegia_caerulea_1
A.thaliana_AT1G07810.1
A.thaliana_AT1G07670.1
Medicago_truncatula_1
Glycine_max_1
Glycine_max_2
Aquilegia_caerulea_2
Selaginella_moellendorffii_1
Physcomitella_patens_1
Physcomitella_patens_2
Physcomitella_patens_3
Selaginella_moellendorffii_2
Glycine_max_3
Glycine_max_4
Aquilegia_caerulea_3
A.thaliana_AT4G00900.1
Chlamydomonas_reinhardtii_1
Volvox_carteri_1
Micromonas_pusilla_1
Micromonas_sp_1
Selaginella_moellendorffii_3
Thalassiosira_oceanica_2
Thalassiosira_pseudonana_3
Pseudo-nitzschia_multiseries_2
Fragilariopsis_cylindrus_9
Fragilariopsis_cylindrus_10
Aquilegia_caerulea_4
Glycine_max_5
Medicago_truncatula_2
A.thaliana_AT1G10130.1
Oryza_sativa_2
Selaginella_moellendorffii_4
Micromonas_pusilla_2
Micromonas_sp_2
Volvox_carteri_2
Chlamydomonas_reinhardtii_2
Dunaliella_salina_1
Ostreococcus_lucimarinus_2
Phaeodactylum_tricornutum_2
Chlamydomonas_reinhardtii_3
Chlamydomonas_reinhardtii_4
Thalassiosira_pseudonana_4
Thalassiosira_pseudonana_5

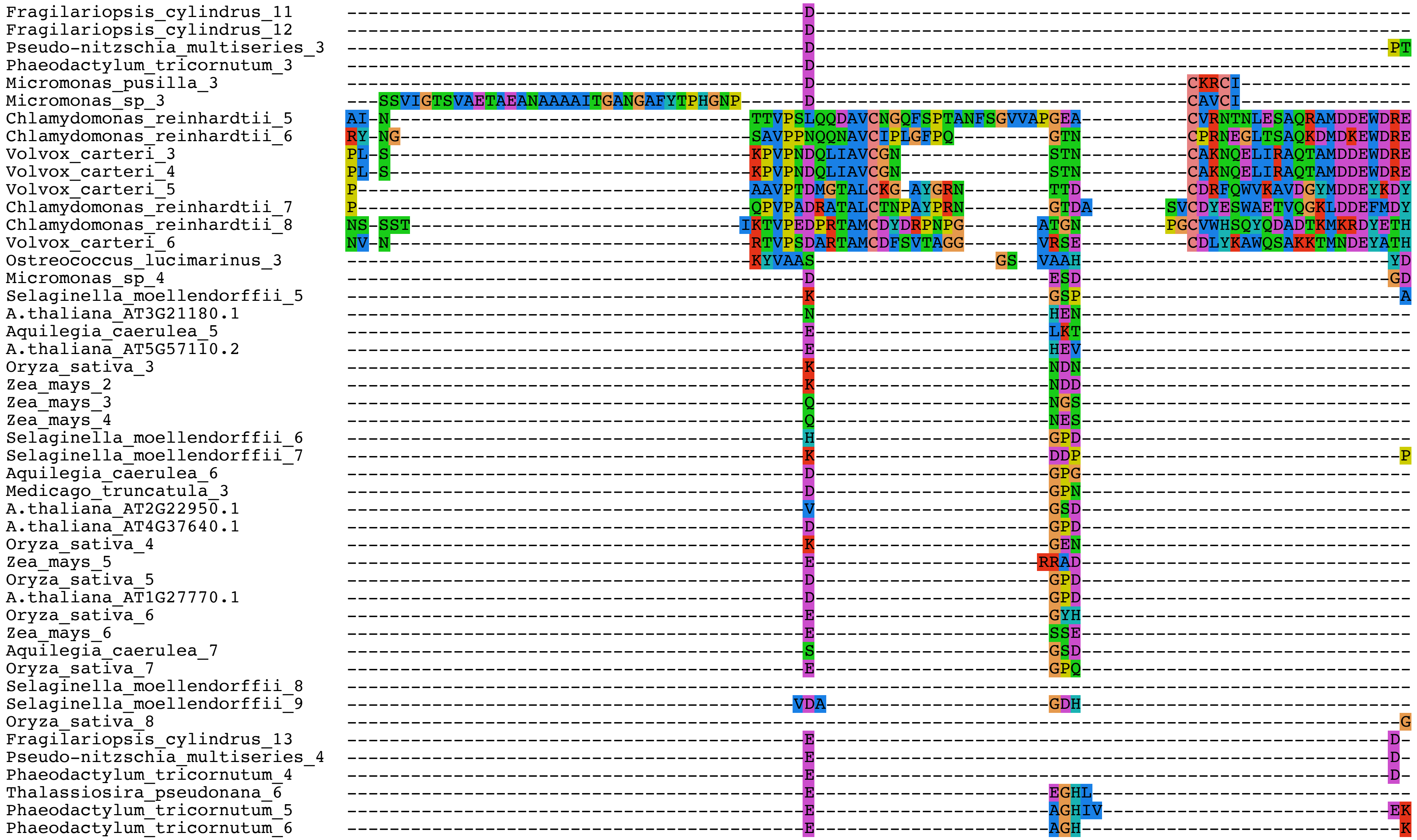

Fragilariopsis_cylindrus_11
Fragilariopsis_cylindrus_12
Pseudo-nitzschia_multiseries_3
Phaeodactylum_tricornutum_3
Micromonas_pusilla_3
Micromonas_sp_3
Chlamydomonas_reinhardtii_5
Chlamydomonas_reinhardtii_6
Volvox_carteri_3
Volvox_carteri_4
Volvox_carteri_5
Chlamydomonas_reinhardtii_7
Chlamydomonas_reinhardtii_8
Volvox_carteri_6
Ostreococcus_lucimarinus_3
Micromonas_sp_4
Selaginella_moellendorffii_5
A.thaliana_AT3G21180.1
Aquilegia_caerulea_5
A.thaliana_AT5G57110.2
Oryza_sativa_3
Zea_mays_2
Zea_mays_3
Zea_mays_4
Selaginella_moellendorffii_6
Selaginella_moellendorffii_7
Aquilegia_caerulea_6
Medicago_truncatula_3
A.thaliana_AT2G22950.1
A.thaliana_AT4G37640.1
Oryza_sativa_4
Zea_mays_5
Oryza_sativa_5
A.thaliana_AT1G27770.1
Oryza_sativa_6
Zea_mays_6
Aquilegia_caerulea_7
Oryza_sativa_7
Selaginella_moellendorffii_8
Selaginella_moellendorffii_9
Oryza_sativa_8
Fragilariopsis_cylindrus_13
Pseudo-nitzschia_multiseries_4
Phaeodactylum_tricornutum_4
Thalassiosira_pseudonana_6
Phaeodactylum_tricornutum_5
Phaeodactylum_tricornutum_6

```
                                2001
Skeletonema_marinoi_ABC          -GVGRELPQTLSLTVLVLME-----------------------------------LLKALSAV-SVN-SSIF---TVGPNQNPWLVAGVALPFALHLAVV
Thalassiosira_pseudonana_1       -GAGRELPQTLSLTVLVCME-----------------------------------LFKALSAV-SVD-SSLL---SVGPNQNPWLMIGVAVPFLLHIAVV
Thalassiosira_oceanica_1         -GVGRELPQTLSLTVLVCME-----------------------------------LFKALSAV-SVD-SSLL---AVGPNQNPWLVAGVALPFLLHVAVI
Phaeodactylum_tricornutum_1      -GSARMLPQTLALTTLVCME-----------------------------------MLKALSAV-SMD-DSIF---RVGPQENKWLILGVSGPFLLHLMVL
Fragilariopsis_cylindrus_1       -NNGRILPQTLSLTTLVVME-----------------------------------MLKALSAV-SVN-DSLL---KVPPWKNKYLLVGVSGPFLLHILVL
Fragilariopsis_cylindrus_2       -NNGRILPQTLSLTTLVVME-----------------------------------MLKALSAV-SVN-DSLL---KVPPWKNKYLLVGVSGPFLLHILVL
Fragilariopsis_cylindrus_3       -NNGRILPQTLSLTTLVVME-----------------------------------MLKALSAV-SVN-DSLL---KVPPWKNKYLLVGVSGPFLLHILVL
Fragilariopsis_cylindrus_4       -NNGRILPQTLSLTTLVVME-----------------------------------MLKALSAV-SVN-DSLL---KVPPWKNKYLLVGVSGPFLLHILVL
Fragilariopsis_cylindrus_5       -NNGRILPQTLSLTTLVVME-----------------------------------MLKALSAV-SVN-DSLL---KVPPWKNKYLLVGVSGPFLLHILVL
Fragilariopsis_cylindrus_6       -NNGRILPQTLSLTTLVVME-----------------------------------MLKALSAV-SVN-DSLL---KVPPWKNKYLLVGVSGPFLLHILVL
Fragilariopsis_cylindrus_7       -NNGRILPQTLSLTTLVVME-----------------------------------MLKALSAV-SVN-DSLL---KVPPWKNKYLLVGVSGPFLLHILVL
Fragilariopsis_cylindrus_8       -NNGRILPQTLSLTTLVVME-----------------------------------MLKALSAV-SVN-DSLL---KVPPWKNKYLLVGVSGPFLLHILVL
Pseudo-nitzschia_multiseries_1   -EAGRTLPQTLSLTTLVCME-----------------------------------MLKALSAV-SMN-DSLF---KVAPWQNKWLLMGVAGPFLLHLSVL
Thalassiosira_pseudonana_2       ---DLATPQTLALTTLVTTE-----------------------------------LLKALCTV-SVD-SSIL---TVPPQKNPWLILGVAVPFALNLGII
Ostreococcus_lucimarinus_1       --AGKVKASTLSLTVLVAIE-----------------------------------MFNALNAL-SED-GSLV---TMPPWRNPYLLIAMLVSFGSHFLIM
Zea_mays_1                       --GGKIKATTLSLSVLVAIE-----------------------------------MFNSLNAL-SED-GSLV---SMPPWVNPWLLLAMSVSFGLHFLIL
Oryza_sativa_1                   --GGKIKATTLSLSVLVAIE-----------------------------------MFNSLNAL-SED-GSLL---SMPPWVNPWLLLAMSVSFGLHFLIL
Aquilegia_caerulea_1             --TGKVKAMTLSLSVLVAIE-----------------------------------MFNSLNAL-SED-GSLL---VMPPWVNPWLLVAMSVSFGLHFLIL
A.thaliana_AT1G07810.1           --QGKIKASTLSLSVLVAIE-----------------------------------MFNSLNAL-SED-GSLV---TMPPWVNPWLLLAMAVSFGLHFVIL
A.thaliana_AT1G07670.1           --QGKIKASTLSLSVLVAIE-----------------------------------MFNSLNAL-SED-GSLV---TMPPWVNPWLLLAMAVSFGLHFVIL
Medicago_truncatula_1            --TGKVKAMTLSLSVLVAIE-----------------------------------MFNSLNAL-SED-GSLL---TMPPWVNPWLLLAMSVSFGLHFLIL
Glycine_max_1                    --TGKVKAMTLSLSVLVAIE-----------------------------------MFNSLNAL-SED-GSLL---TMPPWVNPWLLLAMSVSFGLHFLIL
Glycine_max_2                    --TGKVKAMTLSLSVLVAIE-----------------------------------MFNSLNAL-SED-GSLL---TMPPWVNPWLLLAMSVSFGLHFLIL
Aquilegia_caerulea_2             --AGKVKAMTLSLSVLVSIE-----------------------------------MFNSLNAL-SED-GSLL---VMPPWVNPWLLVAMSVSFGLHFLIL
Selaginella_moellendorffii_1     --DGKVKASTLSLSVLVAIE-----------------------------------MFNSLNAL-SED-GSLL---VMPPWVNPWLLLAMSVSFGLHFLIL
Physcomitella_patens_1           --TGKVKATTLSLSVLVAIE-----------------------------------MFNSLNAL-SED-GSLV---SMPPWVNPWLLLAMGLSFSLHFLIL
Physcomitella_patens_2           --TGKVKATTLSLSVLVAIE-----------------------------------MFNSLNAL-SED-GSLV---SMPPWVNPWLLLAMAVSFSLHFLIL
Physcomitella_patens_3           --TGKVKATTLSLSVLVAIE-----------------------------------MFNSLNAL-SED-GSLL---QIPPWMNPWLLIAMLFSFSLHFLVL
Selaginella_moellendorffii_2     --AGKVKATTLSLSVLVAIE-----------------------------------MFNSLNAL-SED-GSLL---VMPPWRNPWLLVAMAASLGLHFIIL
Glycine_max_3                    --VGKLKAMTLSLSVLVAIE-----------------------------------MFNSLNAL-SEE-NSLR---KLPPWRNPWLLVAMSISFGLHCLIL
Glycine_max_4                    --VGKVKAMTLSLSVLVAIE-----------------------------------MFNSLNAL-SEE-NSLR---KLPPWRNPWLLVAMSISLGLHCLIL
Aquilegia_caerulea_3             --VGKVKAMTLSLSVLVAIE-----------------------------------MFNSLNAL-SED-NSLV---TMPPWRNPLLLLAMSISFGFHCLIL
A.thaliana_AT4G00900.1           --LGKVKPMTLSLTVLVAIE-----------------------------------MFNSLNAL-SED-NSLL---TMPPWRNPWLLVAMTVSFALHCVIL
Chlamydomonas_reinhardtii_1      --AGKAKASTLSLSVIVAIE-----------------------------------MFNALNAL-SED-GSLL---TMPPWANPWLLLAIAISISLHCVIL
Volvox_carteri_1                 --VGKAKASTLSLSVIVAIE-----------------------------------MFNALNAL-SED-GSLL---AMPPWSNPWLLTAISVSVALHCVIL
Micromonas_pusilla_1             --EGKAKASTLSLTTLVVIE-----------------------------------MFNACNAL-SED-ISLF---VMPPWINPWLMVAMFSSFALHFLIL
Micromonas_sp_1                  --AGKIKASTLSLTTLVVIE-----------------------------------MFNACNAL-SED-ISLV---IMPPWINPWLILAMFSSFALHFLIL
Selaginella_moellendorffii_3     --VGKLKPSTLAMSTLVMIE-----------------------------------MFNALNAL-SET-NSLL---KVRPWANKWLLVAIAVSLGLHGTIL
Thalassiosira_oceanica_2         --DGKAKASTLSLSVLVAIE-----------------------------------MFNALNAL-SED-GSLV---TMPPWANPYLLLAMVVSFGMHFVIL
Thalassiosira_pseudonana_3       --DGKVKASTMSLSVLVAIE-----------------------------------MFNALNAL-SED-GSLV---TMPPWSNPYLLLAMVVSFGMHFVIL
Pseudo-nitzschia_multiseries_2   --KGKMKASTLSLSVLVTIE-----------------------------------MFNALNAV-SED-GSLL---TIPPWVNPYLLLAMASSFILHFMIL
Fragilariopsis_cylindrus_9       --KGKMKASTLSLSVLVTIE-----------------------------------MFNALNAV-SED-GSLL---TIPPWVNPYLLLAMASSFIMHFLIL
Fragilariopsis_cylindrus_10      --KGKMKASTLSLSVLVTIE-----------------------------------MFNALNAV-SED-GSLL---TIPPWVNPYLLLAMASSFIMHFLIL
Aquilegia_caerulea_4             ---DDRRPSTVSMTVLVVVE-----------------------------------MFNALNNL-SEN-QSLL---VIAPWSNLWLVGSIILTMLLHILIL
Glycine_max_5                    ---DDRHPSTVSMTVLVVVE-----------------------------------MFNALNNL-SEN-QSLL---VIPPWSNLWLVASIILTMLLHMLIL
Medicago_truncatula_2            ---EDRHPSTVAMTVLVVVE-----------------------------------MFNALNNL-SEN-QSLL---VIPPWSNLWLVGSIVLTMLLHILIL
A.thaliana_AT1G10130.1           ---EDRHPSTVAMTVLVVVE-----------------------------------MFNALNNL-SEN-QSLL---VITPRSNLWLVGSIILTMLLHVLIL
Oryza_sativa_2                   ---EDRHPSTVSMTVLVVVE-----------------------------------MFNALNNL-SEN-QSLL---AIHPWSNLWLVGSIVLTMLLHISVL
Selaginella_moellendorffii_4     ---RDRHPSTISMSVLVVVE-----------------------------------MFNAMNNL-SEN-QSLL---VLPPWSNMWLMASIGVSMLLHFLIL
Micromonas_pusilla_2             ---KNRSPSTMSMSVLVVVE-----------------------------------MFNALNAL-SEN-GSLL---THPPWSNYWLLGAICVSMLLHCVIL
Micromonas_sp_2                  ---KNRGPSTVSMTILVVVE-----------------------------------MFNALNAL-SEN-ESLL---SQSPSSNPWLVGAIVISLLLHVAIL
Volvox_carteri_2                 ---ESQHPRTIAMSVLVVVE-----------------------------------MFNALNNL-SEN-SSLL---VIPPWDNRWLLGAIATSMALHFFIL
Chlamydomonas_reinhardtii_2      ---GSRHPRTIAMSVLVVVE-----------------------------------MFNALNNL-SED-ASLL---TIPPWDNRWLLGAIATSLGLHALIL
Dunaliella_salina_1              ---HSKHPTTISMSVLVVVE-----------------------------------MFNALNNL-SED-SSLL---RIPPWDNKWLVGAIATSMALHFGIL
Ostreococcus_lucimarinus_2       ---KDRRPSTMAMSTLVLIE-----------------------------------MFNALNSL-SEN-KSLF---SHPPTTNVWLLVSIVISMWLHFIIM
Phaeodactylum_tricornutum_2      DGAVNRRDTTMTFMMFCNCD-----------------------------------LFNAYVCR-SAD-KPFY---KINLYSNPAFLWAIGGSVVGQFLVV
Chlamydomonas_reinhardtii_3      GGYNLKQRRSEAFNVLVGAQ-----------------------------------IAYFVNCR-FIKLSCFH---PRVFFGNPIVYISIALVAGIMVFVT
Chlamydomonas_reinhardtii_4      GGYPLKQQRAEAFNTLVGAQ-----------------------------------VAYFVSCR-FLKLSSFH---PRVFFGNPIAYVSISIVAALMVFVT
Thalassiosira_pseudonana_4       AEDRLAMIRAVSFNVLDFGA-----------------------------------MSVMMSARFSYN-SSFH---PRVFKGNPAALASCAIVTVLQIALT
Thalassiosira_pseudonana_5       SDDKMMMMRAVSFNILDFGA-----------------------------------ISVTLSARFAYN-SSMT---LNILRGNPAVLFSVVTVTVLQVFLT
```

```
Fragilariopsis_cylindrus_11      YKTVTHMVHSQASNTLTFGA-----------------------------------CFVTFSARFNLL-SSFH---PRLFRGNVFTWYSFAIVTVLQLCIT
Fragilariopsis_cylindrus_12      YKTVTHMVHSQASNTLTFGA-----------------------------------CFVTFSARFNLL-SSFH---PRLFRGNVFTWYSFAIVTVLQLCIT
Pseudo-nitzschia_multiseries_3   SKDSINRVHSQASNTLTFGA-----------------------------------CFVTLSARFSYQ-NSLH---PRAFQDNVFVTYSLIIVAVLQLAIT
Phaeodactylum_tricornutum_3      LGYTLEETRSQALNTLSFGA-----------------------------------ISVTMSARFSRK-SAFH---ARTFQGNPIAWWSYMVMGLLQVFIT
Micromonas_pusilla_3             -TTSIRRARTTAFIALVYAE-----------------------------------NFRAYCSR-SFE-----NGVWVKPFSNMTMNKAIIFAQLALYFAL
Micromonas_sp_3                  -EESIRRARTATFISLVWAE-----------------------------------GVRAYCSR-SFE-----NPIWVNMFSNMSMNKAVLLAQVTLIIAL
Chlamydomonas_reinhardtii_5      YLLSYKPALSVLFNAFILAQ-----------------------------------VANAFVSR-RIGLELNF---FKGLAHSPIFNGIMVLITALQVLIM
Chlamydomonas_reinhardtii_6      YLKSYKPALSVLFNAFILAQ-----------------------------------VANAFVSR-RIQLEYNF---FKGLANSHIFNAIMVLITALQAIIM
Volvox_carteri_3                 LLKSYKPALSVLFNAFILAQ-----------------------------------VANAFVSR-RIGLELNF---FKGLPRSYIFNVIMVLITALQVVIM
Volvox_carteri_4                 LLKSYKPALSVLFNAFILAQ-----------------------------------VANAFVSR-RIGLELNF---FKGLPRSYIFNVIMVLITALQVVIM
Volvox_carteri_5                 NEDEYKRPLSLLFNIFICTQ-----------------------------------VANEINAR-RINDEYNV---FEGFFGNWIFLAVITITMGLQAIII
Chlamydomonas_reinhardtii_7      EIDEYKRPLSLLFNIFICTQ-----------------------------------VANEINAR-RINDEYDI---FSGLFTNWIFMAVLAITMGAQAIII
Chlamydomonas_reinhardtii_8      MHDEYIHVLSCLFNAFIFCQ-----------------------------------IFNEINAR-RINDEYTI---FVGLFTNPIFCSVIAITVVFQVIII
Volvox_carteri_6                 TDDEYRPVLSILFNAFIFCQ-----------------------------------IFNEINAR-RINDEYTI---FTGLFTNPIFVTVIAVTAVFQIIII
Ostreococcus_lucimarinus_3       CHHQTLELNGFIFNAFVFMQ-----------------------------------VFSEINSR-RIA-DVNV---FANIHNSPIFCGIIALTVGVQVLFI
Micromonas_sp_4                  GHYRTLRVNSVIFNAFVFMQ-----------------------------------IFSEINSR-KIS-EWNV---FEGIQNSPIFCFIIFLTIGTQAAFI
Selaginella_moellendorffii_5     Q--KNVLRNTIIFNSFVLCQ-----------------------------------VFNEINAR-KLQ-KLNV---LKGVFQSYLFCTVIGVTSVIQIVII
A.thaliana_AT3G21180.1           HAHAVEVKNTMIFNAFVMCQ-----------------------------------IFNEFNAR-KPD-EMNV---FRGVNKNPLFVAIVGVTFILQIIIV
Aquilegia_caerulea_5             ----DQAANTLIFNAFVLCQ-----------------------------------IFNEFNAR-KPD-EMNV---FGGITKNRLFMAIVGVTLVLQIVII
A.thaliana_AT5G57110.2           HEHATRVKNTIIFNAFVLCQ-----------------------------------AFNEFNAR-KPD-EKNI---FKGVIKNRLFMGIIVITLVLQVIIV
Oryza_sativa_3                   QAHADKVKNTFIFNTFVLCQ-----------------------------------VFNEFNAR-KPD-ELNI---FKGITGNHLFMAIVAITVVLQALIV
Zea_mays_2                       PAHADKVKNTFIFNTFVLCQ-----------------------------------VFNEFNSR-KPD-ELNI---FKGISGNHLFIGIIVITVILQALIV
Zea_mays_3                       WEHAEKTKNTFVFNAFVFCQ-----------------------------------IFNEFNAR-KPE-EKNV---FKGVANNHLFVAIVGATTVLQILII
Zea_mays_4                       RSDAEKITNTFIFNTFVFCQ-----------------------------------IFNEFNAR-KPE-EKNV---FKGVTKNHLFMGIIGITTVFQILII
Selaginella_moellendorffii_6     ---GNRKLNTIIFNSFVLCQ-----------------------------------LFNEVNSR-KPD-KLNV---FSGFFRNPLFCGVVSVTAVLQVIIV
Selaginella_moellendorffii_7     EGVAHEKLVCIIFNAFVFCQ-----------------------------------VFNEMNAR-NPE-KLNV---FKGFTSNRLFMGVILFTAIVQALLV
Aquilegia_caerulea_6             ---SDLILNTLIFNSFVFCQ-----------------------------------VFNEISSR-EME-KIDV---FEGILDNNVFVTVLSATCIFQIIII
Medicago_truncatula_3            ---SDLVLNTLIFNAFVFCQ-----------------------------------VFNEINSR-EME-KINV---FKGILDNYVFVGVISATIFFQIIIV
A.thaliana_AT2G22950.1           ---STLVLNTLIFNCFVFCQ-----------------------------------VFNEVSSR-EME-EIDV---FKGILDNYVFVVVIGATVFFQIIII
A.thaliana_AT4G37640.1           ---STLMLNTLIFNCFVFCQ-----------------------------------VFNEISSR-EME-EIDV---FKGILDNYVFVVVIGATVFFQIIII
Oryza_sativa_4                   ---SDLVLNTLIFNCFVFCQ-----------------------------------VFNEVSSR-EME-RINV---FEGILDNNVFVAVLGSTVIFQFIIV
Zea_mays_5                       ---SDLVLNTLIFNCFVFCQ-----------------------------------VFNEVSSR-EME-RVNV---LRGILDNNVFAMVLGSTVVFQFVIV
Oryza_sativa_5                   ---AEVVLNTIIFNSFVFCQ-----------------------------------VFNEISSR-EME-KINV---LRGILKNYVFLGVLTSTVVFQFIMV
A.thaliana_AT1G27770.1           ---SDLTLNTLIFNIFVFCQ-----------------------------------VFNEISSR-EME-KIDV---FKGILKNYVFVAVLTCTVVFQVIII
Oryza_sativa_6                   ---ADIVLNTIIFNTFVFCQ-----------------------------------VFNEISSR-EME-DINV---LRGMAGNSIFLGVLTGTIFFQFILV
Zea_mays_6                       ---ANTVLNTIIFNTFVFCQ-----------------------------------VFNEISSR-DME-EINV---LKGLPQNSIFMSILGGTIIFQFILV
Aquilegia_caerulea_7             ---ADSVLNTFIFNTFVFCQ-----------------------------------VFNEINSR-DME-KINV---FRGMFQSWVFIAVMIATVVFQVIII
Oryza_sativa_7                   ---SDKTINTLIFNSFVFCQESVLYFNGNFTILEKVSGGTLRYFLKEGKISLILTVFNEINCR-EME-KINV---LQGIFRNWIFVGILTATVIFQVIIV
Selaginella_moellendorffii_8     ---DTLTLNTLIFNVFVFCQ-----------------------------------VFNELNAR-DME-KLNV---FKHTFNNITFLLVILFTVVFQTILV
Selaginella_moellendorffii_9     ---GKLVLNTIIFNTFVFFQ-----------------------------------VFNEFNSR-EMD-KINV---FRHL-DNRFFLAIVTATVVFQVVLI
Oryza_sativa_8                   AGAGERANGTMIFNAFVLCQ-----------------------------------VFNEFNAR-EIE-RRNV---FAGVHRNRMFLGIVAVTVALQVVMV
Fragilariopsis_cylindrus_13      ---GSTHHFTILFNAFVFCQ-----------------------------------VFNEFNAR-EIGDVFDP---VGSLEKSPMFLVVIVFTVFSQWFIV
Pseudo-nitzschia_multiseries_4   ---GSIHHFTIIFNAFVFCQ-----------------------------------IFNEFNAR-EIGDVFDP---IGALGQSPMFVLVIVFTVISQWVIV
Phaeodactylum_tricornutum_4      ---GSRHHFTILFNAFVFCQ-----------------------------------VFNEFNAR-EIGDRFDP---LRSLSESPMFLLVIVFTMVAQWAIV
Thalassiosira_pseudonana_6       ---ENSIHYTLIFNTFVWMQ-----------------------------------LFNEINSR-NLEGEFNV---FRGIQRNPLFVGILLLTAMLQVVMV
Phaeodactylum_tricornutum_5      EREENSVHYTVIFNTFVWMQ-----------------------------------LFNEINSR-KLKGESNV---FKGVLDNYIFCTILLLTSCLQALIV
Phaeodactylum_tricornutum_6      DRDVNTVHYTLIFNAFVWMQ-----------------------------------LFNEINCR-KLKGEINV---FEGILNNPMFCGILFTTAVLQVLIV
```

```
                               2101
Skeletonema_marinoi_ABC        YSAEL------------GFP-GLAKS-FGLVP--LSLHDWEIALKWSAPILIVEEILKFFG----
Thalassiosira_pseudonana_1     YSSKL------------GLP-GLAKS-FGLVP--LSLHDWKTALKWSAPILIVEEMLKAAGRHR
Thalassiosira_oceanica_1       YSSKL------------GLP-GLAKS-FGLVS-------WARV-------------HRCSQKRV
Phaeodactylum_tricornutum_1    YSSDL------------GIP-ALGES-FGMVP--LSIDDWALVISWAAPILLVDEILKAIGRWV
Fragilariopsis_cylindrus_1     YSSKLSLGLGGSNKYL--------------VP--LTVEEWKTVLLWASPILLVDEILKCIGRRL
Fragilariopsis_cylindrus_2     YSSKLSLGLGGSNKYL--------------VP--LTVEEWKTVLLWASPILLVDEILKCIGRRL
Fragilariopsis_cylindrus_3     YSSKLSLGLGGSNKYLVGLS-GFGKA-FGMVP--LTVEEWKTVLLWASPILLVDEILKCIGRRL
Fragilariopsis_cylindrus_4     YSSKLSLGLGGSNKYLVGLS-GFGKA-FGMVP--LTVEEWKTVLLWASPILLVDEILKCIGRRL
Fragilariopsis_cylindrus_5     YSSKLSLGLGGSNKYLVGLS-GFGKA-FGMVP--LTVEEWKTVLLWASPILLVDEILKCIGRRL
Fragilariopsis_cylindrus_6     YSSKLSLGLGGSNKYLVGLS-GFGKA-FGMVP--LTVEEWKTVLLWASPILLVDEILKCIGR--
Fragilariopsis_cylindrus_7     YSSKLSLGLGGSNKYLVGLS-GFGKA-FGMVP--LTVEEWKTVLLWASPILLVDEILKCIGRRL
Fragilariopsis_cylindrus_8     YSSKLSLGLGGSNKYLVGLS-GFGKA-FGMVP--LTVEEWKTVLLWASPILLVDEILKCIGRRL
Pseudo-nitzschia_multiseries_1 YSSRL------------GLP-GFGKA-FGMVS--LTKEHWKTVLFWSTPILLVDELLKFIGRRL
Thalassiosira_pseudonana_2     YN-----------------P-ALSNS-FGLVP--LTEVDWIHVMLWSSPIVLIDEALKYASRQA
Ostreococcus_lucimarinus_1     YV-----------------P-YFAEI-FSIVP--LDFNEWMLVLLCAAPVCLIDEVLKVFGRVS
Zea_mays_1                     YV-----------------P-FLAQV-FGIVP--LSLNEWLLVVAVAFPVVLIDEVLKFVGRCL
Oryza_sativa_1                 YV-----------------P-FLAQV-FGIVP--LSFNEWLLVIAVAFPVVLIDEVLKFVGRCL
Aquilegia_caerulea_1           YV-----------------P-FLAQV-FGIVP--LSLNEWLLVLAVALPVILIDEVLKFVGRCT
A.thaliana_AT1G07810.1         YV-----------------P-FLAQV-FGIVP--LSLNEWLLVLAVSLPVILIDEVLKFVGRCT
A.thaliana_AT1G07670.1         YV-----------------P-FLAQV-FGIVP--LSLNEWLLVLAVSLPVILIDEVLKFVGRCT
Medicago_truncatula_1          YV-----------------P-FLAKV-FGIVP--LSFNEWLLVLAVALPVILIDEVLKFVGRCT
Glycine_max_1                  YV-----------------P-FLAQV-FGIVP--LSFNEWLLVLVVALPVILIDEILKFVGRCT
Glycine_max_2                  YV-----------------P-FLAQV-FGIVP--LSFNEWLLVLVVALPVILIDEILKFVGRCT
Aquilegia_caerulea_2           YV-----------------P-FLAQV-FGIVP--LSLNEWFLVLAVALPVILIDEVLKFVGRCT
Selaginella_moellendorffii_1   YV-----------------P-FLANI-FGIVP--LSFNEWLLVIIVSFPVILIDELLKFVGRKM
Physcomitella_patens_1         YI-----------------P-FLAKI-FGIVP--LSLLEWLLVLVVSLPVILIDEVLKFIGRRM
Physcomitella_patens_2         YI-----------------P-FLATI-FGIVP--LSLNEWLLVLVVSLPVILIDEGLKFIGRRM
Physcomitella_patens_3         YV-----------------P-VLASI-FGIVP--LNSNEWLFVFVLAFPVILIDELLKFIGRRM
Selaginella_moellendorffii_2   YV-----------------P-LLAQV-FGIVP--LSWREWLLVLVVSLPVILIDEVLKFLGRRM
Glycine_max_3                  YT-----------------P-FLAEV-FGVIP--LSLNEWFMVLLISAPVILIDEILKLVVRSQ
Glycine_max_4                  YT-----------------P-FLAEV-FGVIP--LSLNEWFMVLLISAPVILIDEILKLVVRSH
Aquilegia_caerulea_3           YV-----------------P-FLADV-FGIVP--LTLKEWVLVILVSAPVVVIDEVLKLVGRSR
A.thaliana_AT4G00900.1         YV-----------------P-FLANV-FGIVP--LSFREWFVVILVSFPVILIDEALKFIGRCR
Chlamydomonas_reinhardtii_1    YV-----------------P-FLADV-FAIVP--LSWPEWQLVLLWSAPVVLLDEVLKAVGRLL
Volvox_carteri_1               YV-----------------P-FLADV-FAIVP--LSWPEWQLVLLWSAPVILLDEVLKFVGRII
Micromonas_pusilla_1           YV-----------------P-ALATI-FSIVP--LDANEWALVCACAAPVWLIDEVLKFIGRNF
Micromonas_sp_1                YV-----------------P-ALATI-FSIVP--LDLNEWLLVCAAASPVWIIDEILKFIGRNF
Selaginella_moellendorffii_3   YT-----------------P-WLAEV-FGVVP--LDWNDWLLVLAFSFPVIPLDEALKLAGRVF
Thalassiosira_oceanica_2       YV-----------------D-SLADM-FNVTP--LDKDEWMLVLAFSLPVIFIDEILKFAGRQM
Thalassiosira_pseudonana_3     YV-----------------D-FLADM-FNVTP--LDWDEWKVVLAFSLPVIFIDEILKFVGRKM
Pseudo-nitzschia_multiseries_2 YV-----------------P-WMATI-FSISP--HDAVDWILVFLFSFPVIIIDEMLKYVGRGM
Fragilariopsis_cylindrus_9     YV-----------------P-AMATV-FSISP--HNTIDWILVFLFSFPVIILDEILKYIGRRR
Fragilariopsis_cylindrus_10    YV-----------------P-AMATV-FSISP--HNTIDWILVFLFSFPVIILDEILKYIGRRR
Aquilegia_caerulea_4           YV-----------------Q-PLSIL-FSVTP--LSWAEWTVVLYLSFPVLIIDEILKFFSRNS
Glycine_max_5                  YV-----------------H-PLSVL-FSVTP--LSWTDWTVVLYLSLPVIVIDEVLKFFSRNP
Medicago_truncatula_2          YV-----------------R-PLSVL-FSVTP--LSWADWMAVLYLSLPVIIIDEILKFFSRNP
A.thaliana_AT1G10130.1         YV-----------------H-PLAVL-FSVTP--LSWAEWTAVLYLSFPVIIIDELLKFLSRNT
Oryza_sativa_2                 YI-----------------E-PLSAL-FS--------------------VILIDEVLKFFSRSS
Selaginella_moellendorffii_4   YL-----------------R-PLSLL-FSVTP--LSWAEWKAVLWFSFPVILVDEVLKVLSRQT
Micromonas_pusilla_2           YV-----------------P-WLAST-FSVAP--LSTAEWNAVIKFSFPVILLDED--------
Micromonas_sp_2                YV-----------------P-WLATI-FSVTP--LSSHEWCAVFWFSVPVILVDETLKFVTRSG
Volvox_carteri_2               YV-----------------G-PAAAL-FGVTS--LNGAEWLAVLALSAPVVLLDELMKWISRRV
Chlamydomonas_reinhardtii_2    YA-----------------P-PAAAL-FGVTG--LSGAEWAAVVWLSAPVVLLDEVMKALSRNV
Dunaliella_salina_1            YT-------------------GASAM-FGVTG--LSFAEWAMVLQLSAPVVLVDEVMKAWSRRR
Ostreococcus_lucimarinus_2     YV-----------------P-SFAKT-FTITA--LNYEEWRAVFWFSIPVIFIDEVLKYVTRAH
Phaeodactylum_tricornutum_2    YW-----------------L-PLQKV-FQTEA--LTFYDIAYIVLLSSMVLWLDALRKTFFPSV
Chlamydomonas_reinhardtii_3    YV-----------------P-GVNGF-FHMTG-SMDGIQWARVVVCTAIVFVLVEVEKALVDPL
Chlamydomonas_reinhardtii_4    YV-----------------P-GVNGF-FHMSG--MDGIQWARVVVATFVVYVIVEVEKALVDPL
Thalassiosira_pseudonana_4     YI-----------------P-GLNSFVFSMNG--MDGFGWALVIASMVLVFVVMETEKAIRR--
Thalassiosira_pseudonana_5     YT-----------------P-GLNSFVFSMMG--MDGFGWALVIAAMVVVFAVMETEKAIRRNL
```

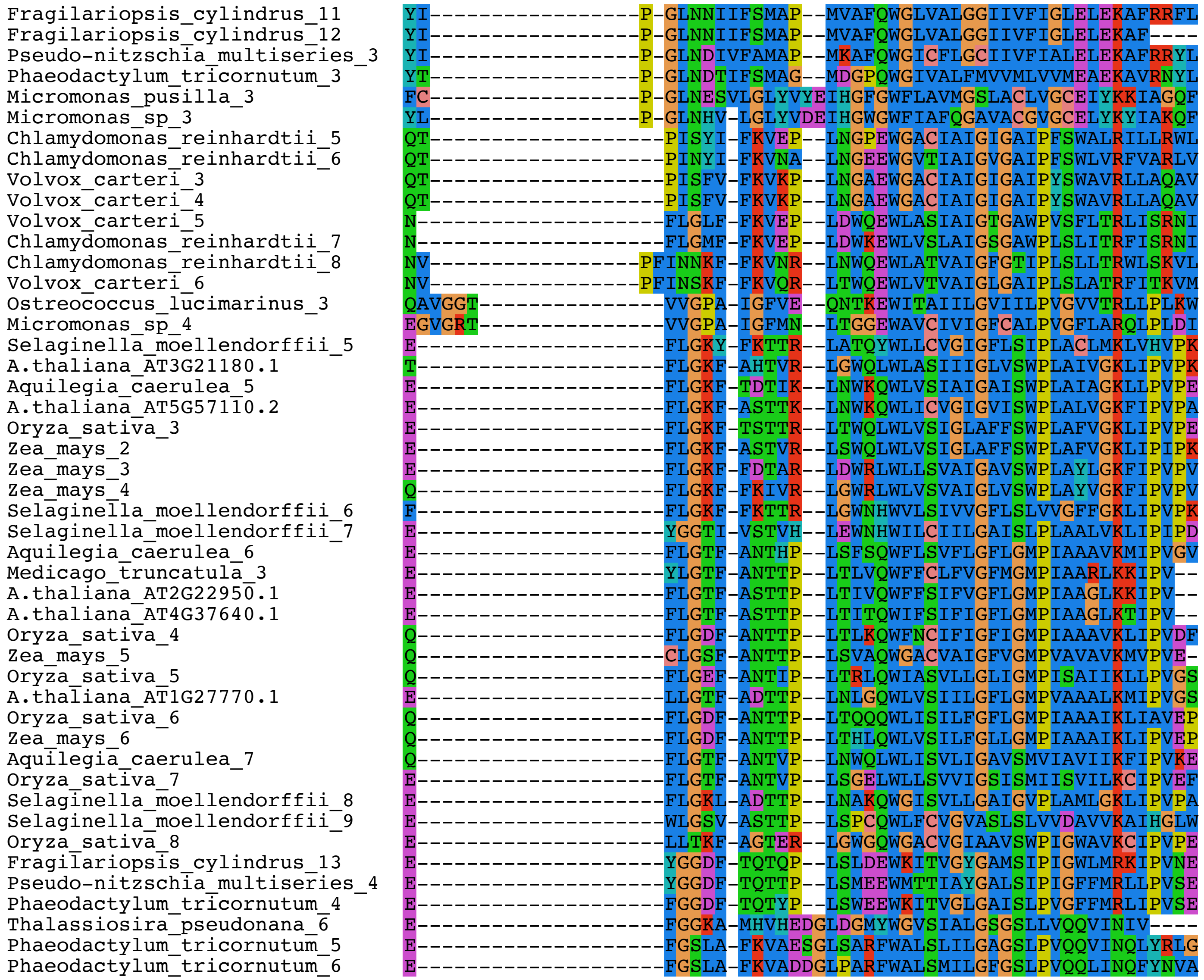

Supplementary figure 8.

Alignment of the ABC-transporter domains used in the phylogenetic analysis presented in supplementary figure 7. The length of the alignment has been adjusted to the length of the domain identified in *S. marinoi.* Numbers above the sequences indicate the positions in the alignment and the background color of the letters indicates size, shape, solubility, and ionization properties of the different amino acids.
